# Supplementary material for: The Impact of Melanoma Imaging Biomarker Cues on Detection Sensitivity and Specificity in Melanoma versus Clinically Atypical Nevi
Source: Cancers (Basel). 2024 Sep 4;16(17):3077. doi: 10.3390/cancers16173077 (PMC11394255; doi:10.3390/cancers16173077)
Supplement: Supplementary file 1 [file cancers-16-03077-s001.zip › cancers-3154863-supplementary/Supplementary File 1.pdf]

[image number 1 appears] I will be showing you a set of images and asking you to tell me whether the image is a melanoma or nevus. You'll see the image and answer: melanoma or nevus. Then you'll see that same image, with more information and again answer: melanoma or nevus. Then you'll get to see the corresponding pathology diagnosis, which is the right answer from the skin biopsy after the image you just screened.

This is not "would I biopsy?" …. Although you would biopsy most of these lesions, half are melanomas and half are nevi and your job is to predict, based on 3 factors. [image number 2 appears]

***The first factor*** is the ensemble classifier score, between 1 for melanoma and 0 for nevus. It's based on many imaging biomarkers. The number on the bottom right is a single imaging biomarker and that's the ***second of three factors*** you will consider. The third is your instinct, which is your best guess based on your training.

The single imaging biomarker on the bottom right the clock-sweep melanoma radar. It's the irregularity in brightness as a function of angle. Starting on the clock face at noon and going clockwise, we hit the darkest angle in blue, the brightest angle in green and the angle of max brightness variation in red. So given the risk score & clock sweep analysis, Is this a melanoma or nevus?

[image number 3 appears] Good, a nevus with a low score and low angular deviation. For each image, you need to quickly use these three factors (your instinct, the risk score, and the clock sweep analysis) to make a snap decision. Keep in mind that none of these three factors are going to be perfect on their own.
**First one "does this change your mind? Melanoma or nevus?**
