# Supplementary figures and images for: The Impact of Melanoma Imaging Biomarker Cues on Detection Sensitivity and Specificity in Melanoma versus Clinically Atypical Nevi (part 2 of 2)
Source: Cancers (Basel). 2024 Sep 4;16(17):3077. doi: 10.3390/cancers16173077 (PMC11394255; doi:10.3390/cancers16173077)

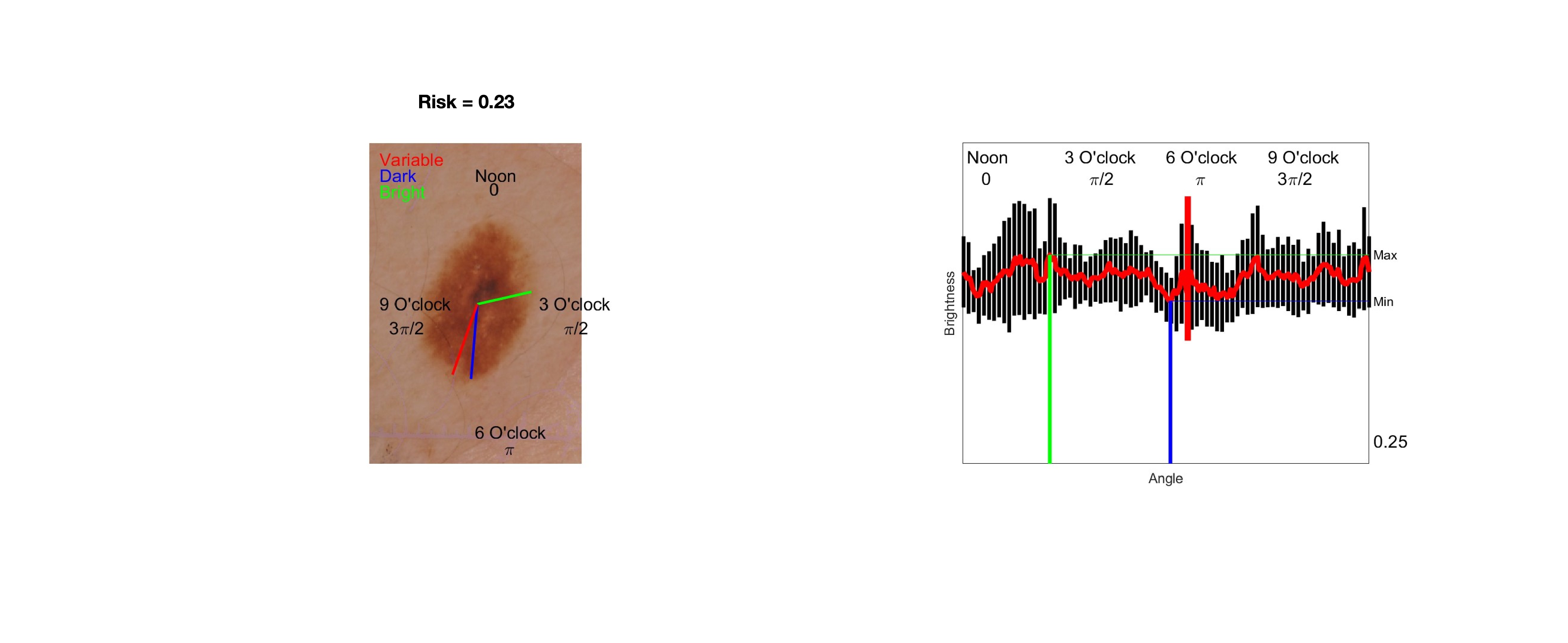

Supplement: Supplementary file 1 [file cancers-16-03077-s001.zip › cancers-3154863-supplementary/Supplementary File 2/034B.jpg]

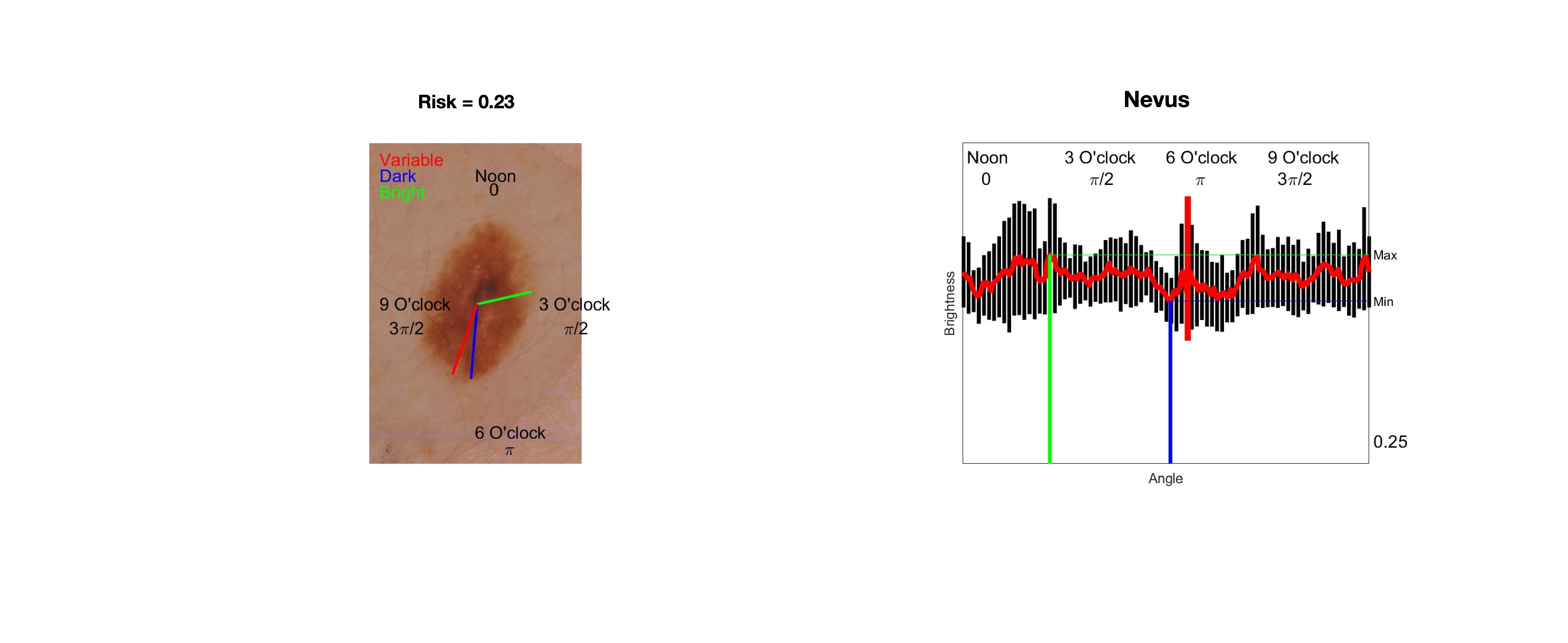

Supplement: Supplementary file 1 [file cancers-16-03077-s001.zip › cancers-3154863-supplementary/Supplementary File 2/034C.jpg]

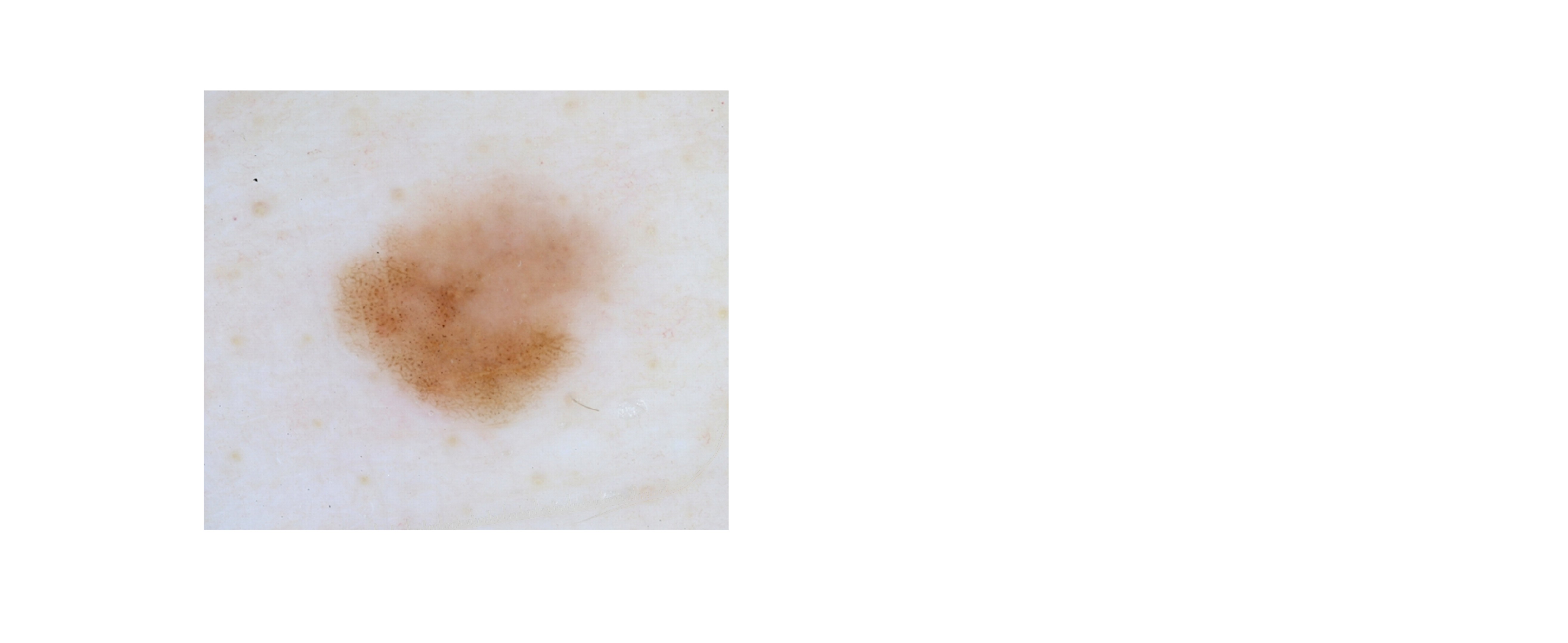

Supplement: Supplementary file 1 [file cancers-16-03077-s001.zip › cancers-3154863-supplementary/Supplementary File 2/035A.jpg]

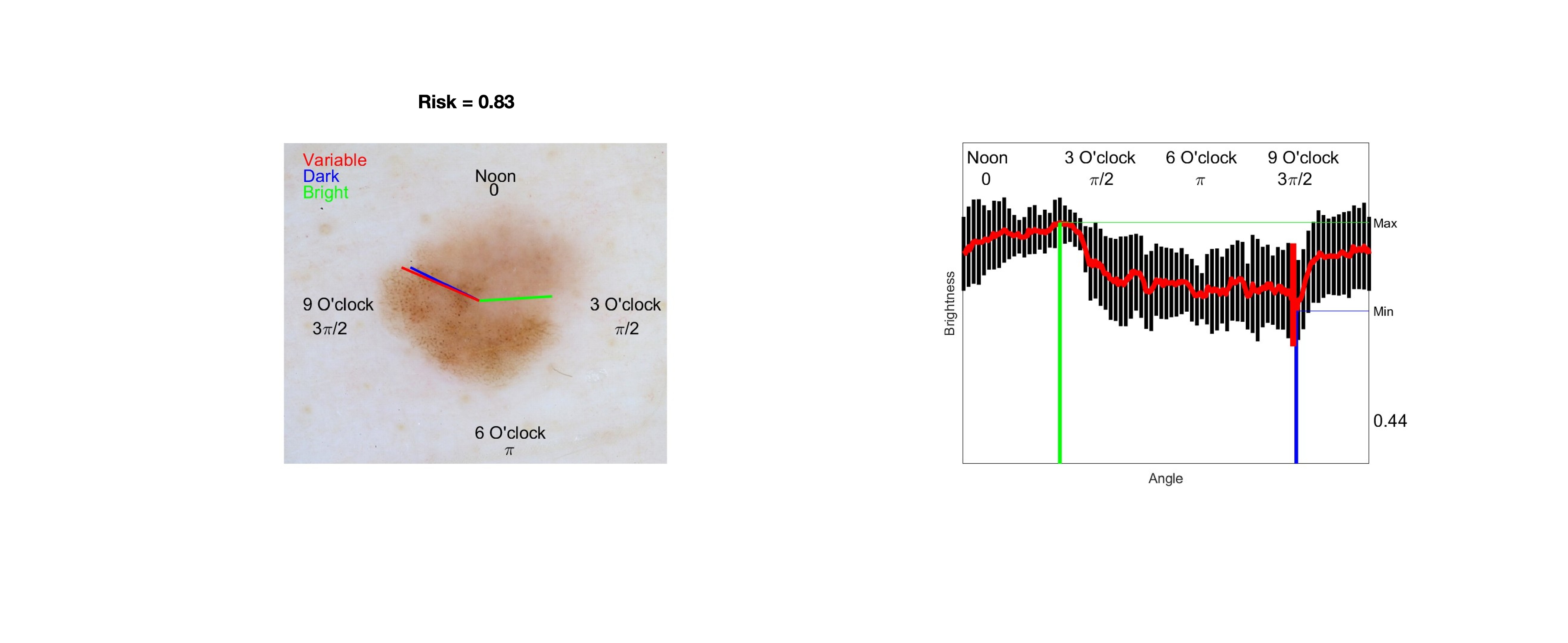

Supplement: Supplementary file 1 [file cancers-16-03077-s001.zip › cancers-3154863-supplementary/Supplementary File 2/035B.jpg]

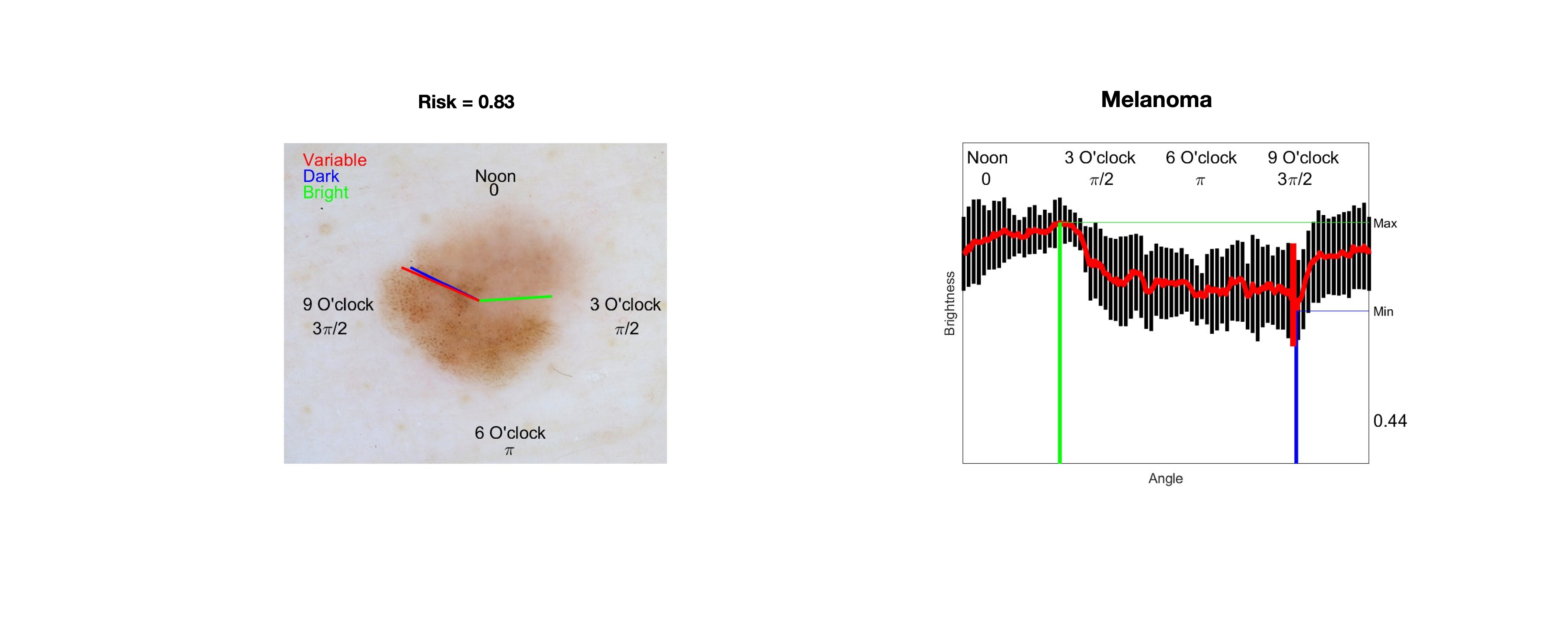

Supplement: Supplementary file 1 [file cancers-16-03077-s001.zip › cancers-3154863-supplementary/Supplementary File 2/035C.jpg]

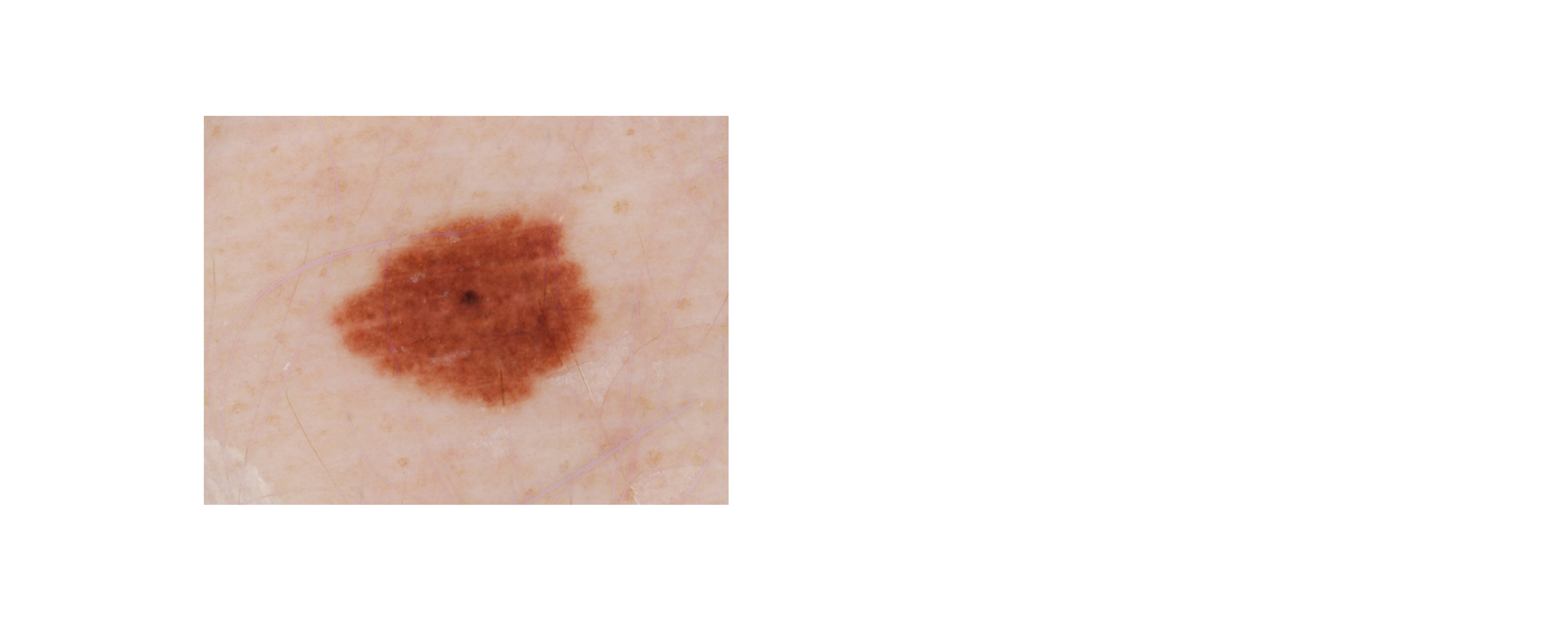

Supplement: Supplementary file 1 [file cancers-16-03077-s001.zip › cancers-3154863-supplementary/Supplementary File 2/036A.jpg]

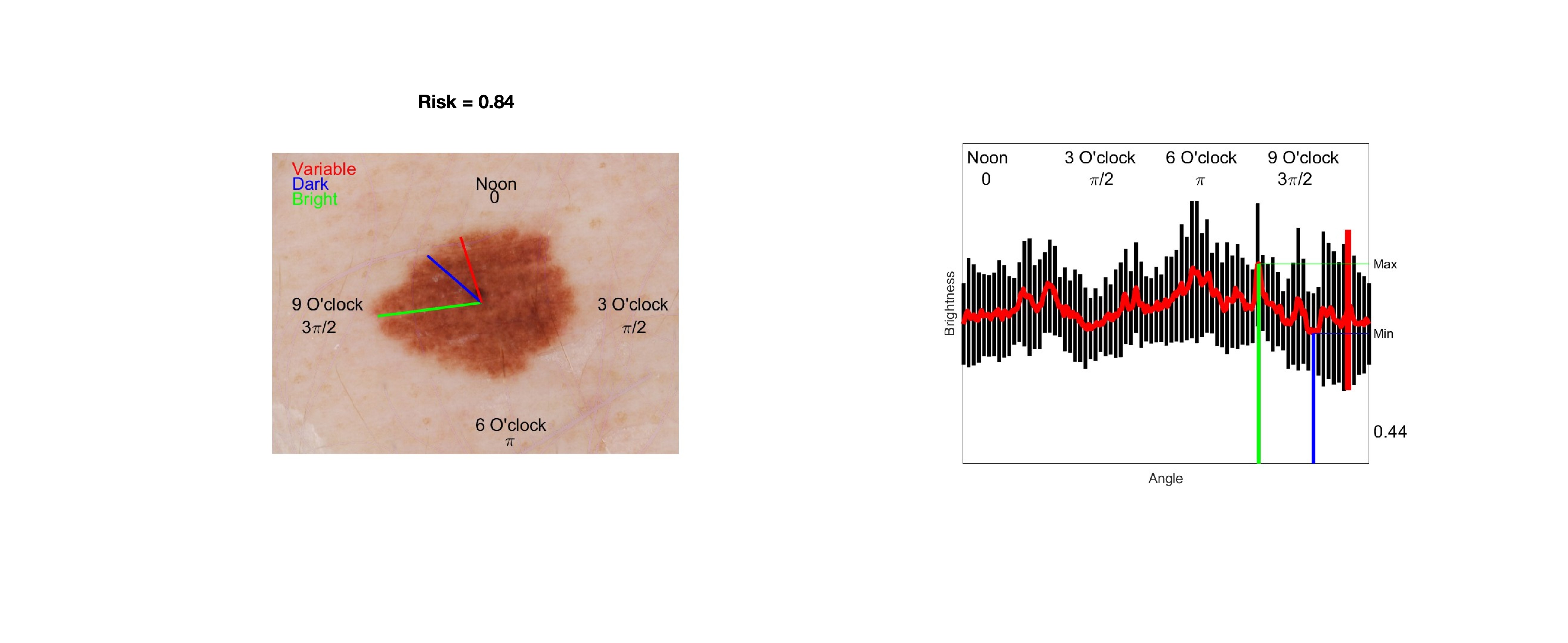

Supplement: Supplementary file 1 [file cancers-16-03077-s001.zip › cancers-3154863-supplementary/Supplementary File 2/036B.jpg]

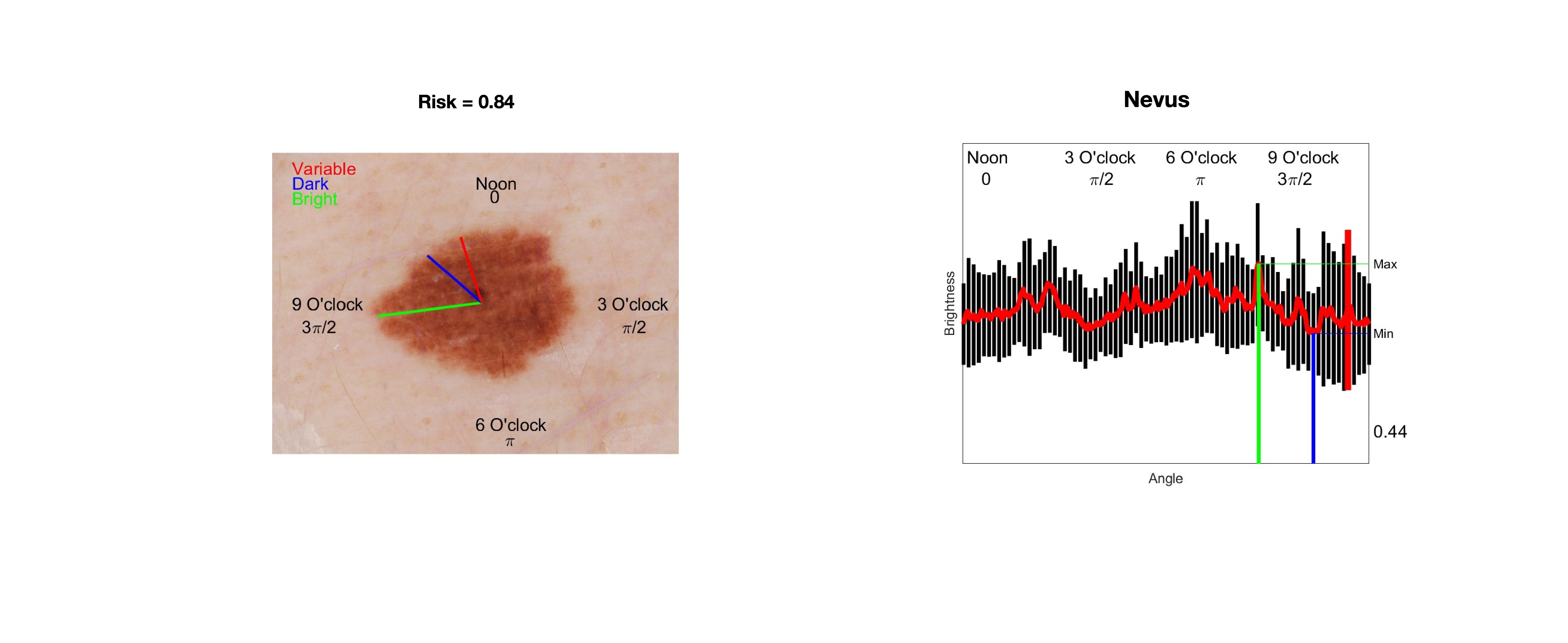

Supplement: Supplementary file 1 [file cancers-16-03077-s001.zip › cancers-3154863-supplementary/Supplementary File 2/036C.jpg]

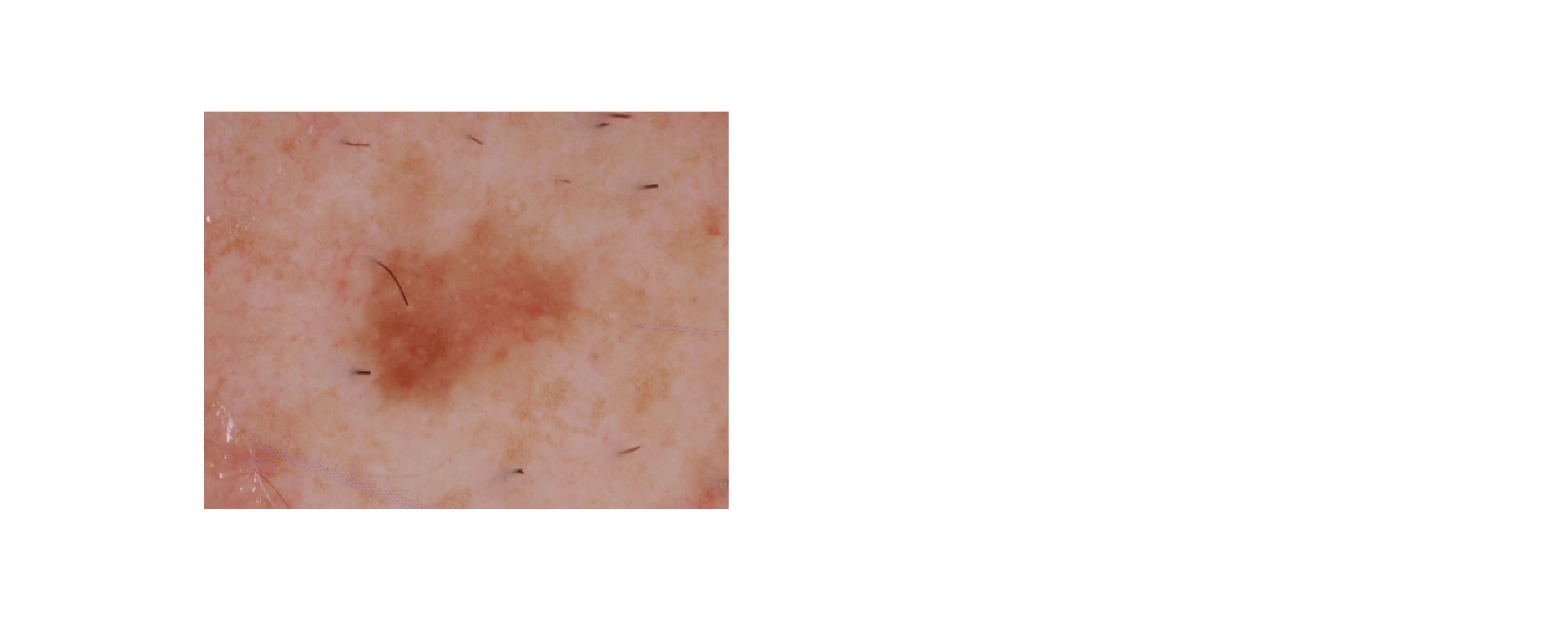

Supplement: Supplementary file 1 [file cancers-16-03077-s001.zip › cancers-3154863-supplementary/Supplementary File 2/037A.jpg]

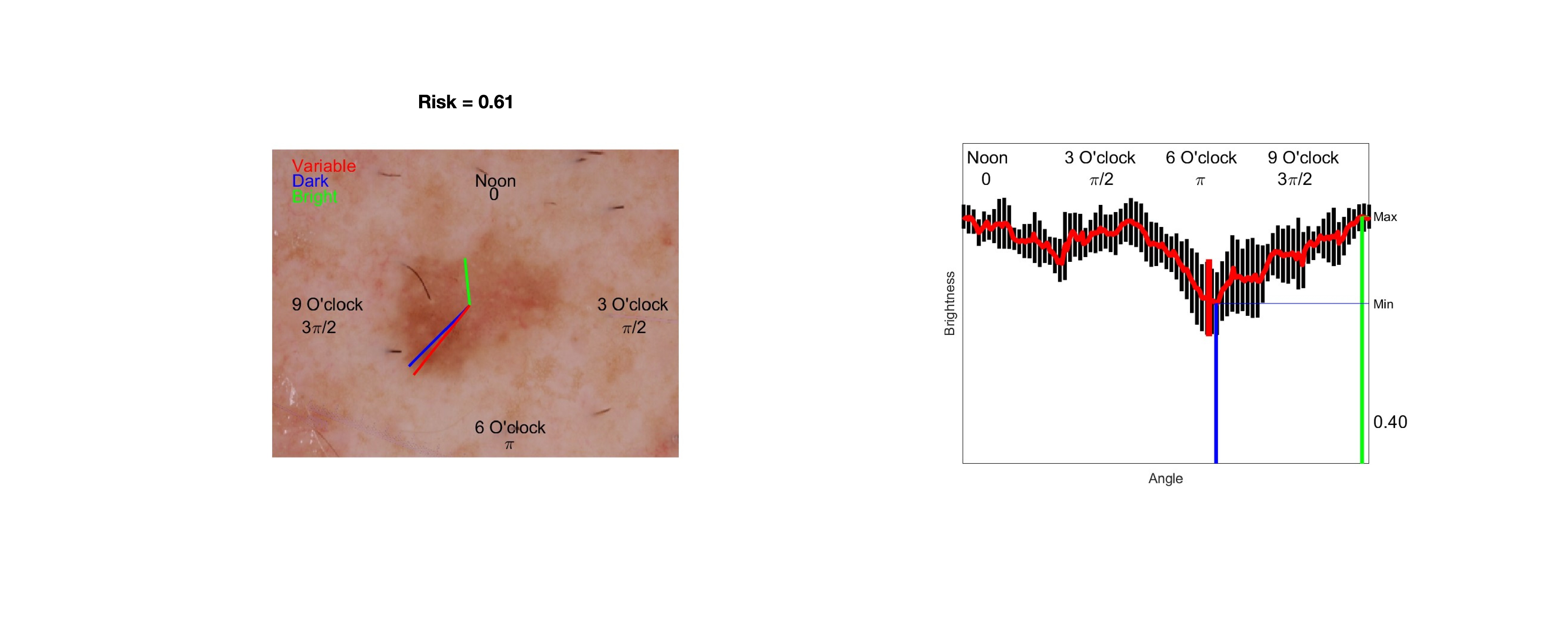

Supplement: Supplementary file 1 [file cancers-16-03077-s001.zip › cancers-3154863-supplementary/Supplementary File 2/037B.jpg]

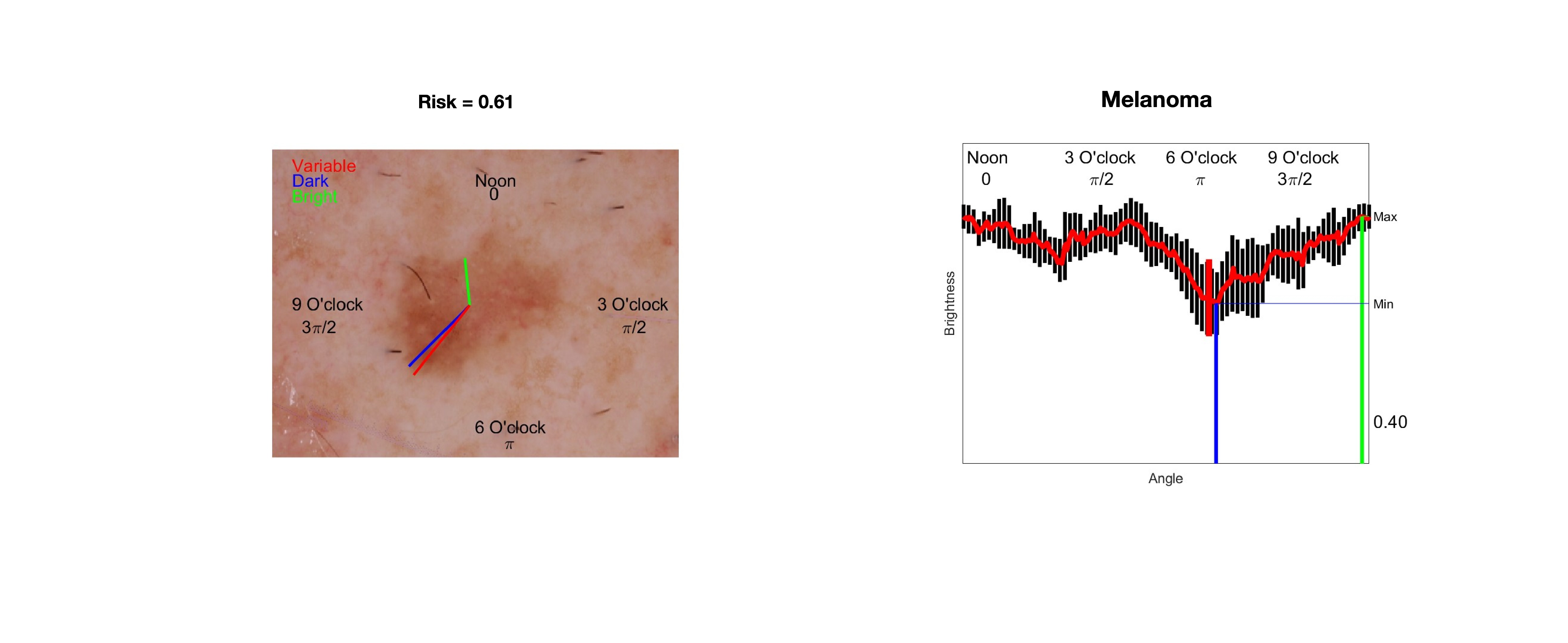

Supplement: Supplementary file 1 [file cancers-16-03077-s001.zip › cancers-3154863-supplementary/Supplementary File 2/037C.jpg]

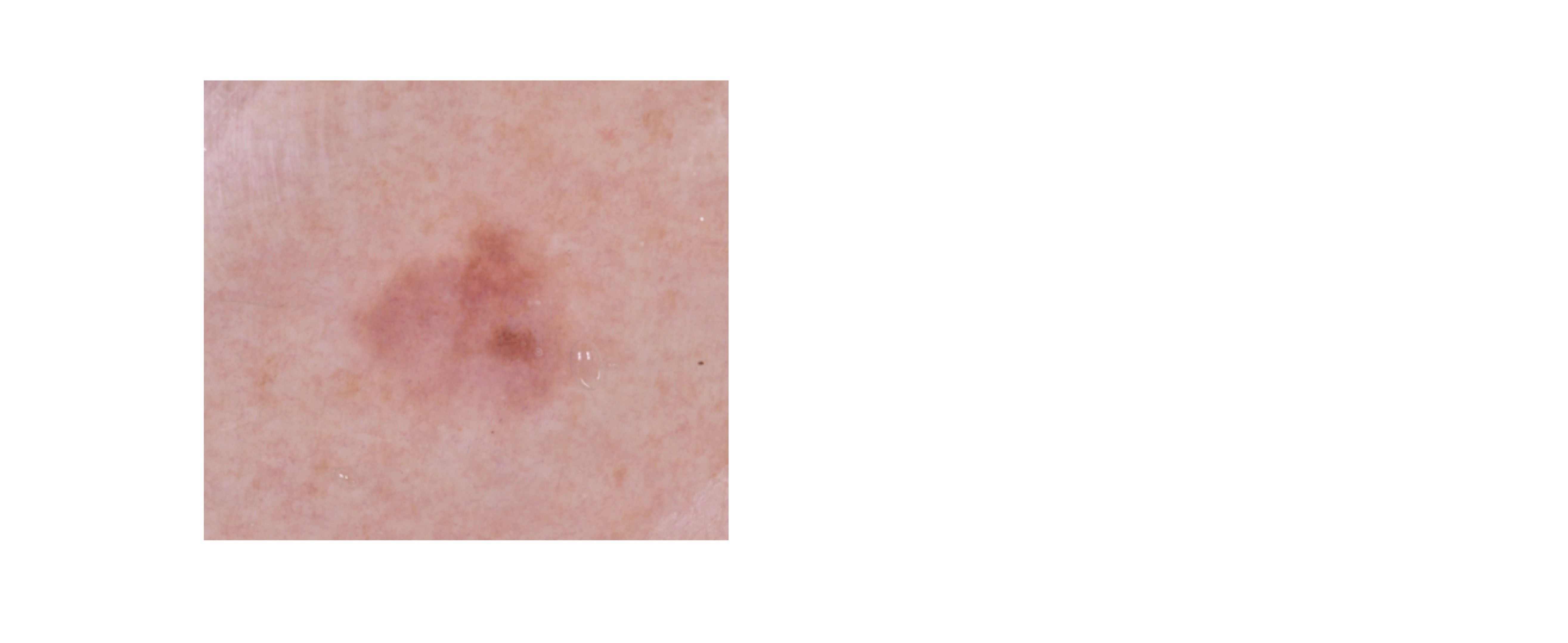

Supplement: Supplementary file 1 [file cancers-16-03077-s001.zip › cancers-3154863-supplementary/Supplementary File 2/038A.jpg]

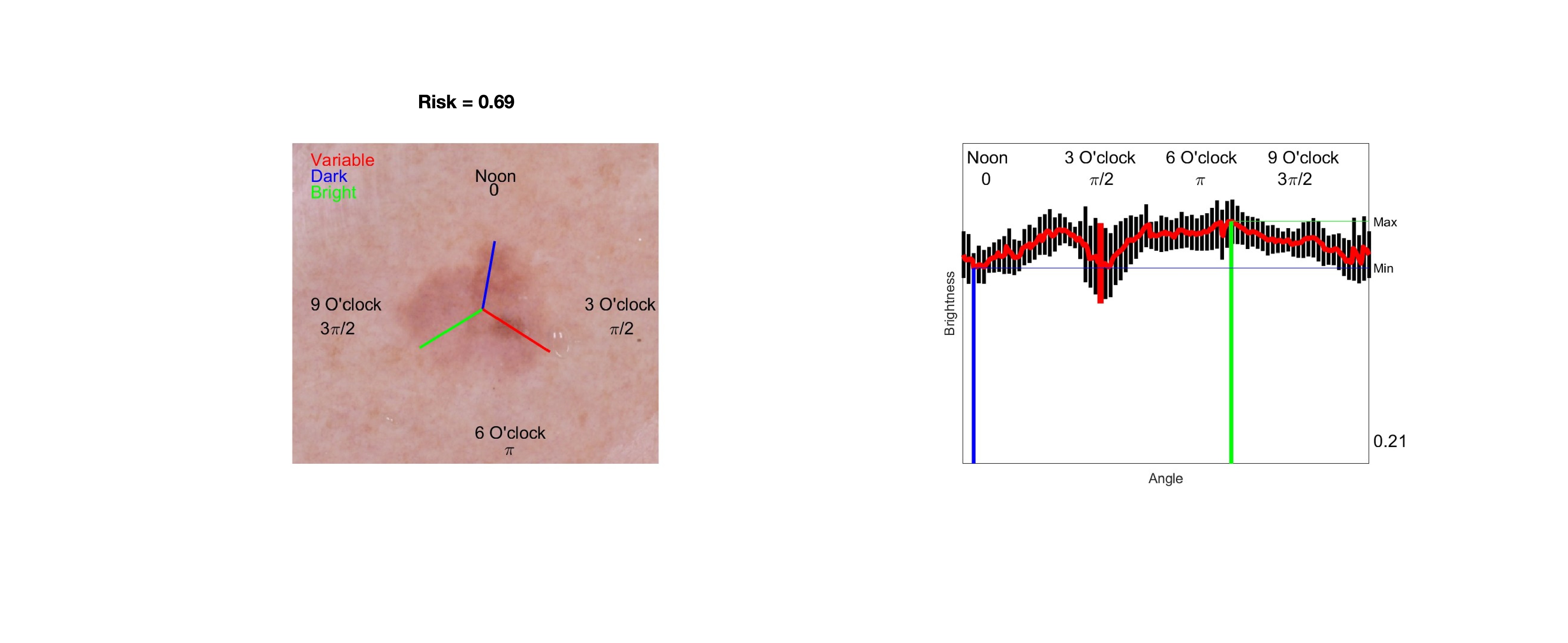

Supplement: Supplementary file 1 [file cancers-16-03077-s001.zip › cancers-3154863-supplementary/Supplementary File 2/038B.jpg]

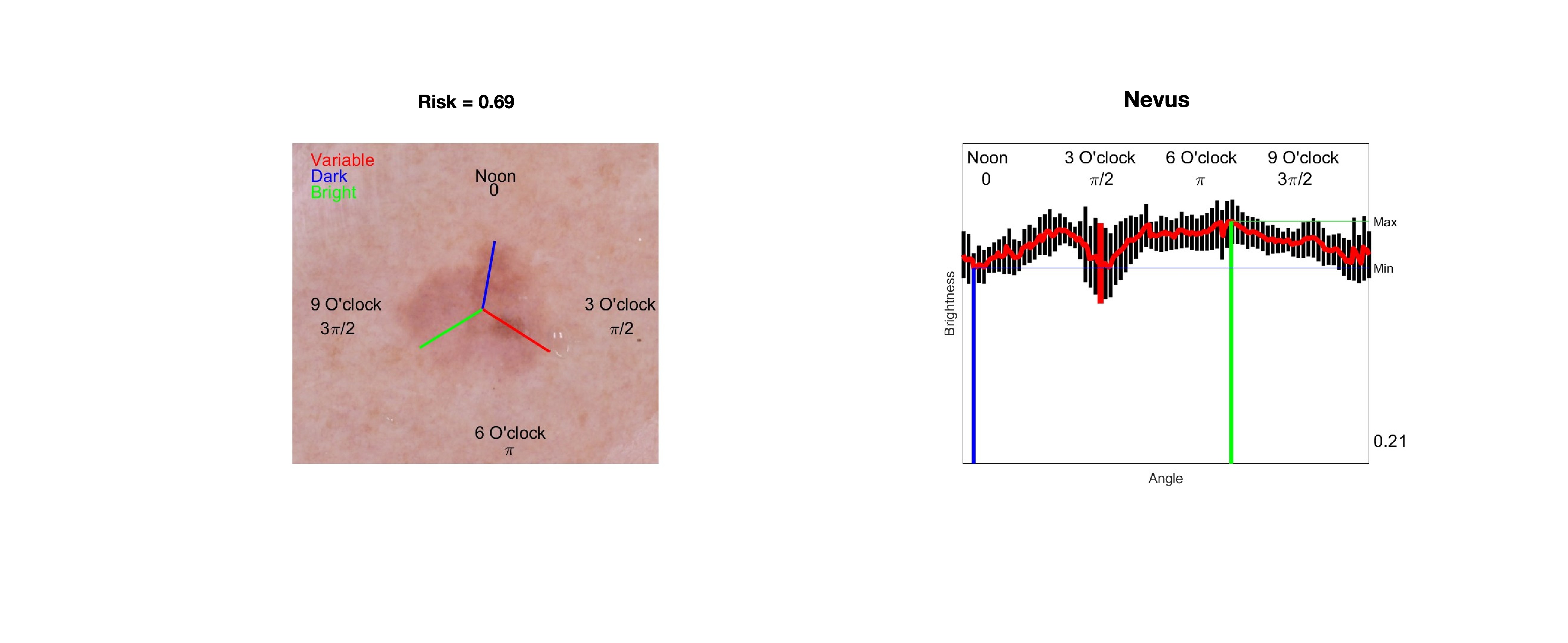

Supplement: Supplementary file 1 [file cancers-16-03077-s001.zip › cancers-3154863-supplementary/Supplementary File 2/038C.jpg]

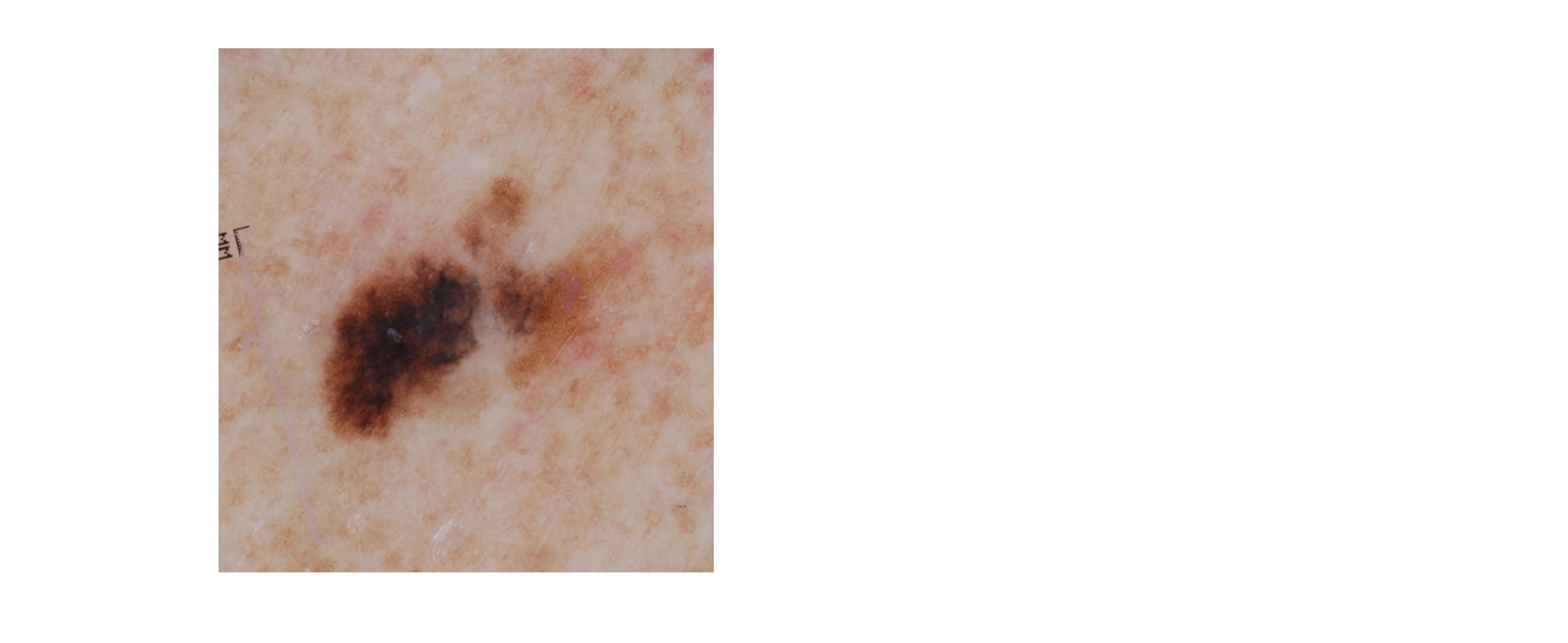

Supplement: Supplementary file 1 [file cancers-16-03077-s001.zip › cancers-3154863-supplementary/Supplementary File 2/039A.jpg]

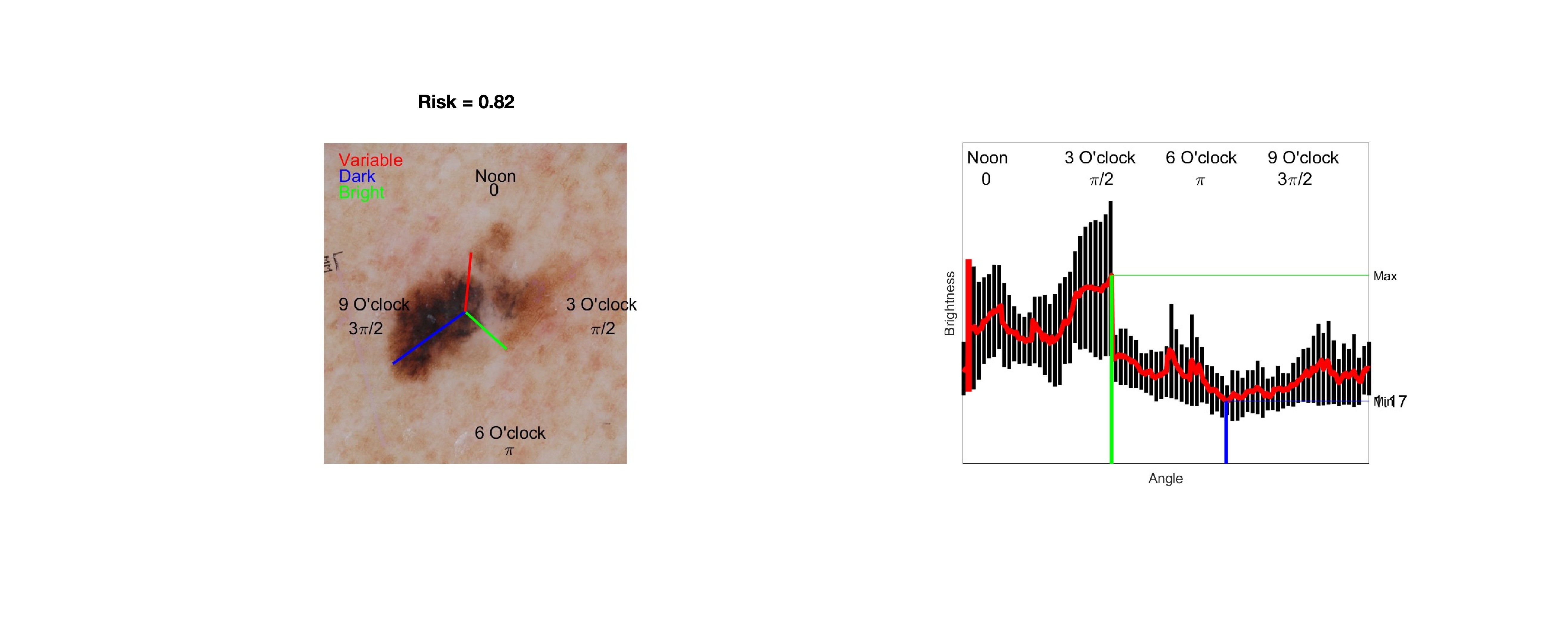

Supplement: Supplementary file 1 [file cancers-16-03077-s001.zip › cancers-3154863-supplementary/Supplementary File 2/039B.jpg]

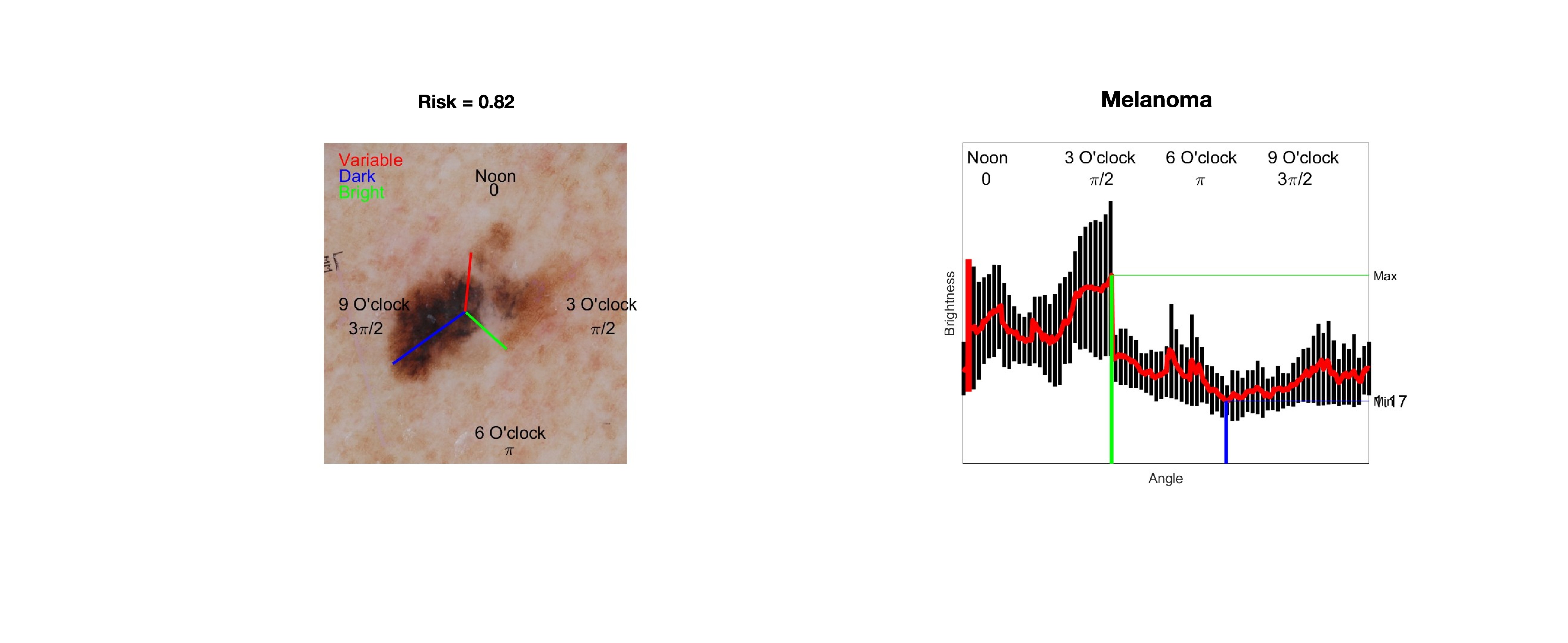

Supplement: Supplementary file 1 [file cancers-16-03077-s001.zip › cancers-3154863-supplementary/Supplementary File 2/039C.jpg]

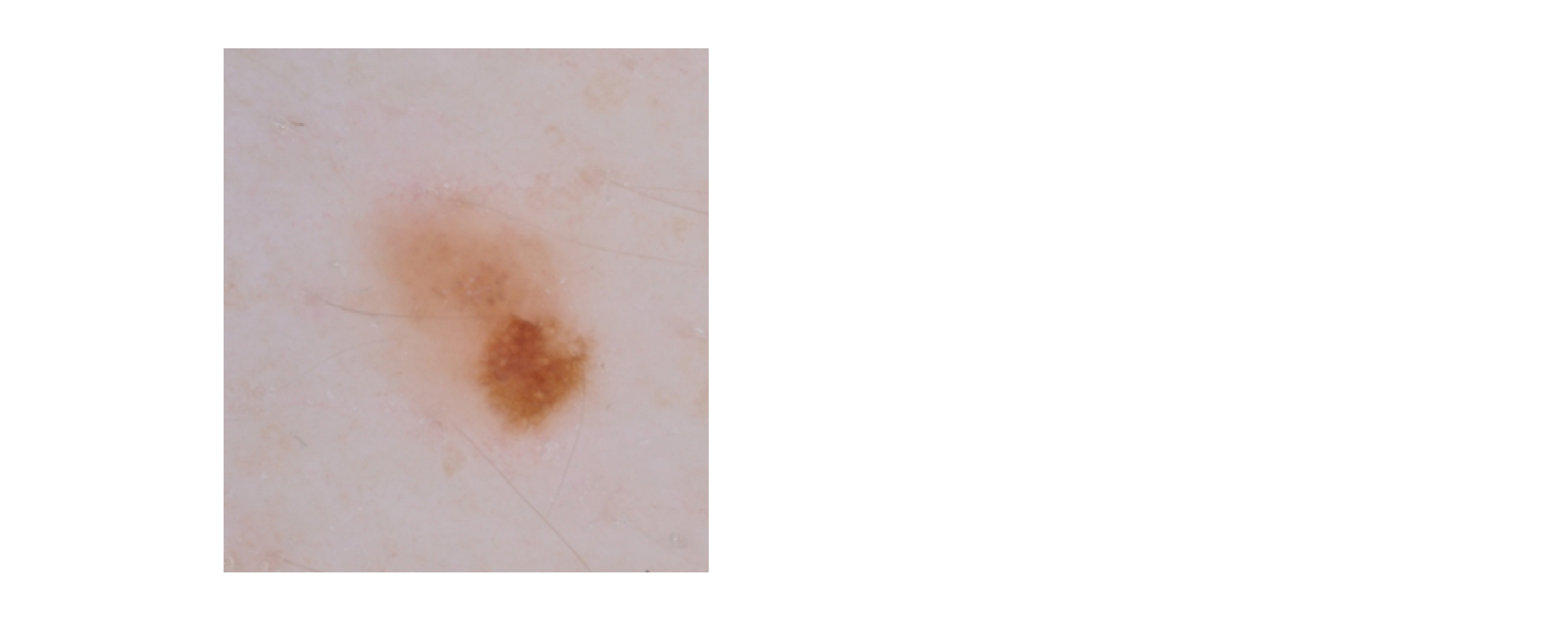

Supplement: Supplementary file 1 [file cancers-16-03077-s001.zip › cancers-3154863-supplementary/Supplementary File 2/040A.jpg]

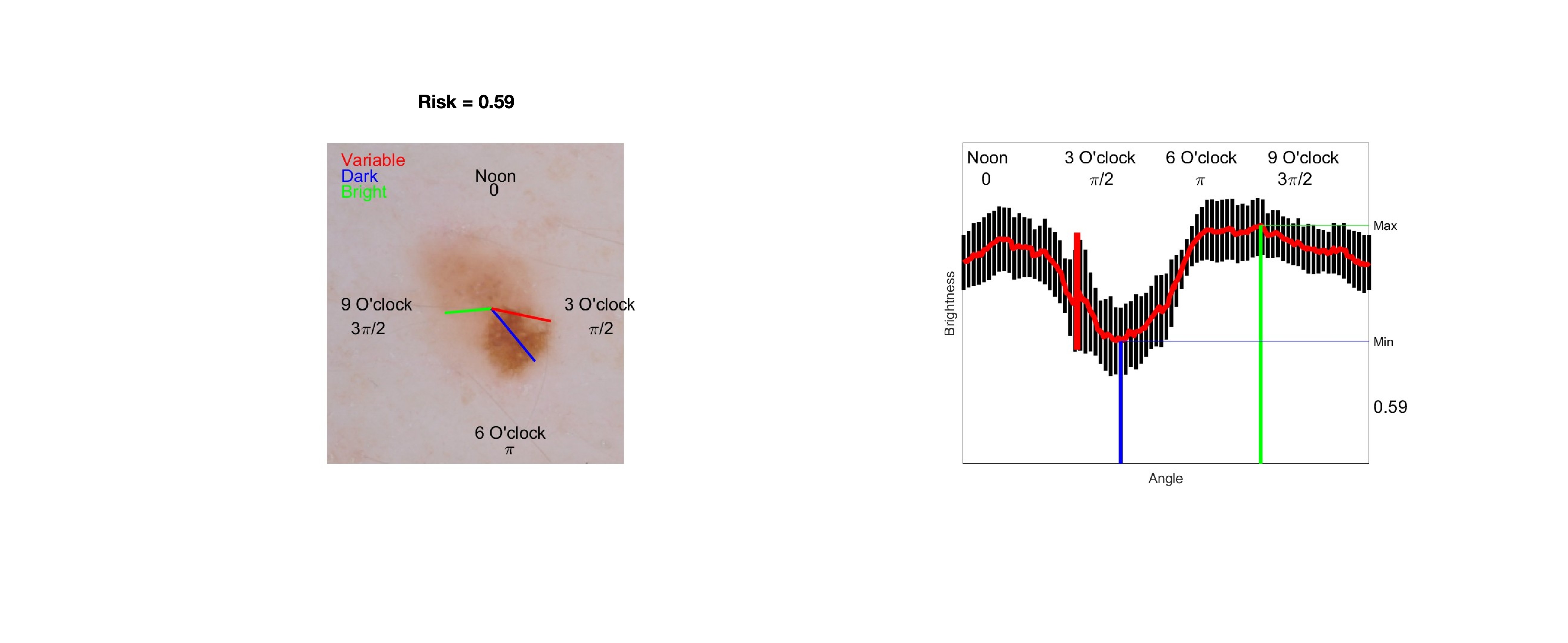

Supplement: Supplementary file 1 [file cancers-16-03077-s001.zip › cancers-3154863-supplementary/Supplementary File 2/040B.jpg]

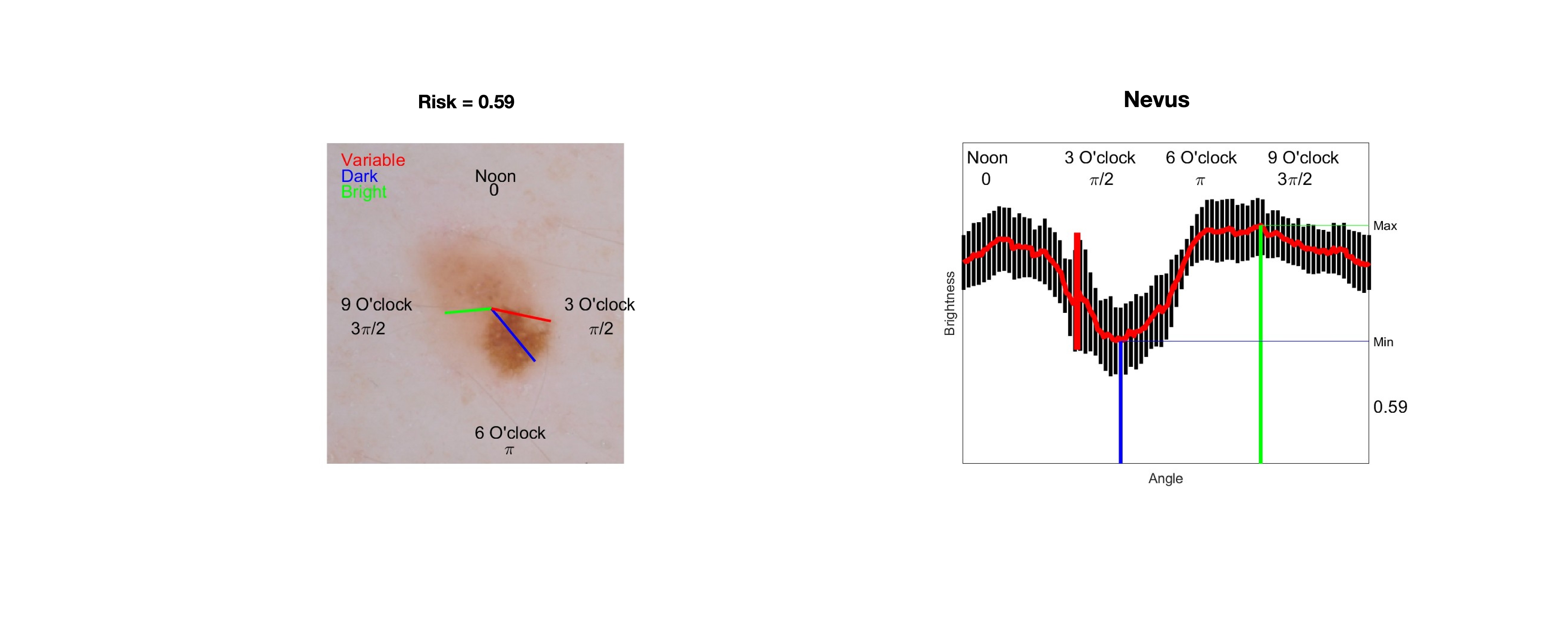

Supplement: Supplementary file 1 [file cancers-16-03077-s001.zip › cancers-3154863-supplementary/Supplementary File 2/040C.jpg]

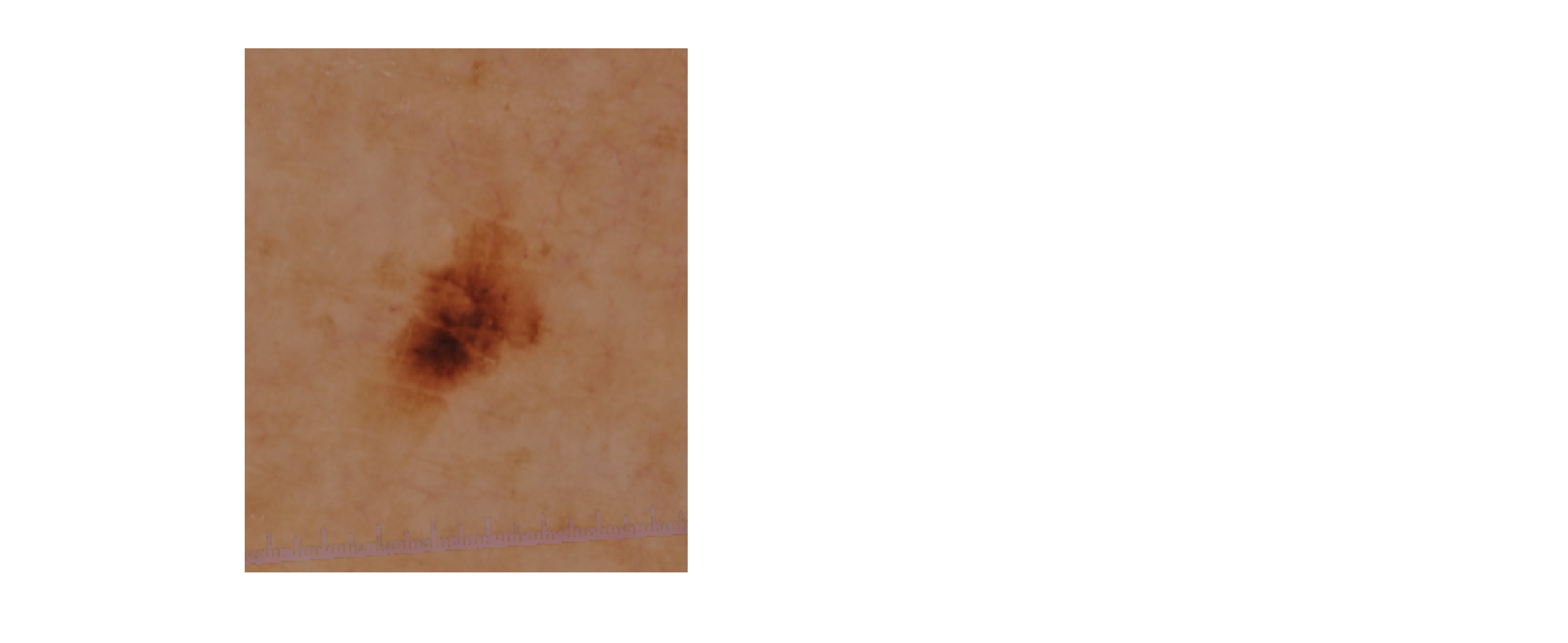

Supplement: Supplementary file 1 [file cancers-16-03077-s001.zip › cancers-3154863-supplementary/Supplementary File 2/041A.jpg]

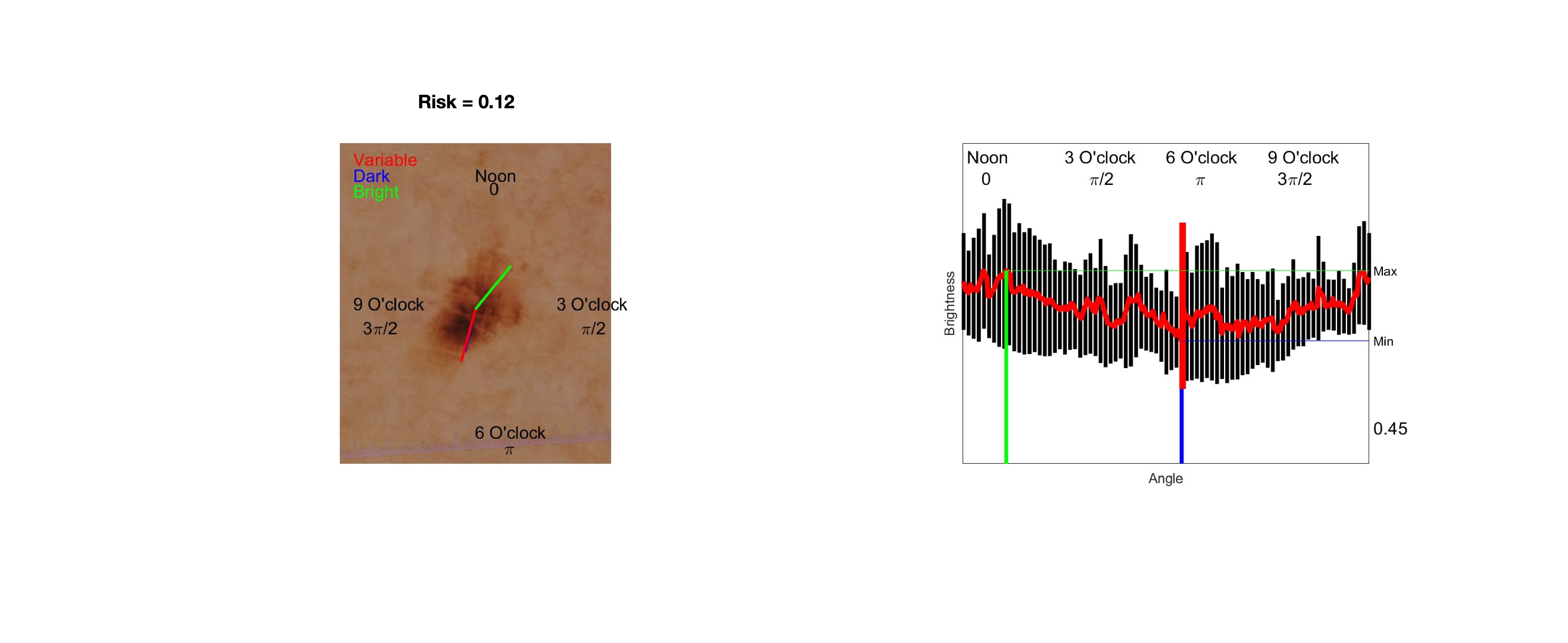

Supplement: Supplementary file 1 [file cancers-16-03077-s001.zip › cancers-3154863-supplementary/Supplementary File 2/041B.jpg]

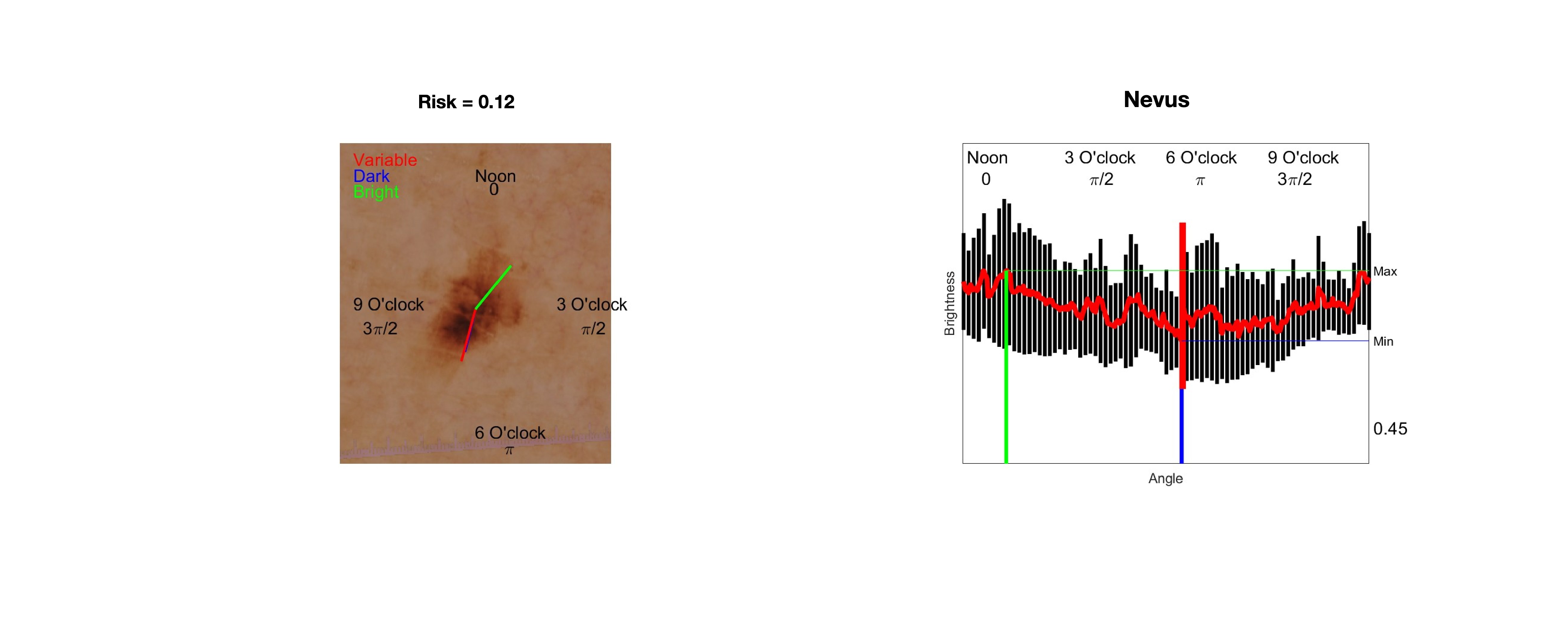

Supplement: Supplementary file 1 [file cancers-16-03077-s001.zip › cancers-3154863-supplementary/Supplementary File 2/041C.jpg]

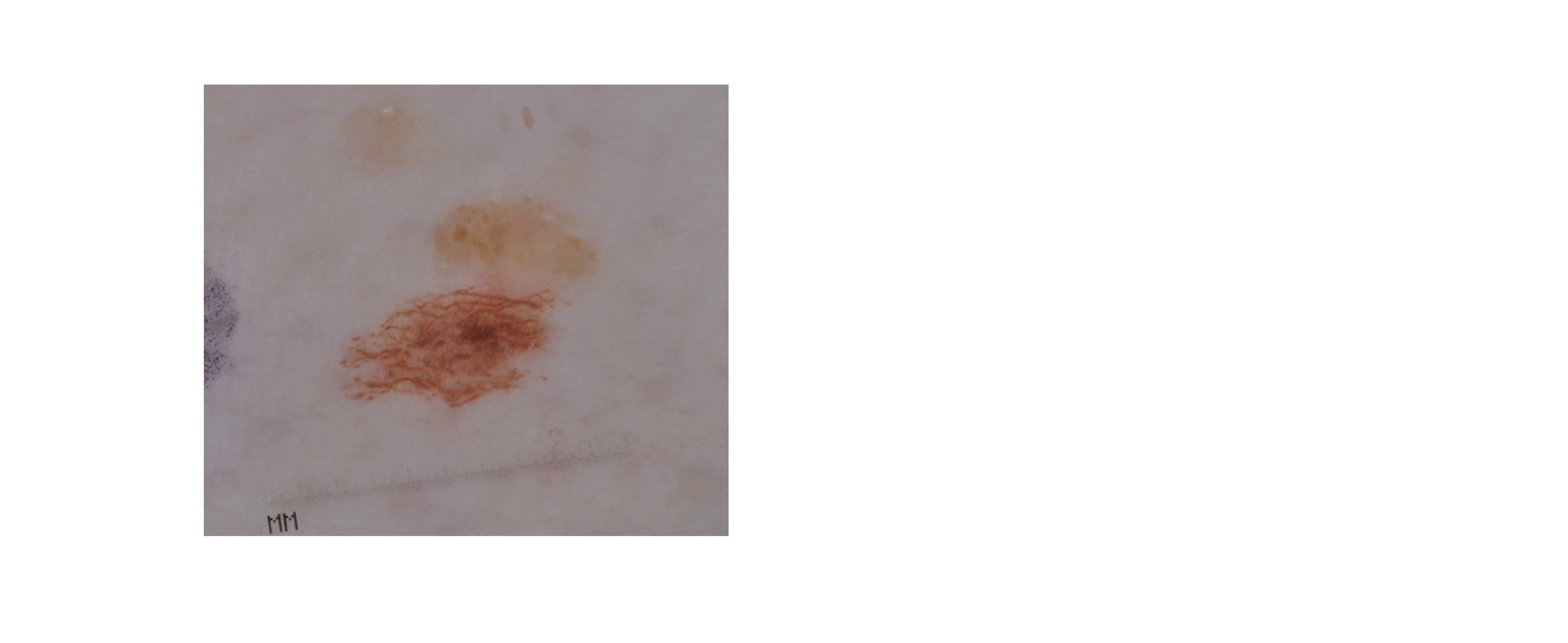

Supplement: Supplementary file 1 [file cancers-16-03077-s001.zip › cancers-3154863-supplementary/Supplementary File 2/042A.jpg]

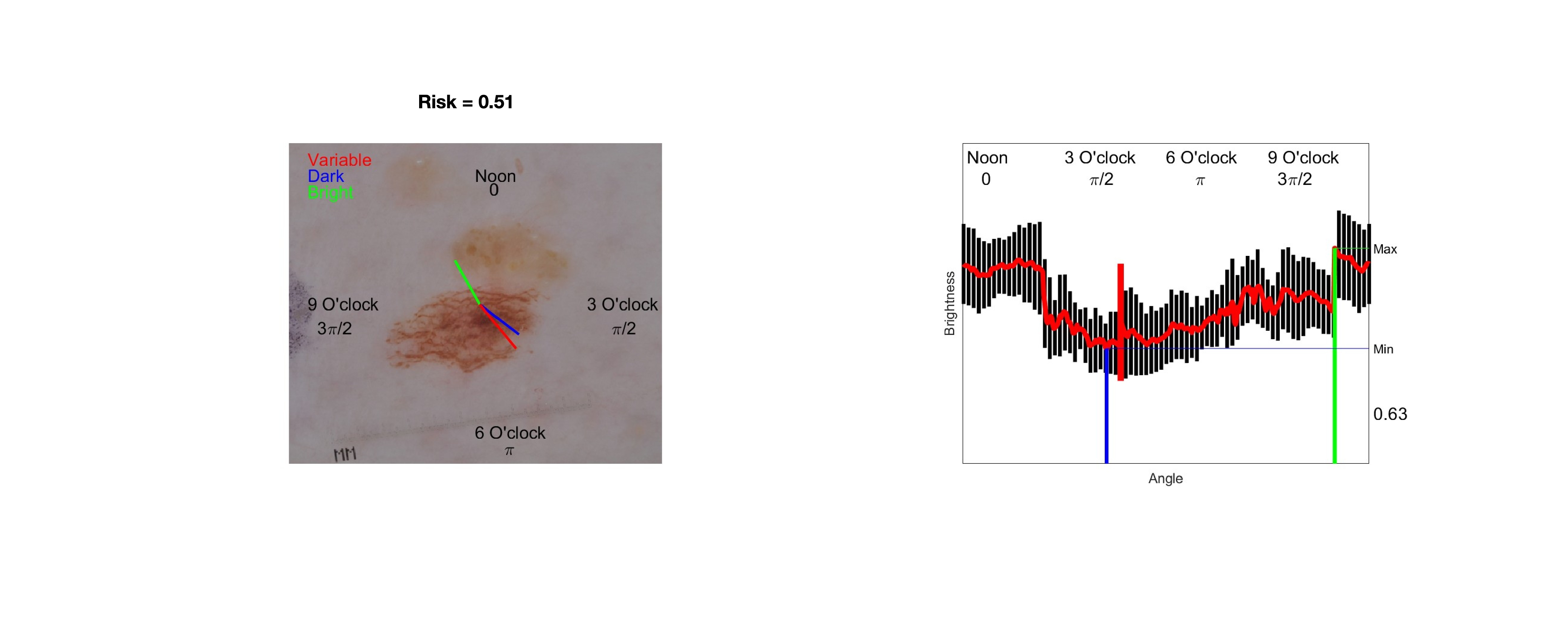

Supplement: Supplementary file 1 [file cancers-16-03077-s001.zip › cancers-3154863-supplementary/Supplementary File 2/042B.jpg]

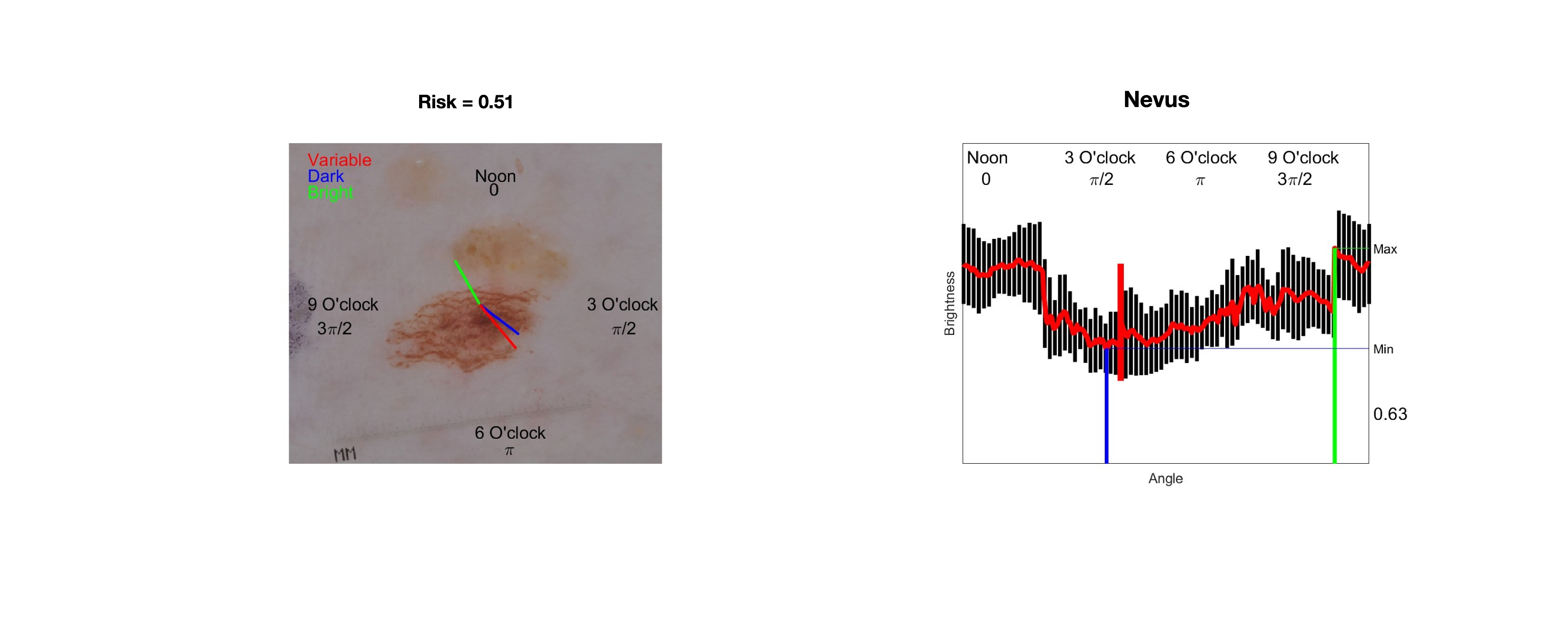

Supplement: Supplementary file 1 [file cancers-16-03077-s001.zip › cancers-3154863-supplementary/Supplementary File 2/042C.jpg]

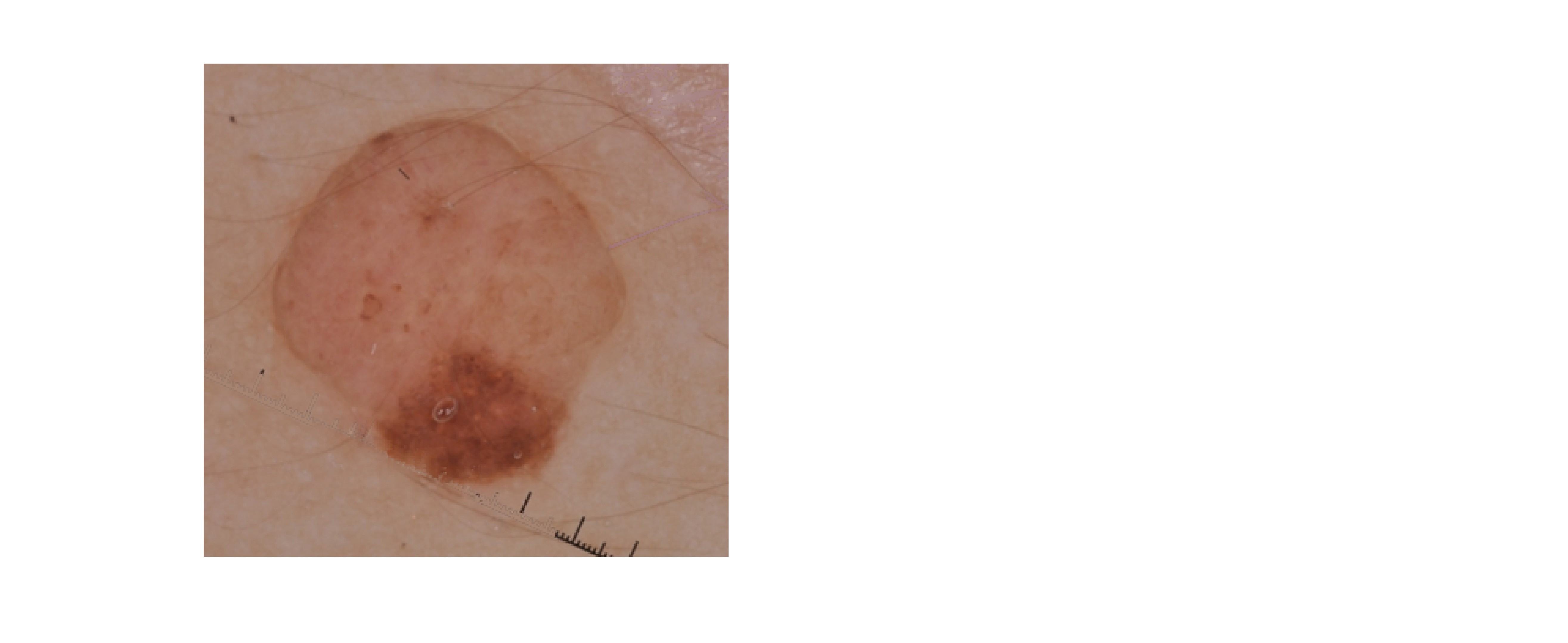

Supplement: Supplementary file 1 [file cancers-16-03077-s001.zip › cancers-3154863-supplementary/Supplementary File 2/043A.jpg]

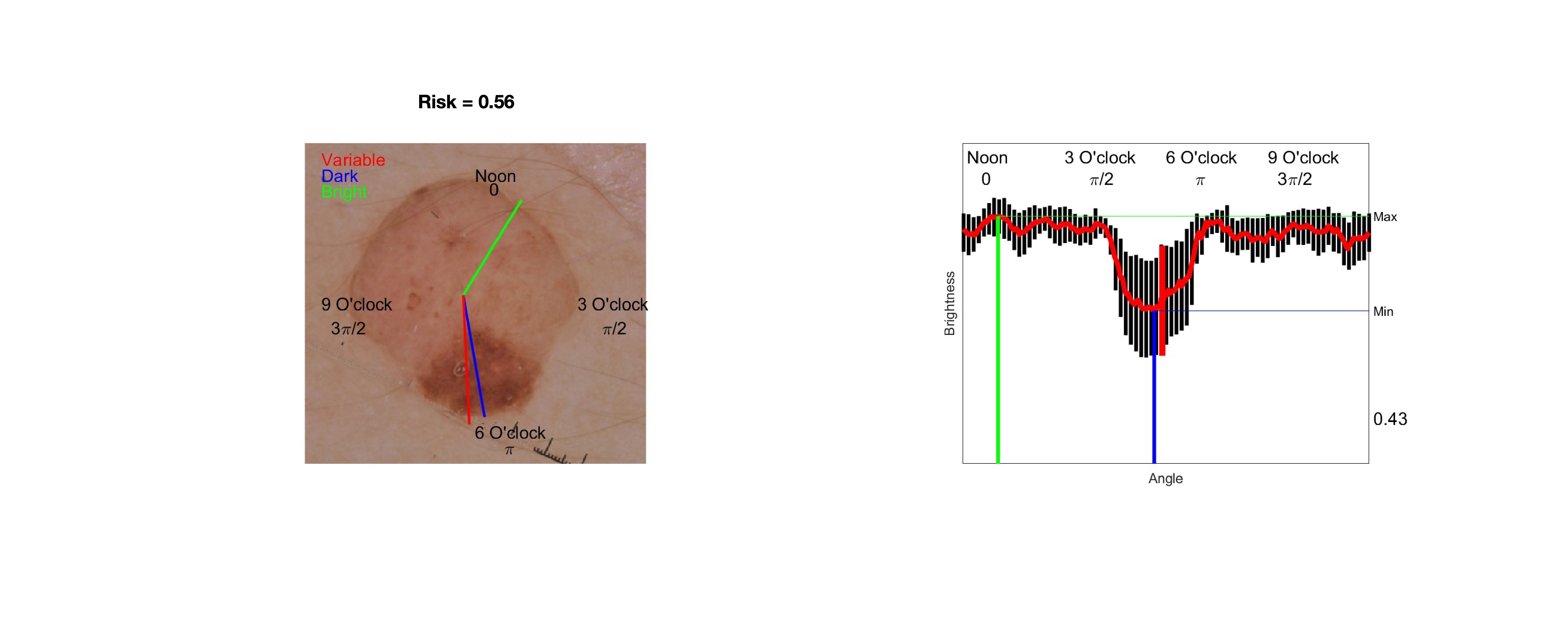

Supplement: Supplementary file 1 [file cancers-16-03077-s001.zip › cancers-3154863-supplementary/Supplementary File 2/043B.jpg]

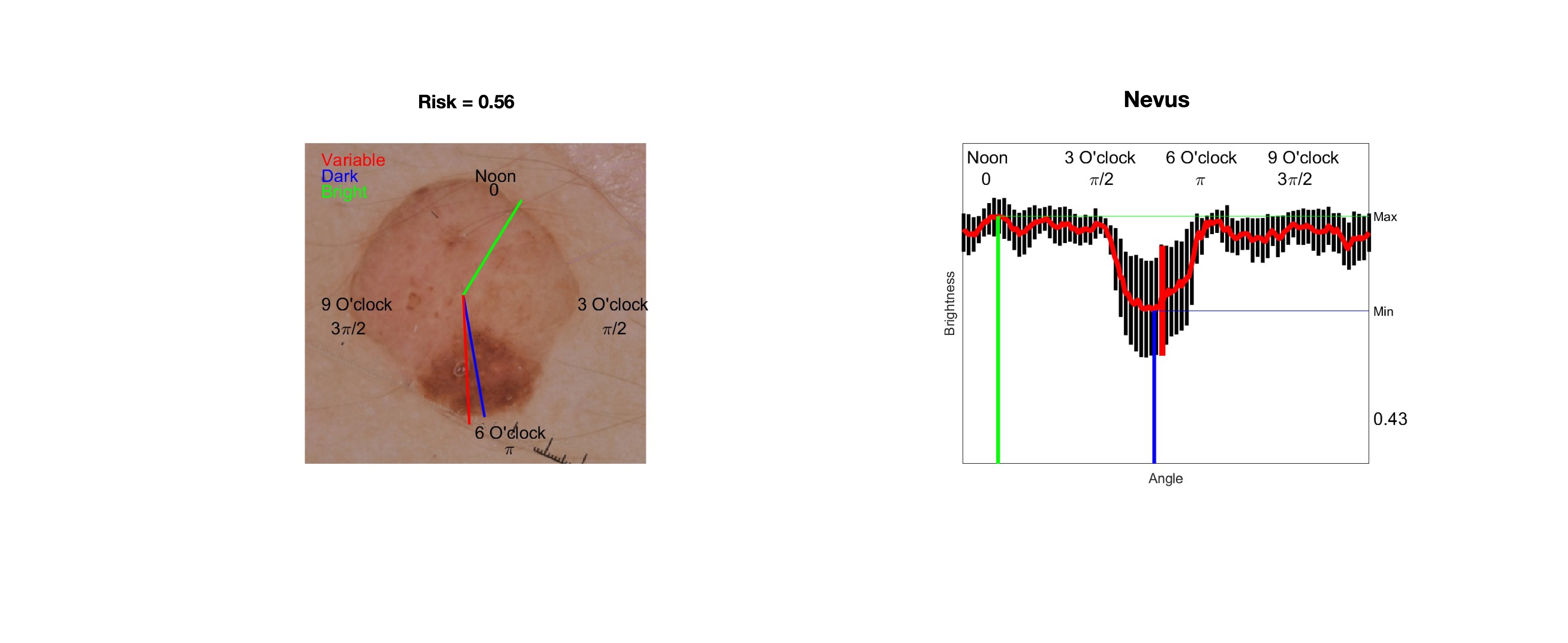

Supplement: Supplementary file 1 [file cancers-16-03077-s001.zip › cancers-3154863-supplementary/Supplementary File 2/043C.jpg]

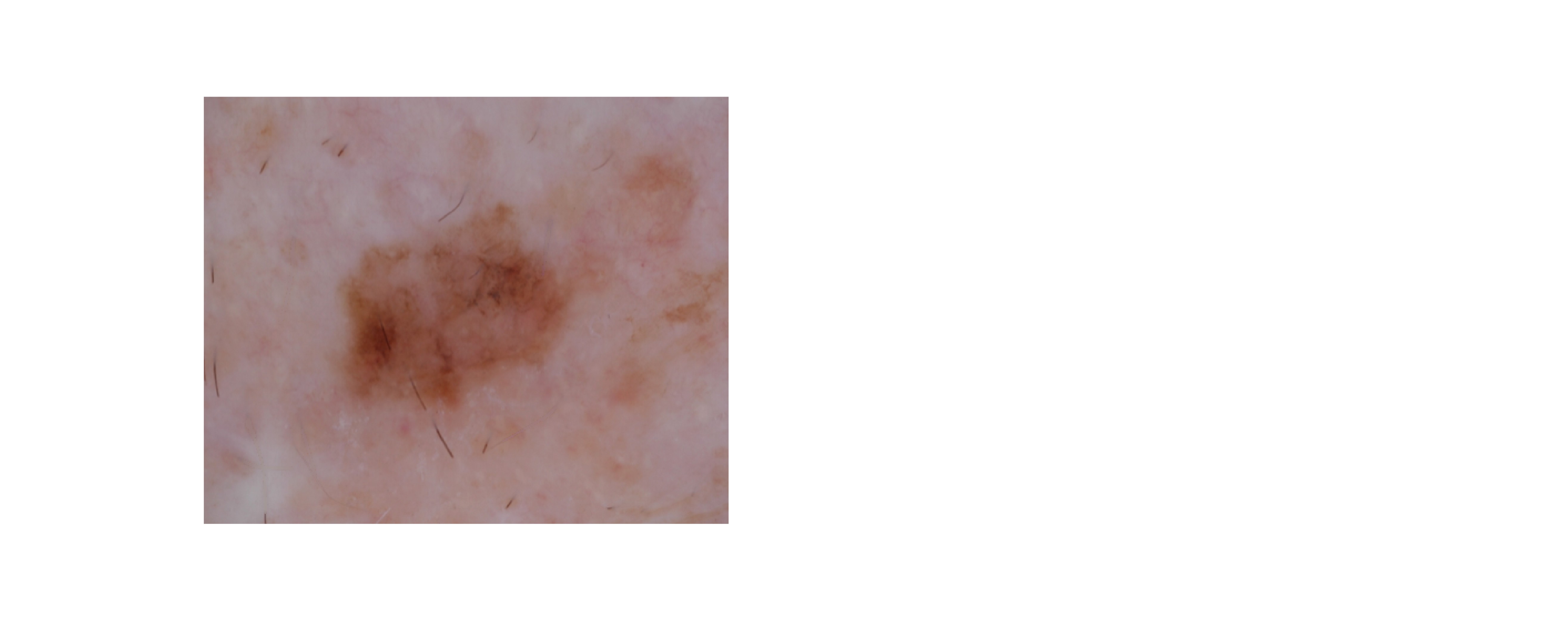

Supplement: Supplementary file 1 [file cancers-16-03077-s001.zip › cancers-3154863-supplementary/Supplementary File 2/044A.jpg]

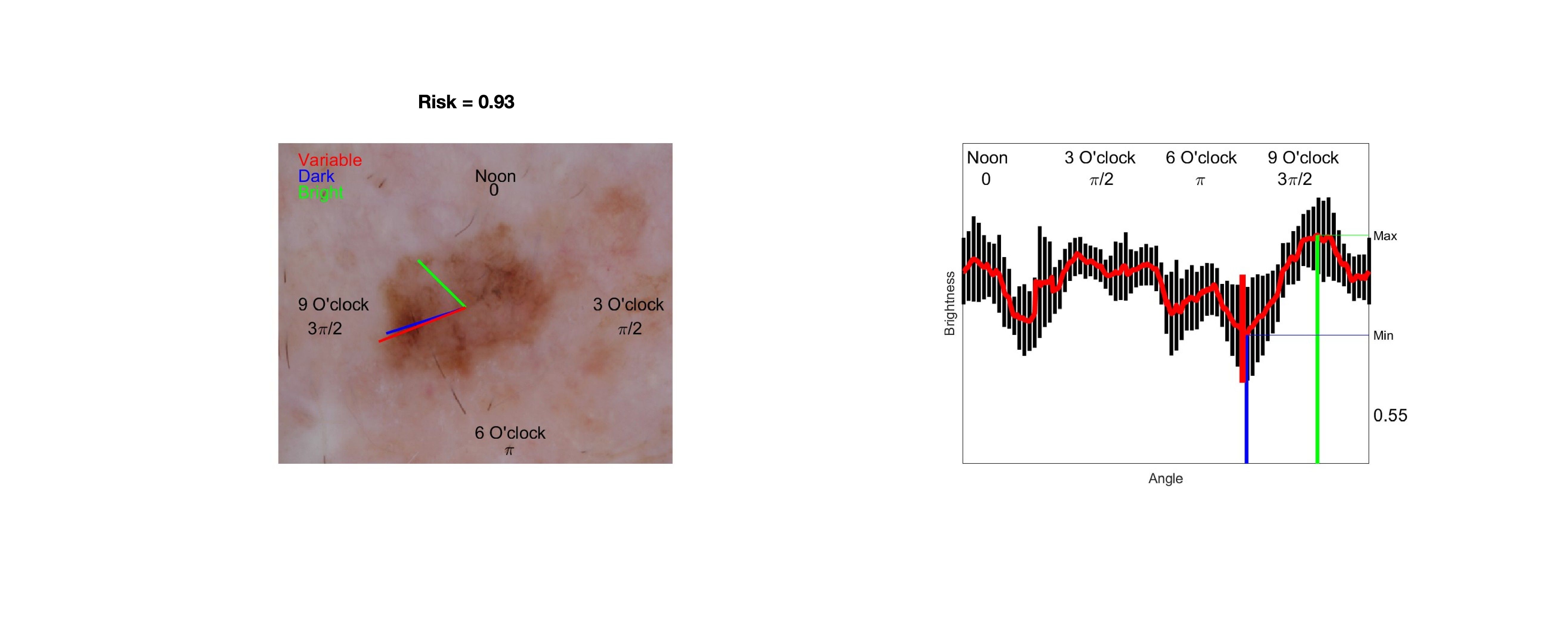

Supplement: Supplementary file 1 [file cancers-16-03077-s001.zip › cancers-3154863-supplementary/Supplementary File 2/044B.jpg]

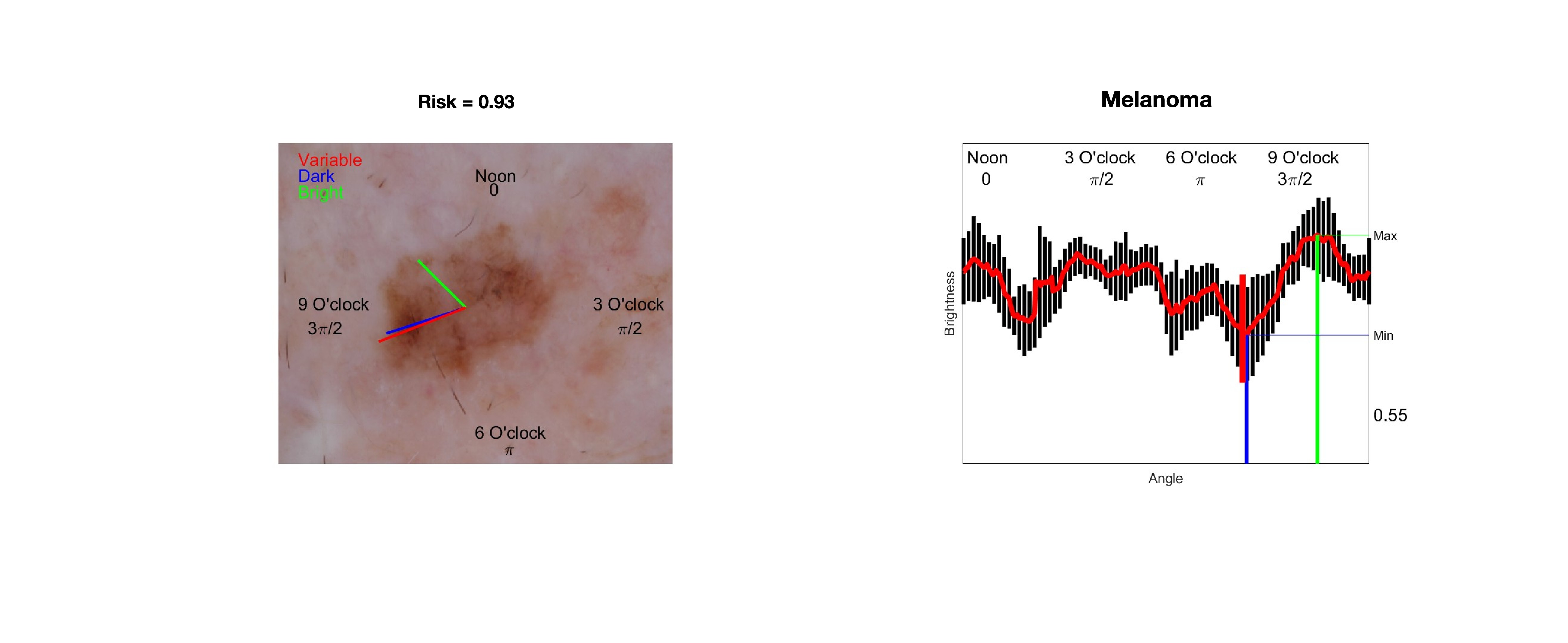

Supplement: Supplementary file 1 [file cancers-16-03077-s001.zip › cancers-3154863-supplementary/Supplementary File 2/044C.jpg]

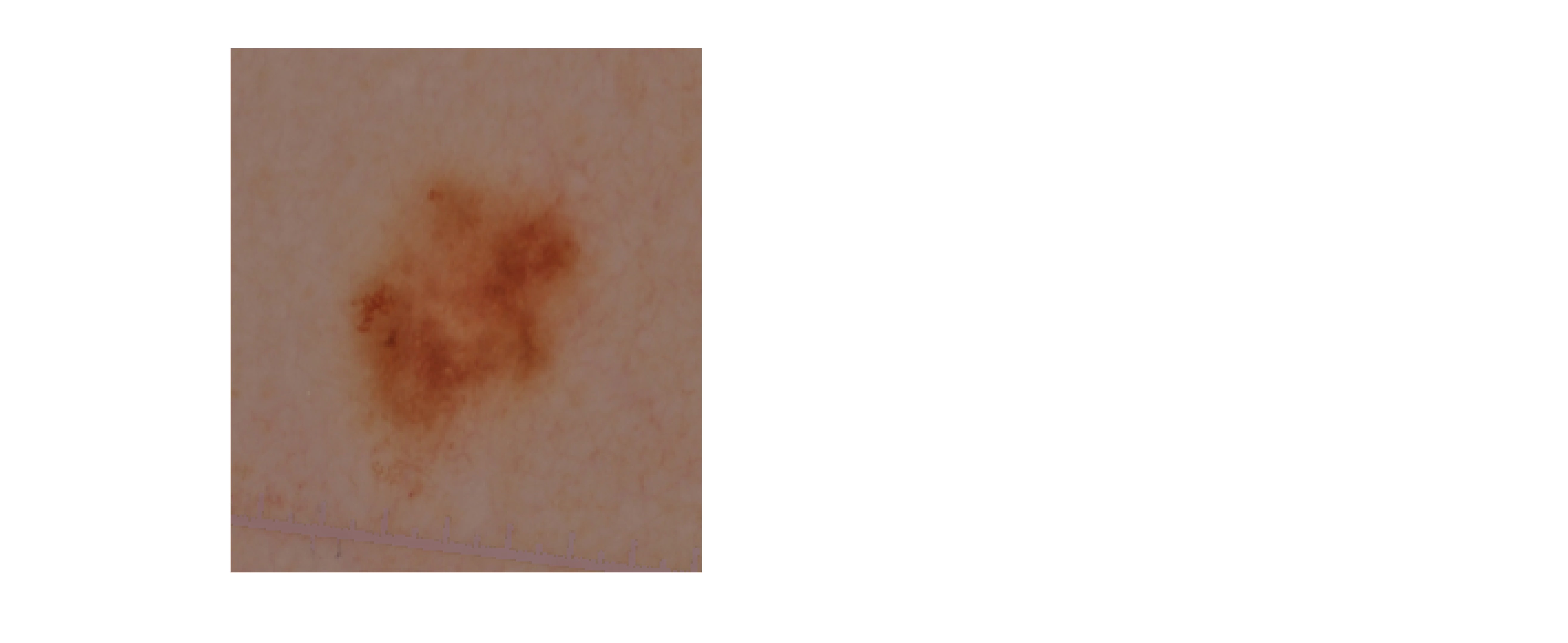

Supplement: Supplementary file 1 [file cancers-16-03077-s001.zip › cancers-3154863-supplementary/Supplementary File 2/045A.jpg]

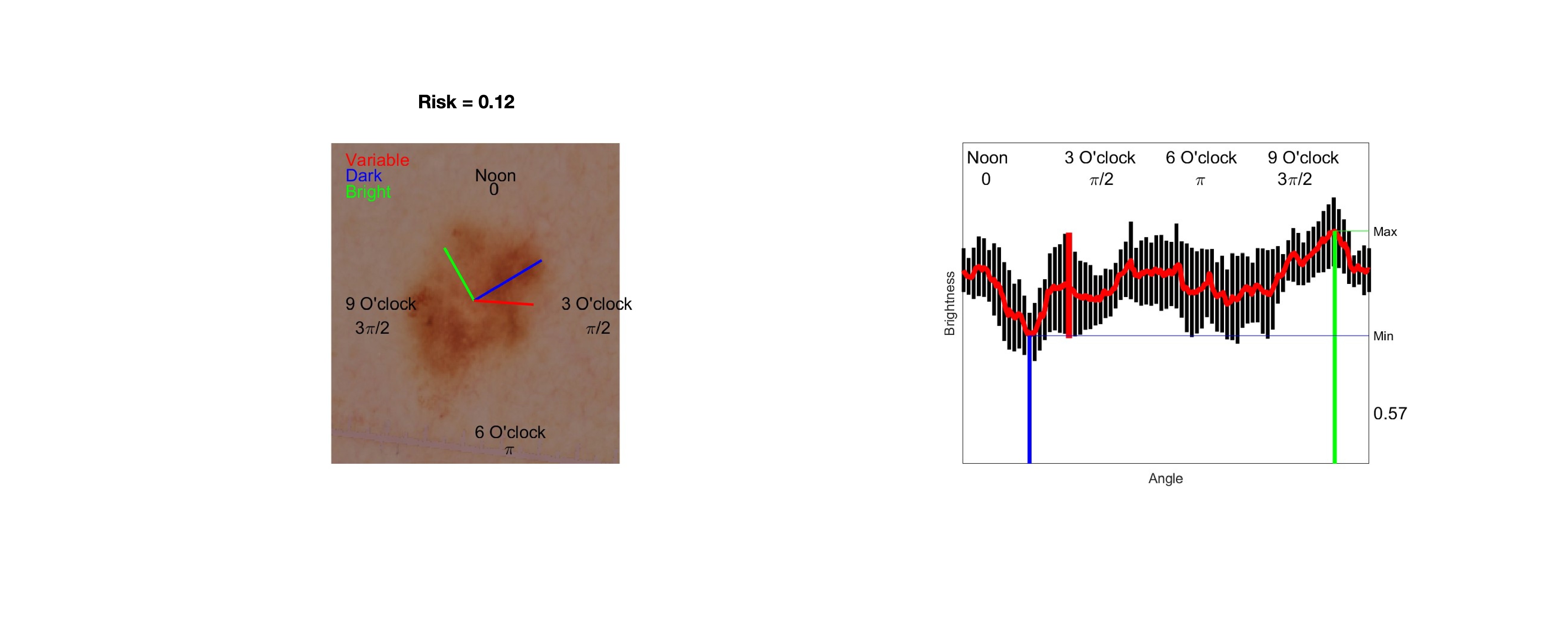

Supplement: Supplementary file 1 [file cancers-16-03077-s001.zip › cancers-3154863-supplementary/Supplementary File 2/045B.jpg]

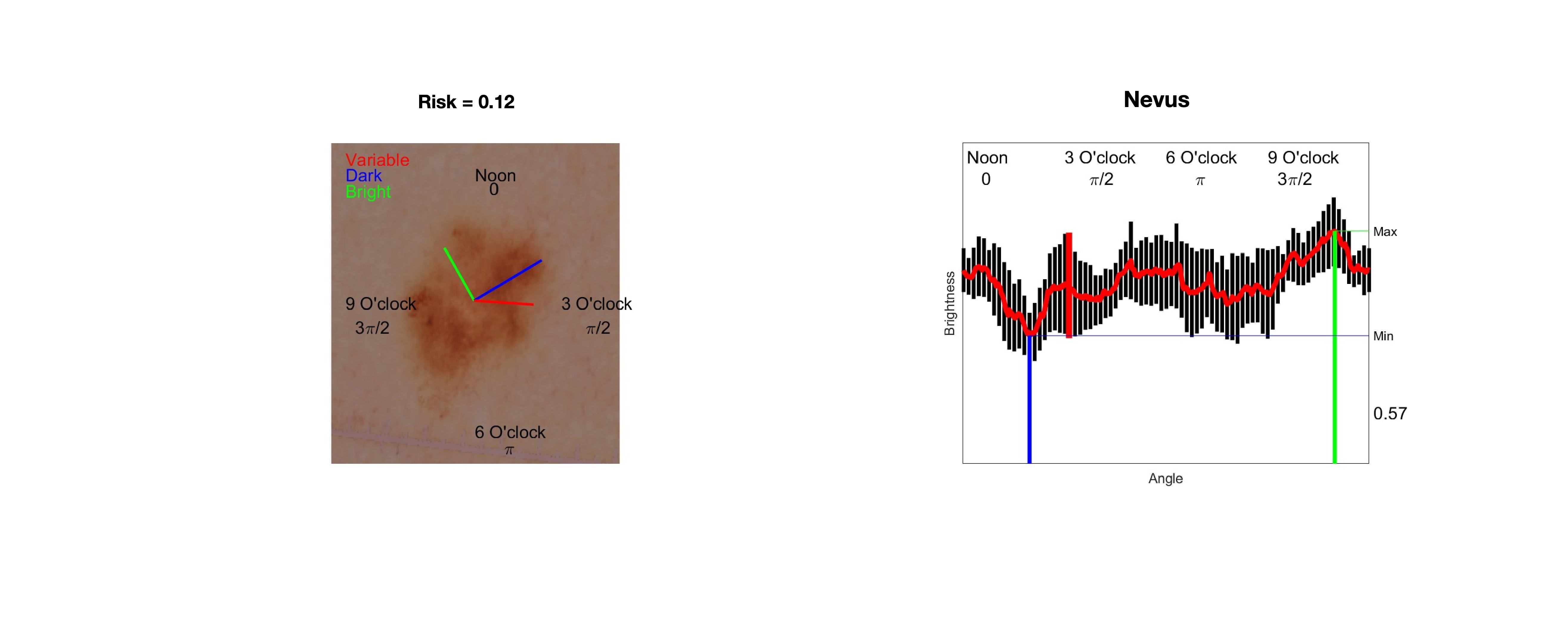

Supplement: Supplementary file 1 [file cancers-16-03077-s001.zip › cancers-3154863-supplementary/Supplementary File 2/045C.jpg]

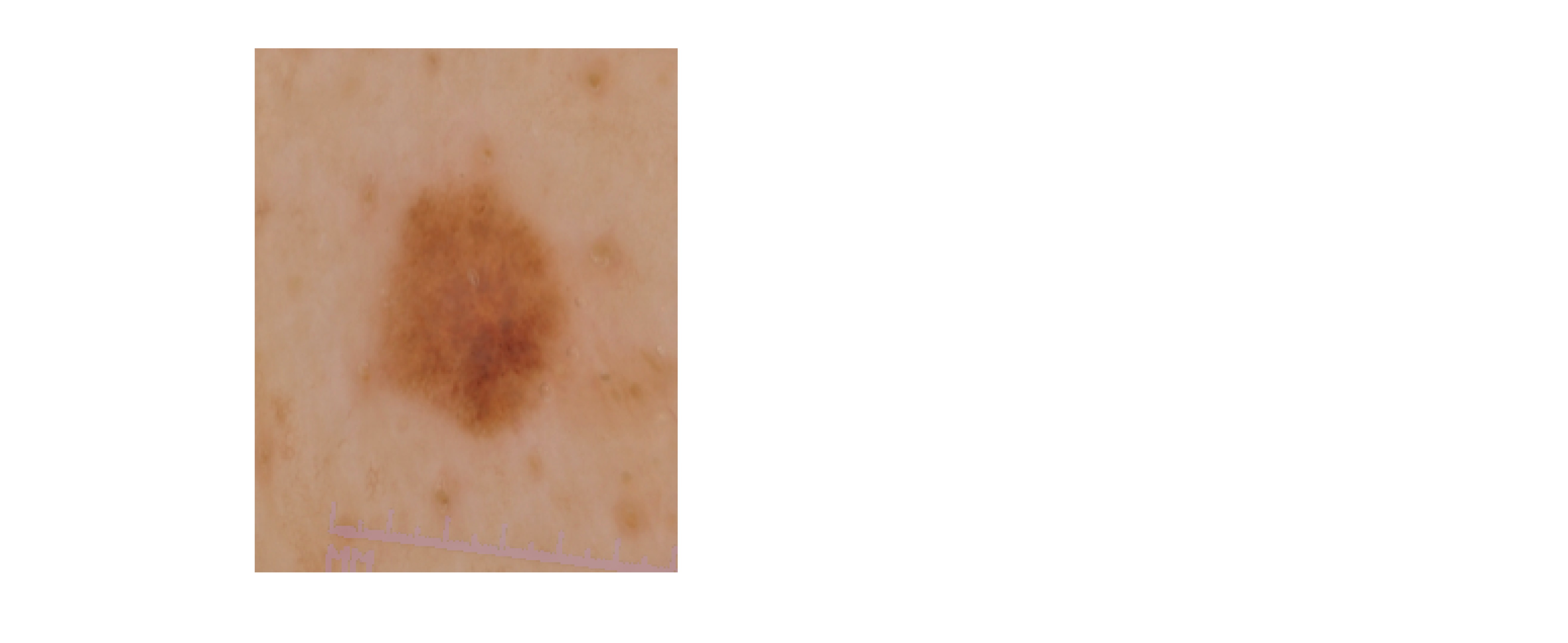

Supplement: Supplementary file 1 [file cancers-16-03077-s001.zip › cancers-3154863-supplementary/Supplementary File 2/046A.jpg]

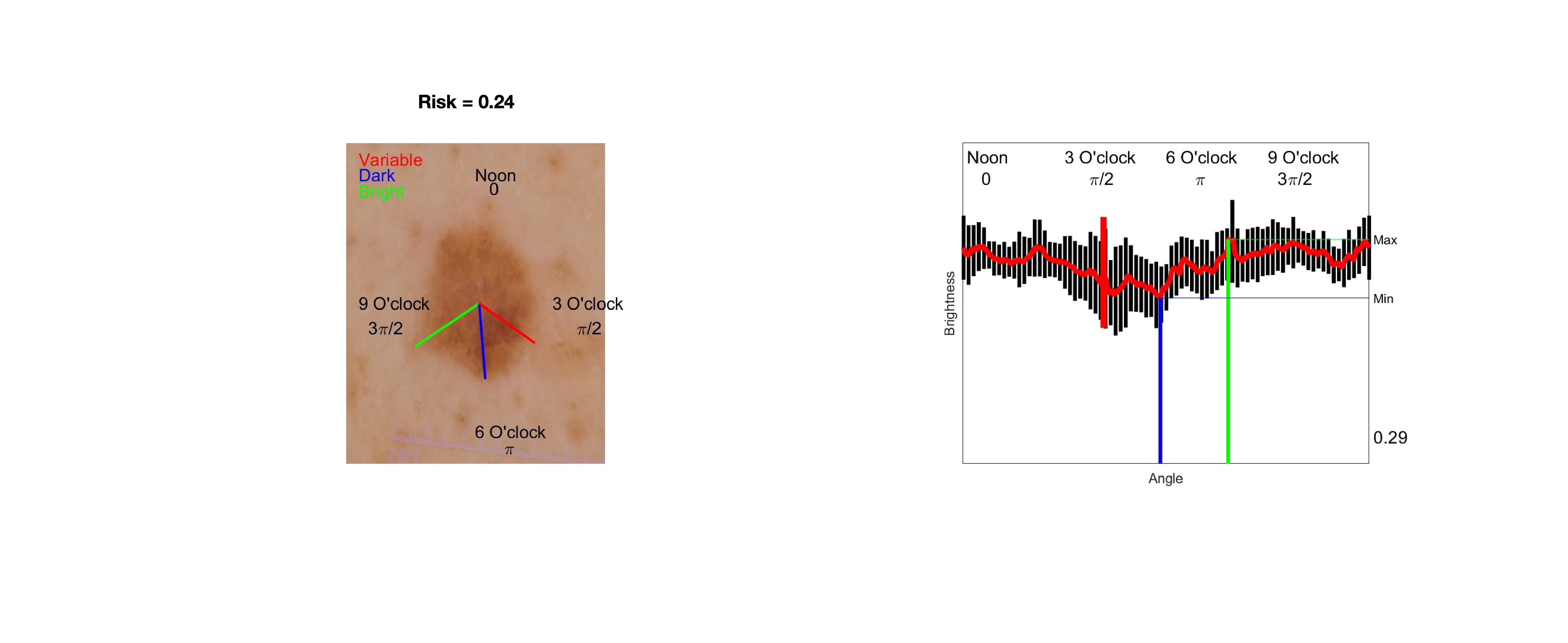

Supplement: Supplementary file 1 [file cancers-16-03077-s001.zip › cancers-3154863-supplementary/Supplementary File 2/046B.jpg]

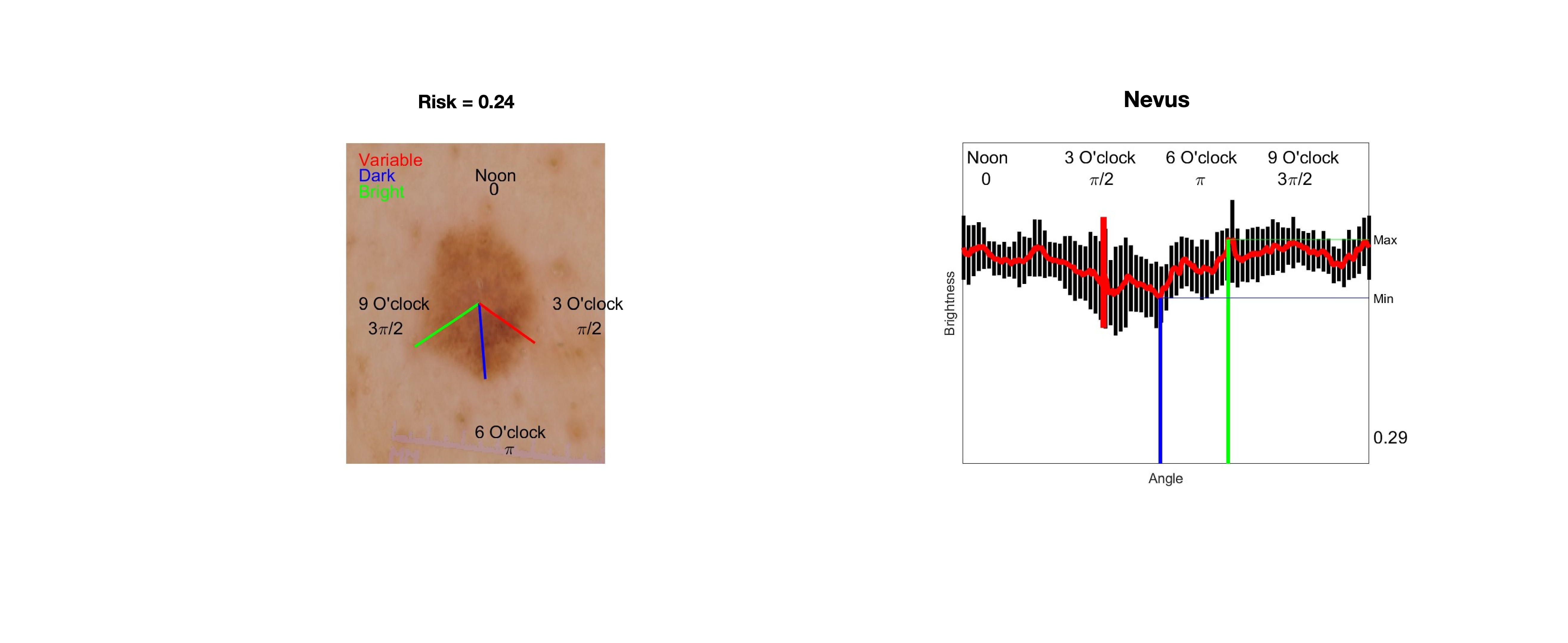

Supplement: Supplementary file 1 [file cancers-16-03077-s001.zip › cancers-3154863-supplementary/Supplementary File 2/046C.jpg]

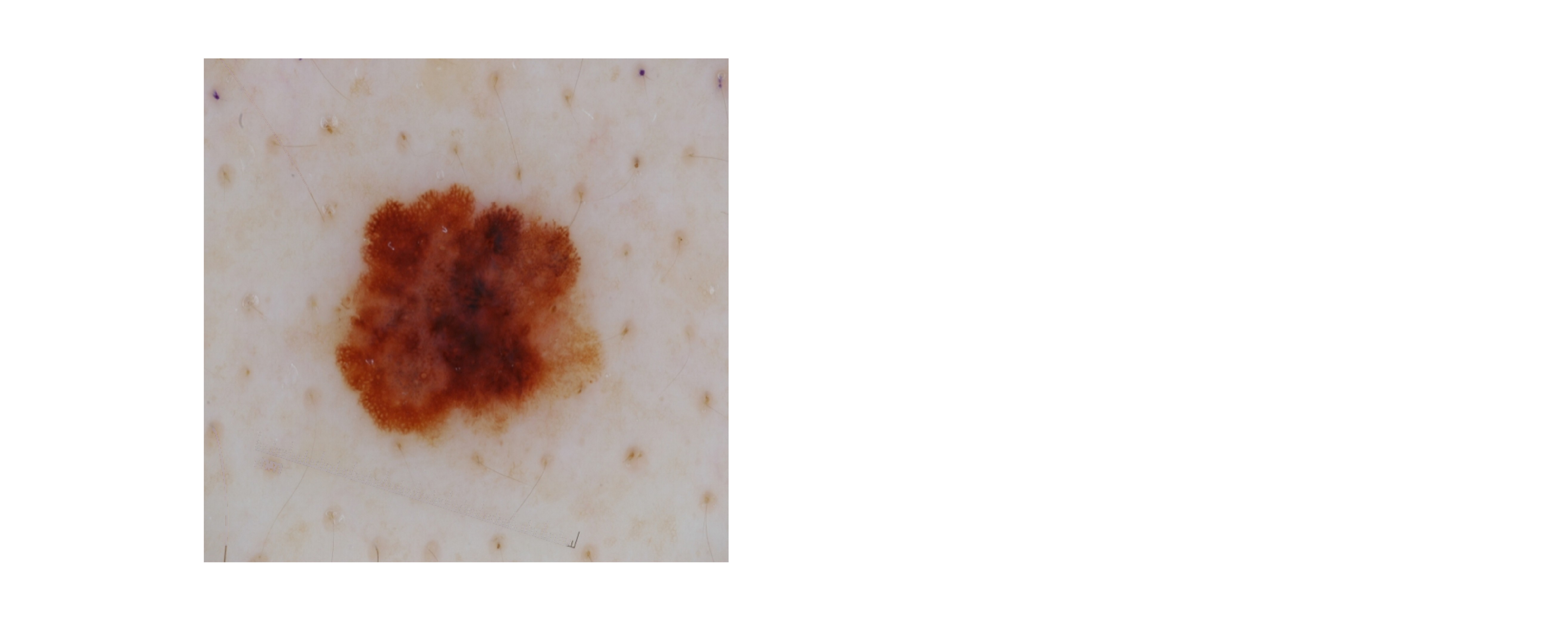

Supplement: Supplementary file 1 [file cancers-16-03077-s001.zip › cancers-3154863-supplementary/Supplementary File 2/047A.jpg]

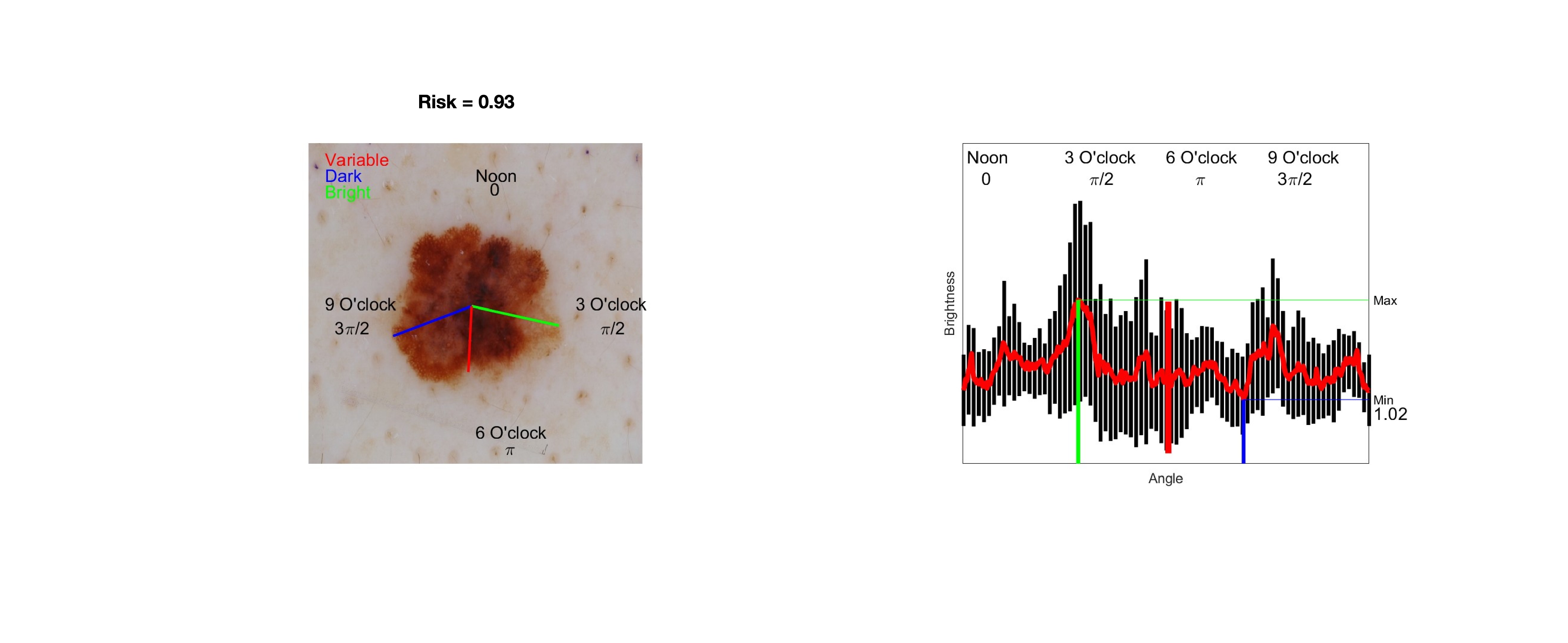

Supplement: Supplementary file 1 [file cancers-16-03077-s001.zip › cancers-3154863-supplementary/Supplementary File 2/047B.jpg]

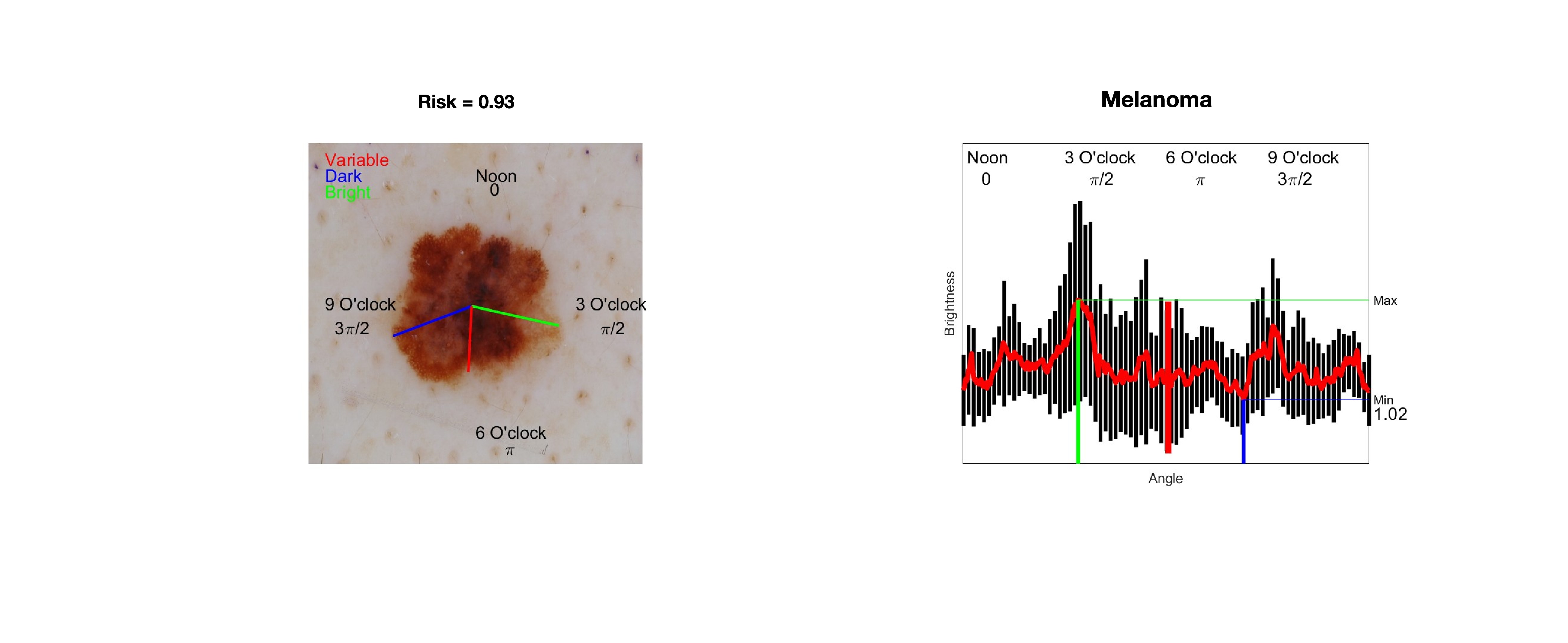

Supplement: Supplementary file 1 [file cancers-16-03077-s001.zip › cancers-3154863-supplementary/Supplementary File 2/047C.jpg]

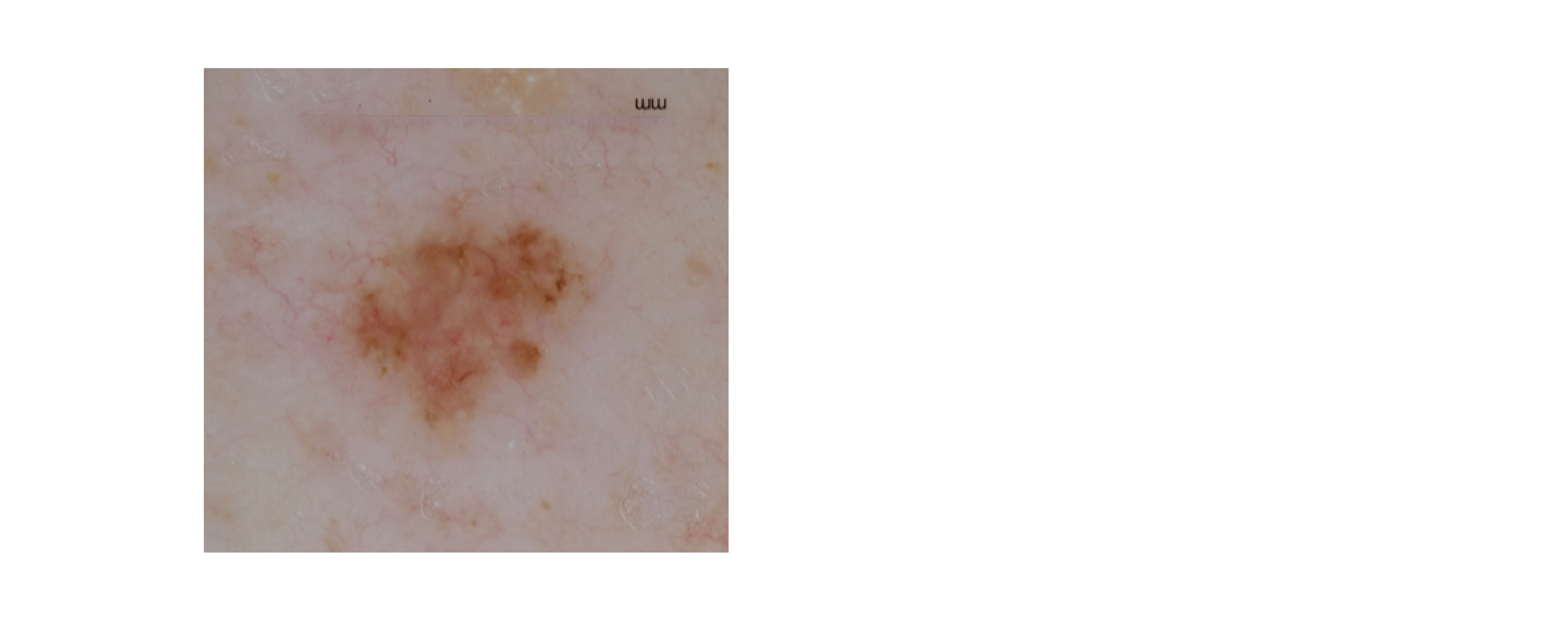

Supplement: Supplementary file 1 [file cancers-16-03077-s001.zip › cancers-3154863-supplementary/Supplementary File 2/048A.jpg]

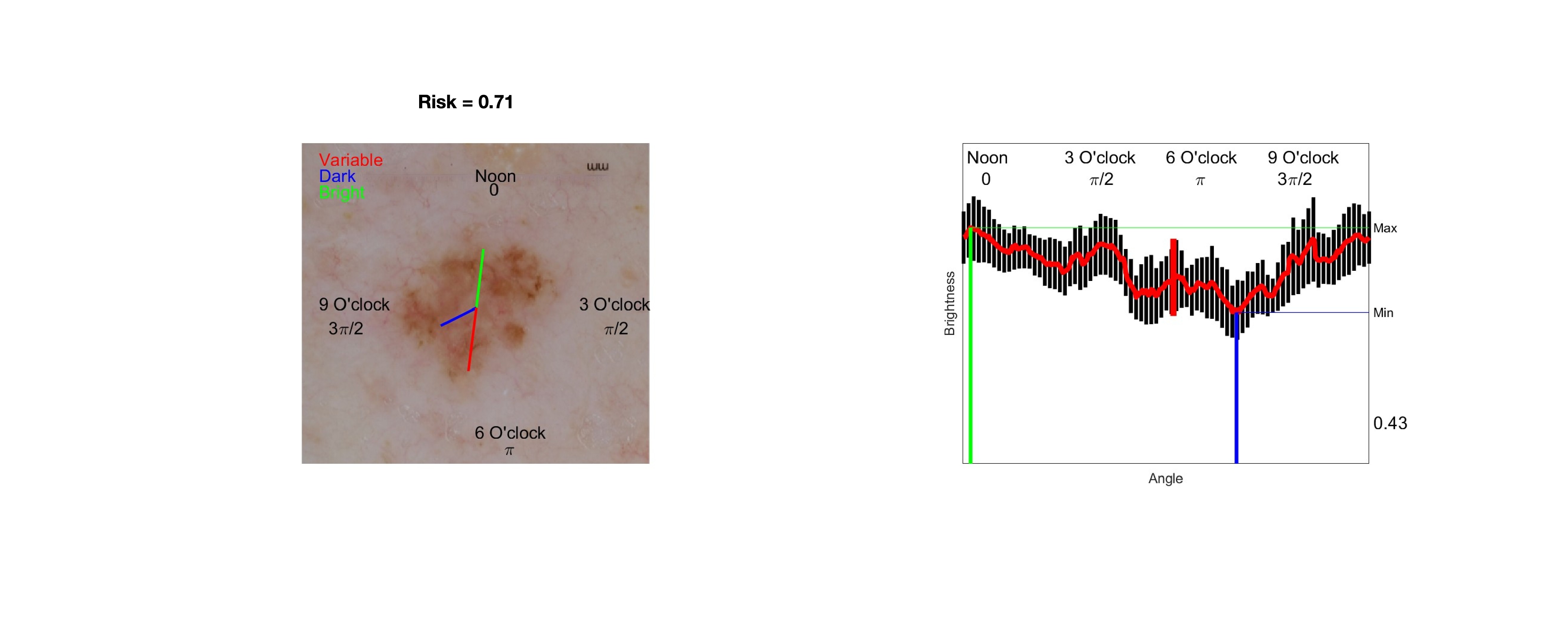

Supplement: Supplementary file 1 [file cancers-16-03077-s001.zip › cancers-3154863-supplementary/Supplementary File 2/048B.jpg]

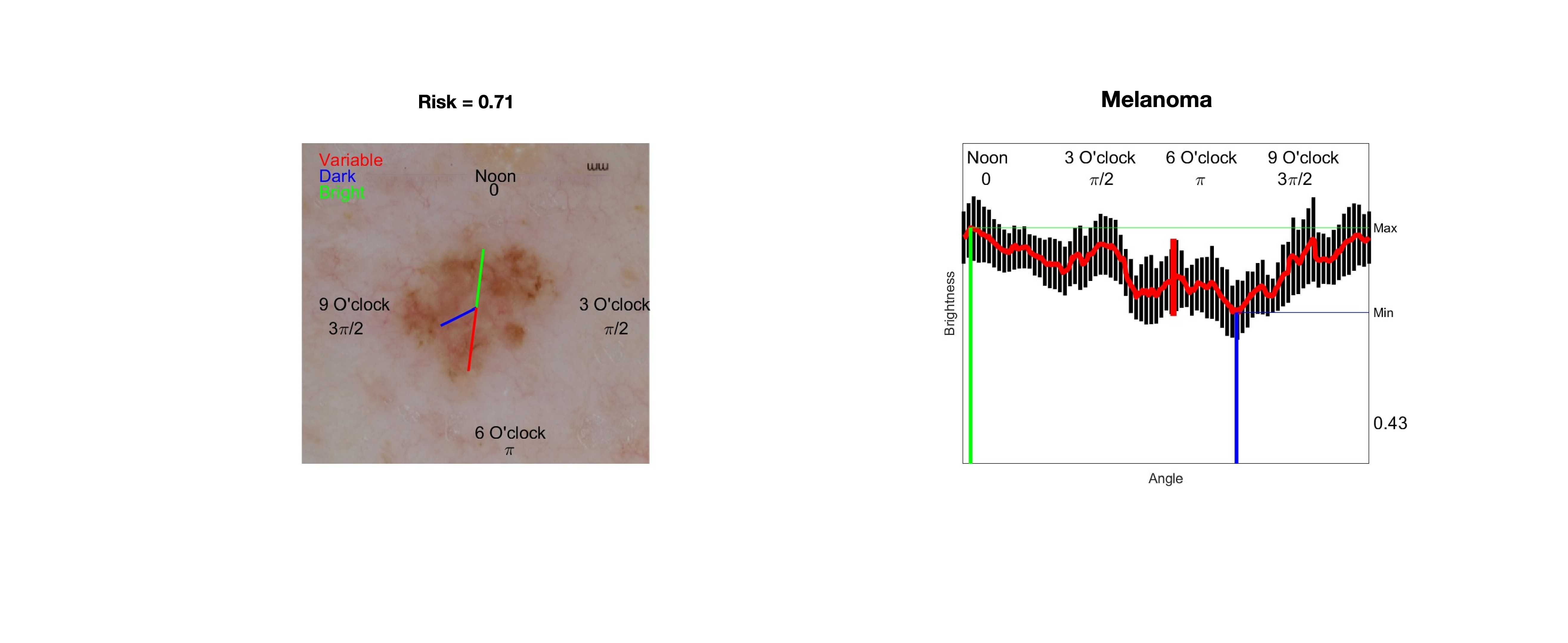

Supplement: Supplementary file 1 [file cancers-16-03077-s001.zip › cancers-3154863-supplementary/Supplementary File 2/048C.jpg]

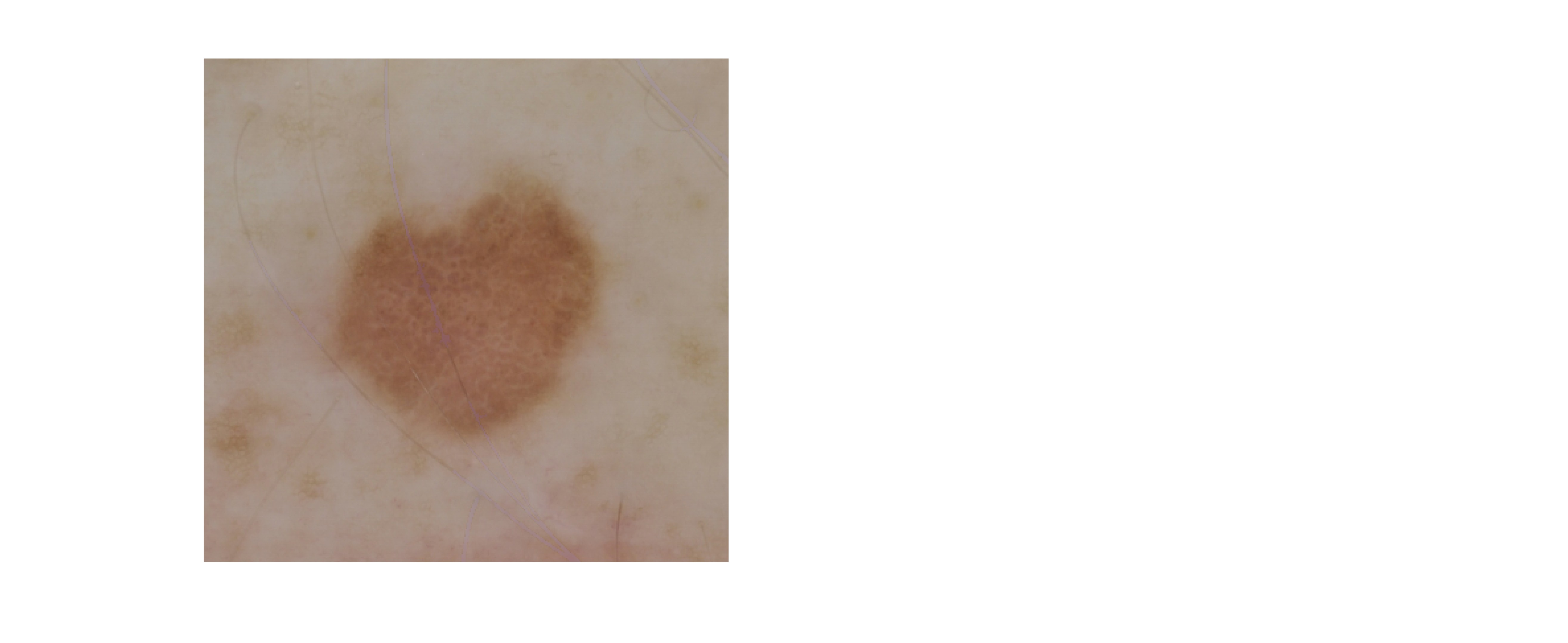

Supplement: Supplementary file 1 [file cancers-16-03077-s001.zip › cancers-3154863-supplementary/Supplementary File 2/049A.jpg]

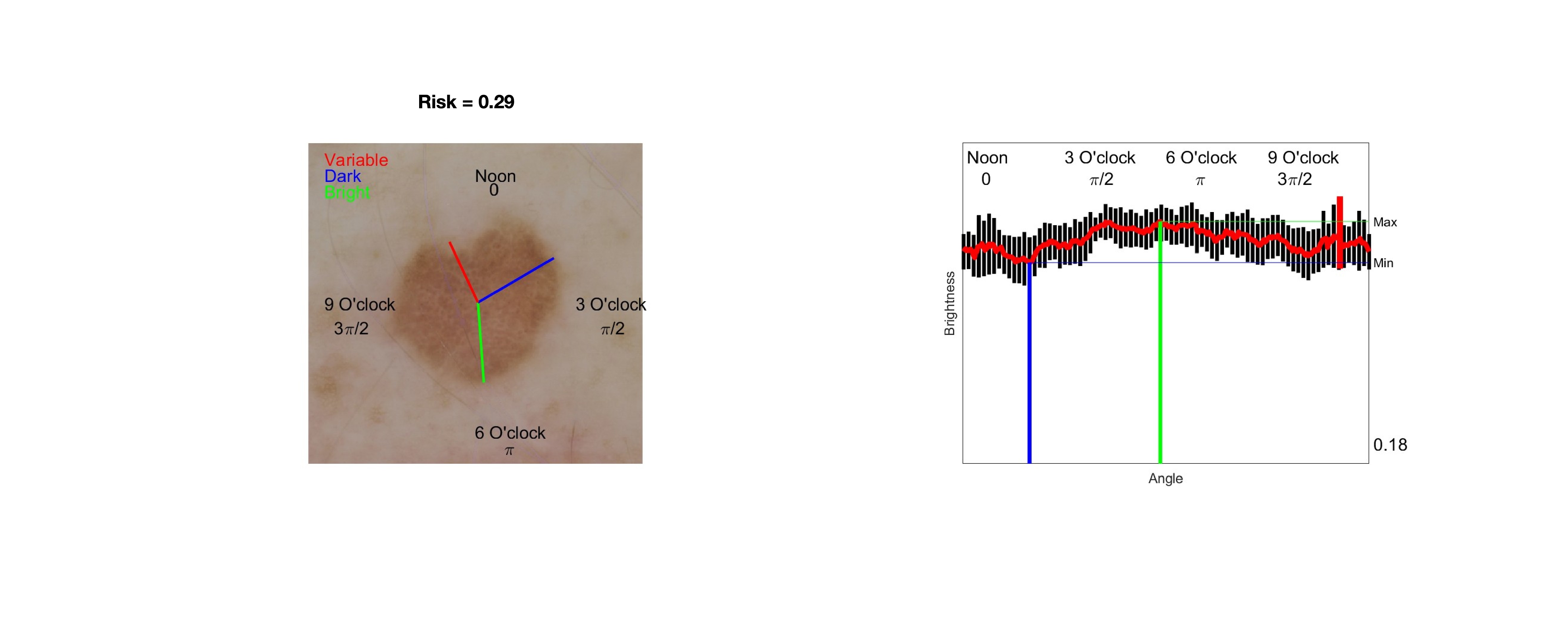

Supplement: Supplementary file 1 [file cancers-16-03077-s001.zip › cancers-3154863-supplementary/Supplementary File 2/049B.jpg]

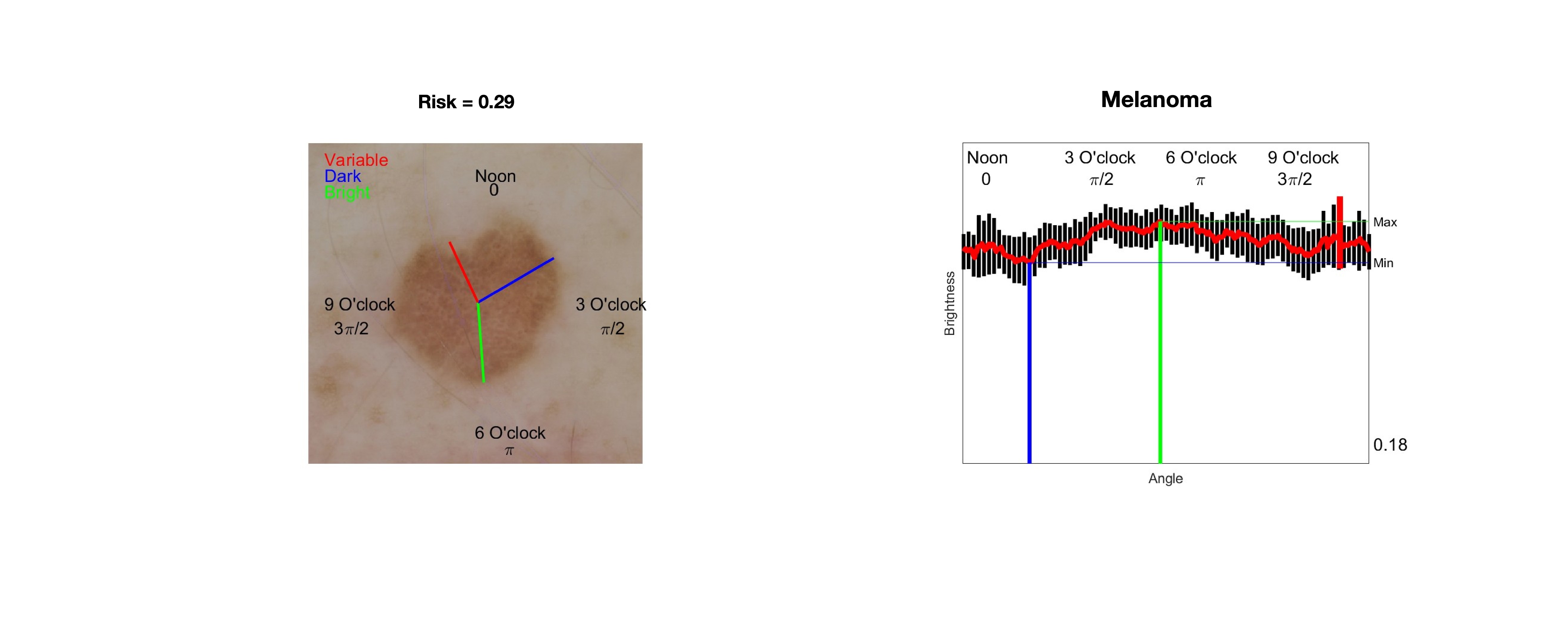

Supplement: Supplementary file 1 [file cancers-16-03077-s001.zip › cancers-3154863-supplementary/Supplementary File 2/049C.jpg]

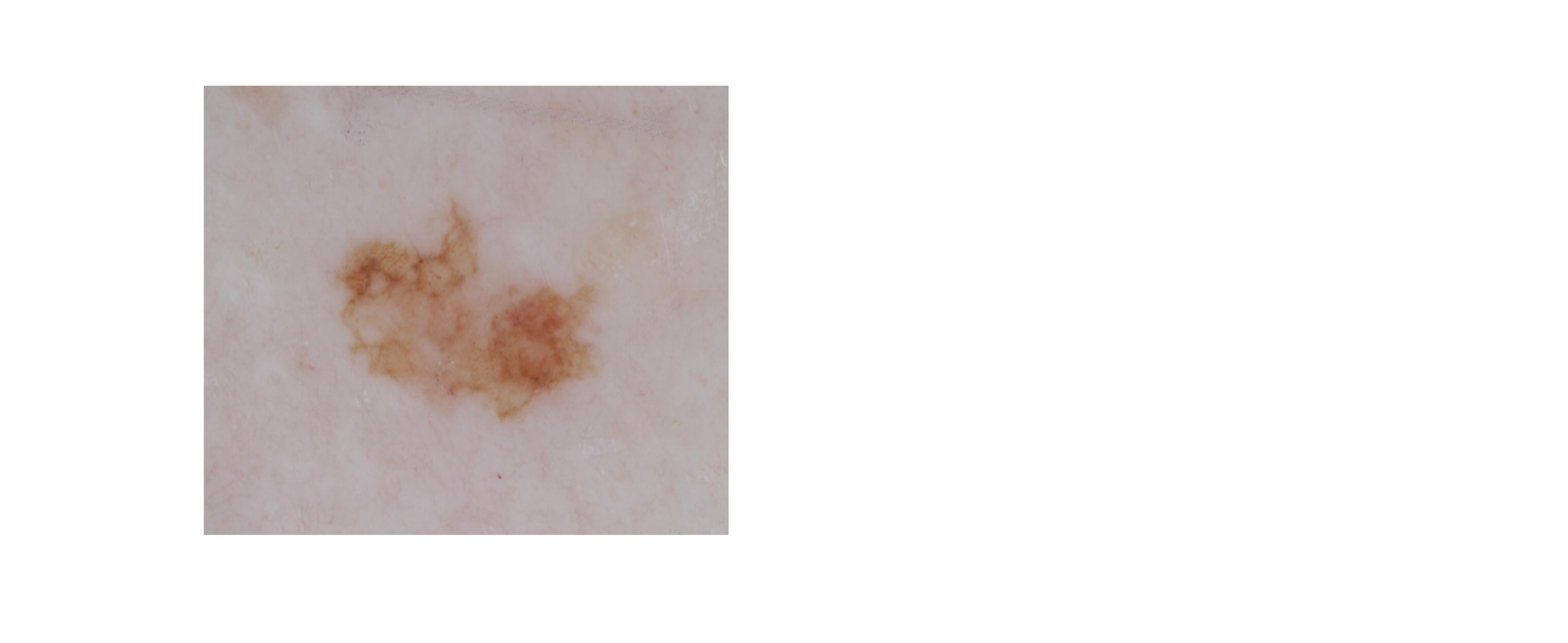

Supplement: Supplementary file 1 [file cancers-16-03077-s001.zip › cancers-3154863-supplementary/Supplementary File 2/050A.jpg]

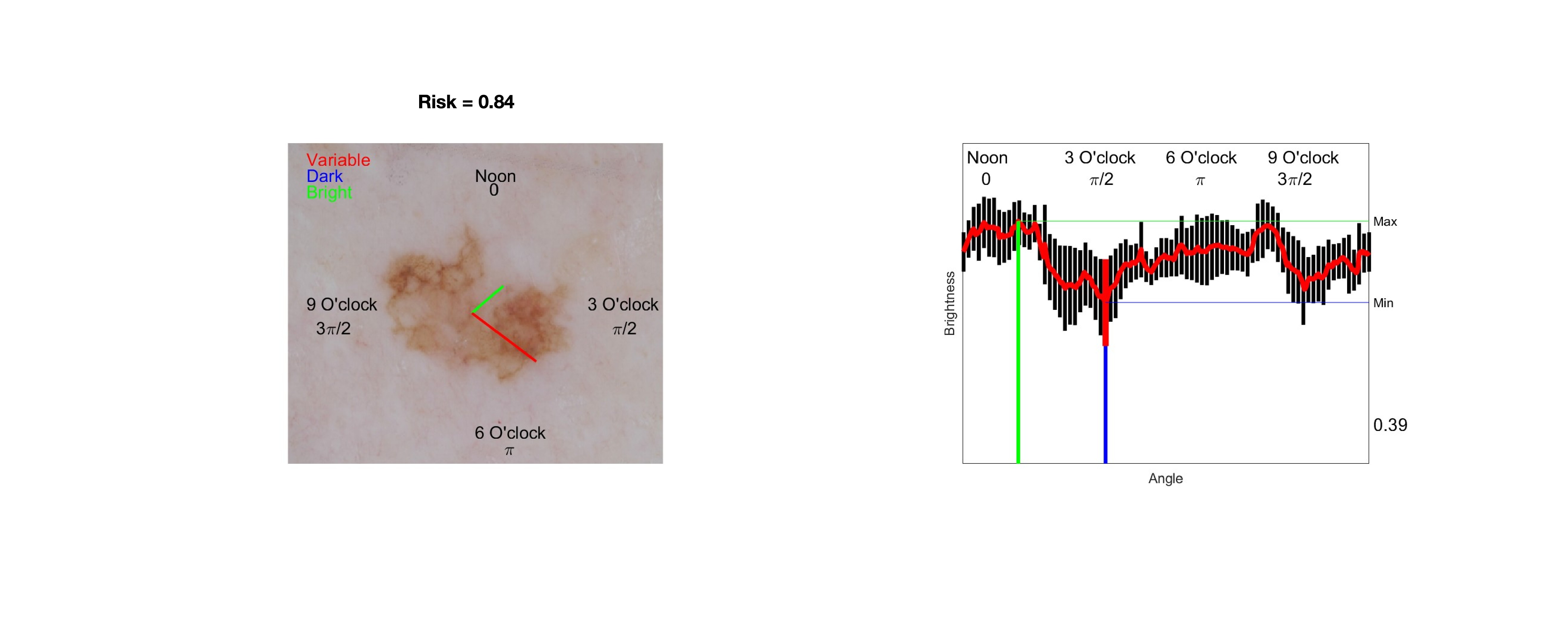

Supplement: Supplementary file 1 [file cancers-16-03077-s001.zip › cancers-3154863-supplementary/Supplementary File 2/050B.jpg]

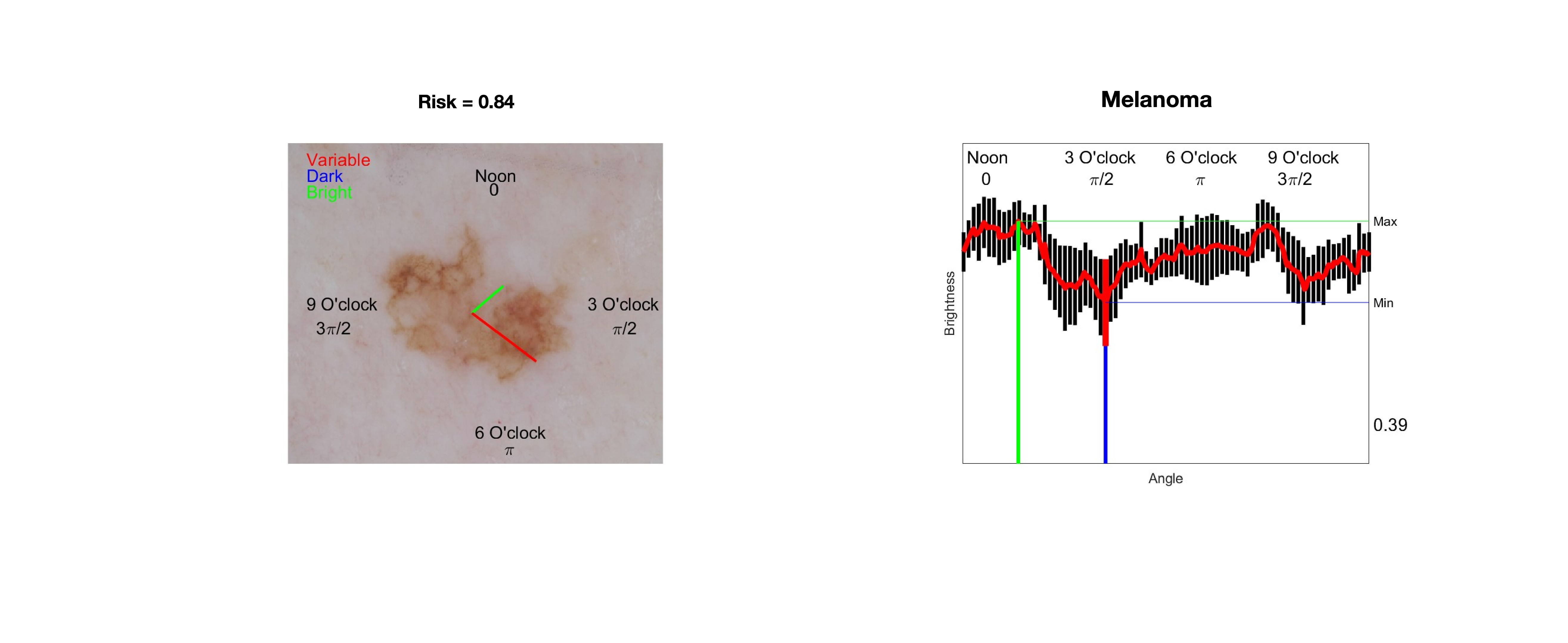

Supplement: Supplementary file 1 [file cancers-16-03077-s001.zip › cancers-3154863-supplementary/Supplementary File 2/050C.jpg]

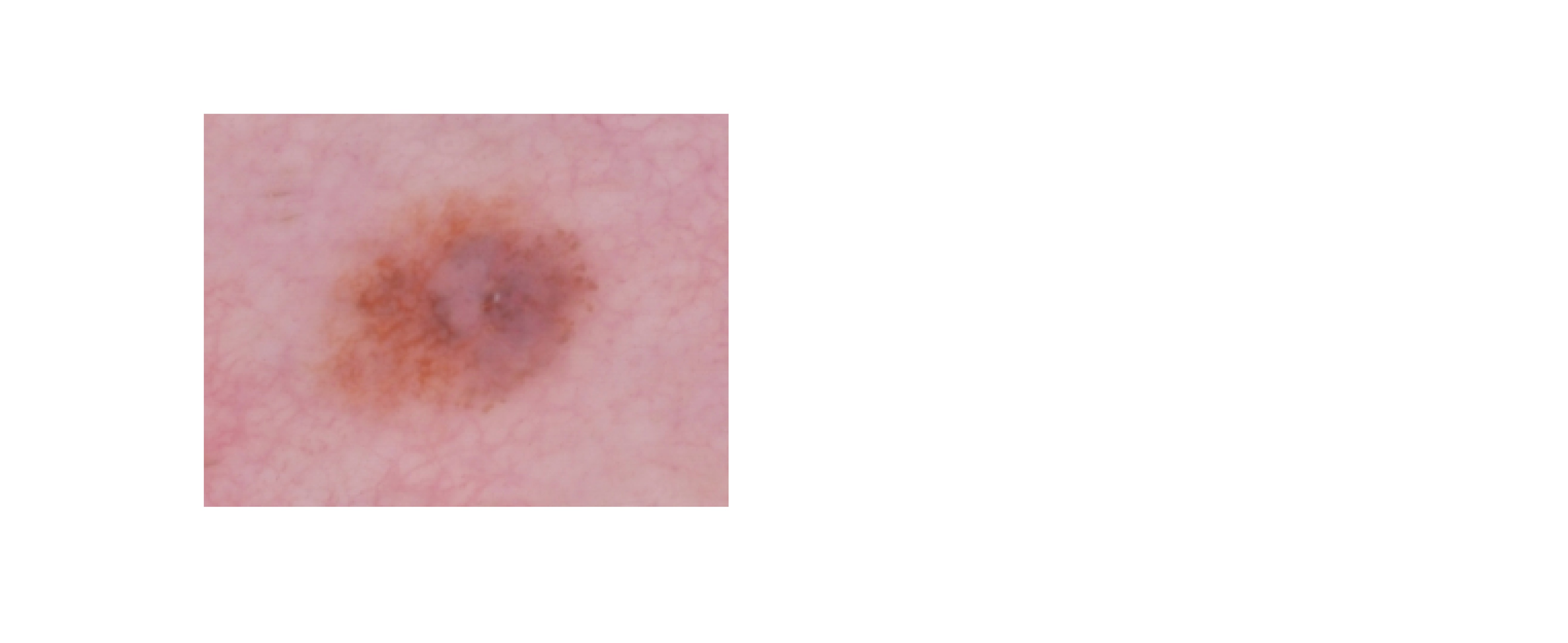

Supplement: Supplementary file 1 [file cancers-16-03077-s001.zip › cancers-3154863-supplementary/Supplementary File 2/051A.jpg]

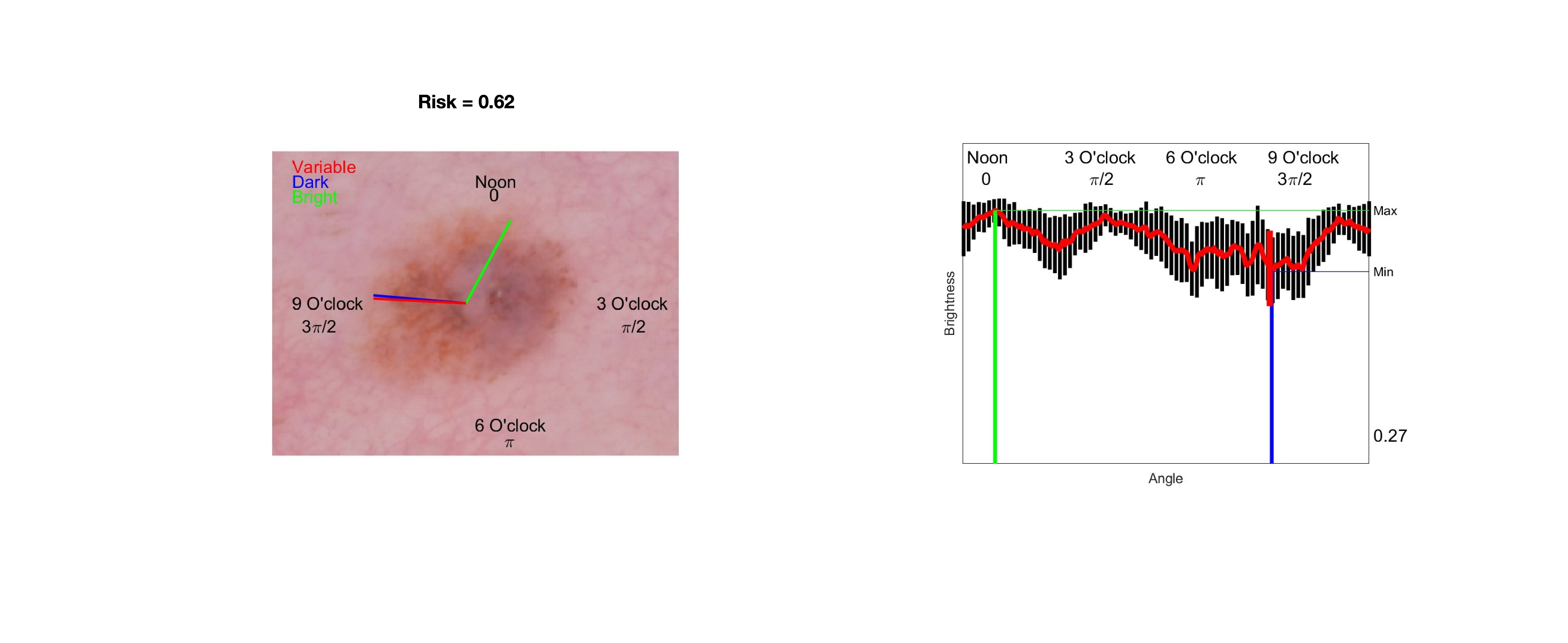

Supplement: Supplementary file 1 [file cancers-16-03077-s001.zip › cancers-3154863-supplementary/Supplementary File 2/051B.jpg]

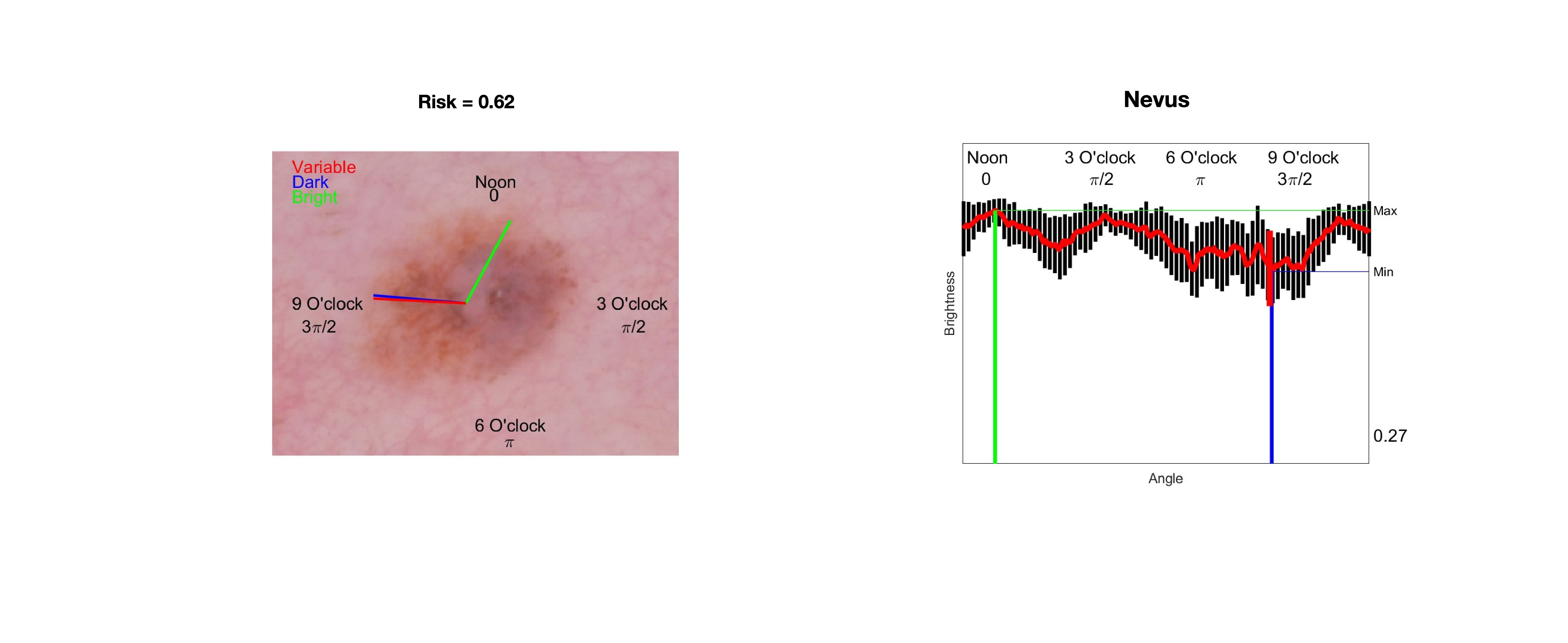

Supplement: Supplementary file 1 [file cancers-16-03077-s001.zip › cancers-3154863-supplementary/Supplementary File 2/051C.jpg]

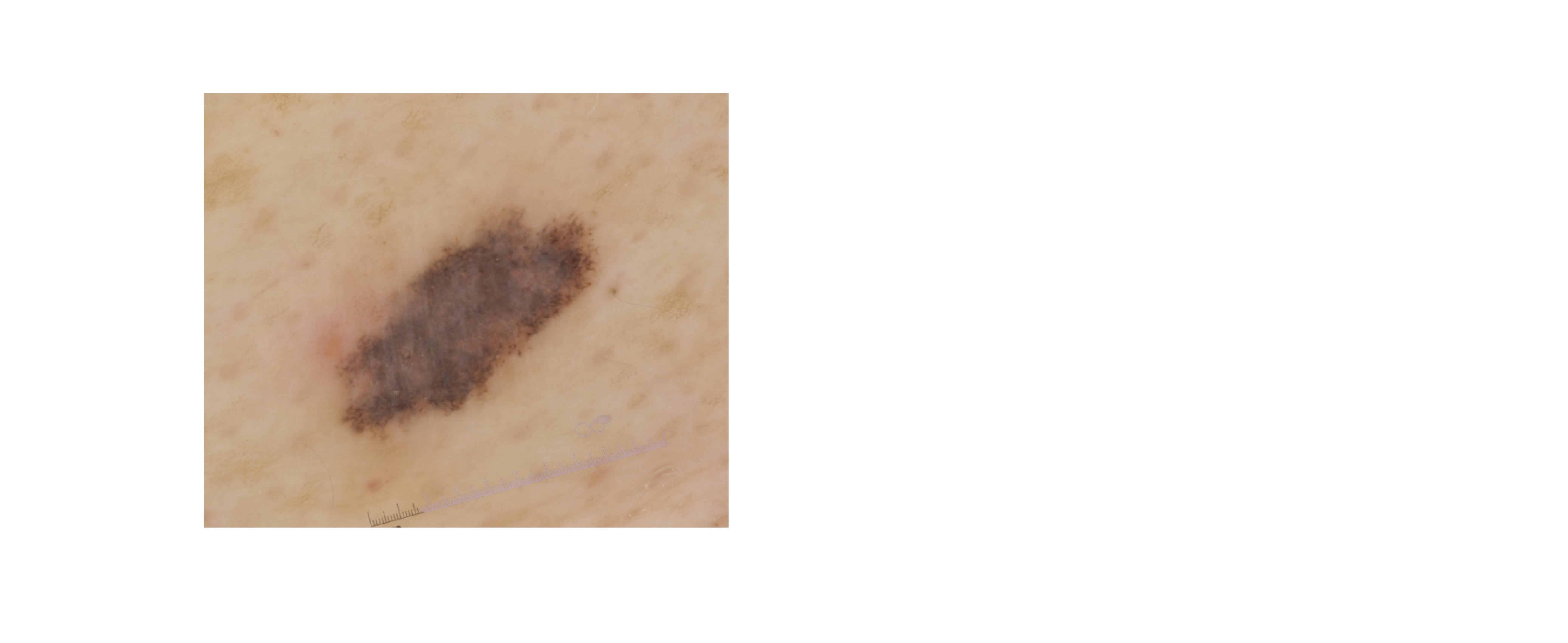

Supplement: Supplementary file 1 [file cancers-16-03077-s001.zip › cancers-3154863-supplementary/Supplementary File 2/052A.jpg]

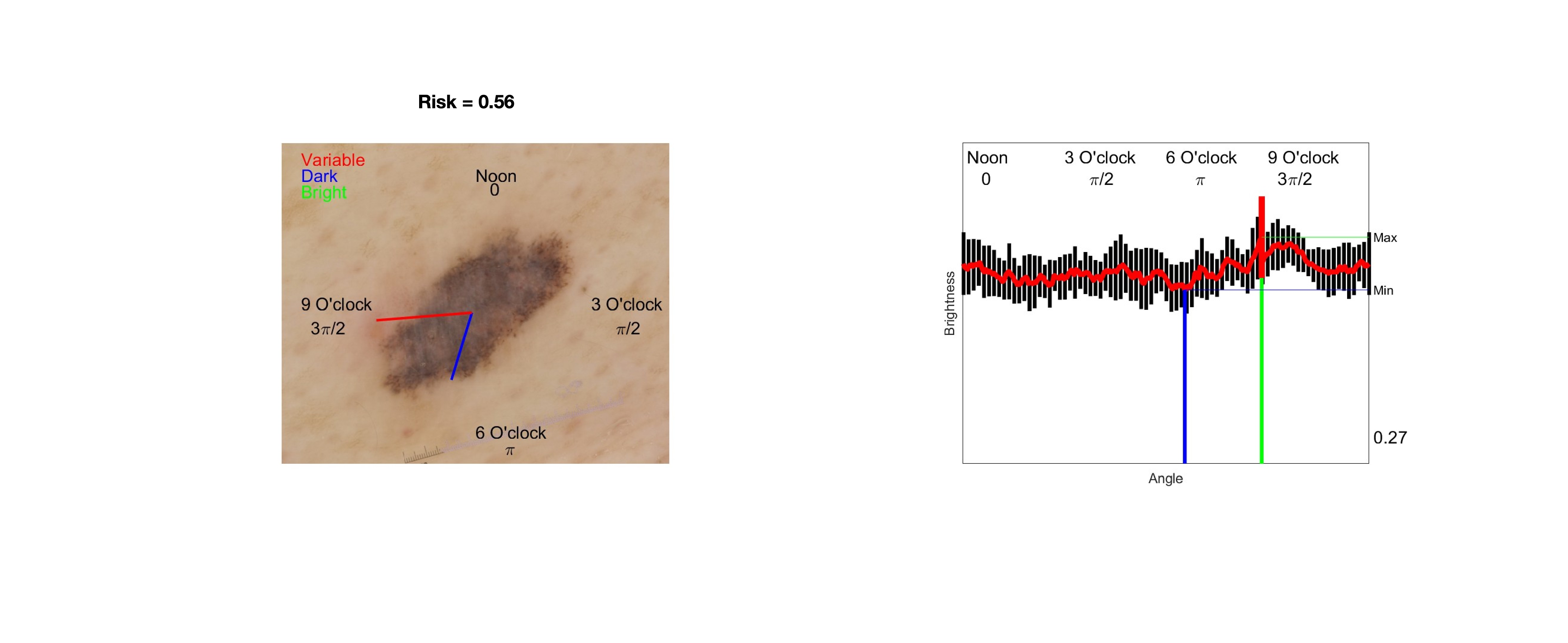

Supplement: Supplementary file 1 [file cancers-16-03077-s001.zip › cancers-3154863-supplementary/Supplementary File 2/052B.jpg]

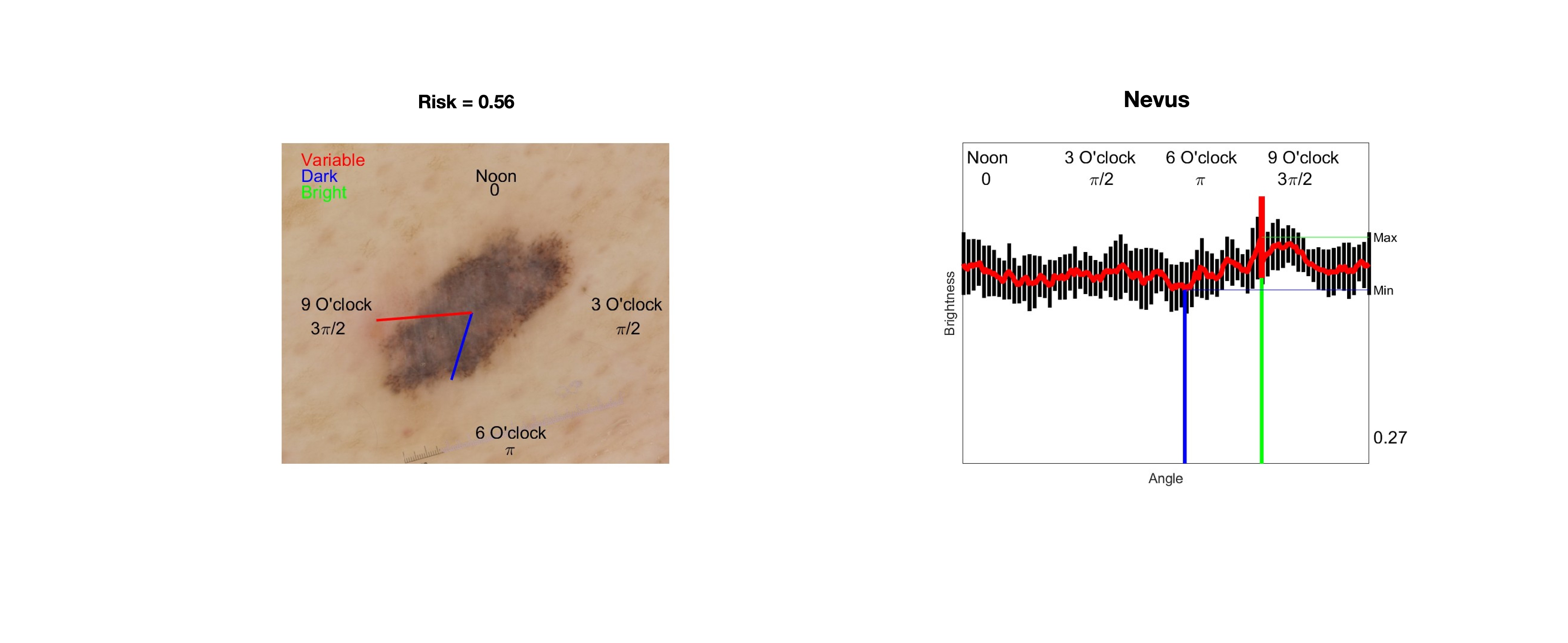

Supplement: Supplementary file 1 [file cancers-16-03077-s001.zip › cancers-3154863-supplementary/Supplementary File 2/052C.jpg]

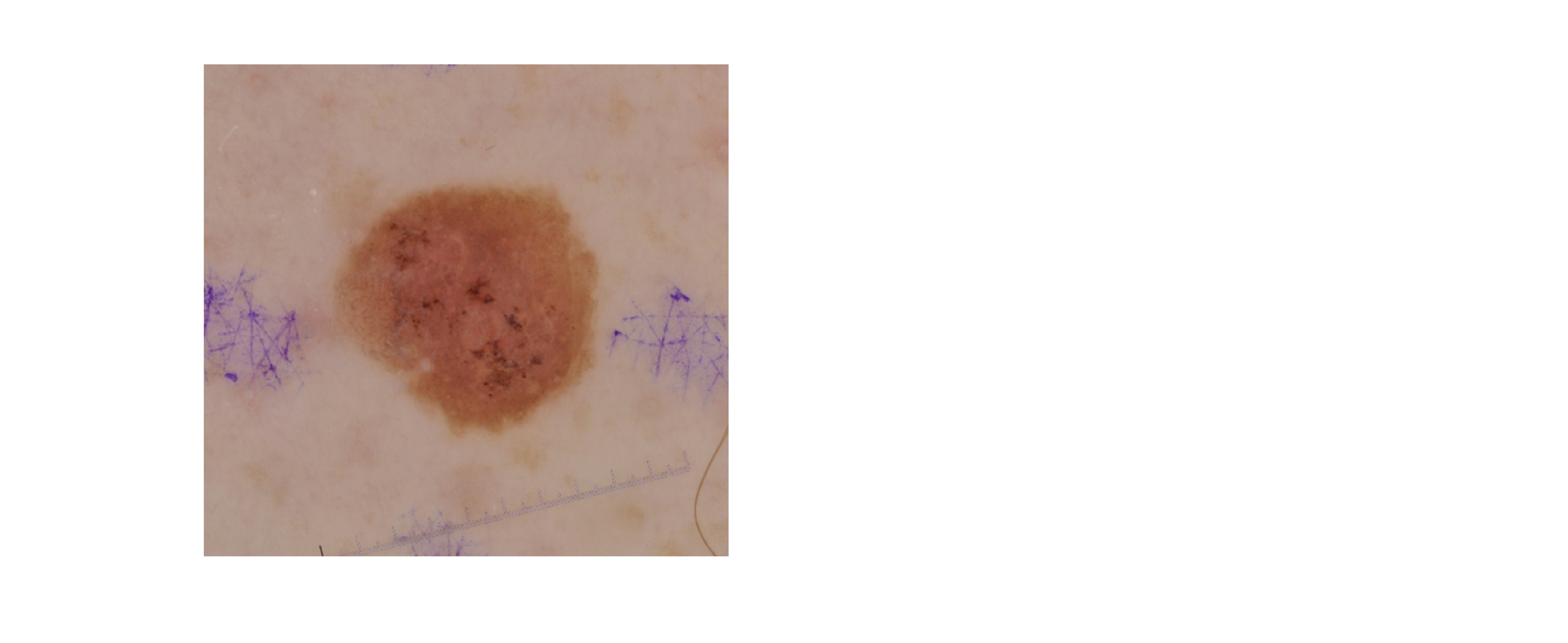

Supplement: Supplementary file 1 [file cancers-16-03077-s001.zip › cancers-3154863-supplementary/Supplementary File 2/053A.jpg]

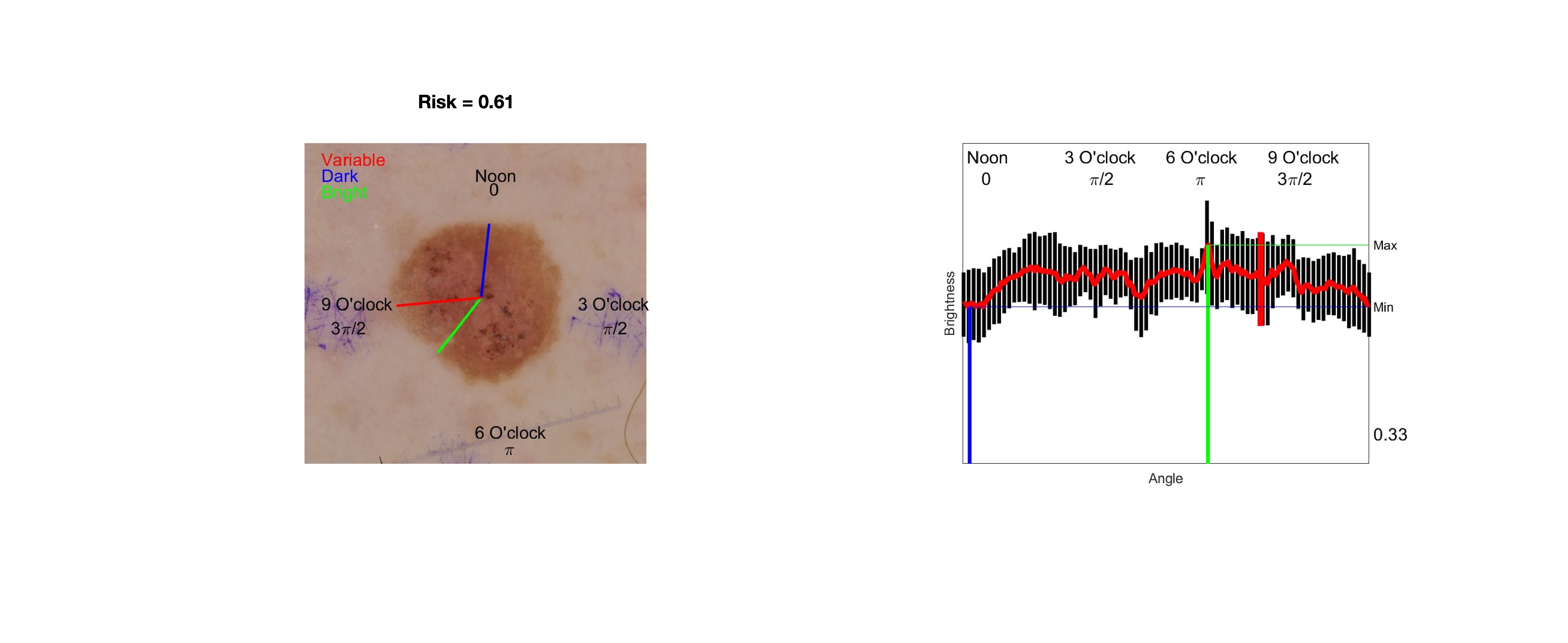

Supplement: Supplementary file 1 [file cancers-16-03077-s001.zip › cancers-3154863-supplementary/Supplementary File 2/053B.jpg]

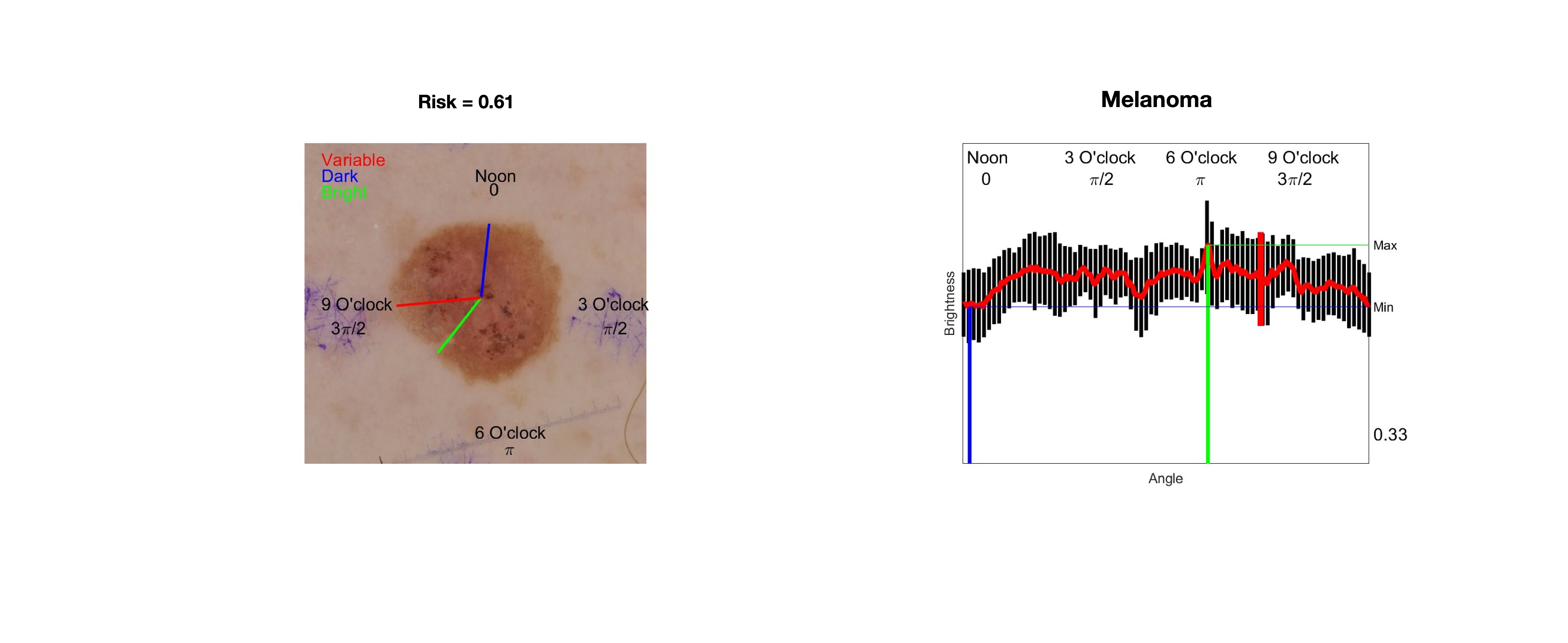

Supplement: Supplementary file 1 [file cancers-16-03077-s001.zip › cancers-3154863-supplementary/Supplementary File 2/053C.jpg]

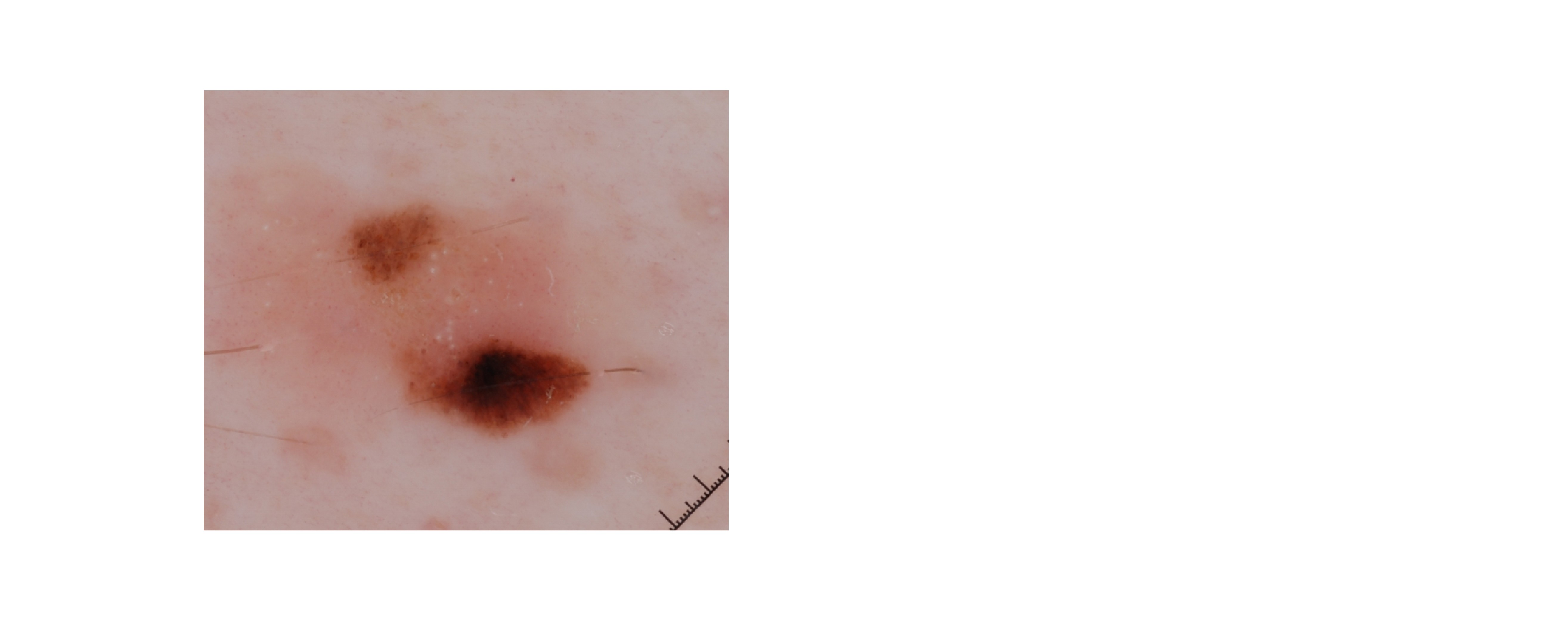

Supplement: Supplementary file 1 [file cancers-16-03077-s001.zip › cancers-3154863-supplementary/Supplementary File 2/054A.jpg]

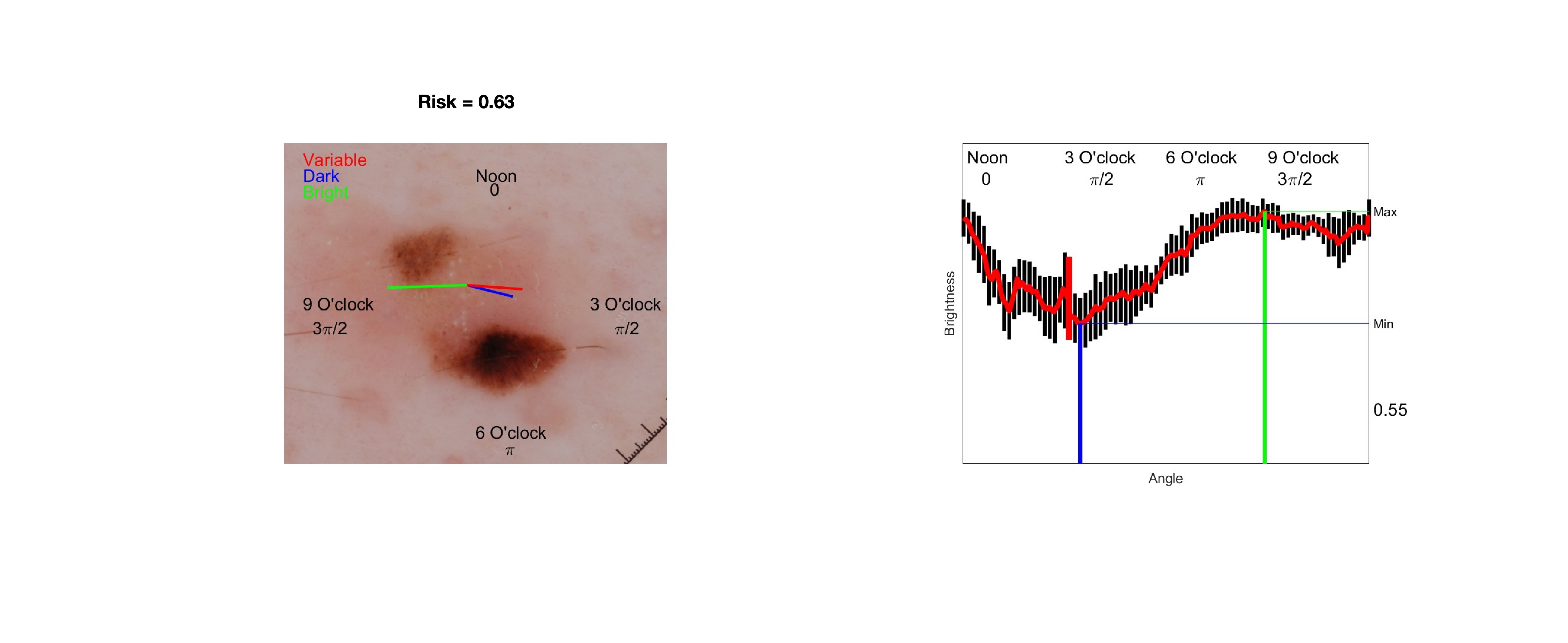

Supplement: Supplementary file 1 [file cancers-16-03077-s001.zip › cancers-3154863-supplementary/Supplementary File 2/054B.jpg]

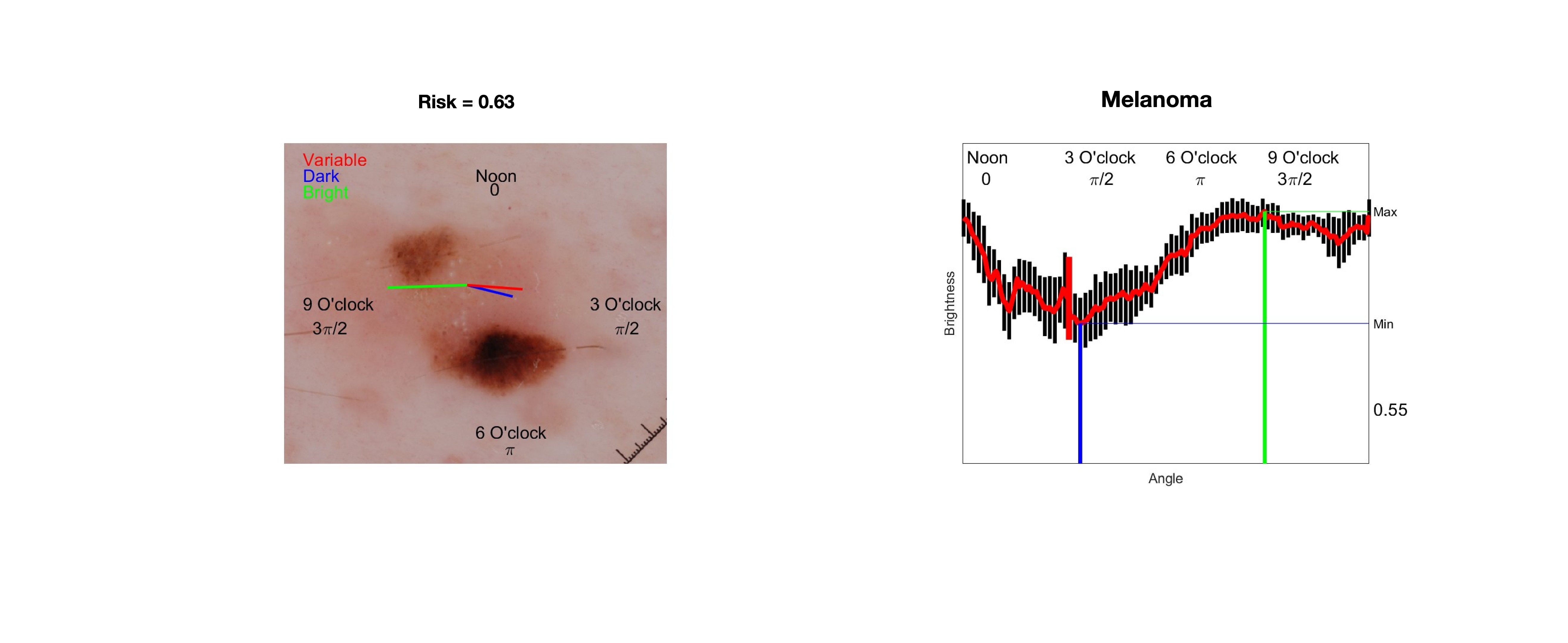

Supplement: Supplementary file 1 [file cancers-16-03077-s001.zip › cancers-3154863-supplementary/Supplementary File 2/054C.jpg]

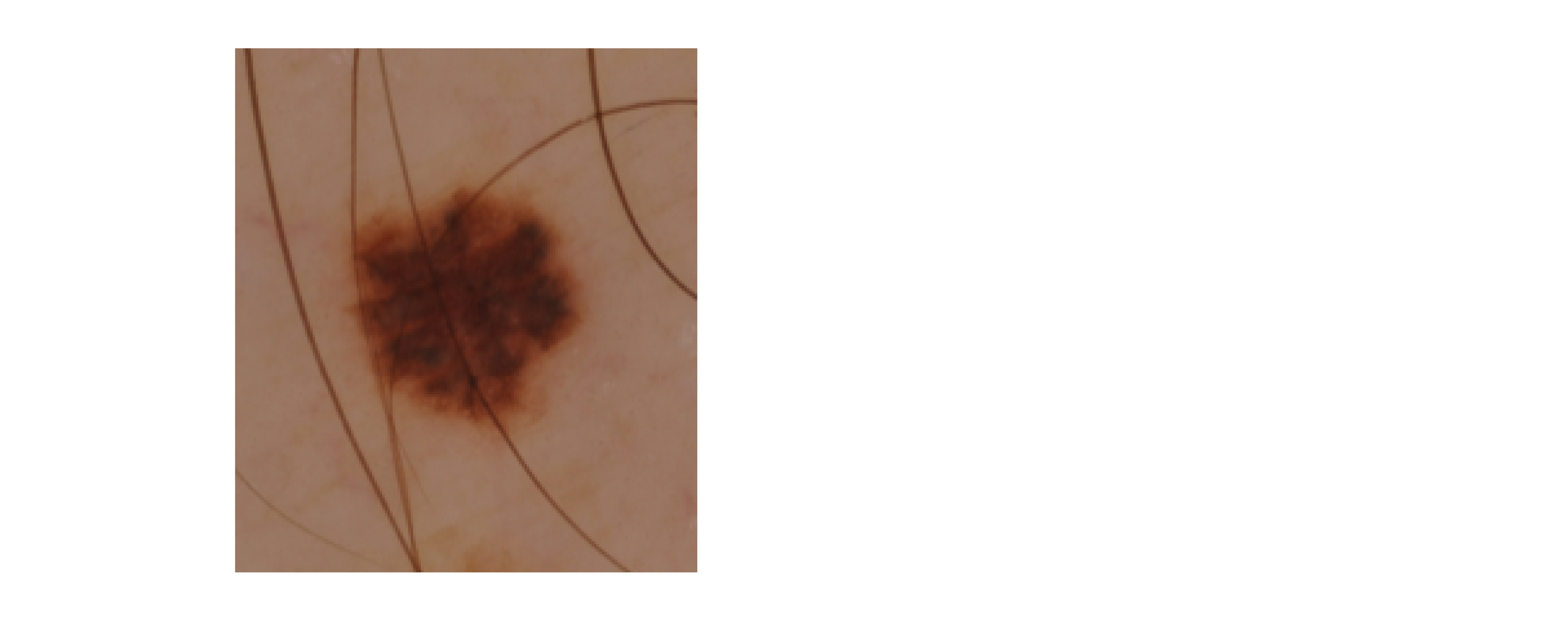

Supplement: Supplementary file 1 [file cancers-16-03077-s001.zip › cancers-3154863-supplementary/Supplementary File 2/055A.jpg]

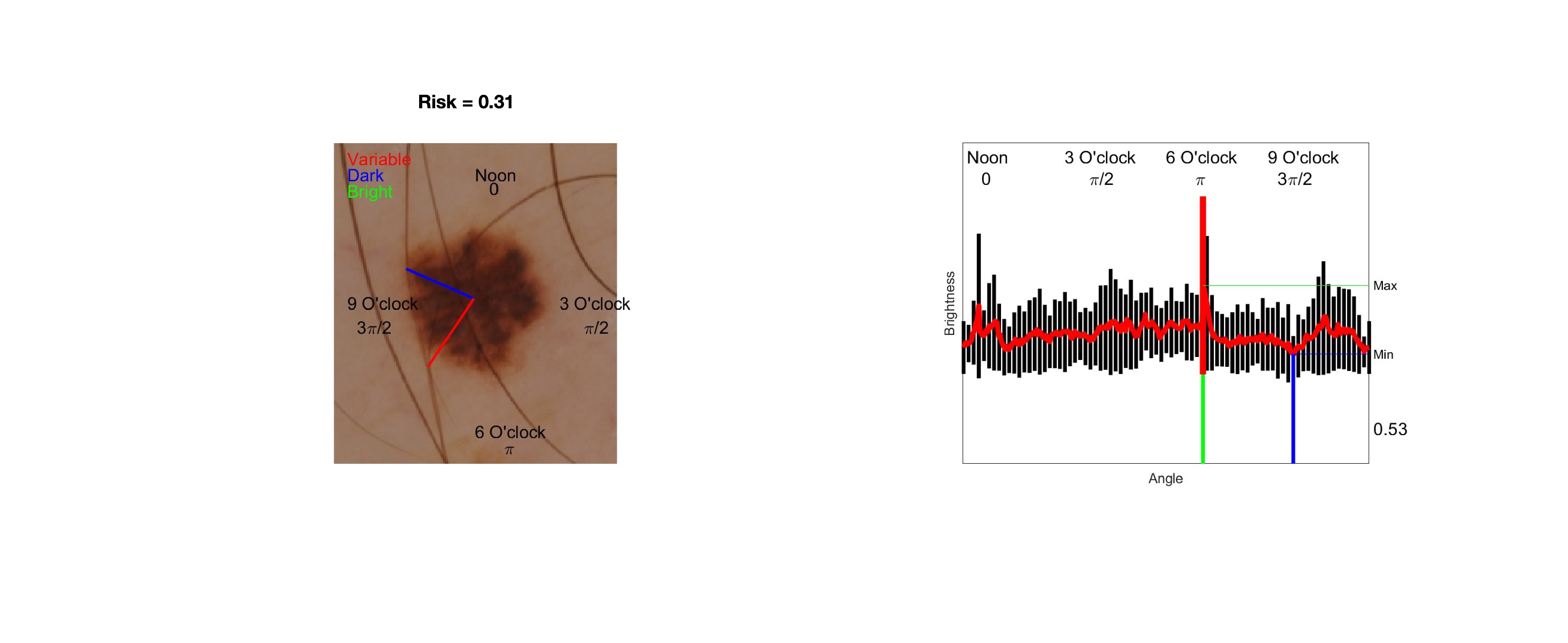

Supplement: Supplementary file 1 [file cancers-16-03077-s001.zip › cancers-3154863-supplementary/Supplementary File 2/055B.jpg]

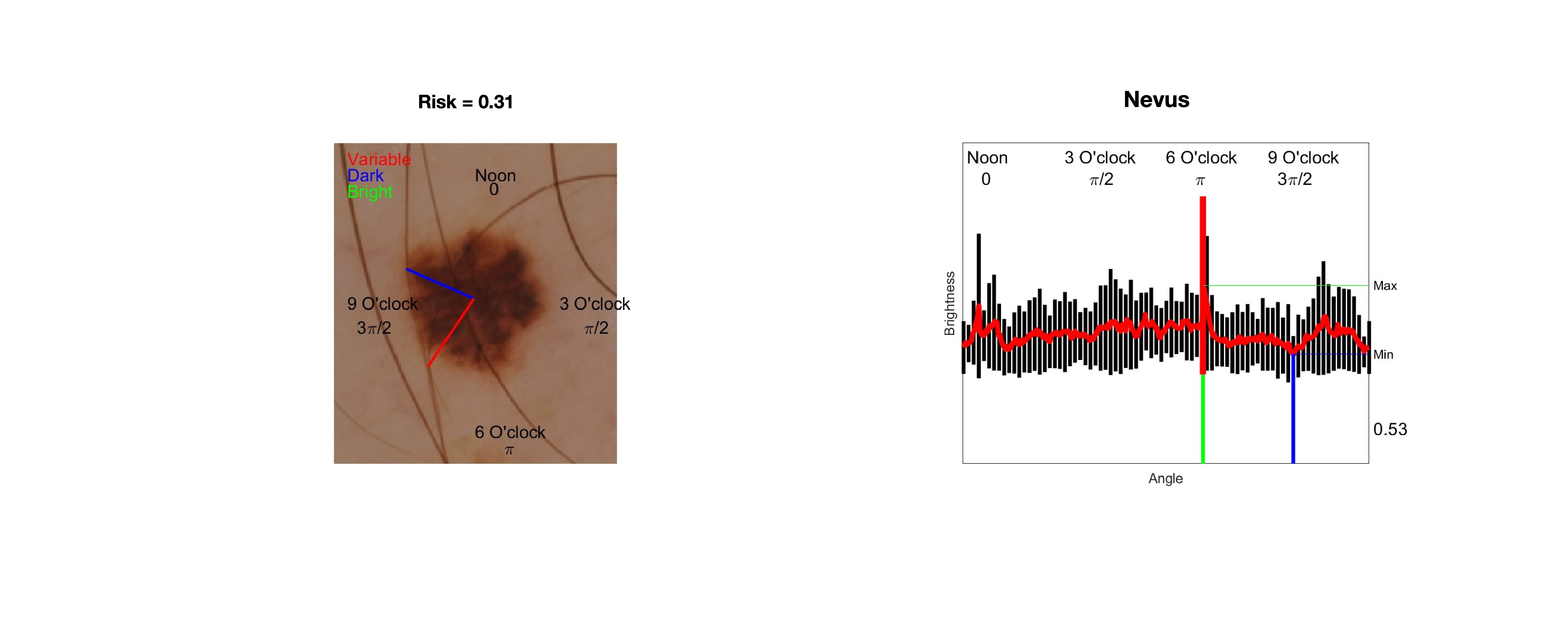

Supplement: Supplementary file 1 [file cancers-16-03077-s001.zip › cancers-3154863-supplementary/Supplementary File 2/055C.jpg]

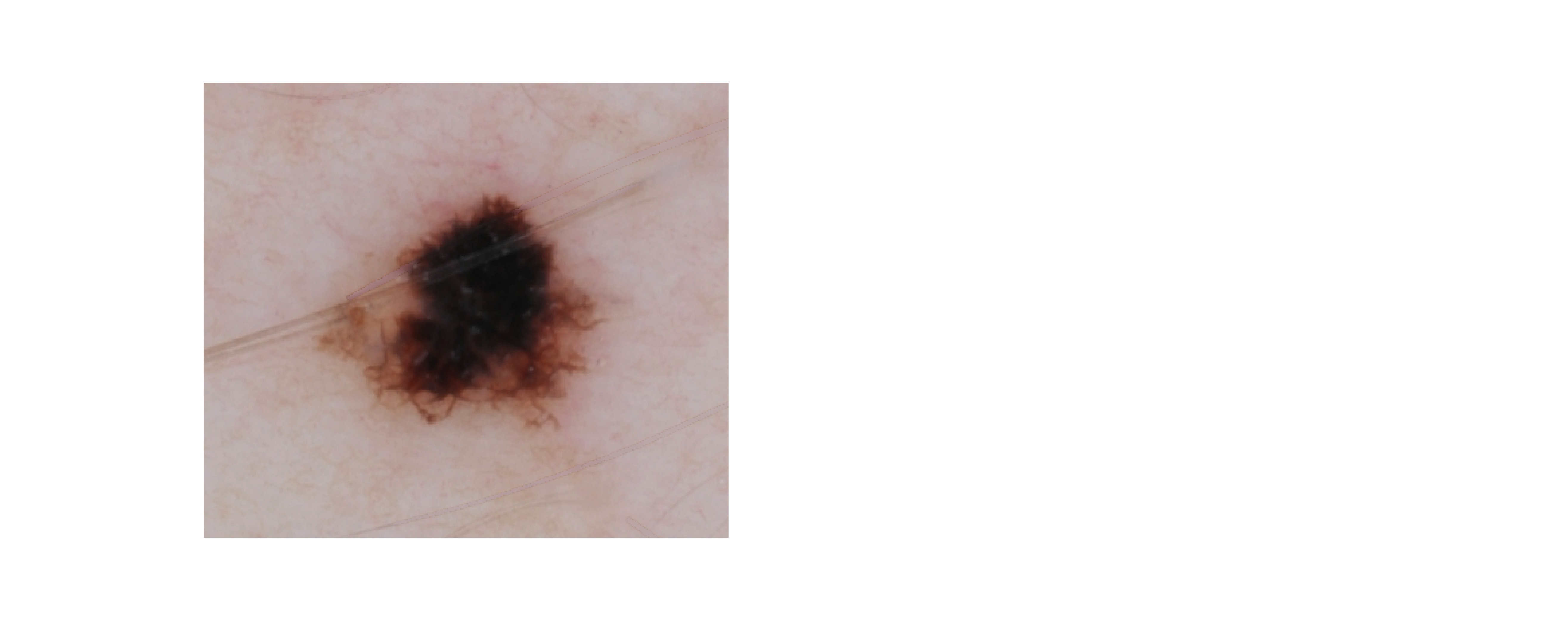

Supplement: Supplementary file 1 [file cancers-16-03077-s001.zip › cancers-3154863-supplementary/Supplementary File 2/056A.jpg]

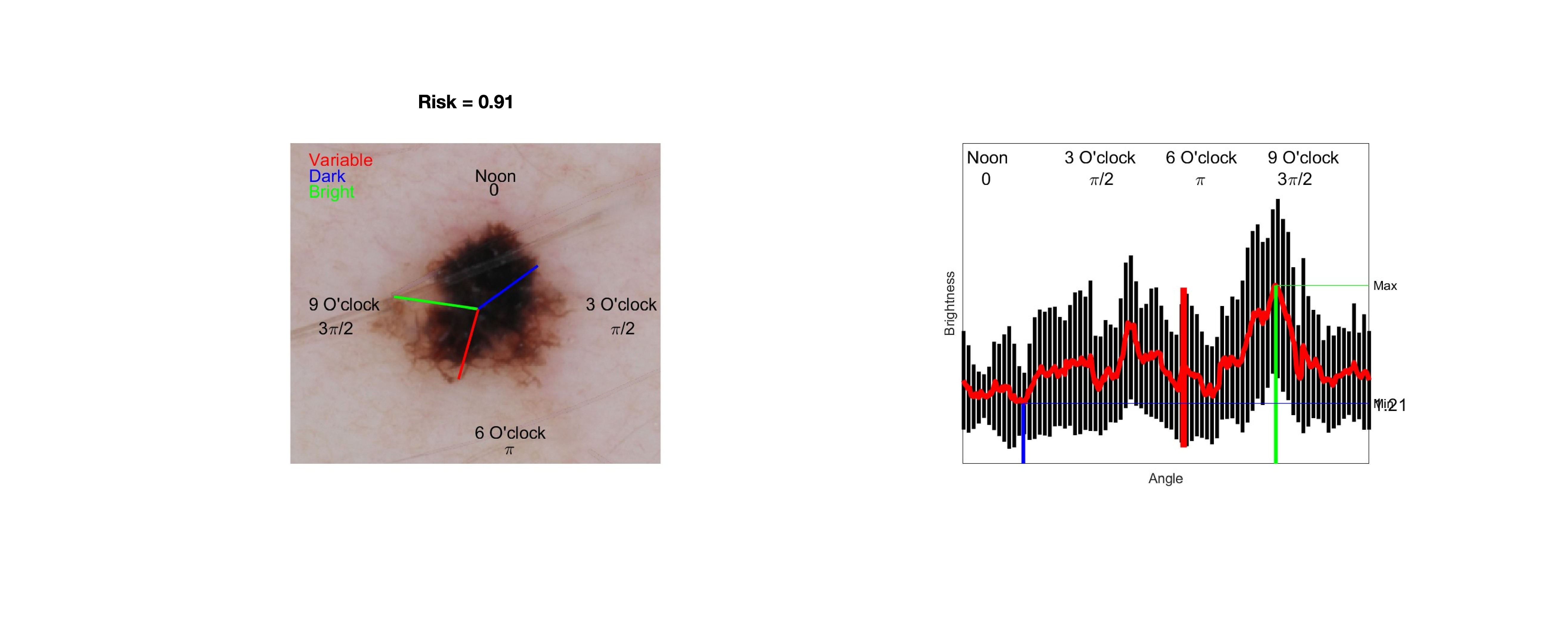

Supplement: Supplementary file 1 [file cancers-16-03077-s001.zip › cancers-3154863-supplementary/Supplementary File 2/056B.jpg]

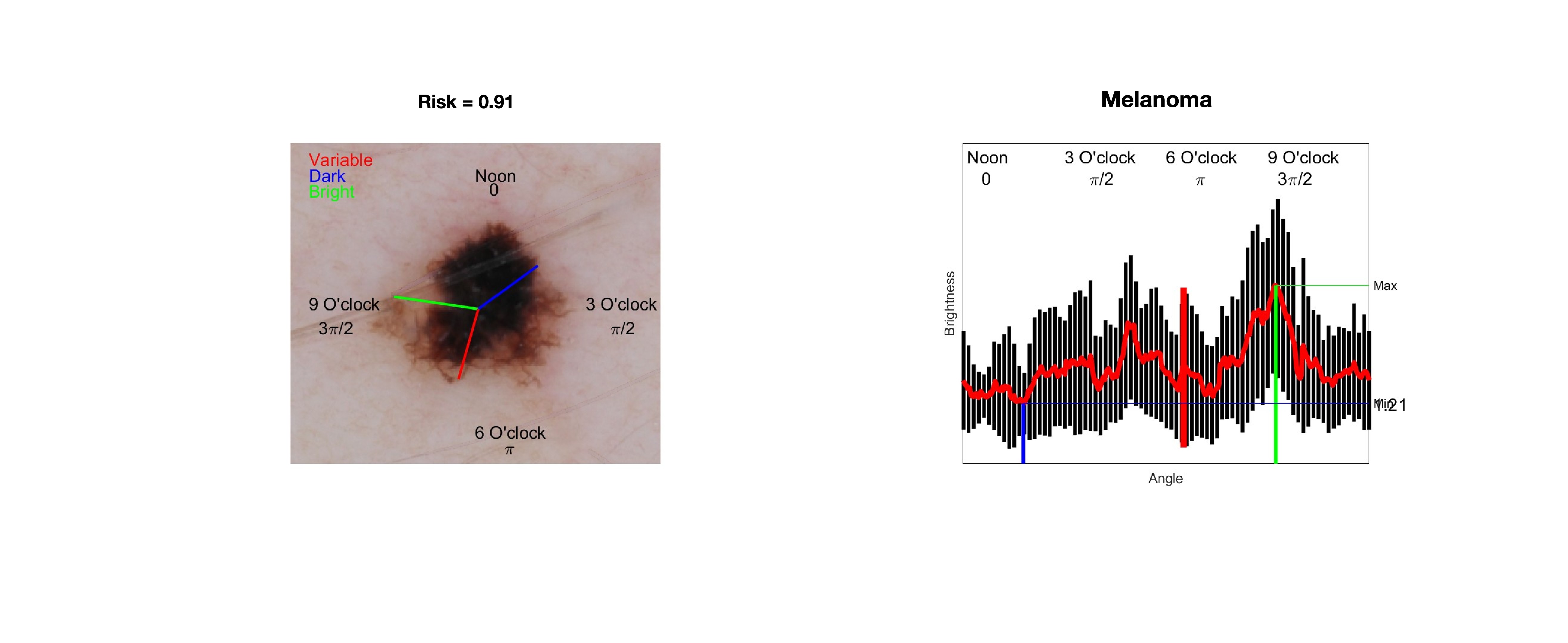

Supplement: Supplementary file 1 [file cancers-16-03077-s001.zip › cancers-3154863-supplementary/Supplementary File 2/056C.jpg]

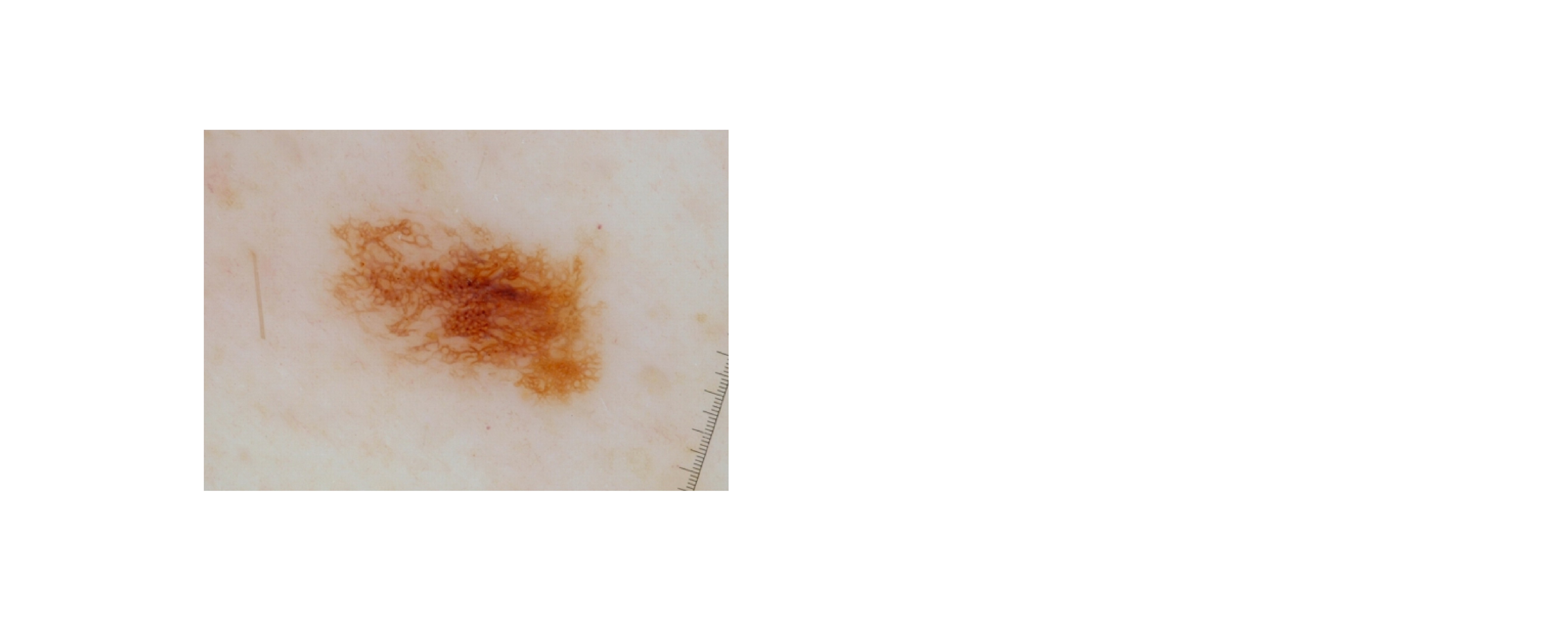

Supplement: Supplementary file 1 [file cancers-16-03077-s001.zip › cancers-3154863-supplementary/Supplementary File 2/057A.jpg]

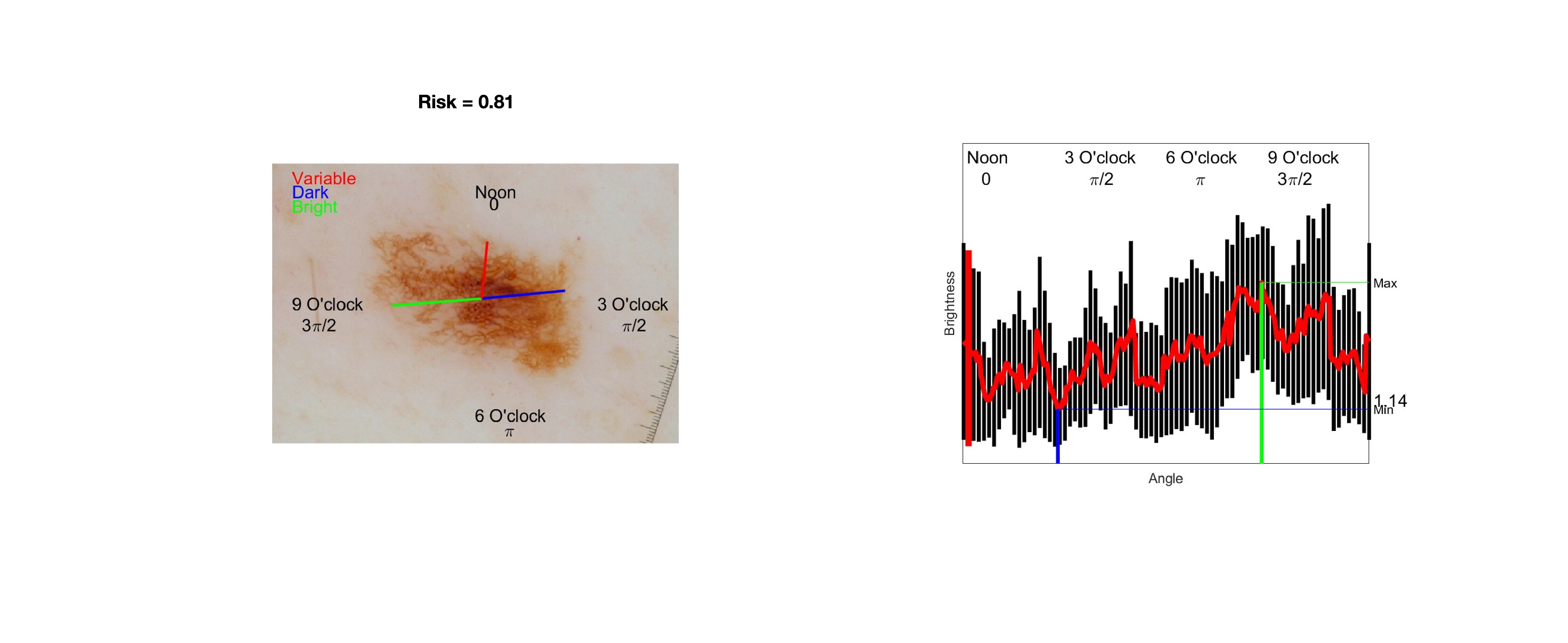

Supplement: Supplementary file 1 [file cancers-16-03077-s001.zip › cancers-3154863-supplementary/Supplementary File 2/057B.jpg]

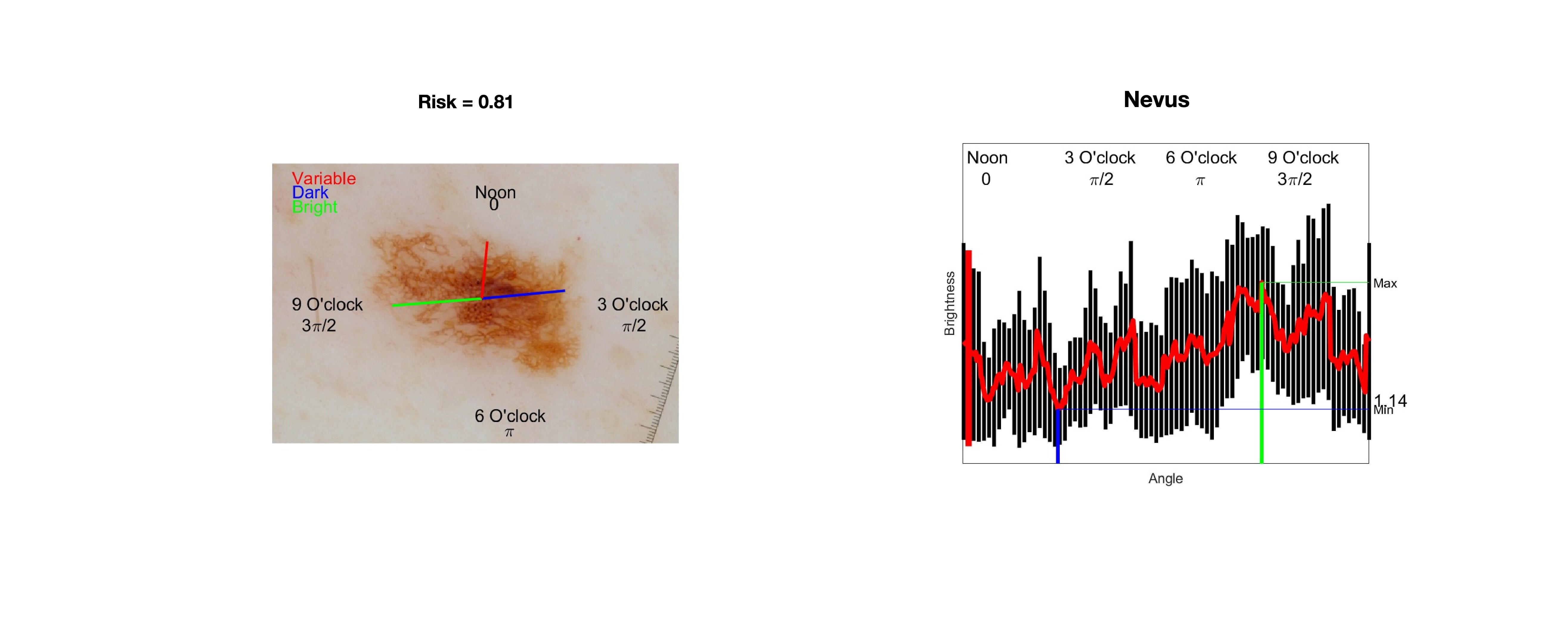

Supplement: Supplementary file 1 [file cancers-16-03077-s001.zip › cancers-3154863-supplementary/Supplementary File 2/057C.jpg]

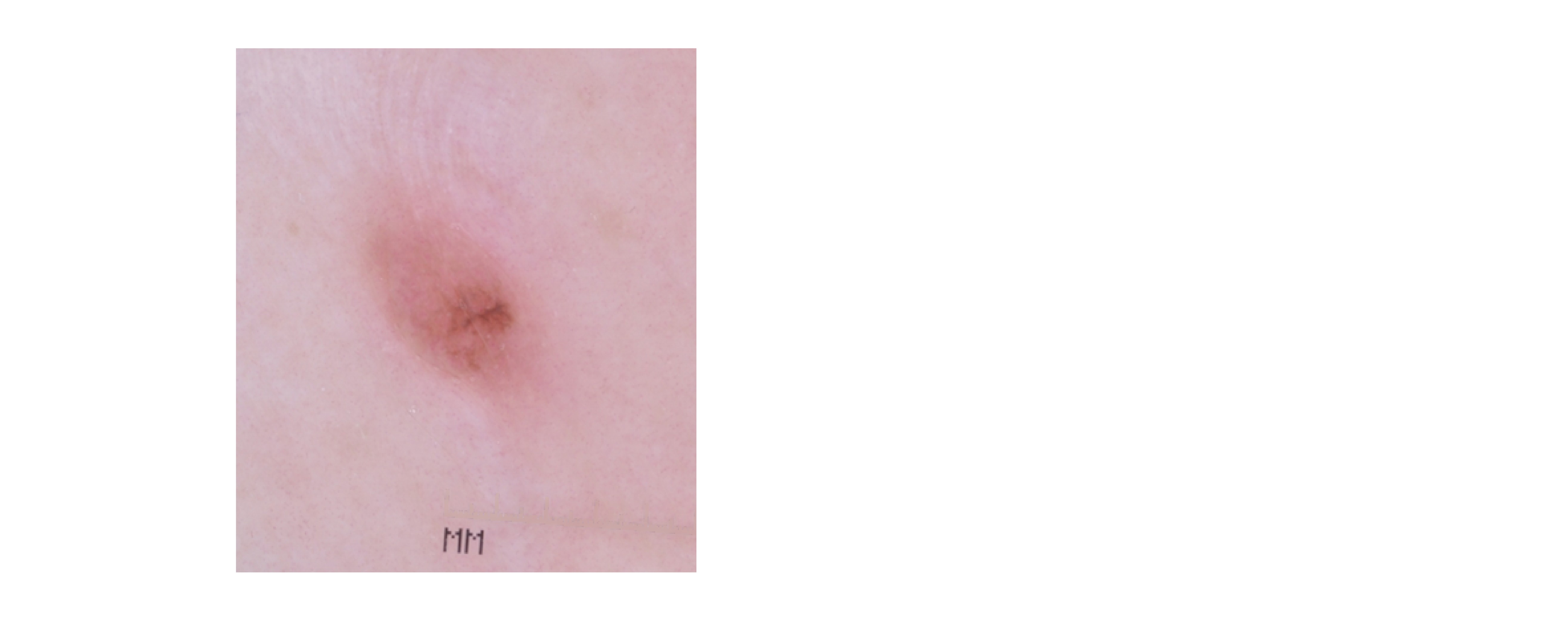

Supplement: Supplementary file 1 [file cancers-16-03077-s001.zip › cancers-3154863-supplementary/Supplementary File 2/058A.jpg]

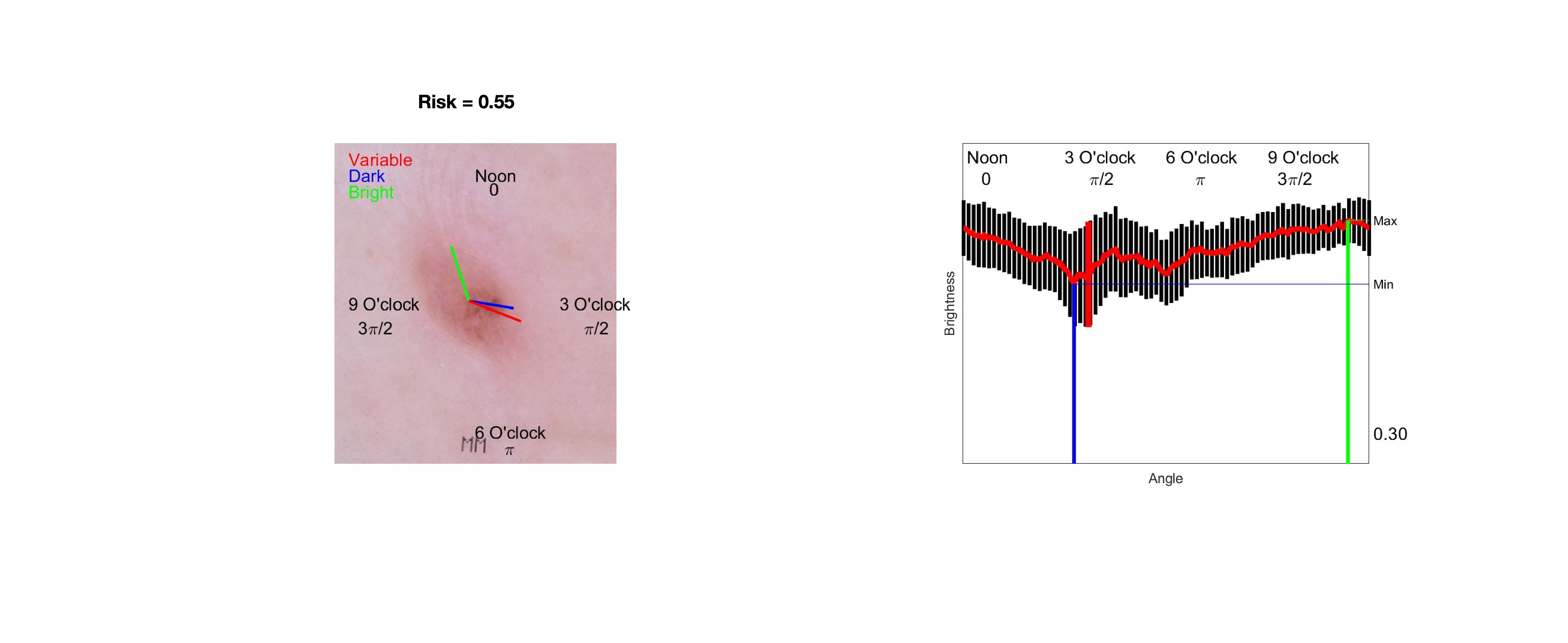

Supplement: Supplementary file 1 [file cancers-16-03077-s001.zip › cancers-3154863-supplementary/Supplementary File 2/058B.jpg]

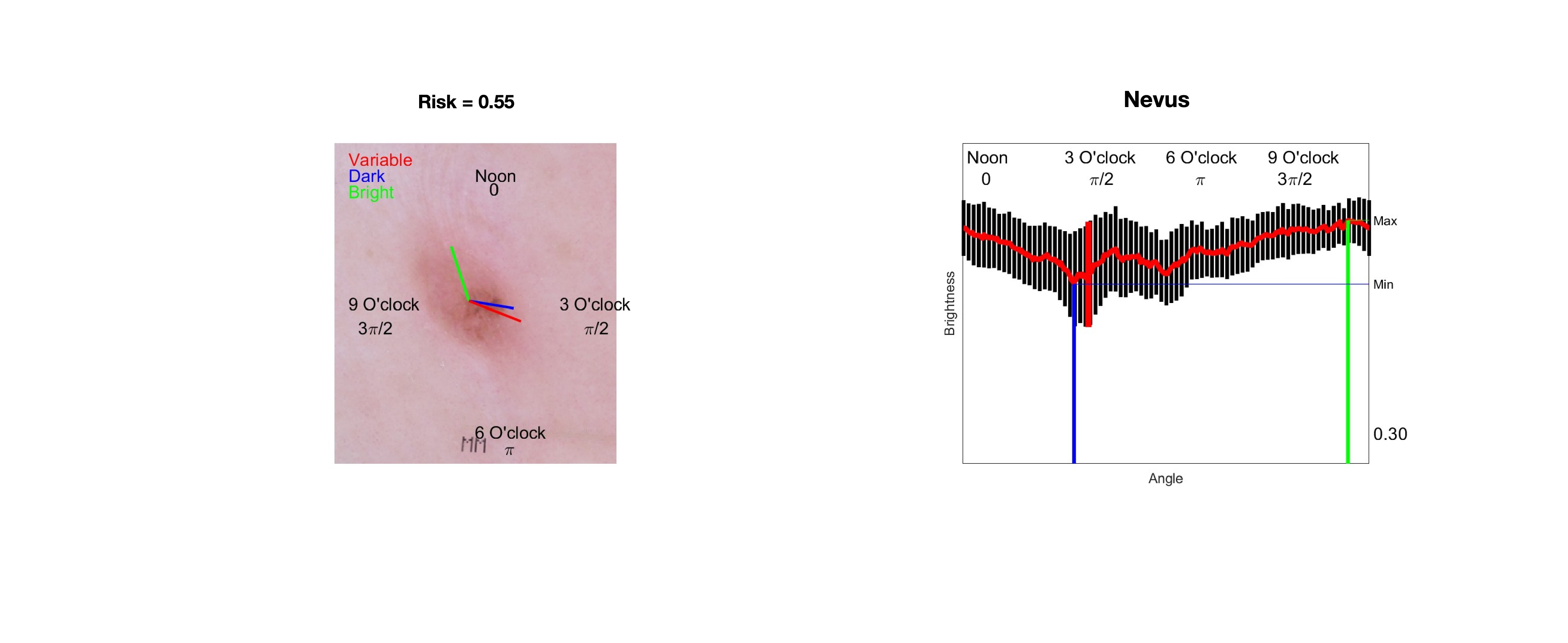

Supplement: Supplementary file 1 [file cancers-16-03077-s001.zip › cancers-3154863-supplementary/Supplementary File 2/058C.jpg]

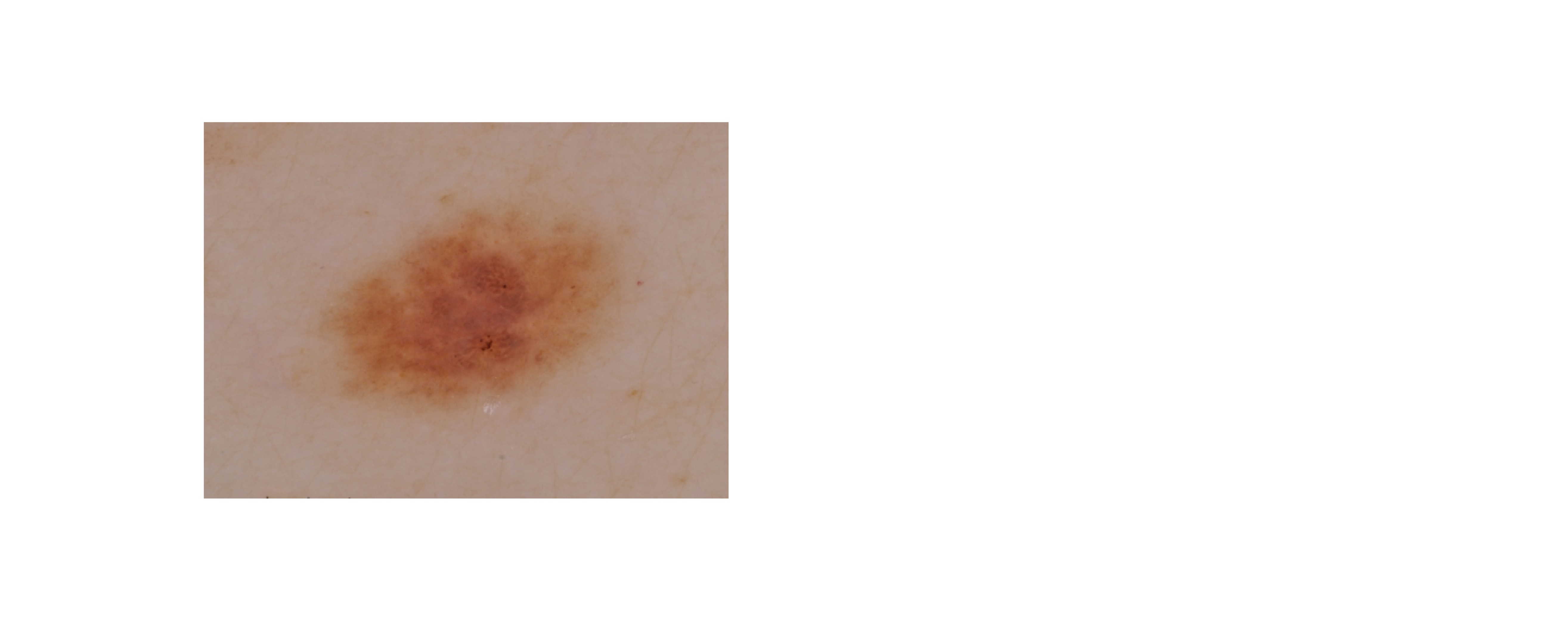

Supplement: Supplementary file 1 [file cancers-16-03077-s001.zip › cancers-3154863-supplementary/Supplementary File 2/059A.jpg]

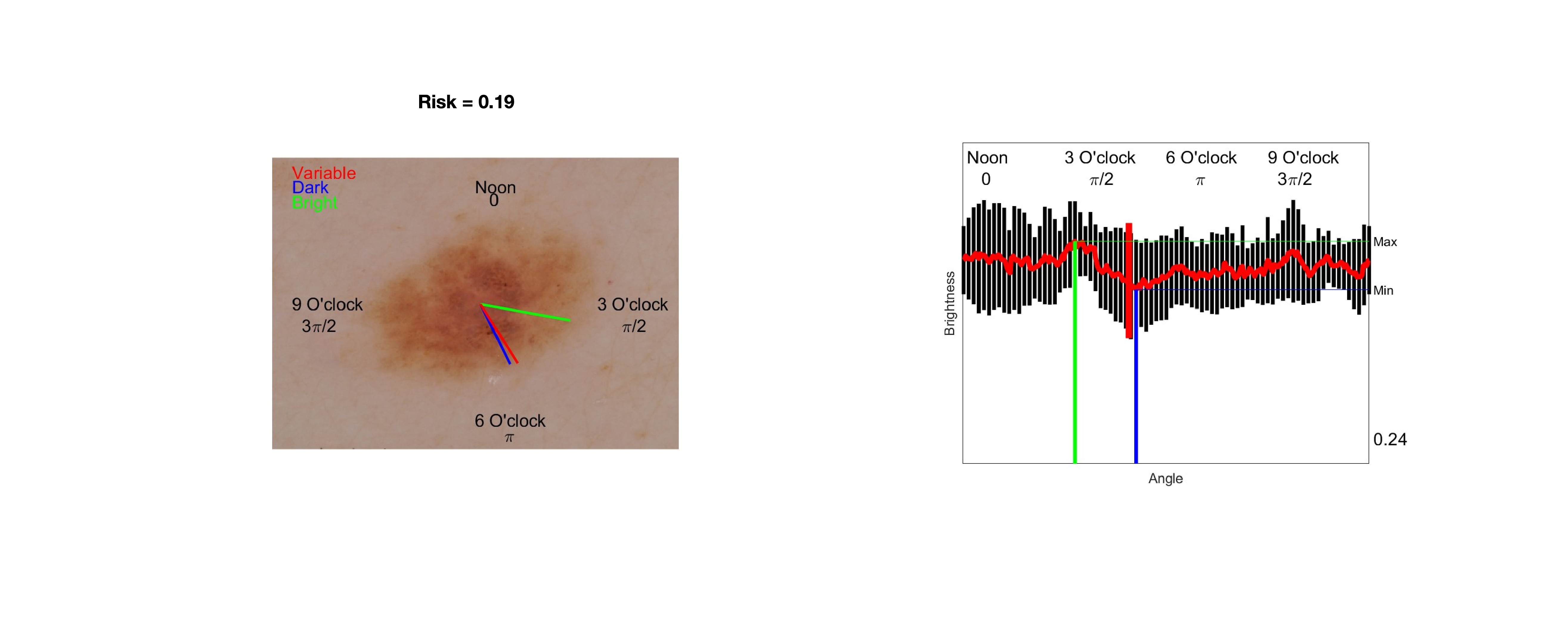

Supplement: Supplementary file 1 [file cancers-16-03077-s001.zip › cancers-3154863-supplementary/Supplementary File 2/059B.jpg]

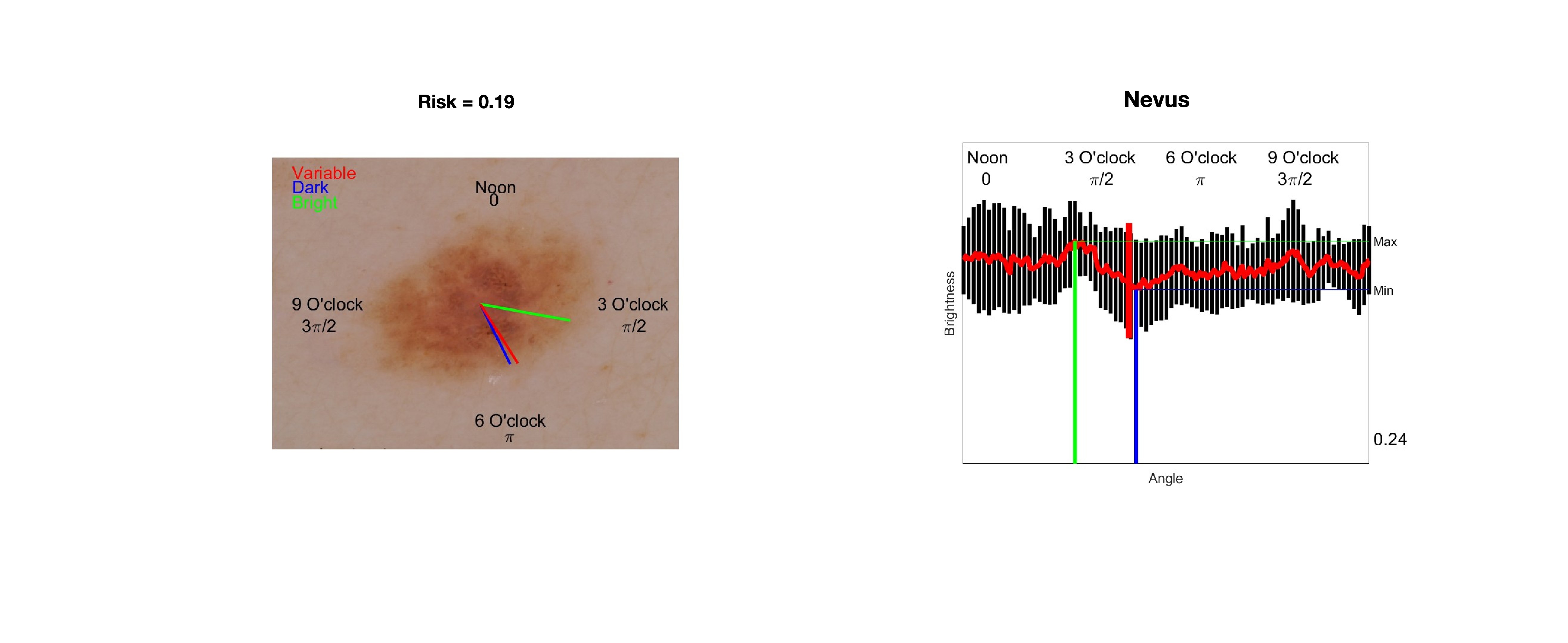

Supplement: Supplementary file 1 [file cancers-16-03077-s001.zip › cancers-3154863-supplementary/Supplementary File 2/059C.jpg]

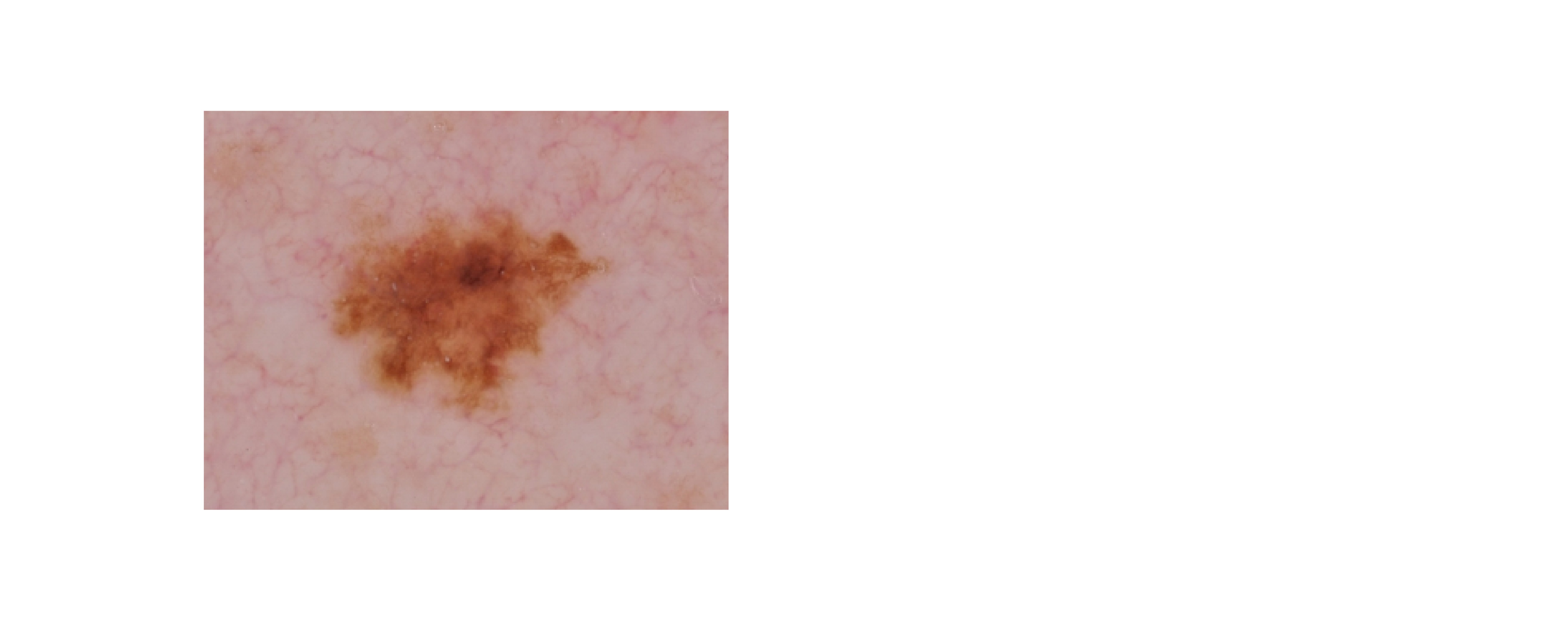

Supplement: Supplementary file 1 [file cancers-16-03077-s001.zip › cancers-3154863-supplementary/Supplementary File 2/060A.jpg]

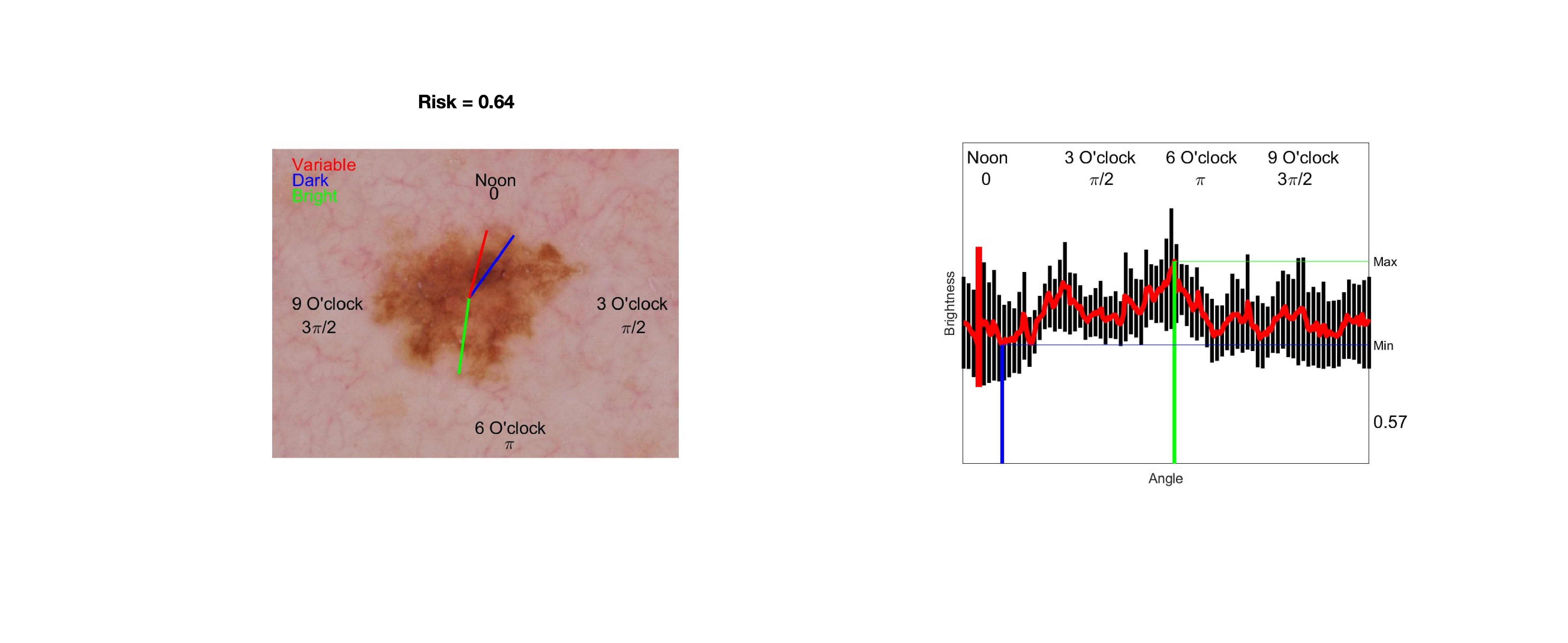

Supplement: Supplementary file 1 [file cancers-16-03077-s001.zip › cancers-3154863-supplementary/Supplementary File 2/060B.jpg]

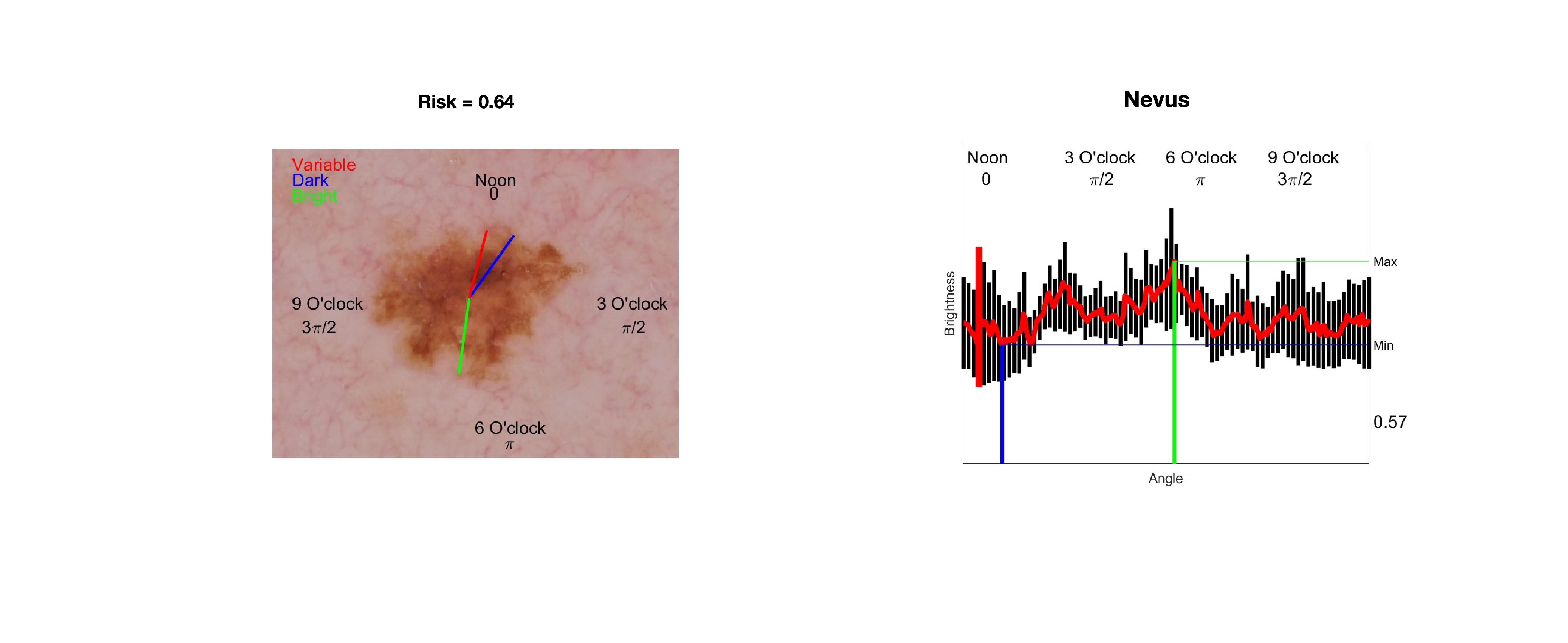

Supplement: Supplementary file 1 [file cancers-16-03077-s001.zip › cancers-3154863-supplementary/Supplementary File 2/060C.jpg]

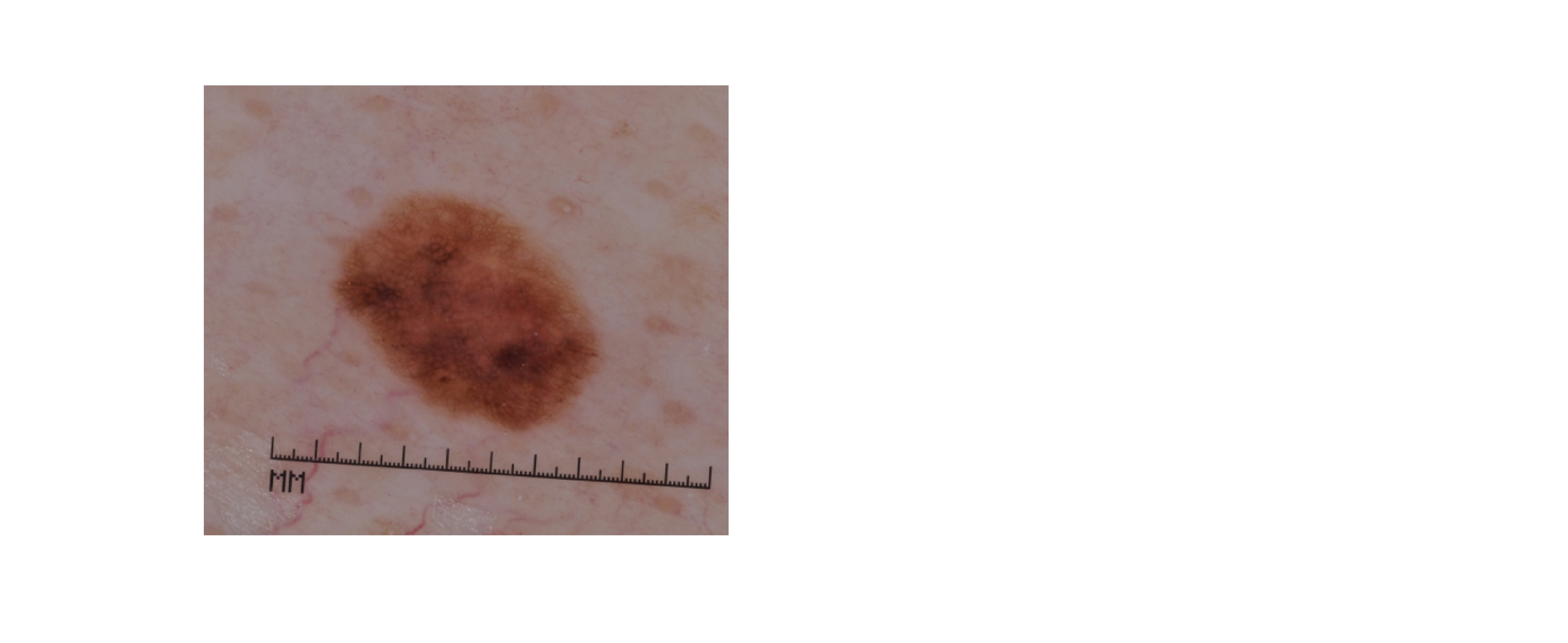

Supplement: Supplementary file 1 [file cancers-16-03077-s001.zip › cancers-3154863-supplementary/Supplementary File 2/061A.jpg]

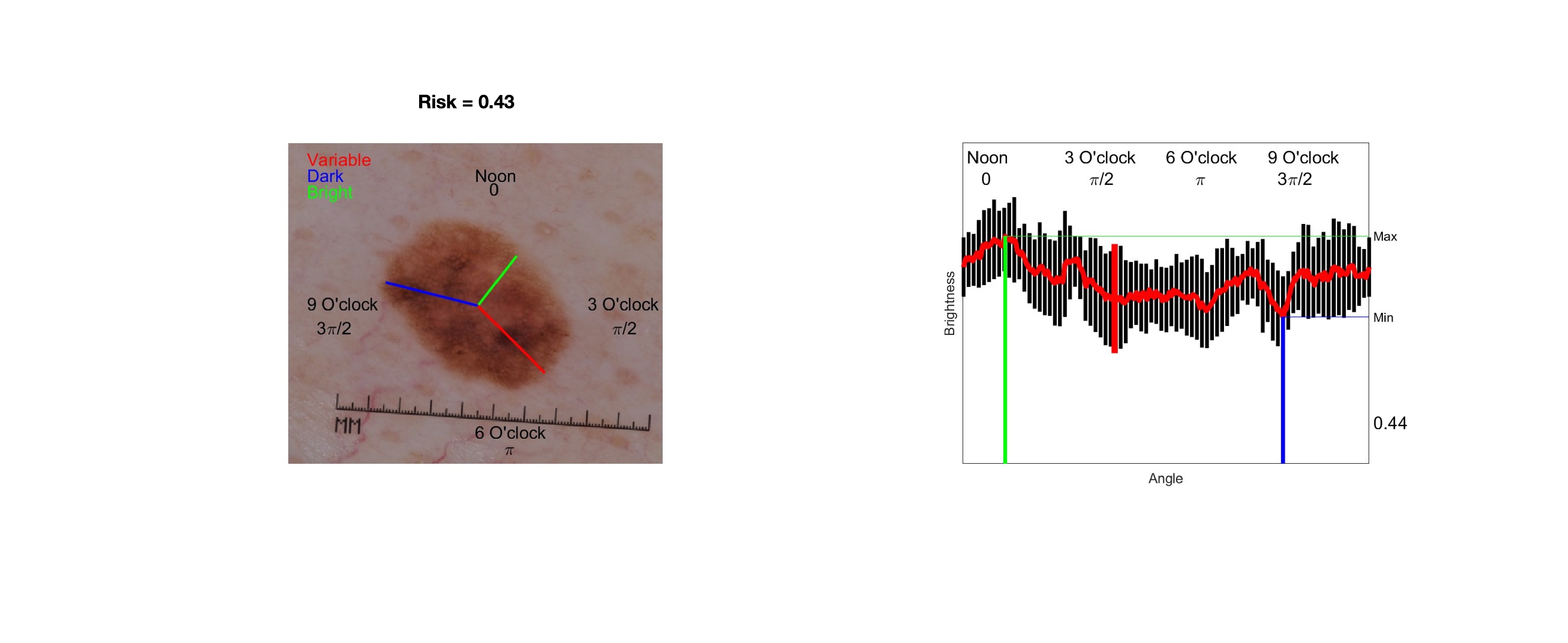

Supplement: Supplementary file 1 [file cancers-16-03077-s001.zip › cancers-3154863-supplementary/Supplementary File 2/061B.jpg]

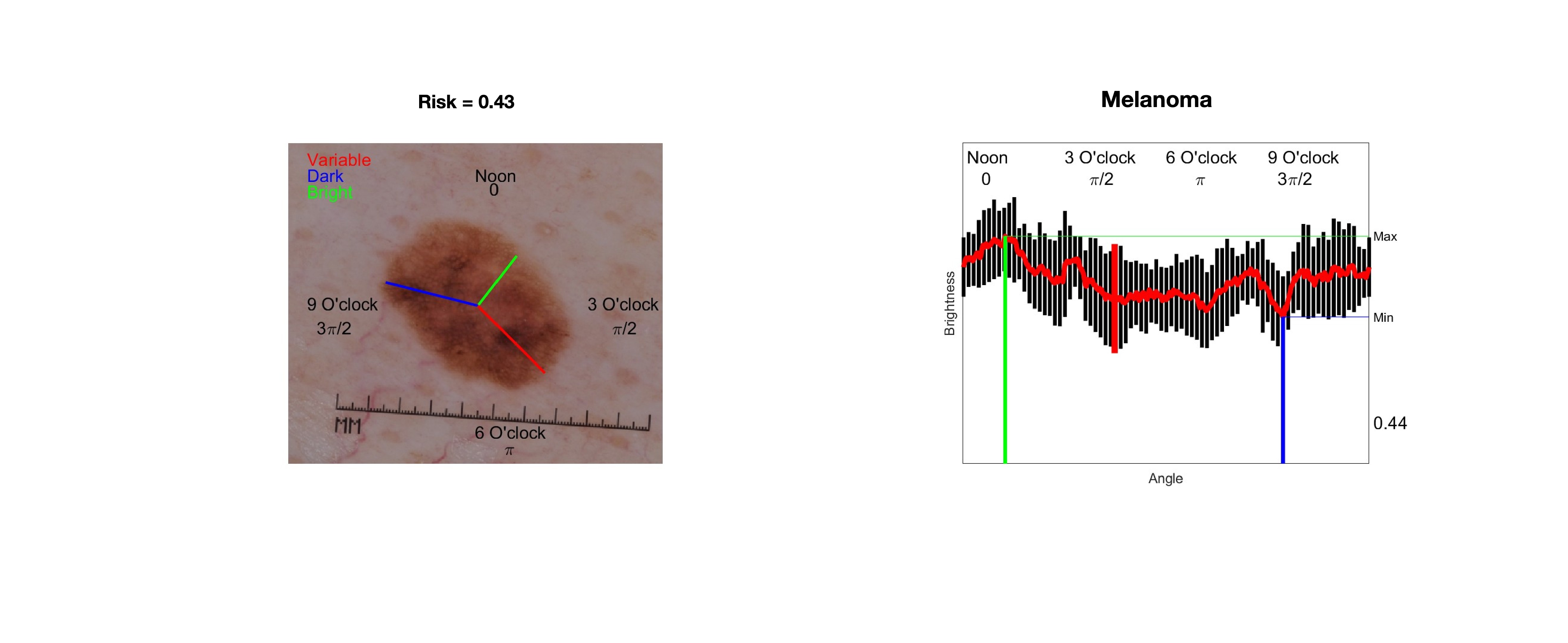

Supplement: Supplementary file 1 [file cancers-16-03077-s001.zip › cancers-3154863-supplementary/Supplementary File 2/061C.jpg]

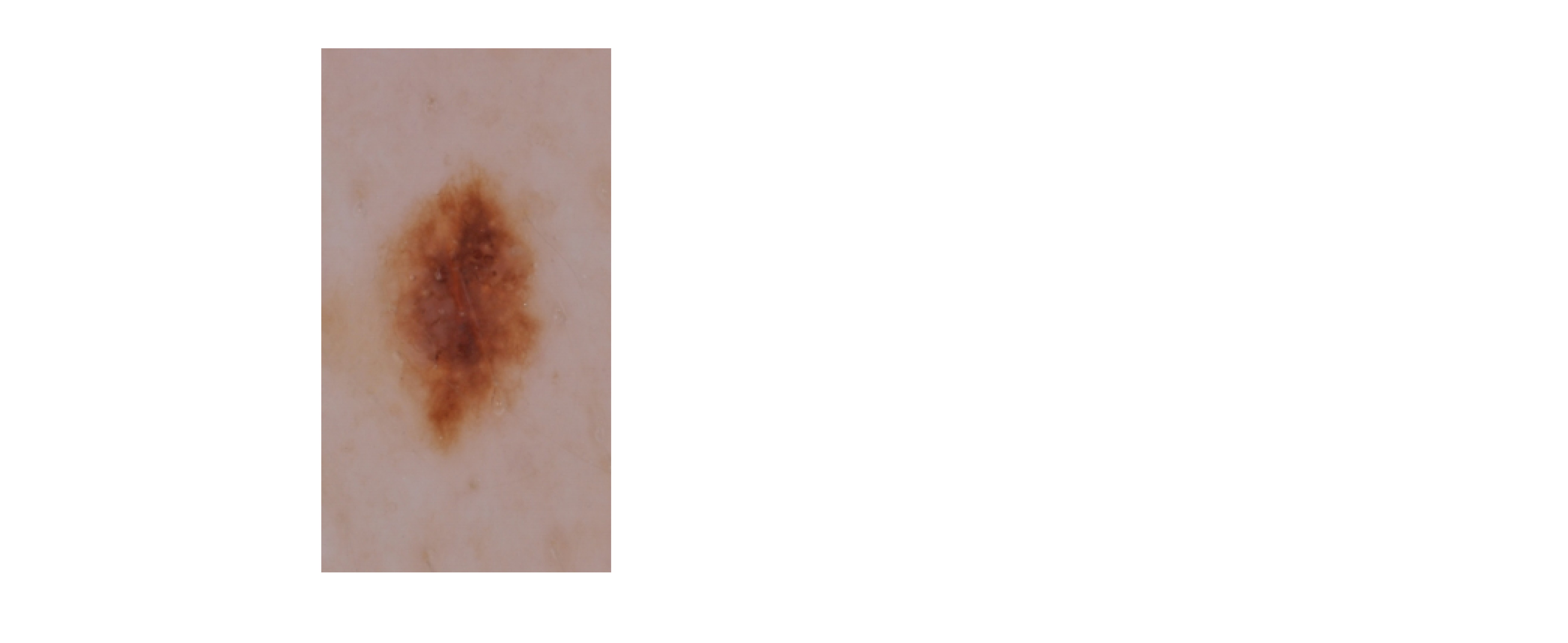

Supplement: Supplementary file 1 [file cancers-16-03077-s001.zip › cancers-3154863-supplementary/Supplementary File 2/062A.jpg]

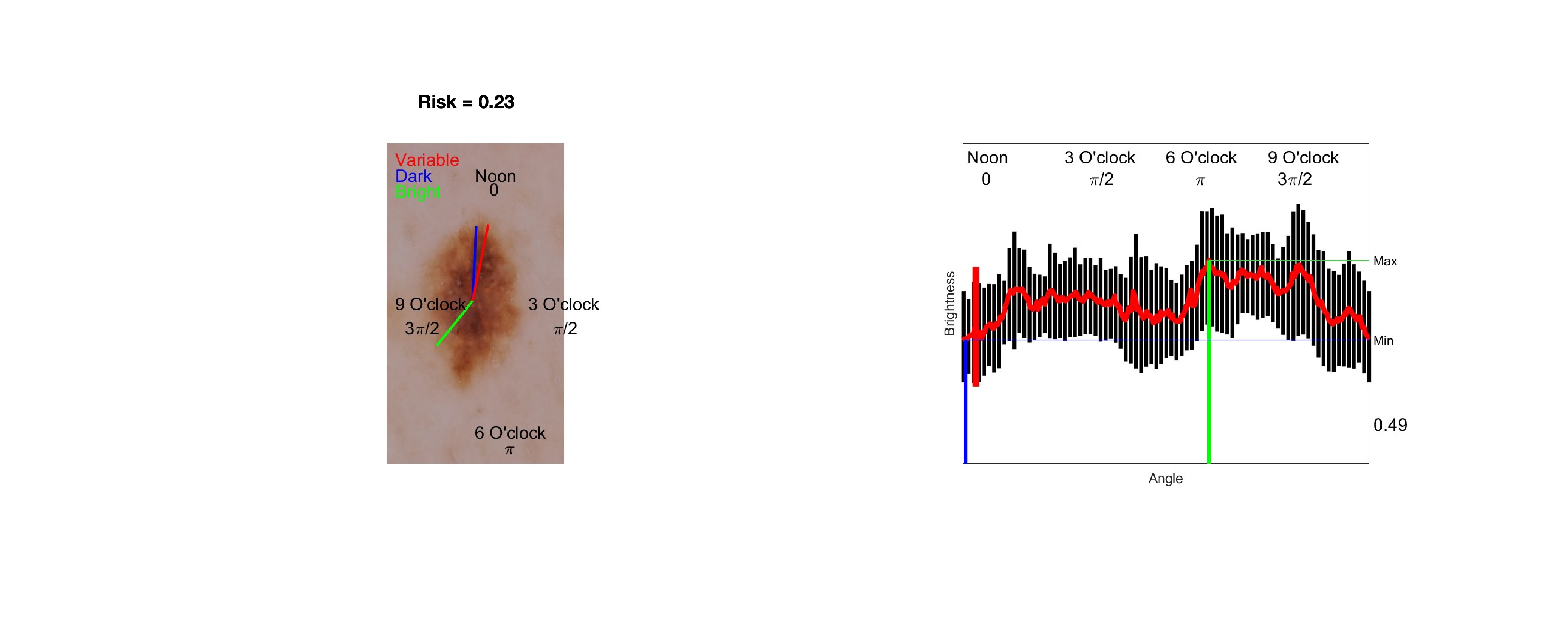

Supplement: Supplementary file 1 [file cancers-16-03077-s001.zip › cancers-3154863-supplementary/Supplementary File 2/062B.jpg]

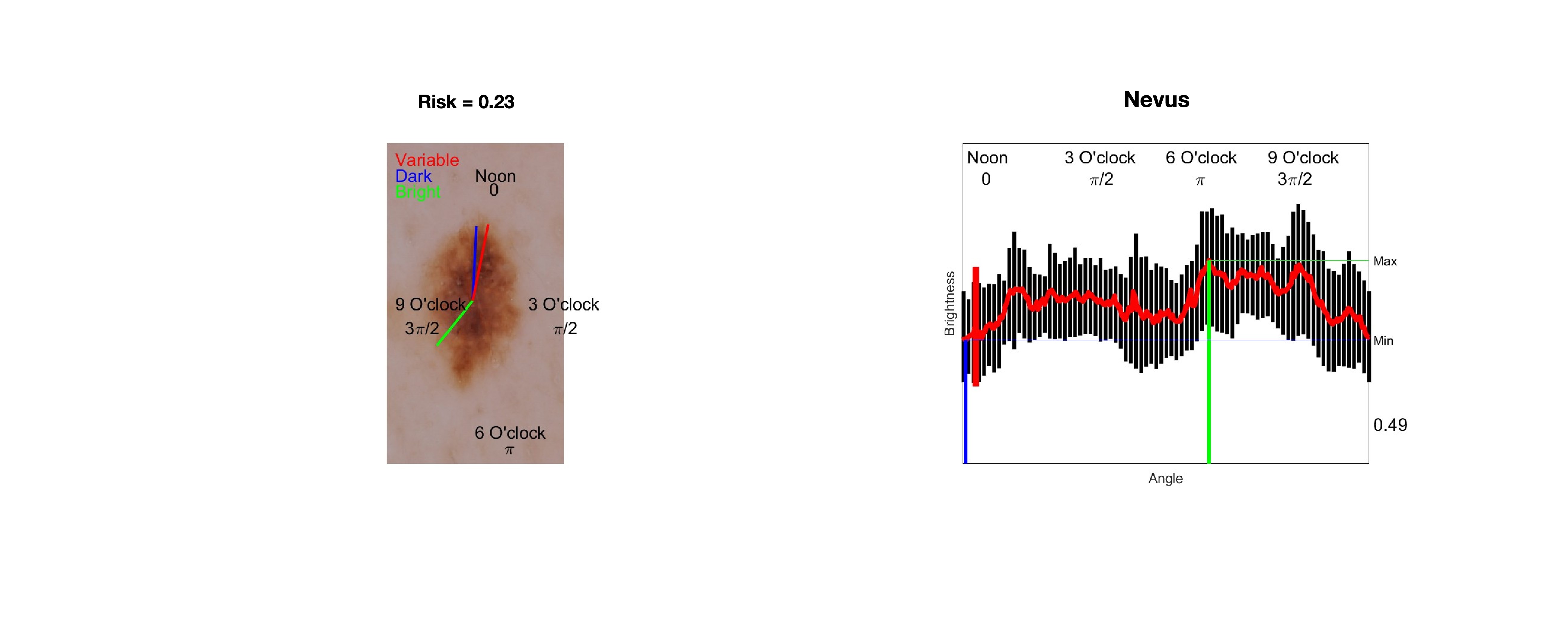

Supplement: Supplementary file 1 [file cancers-16-03077-s001.zip › cancers-3154863-supplementary/Supplementary File 2/062C.jpg]

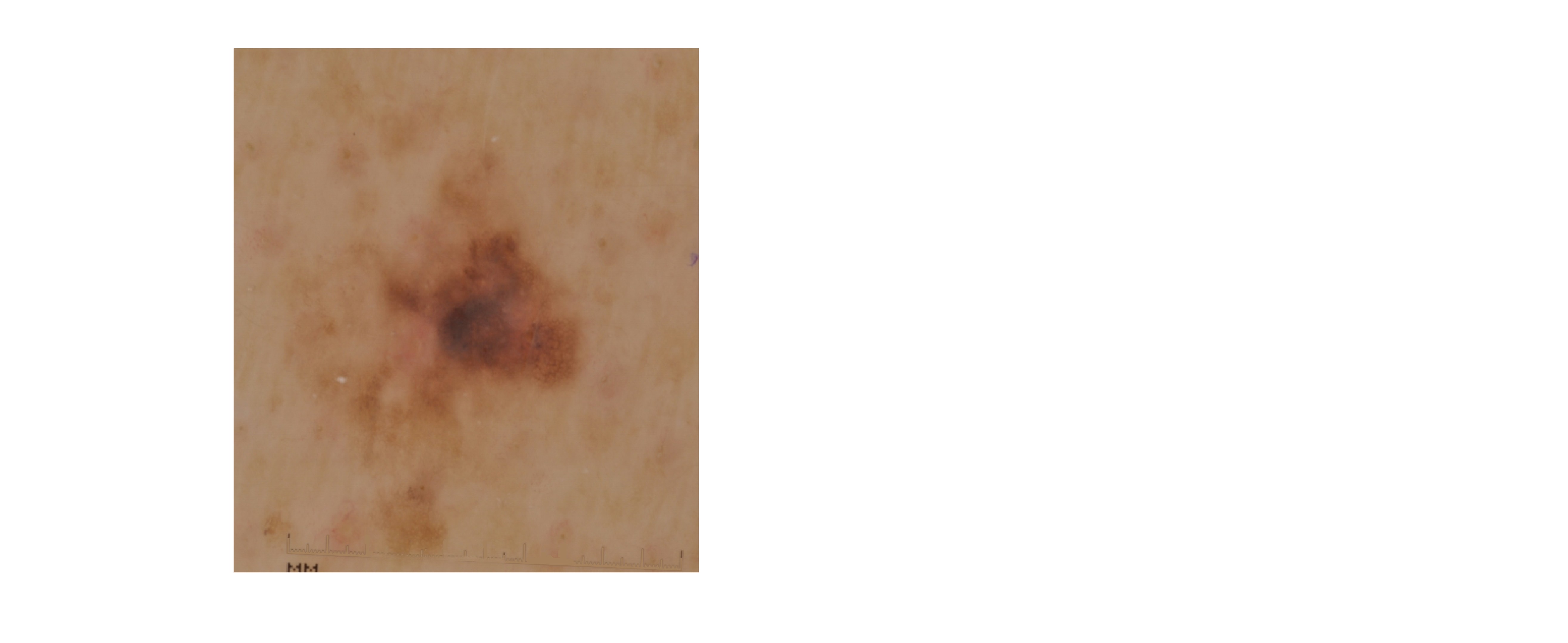

Supplement: Supplementary file 1 [file cancers-16-03077-s001.zip › cancers-3154863-supplementary/Supplementary File 2/063A.jpg]

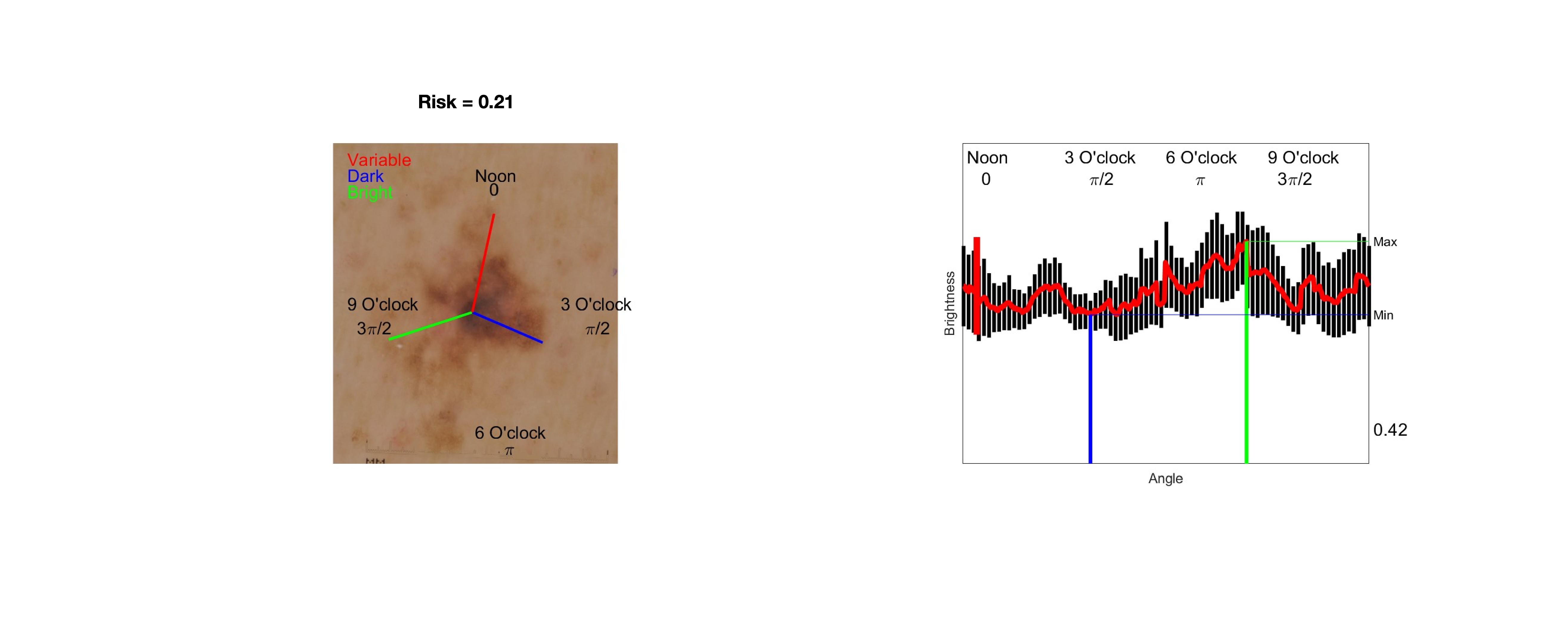

Supplement: Supplementary file 1 [file cancers-16-03077-s001.zip › cancers-3154863-supplementary/Supplementary File 2/063B.jpg]

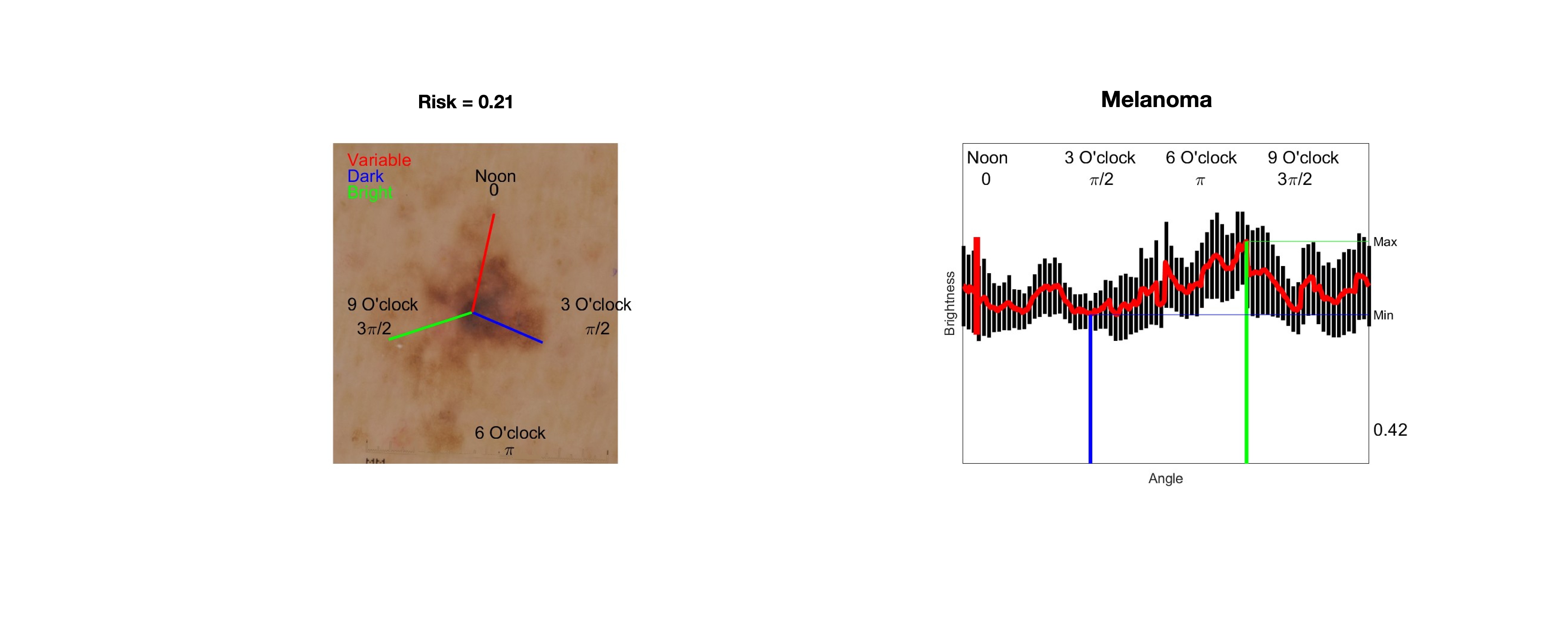

Supplement: Supplementary file 1 [file cancers-16-03077-s001.zip › cancers-3154863-supplementary/Supplementary File 2/063C.jpg]

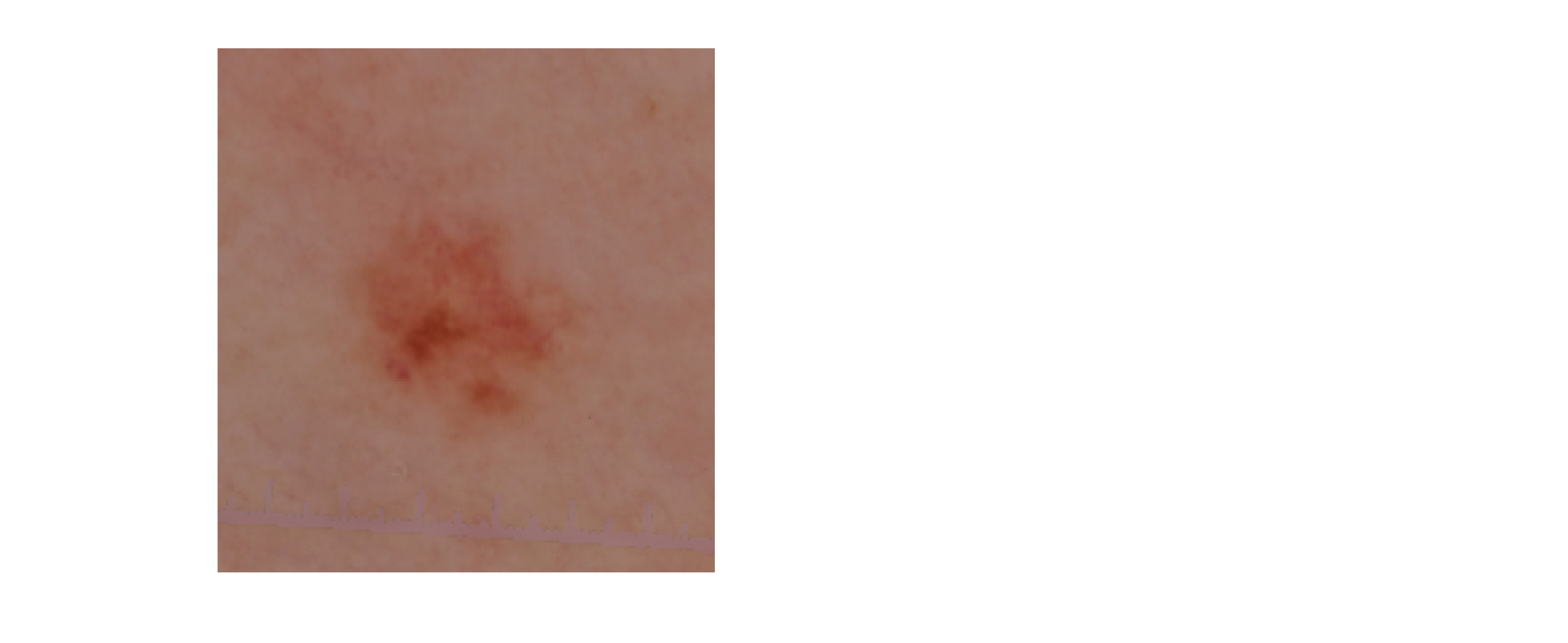

Supplement: Supplementary file 1 [file cancers-16-03077-s001.zip › cancers-3154863-supplementary/Supplementary File 2/064A.jpg]

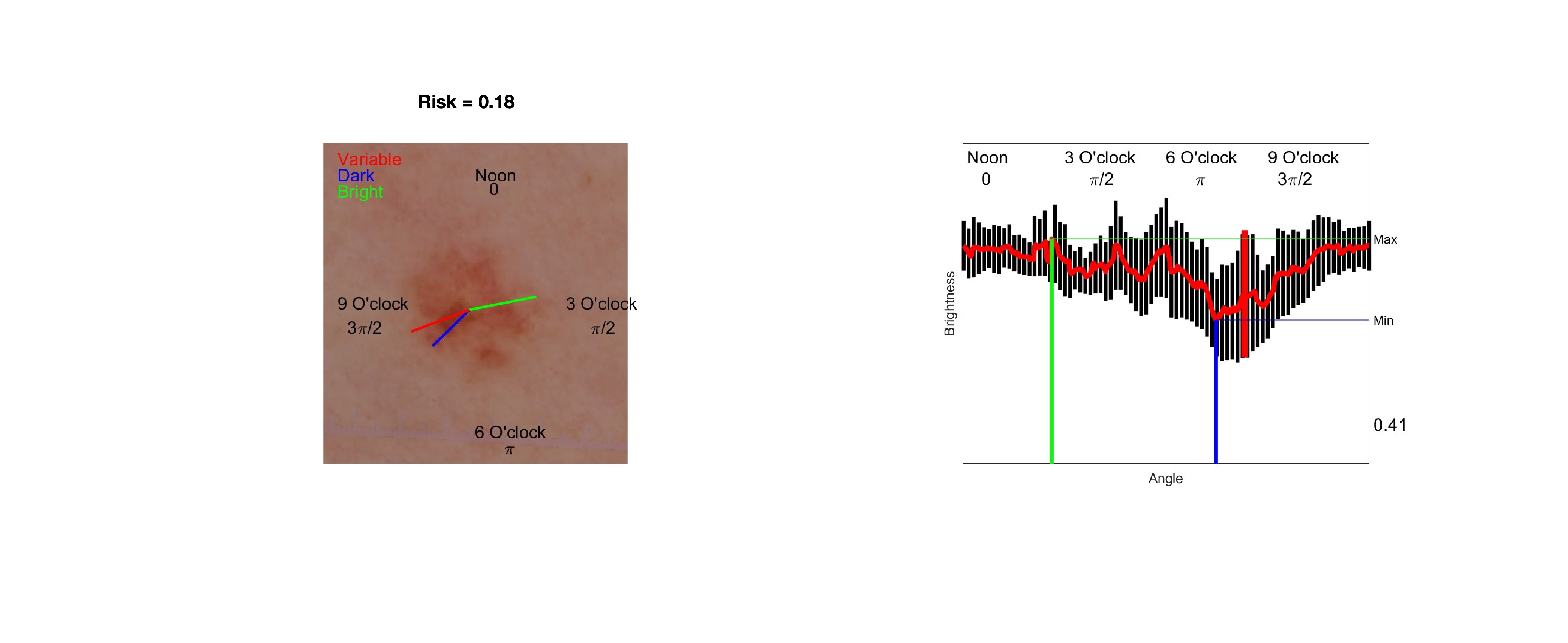

Supplement: Supplementary file 1 [file cancers-16-03077-s001.zip › cancers-3154863-supplementary/Supplementary File 2/064B.jpg]

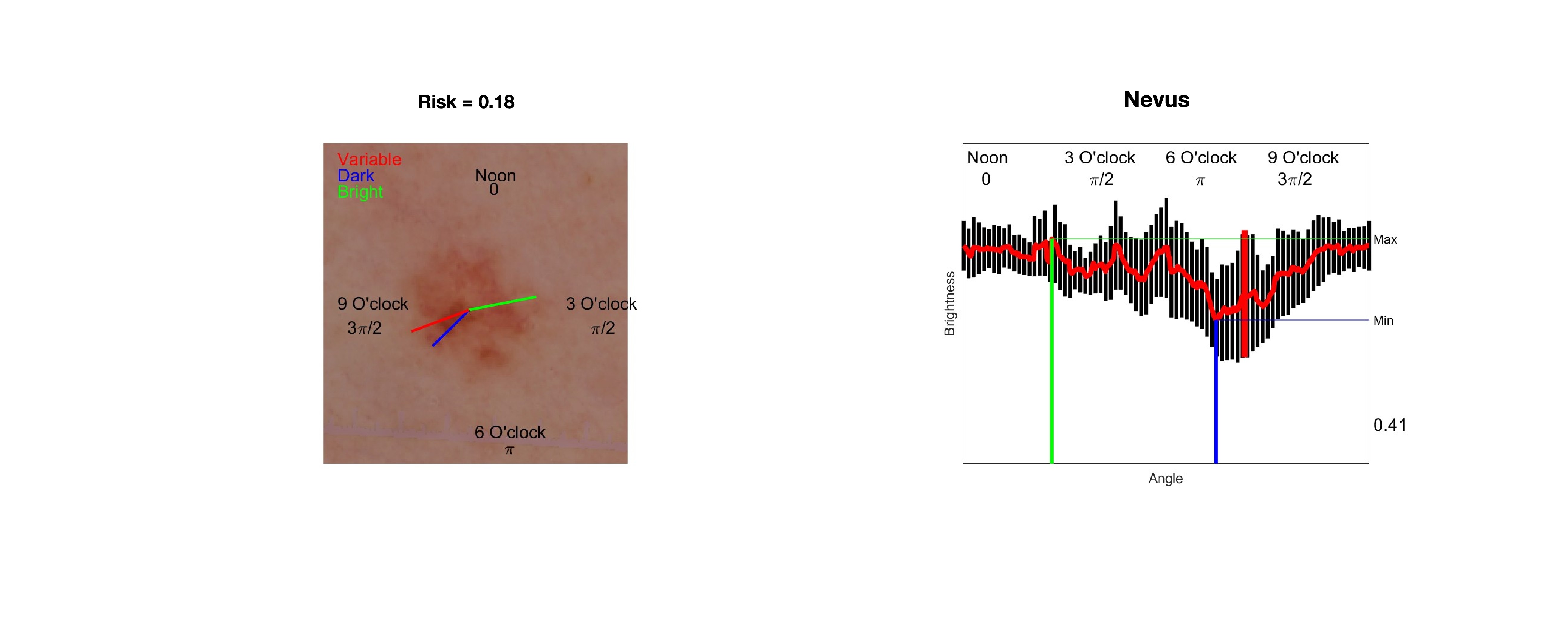

Supplement: Supplementary file 1 [file cancers-16-03077-s001.zip › cancers-3154863-supplementary/Supplementary File 2/064C.jpg]

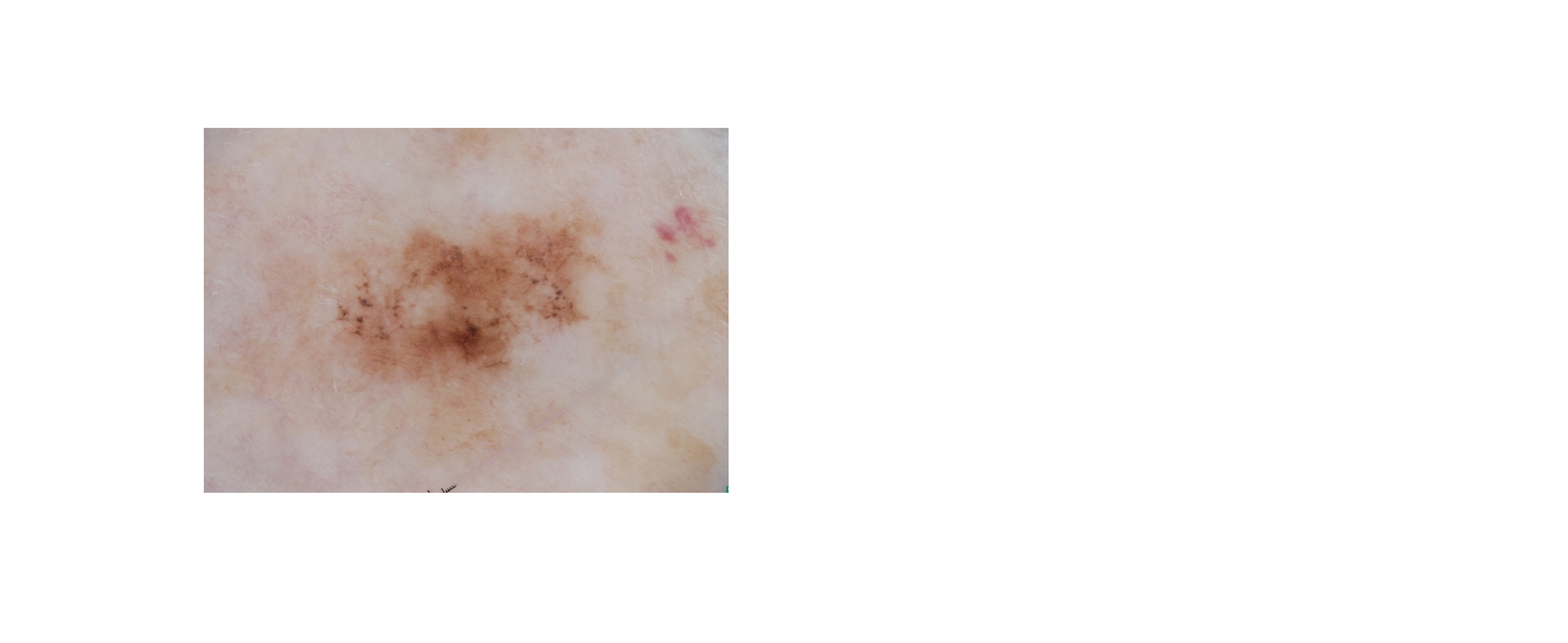

Supplement: Supplementary file 1 [file cancers-16-03077-s001.zip › cancers-3154863-supplementary/Supplementary File 2/065A.jpg]

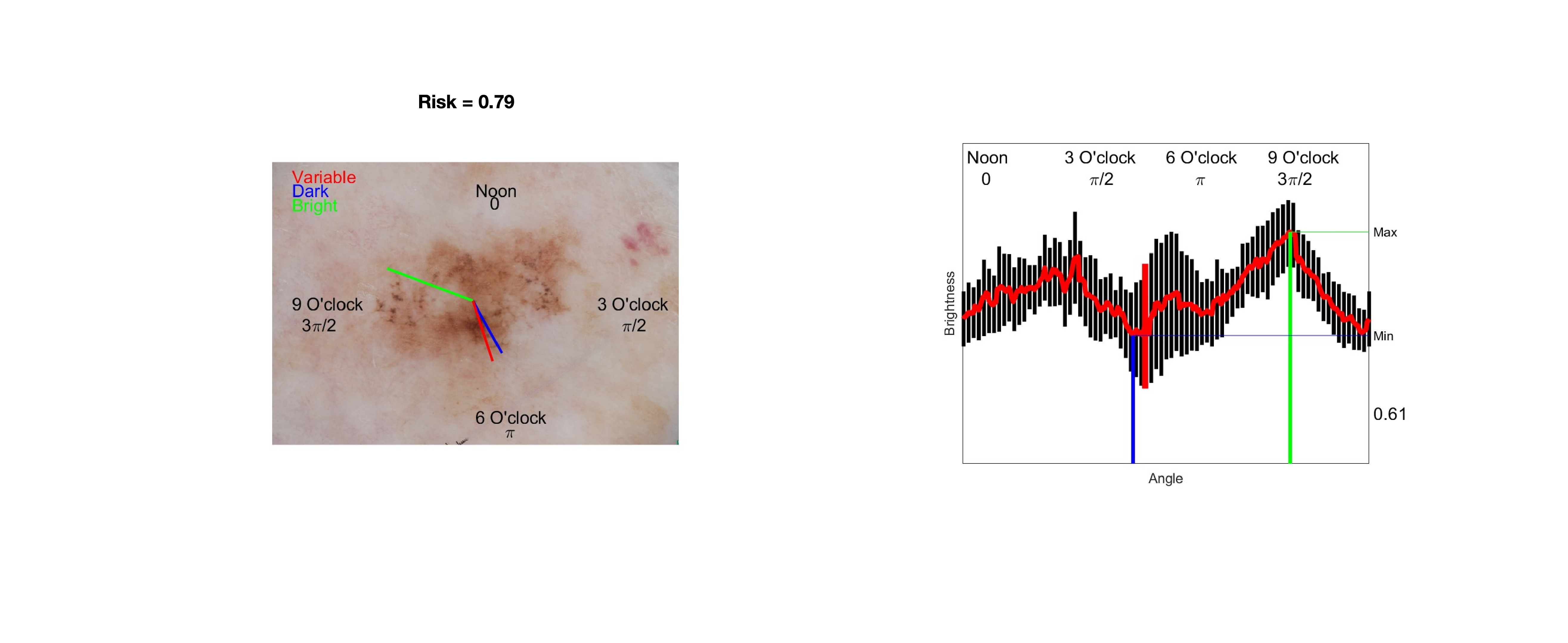

Supplement: Supplementary file 1 [file cancers-16-03077-s001.zip › cancers-3154863-supplementary/Supplementary File 2/065B.jpg]

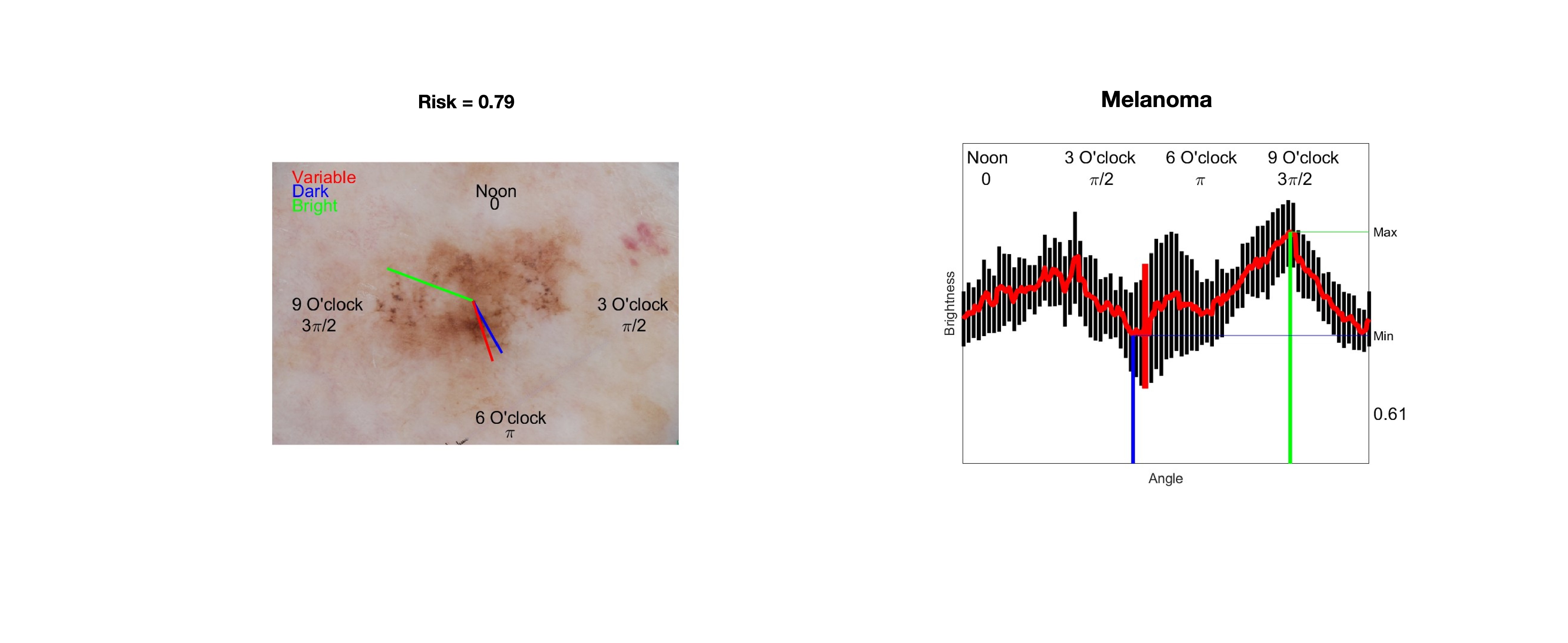

Supplement: Supplementary file 1 [file cancers-16-03077-s001.zip › cancers-3154863-supplementary/Supplementary File 2/065C.jpg]

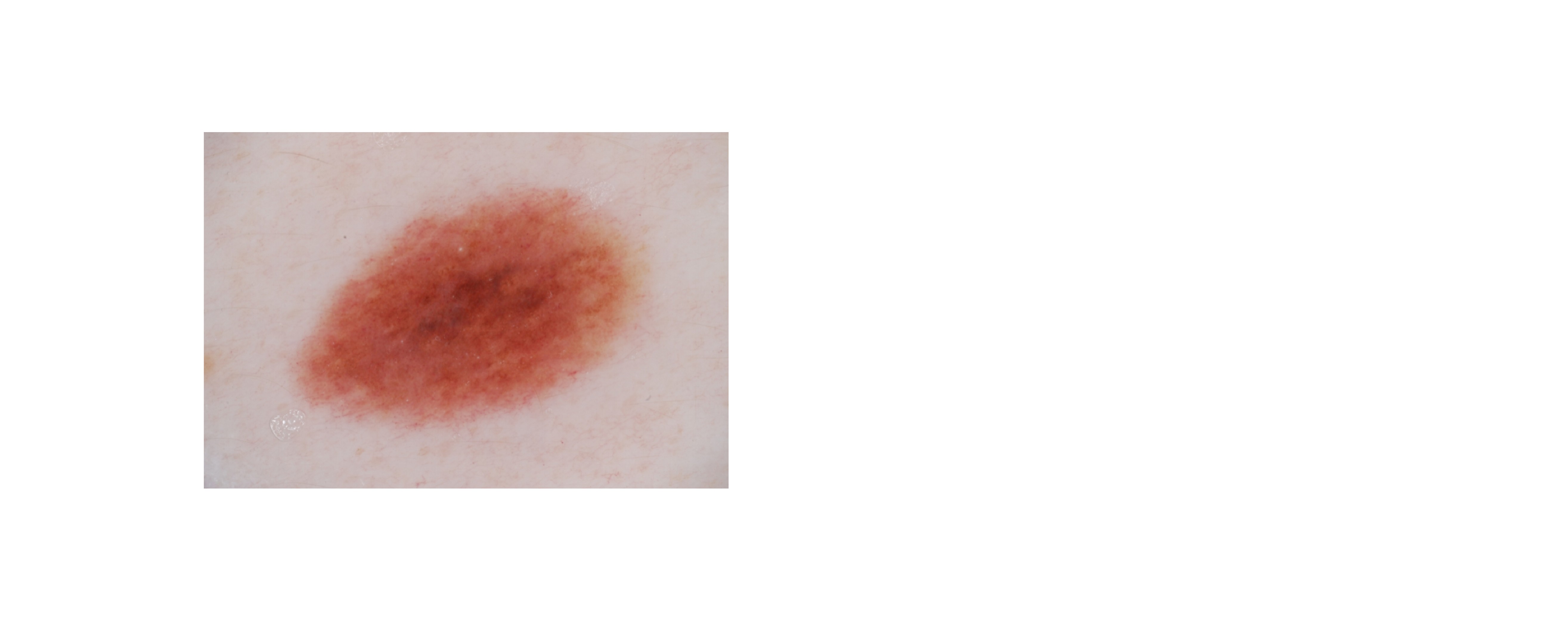

Supplement: Supplementary file 1 [file cancers-16-03077-s001.zip › cancers-3154863-supplementary/Supplementary File 2/066A.jpg]

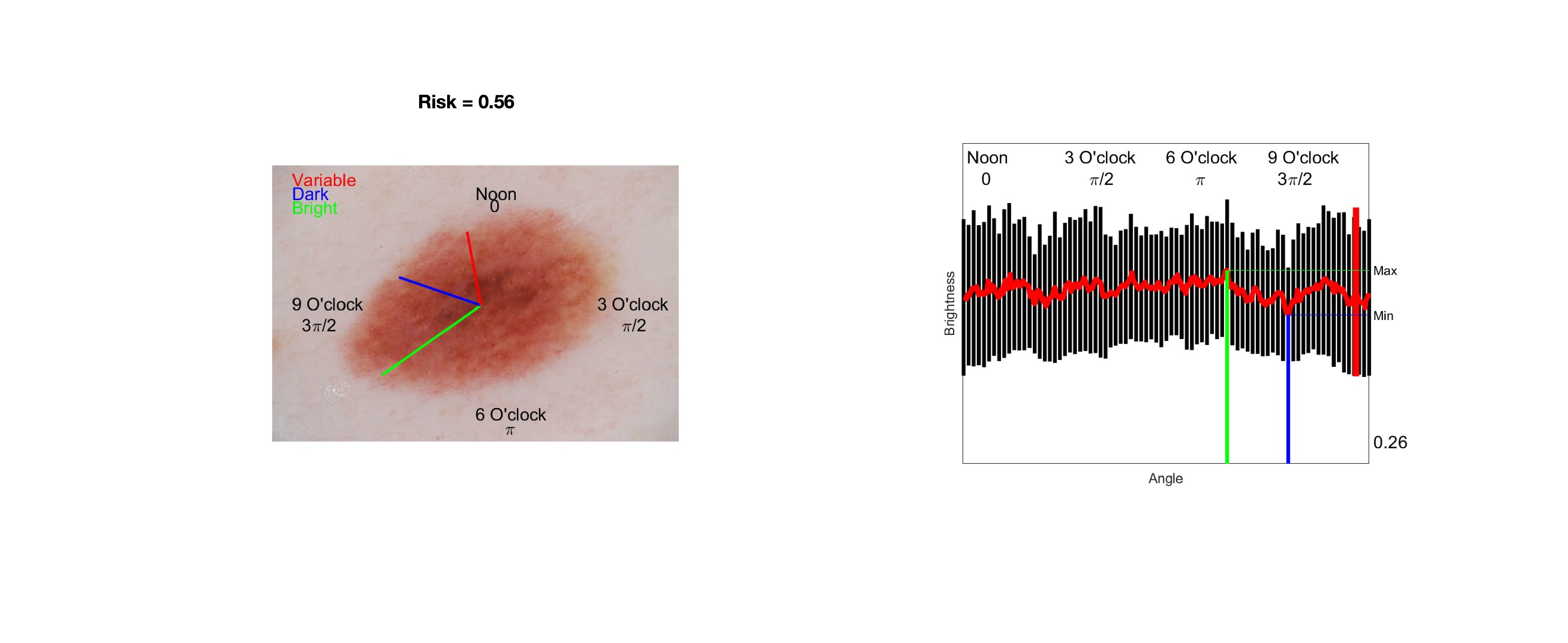

Supplement: Supplementary file 1 [file cancers-16-03077-s001.zip › cancers-3154863-supplementary/Supplementary File 2/066B.jpg]

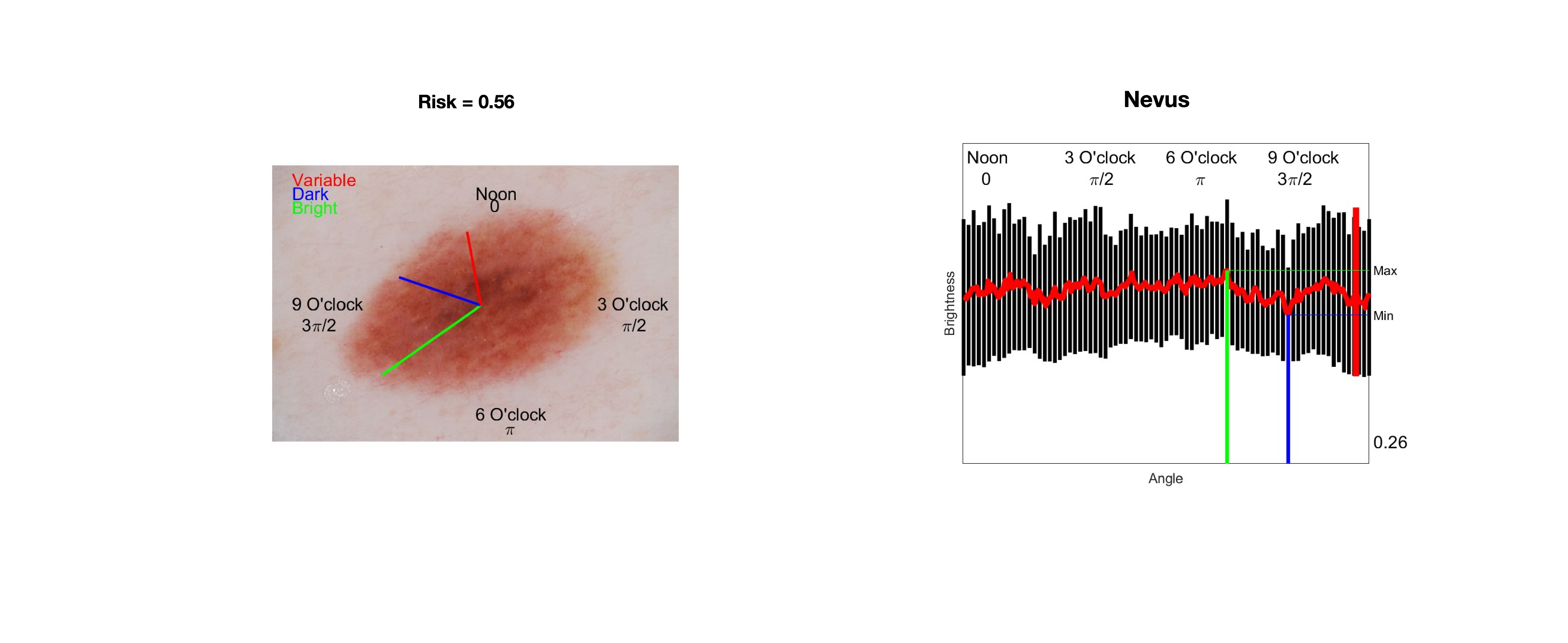

Supplement: Supplementary file 1 [file cancers-16-03077-s001.zip › cancers-3154863-supplementary/Supplementary File 2/066C.jpg]

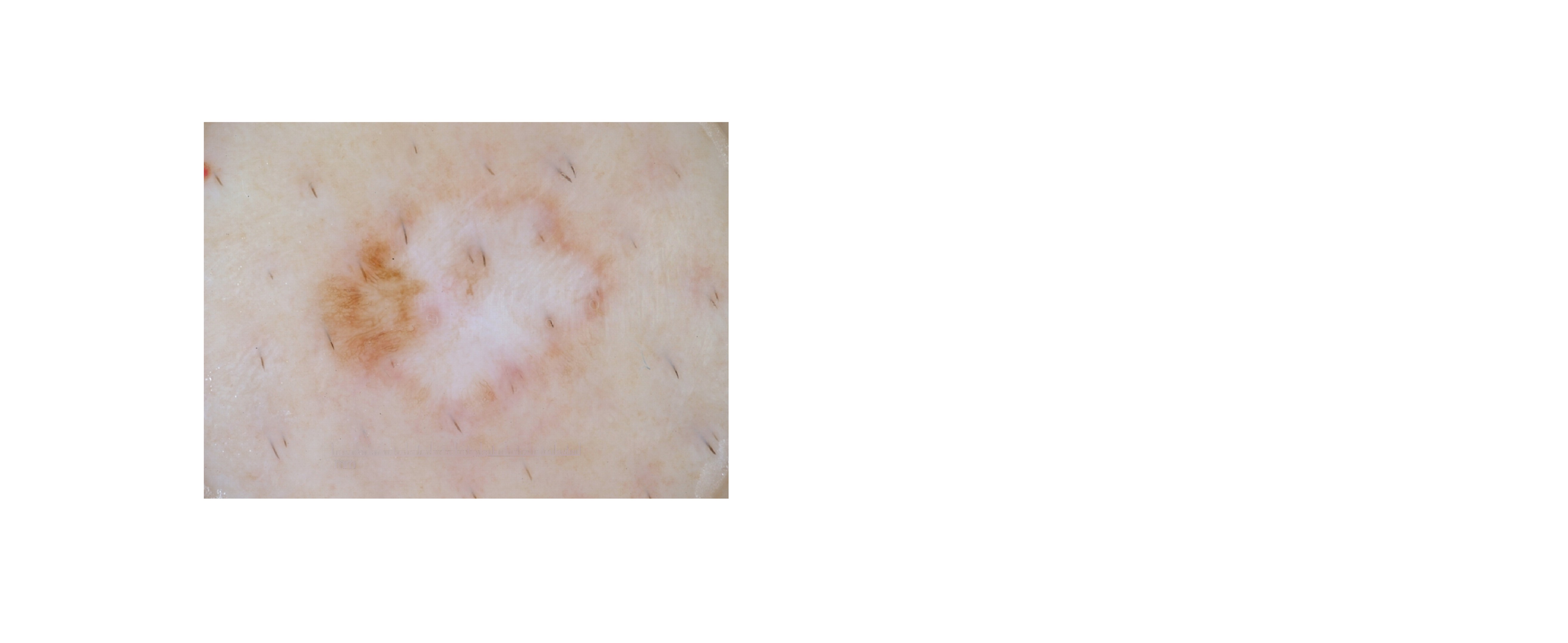

Supplement: Supplementary file 1 [file cancers-16-03077-s001.zip › cancers-3154863-supplementary/Supplementary File 2/067A.jpg]
