# Supplementary figures and images for: The Impact of Melanoma Imaging Biomarker Cues on Detection Sensitivity and Specificity in Melanoma versus Clinically Atypical Nevi (part 1 of 2)
Source: Cancers (Basel). 2024 Sep 4;16(17):3077. doi: 10.3390/cancers16173077 (PMC11394255; doi:10.3390/cancers16173077)

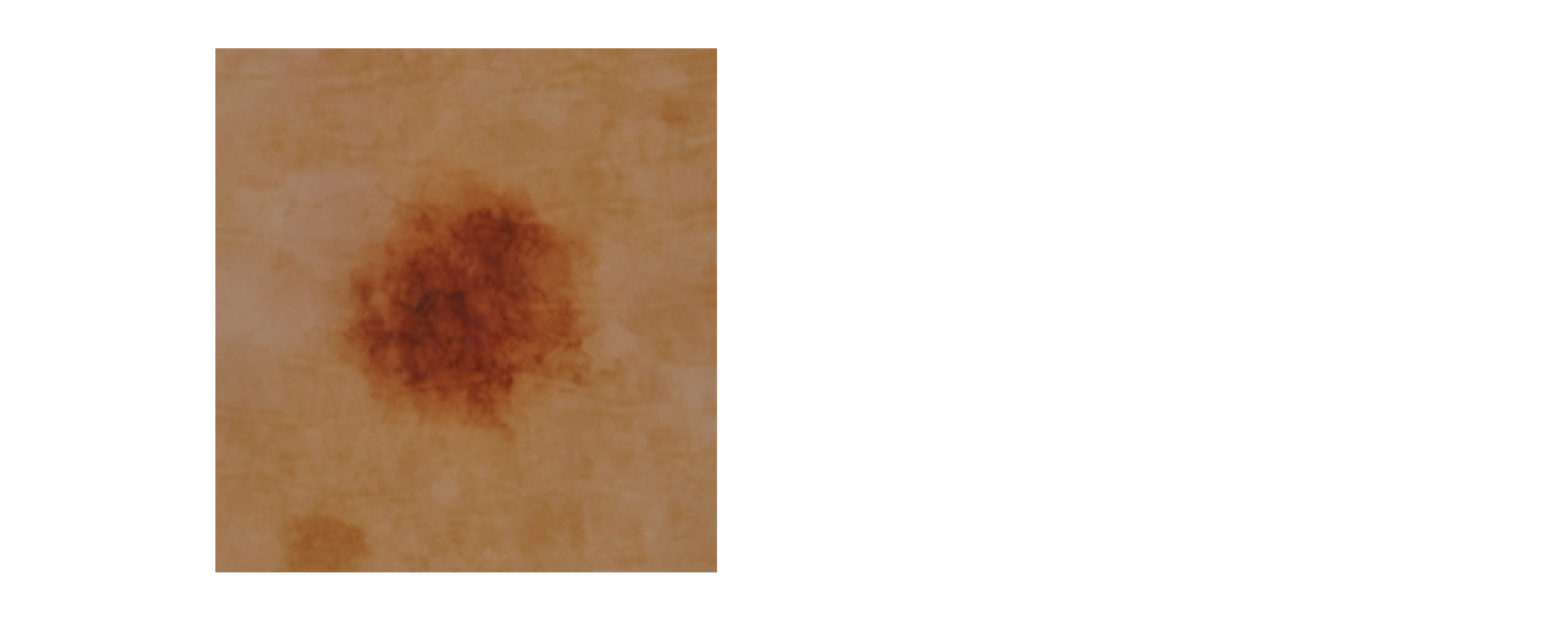

Supplement: Supplementary file 1 [file cancers-16-03077-s001.zip › cancers-3154863-supplementary/Supplementary File 2/001A.jpg]

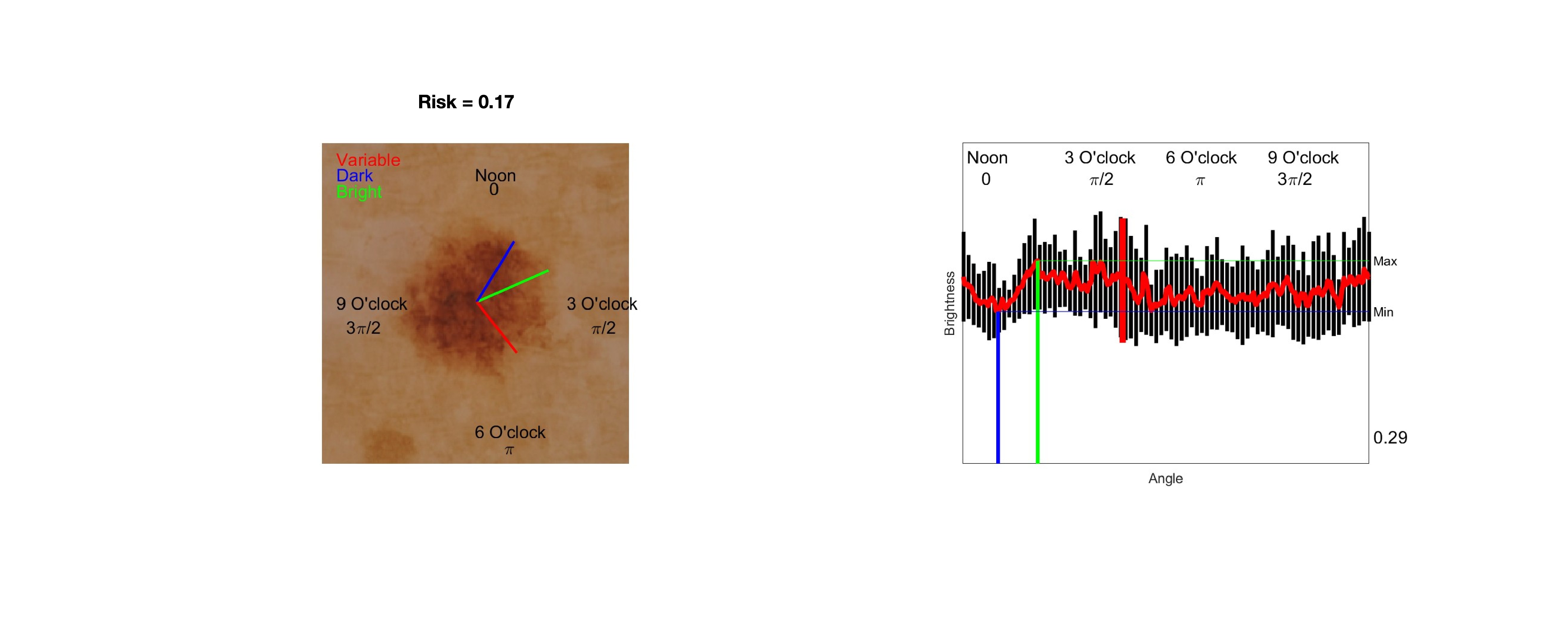

Supplement: Supplementary file 1 [file cancers-16-03077-s001.zip › cancers-3154863-supplementary/Supplementary File 2/001B.jpg]

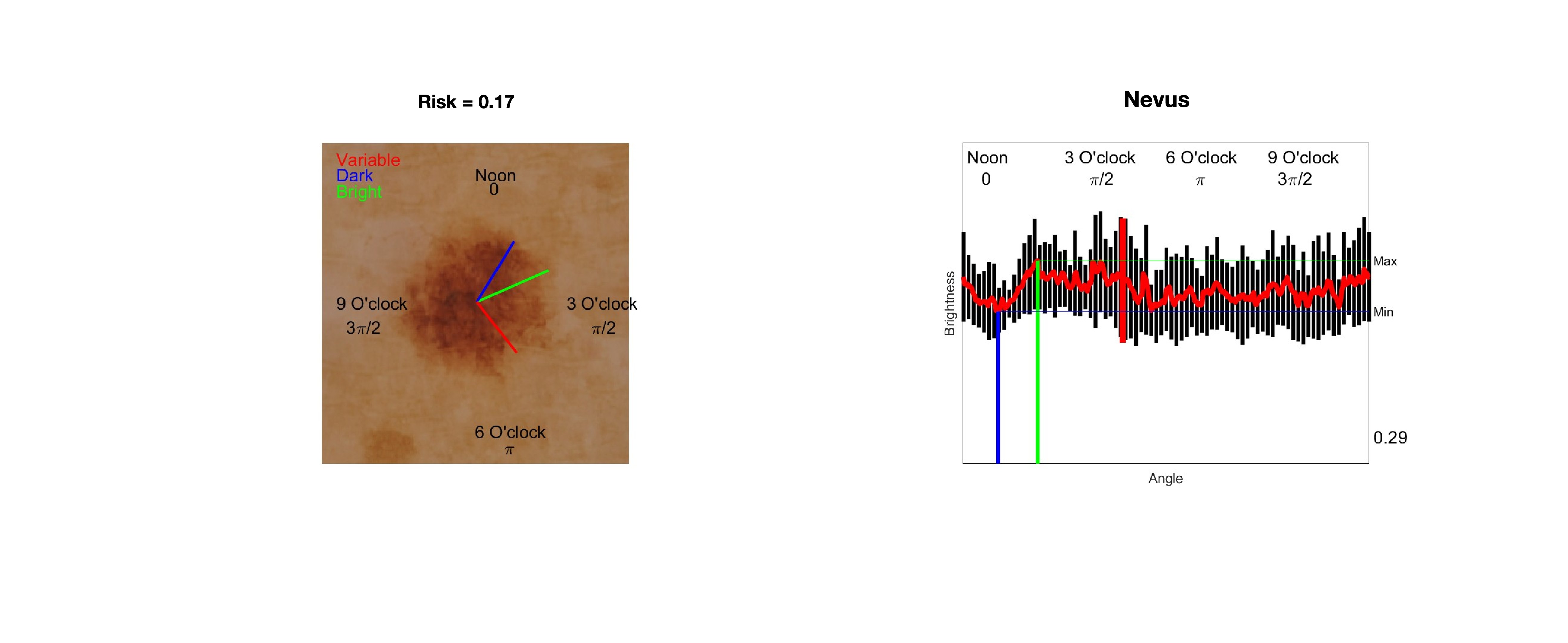

Supplement: Supplementary file 1 [file cancers-16-03077-s001.zip › cancers-3154863-supplementary/Supplementary File 2/001C.jpg]

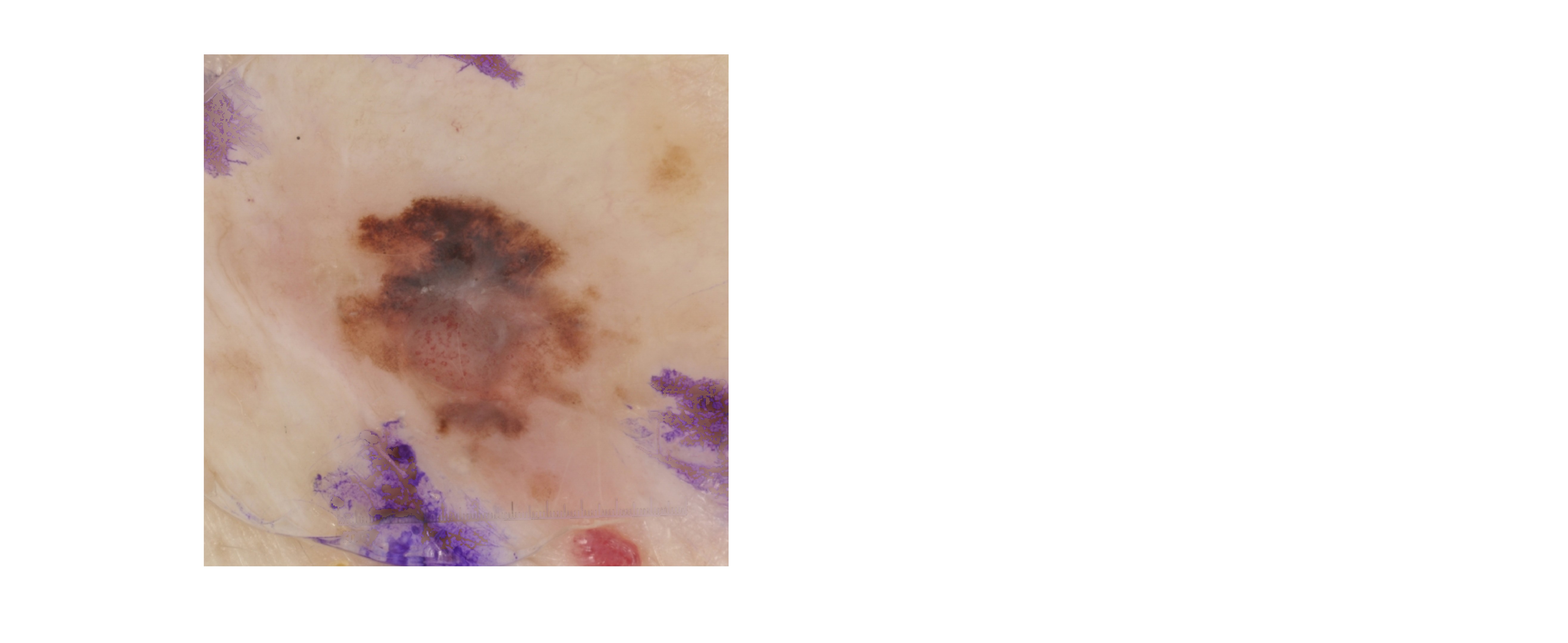

Supplement: Supplementary file 1 [file cancers-16-03077-s001.zip › cancers-3154863-supplementary/Supplementary File 2/002A.jpg]

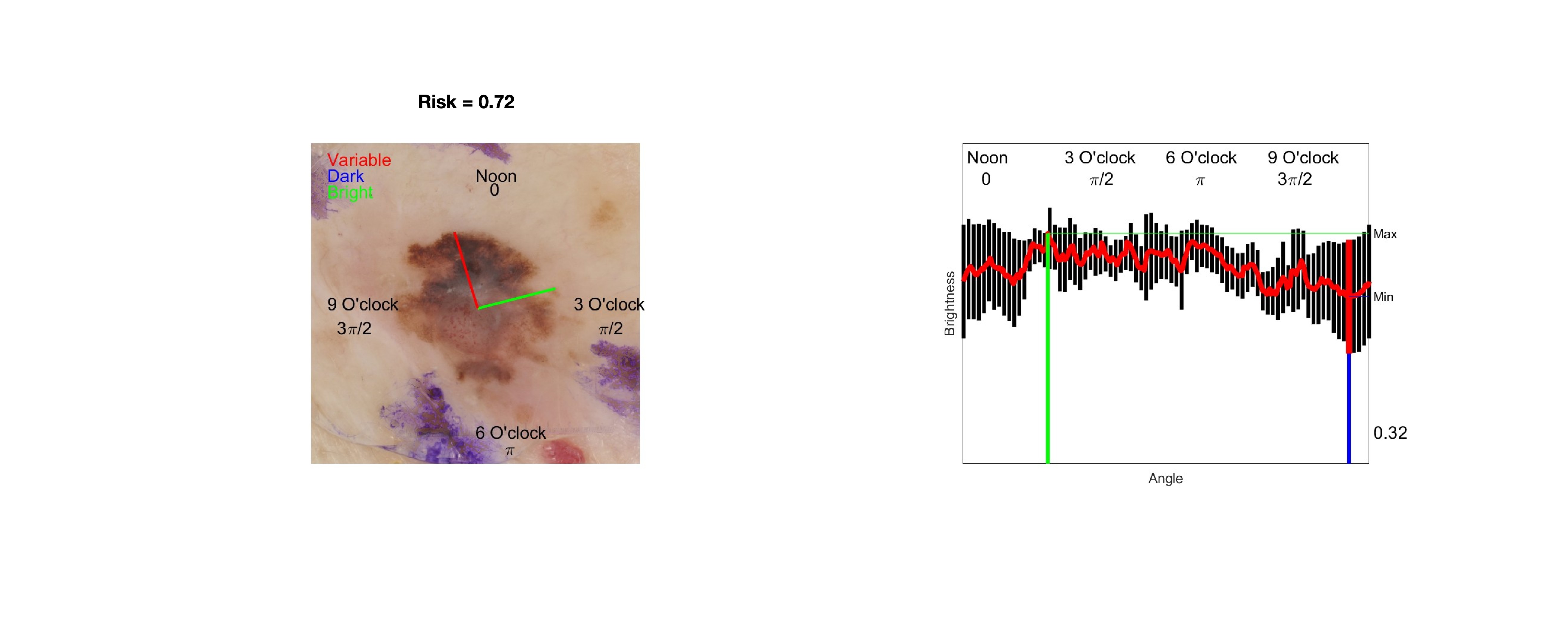

Supplement: Supplementary file 1 [file cancers-16-03077-s001.zip › cancers-3154863-supplementary/Supplementary File 2/002B.jpg]

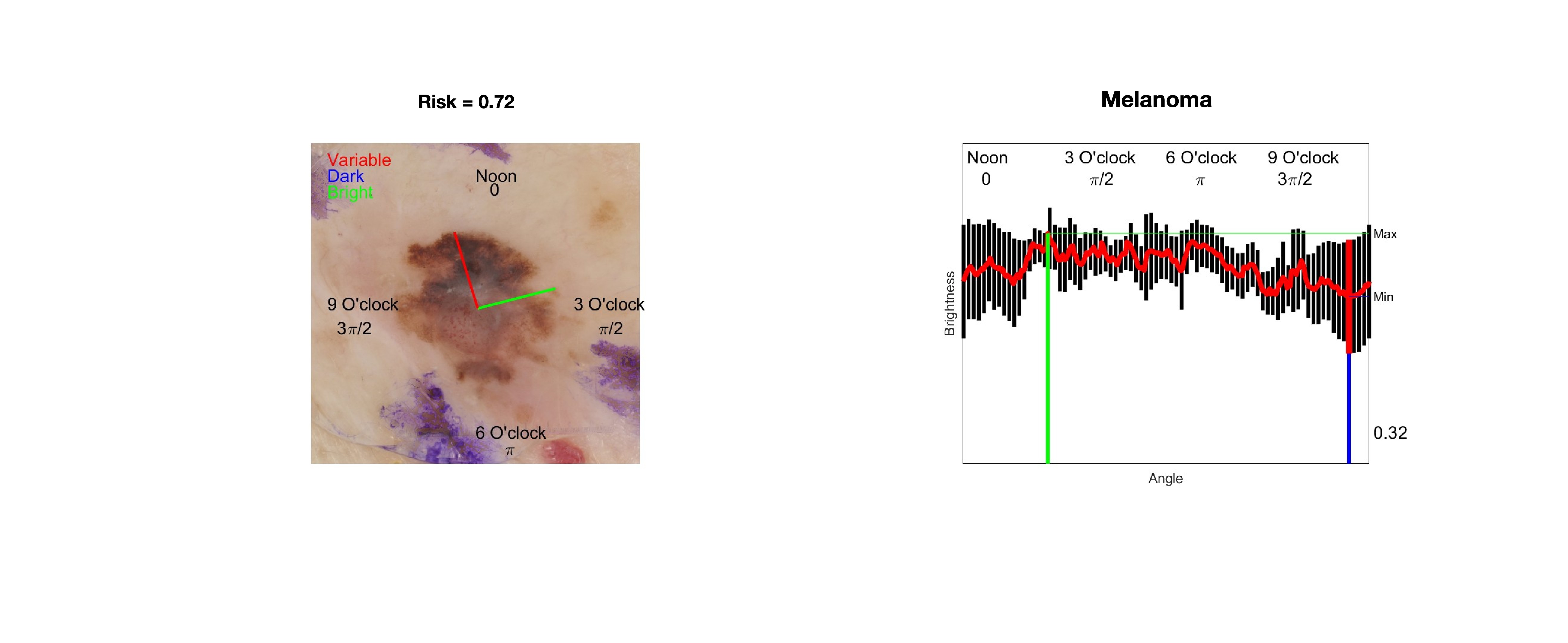

Supplement: Supplementary file 1 [file cancers-16-03077-s001.zip › cancers-3154863-supplementary/Supplementary File 2/002C.jpg]

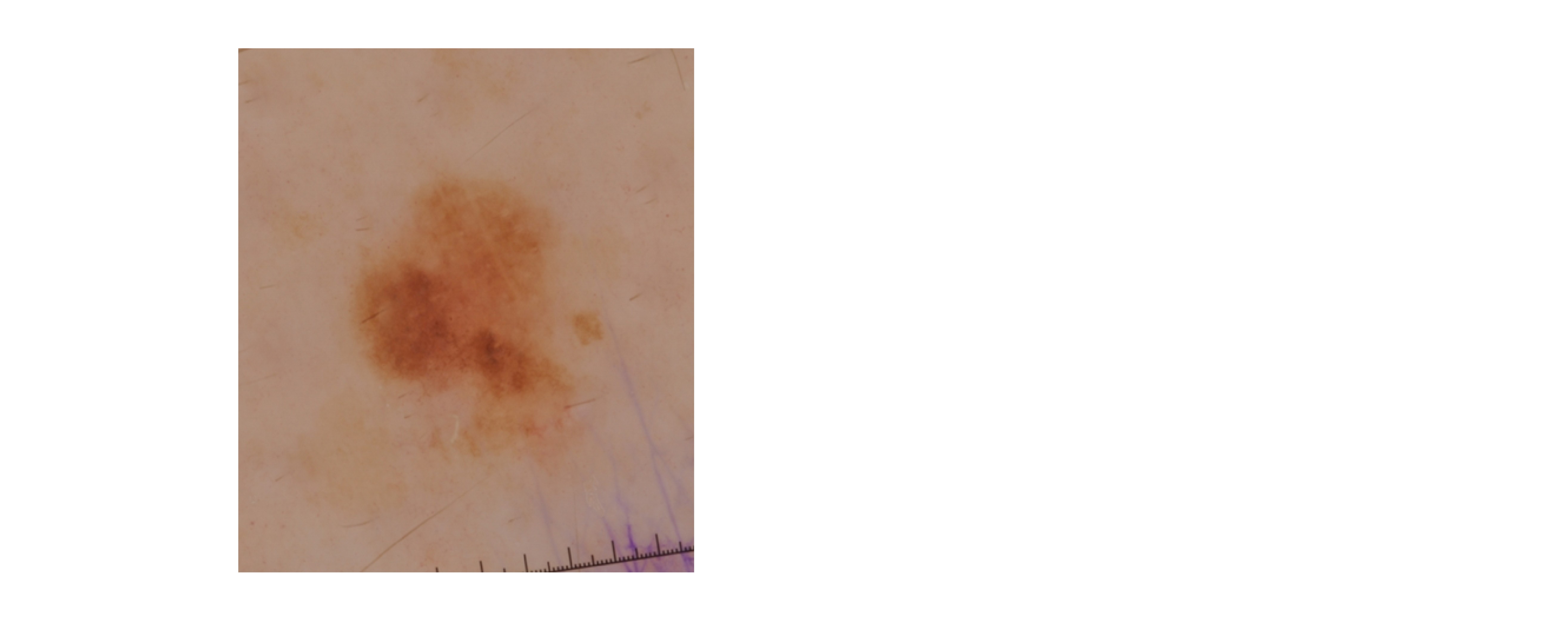

Supplement: Supplementary file 1 [file cancers-16-03077-s001.zip › cancers-3154863-supplementary/Supplementary File 2/003A.jpg]

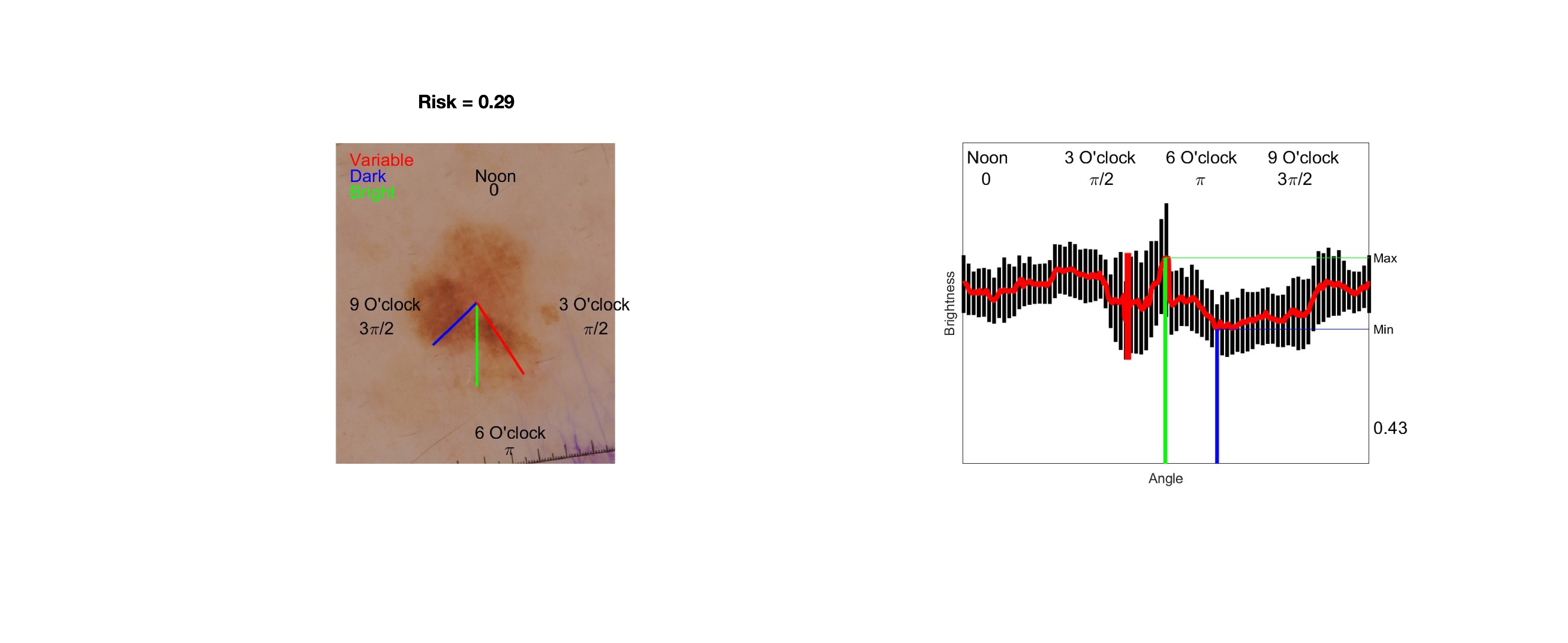

Supplement: Supplementary file 1 [file cancers-16-03077-s001.zip › cancers-3154863-supplementary/Supplementary File 2/003B.jpg]

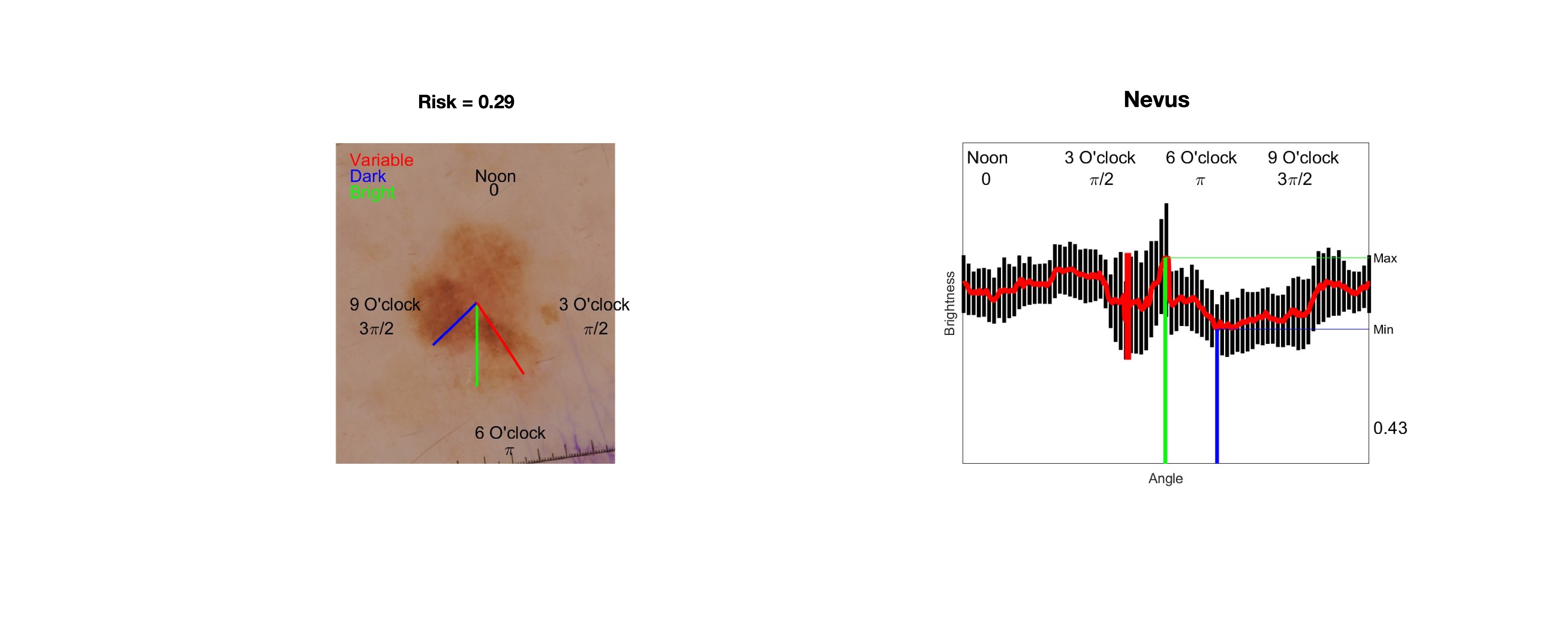

Supplement: Supplementary file 1 [file cancers-16-03077-s001.zip › cancers-3154863-supplementary/Supplementary File 2/003C.jpg]

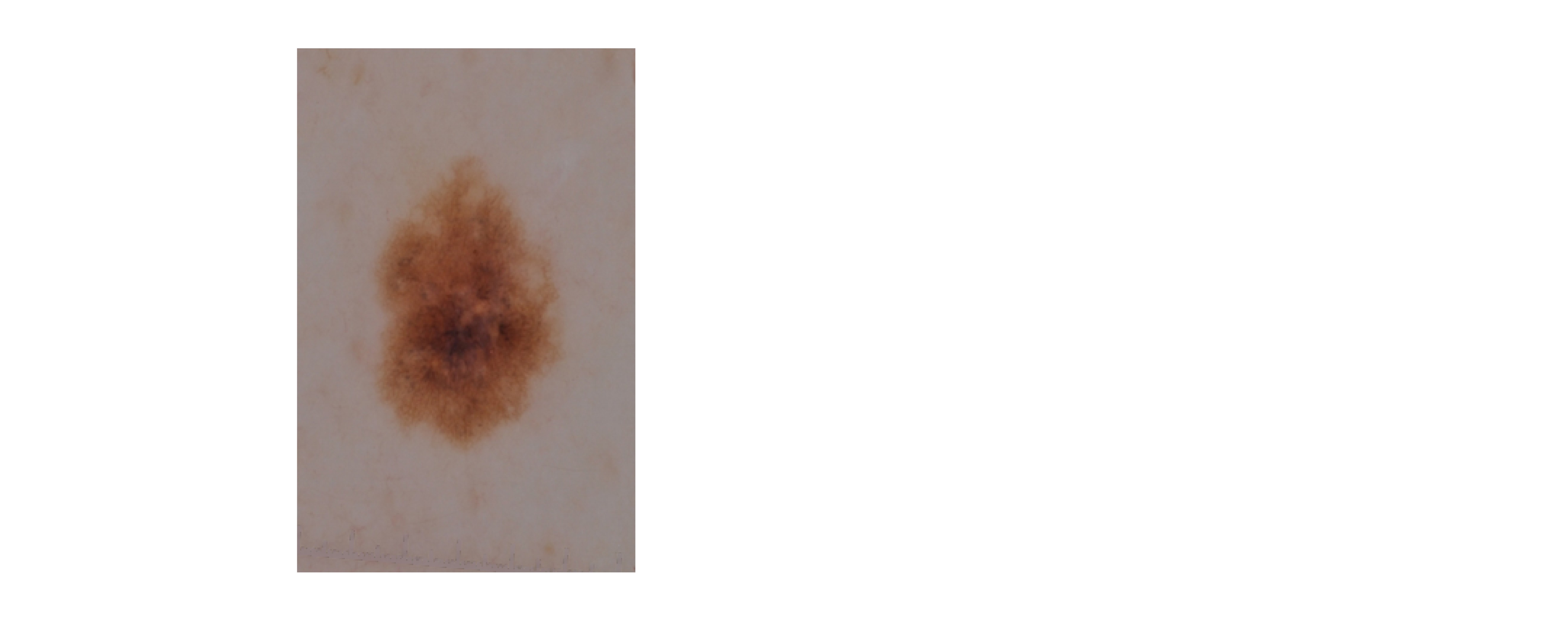

Supplement: Supplementary file 1 [file cancers-16-03077-s001.zip › cancers-3154863-supplementary/Supplementary File 2/004A.jpg]

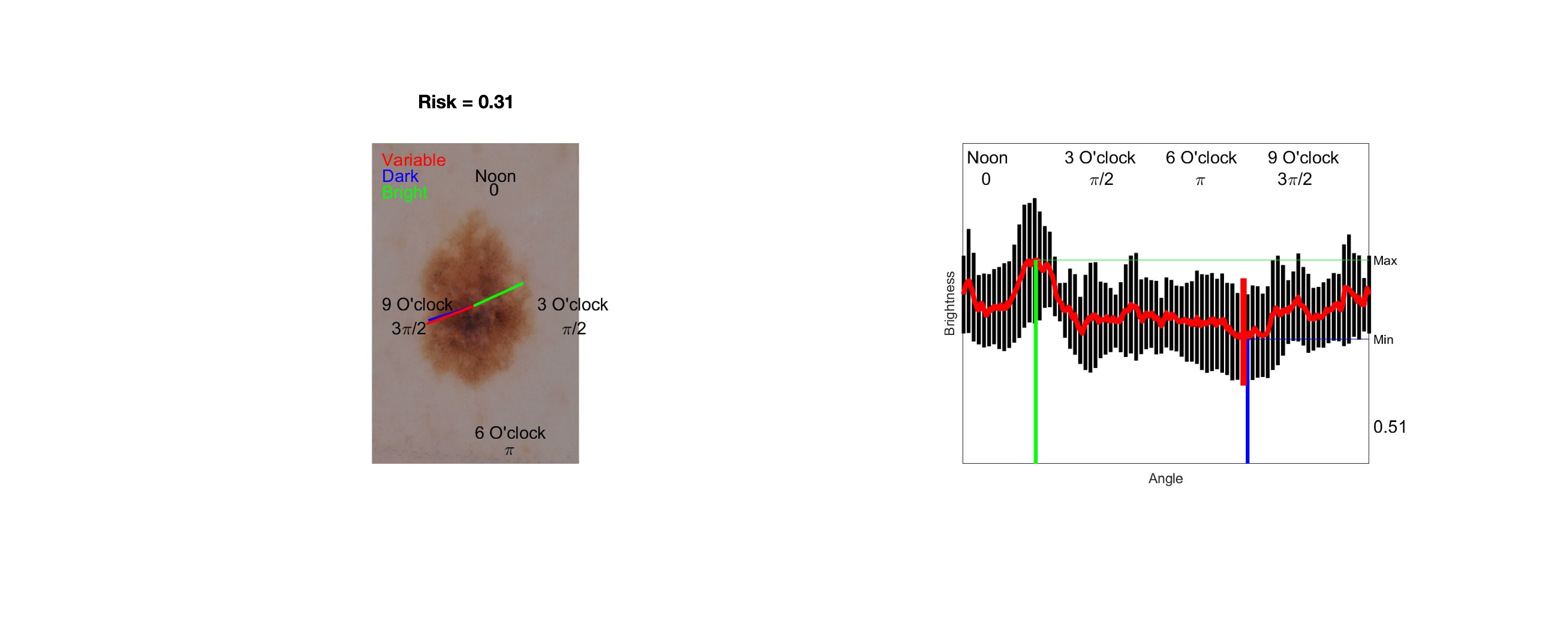

Supplement: Supplementary file 1 [file cancers-16-03077-s001.zip › cancers-3154863-supplementary/Supplementary File 2/004B.jpg]

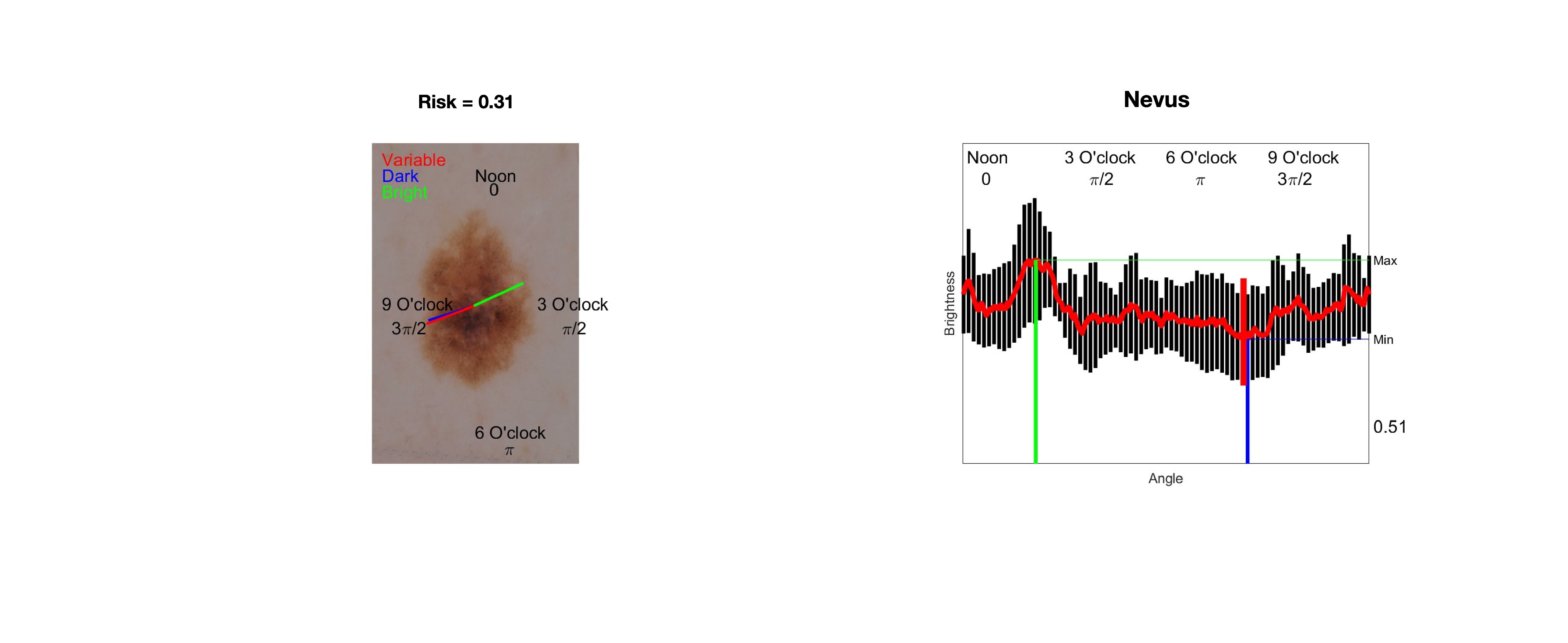

Supplement: Supplementary file 1 [file cancers-16-03077-s001.zip › cancers-3154863-supplementary/Supplementary File 2/004C.jpg]

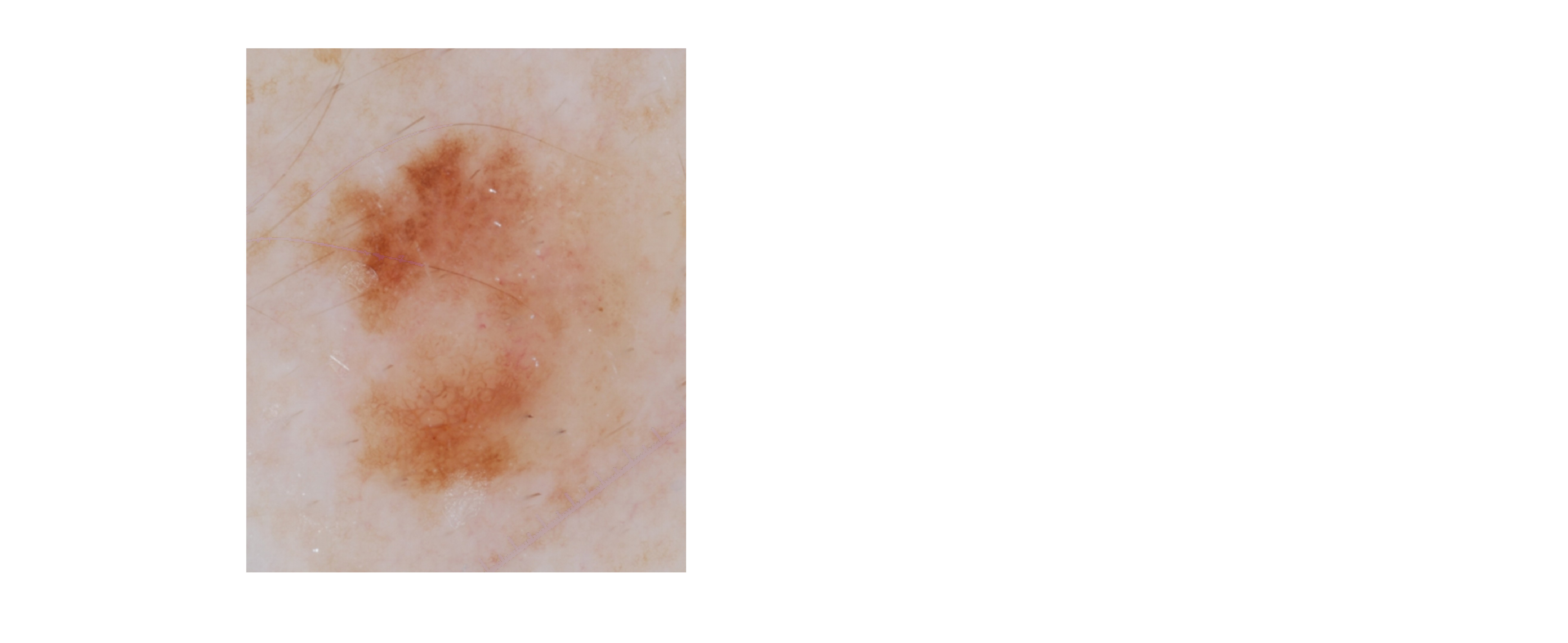

Supplement: Supplementary file 1 [file cancers-16-03077-s001.zip › cancers-3154863-supplementary/Supplementary File 2/005A.jpg]

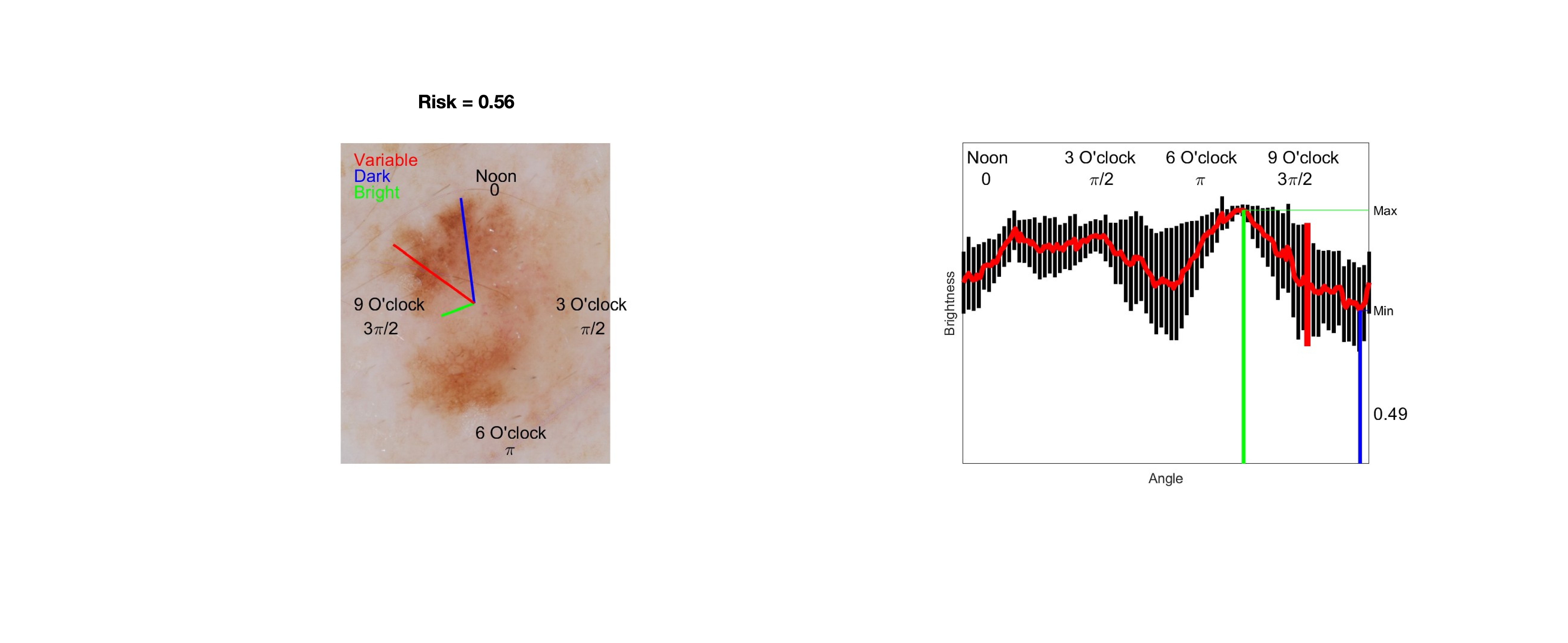

Supplement: Supplementary file 1 [file cancers-16-03077-s001.zip › cancers-3154863-supplementary/Supplementary File 2/005B.jpg]

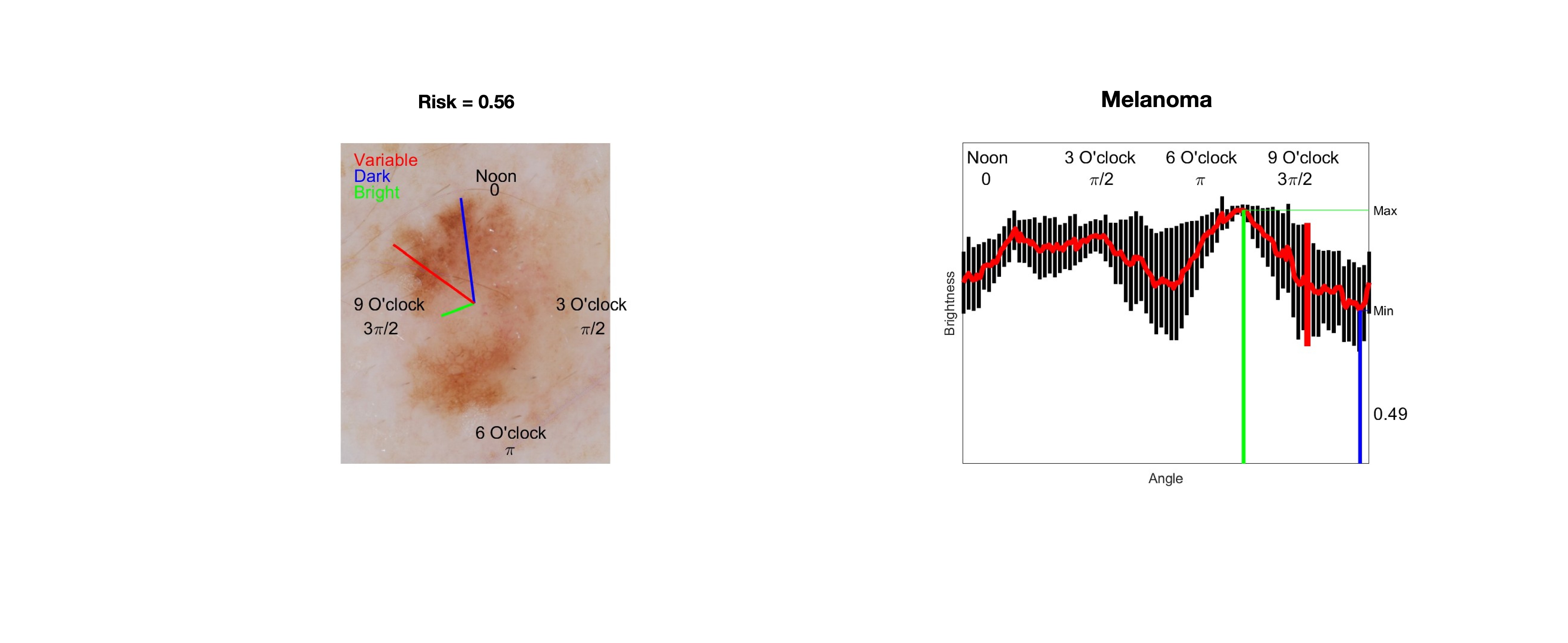

Supplement: Supplementary file 1 [file cancers-16-03077-s001.zip › cancers-3154863-supplementary/Supplementary File 2/005C.jpg]

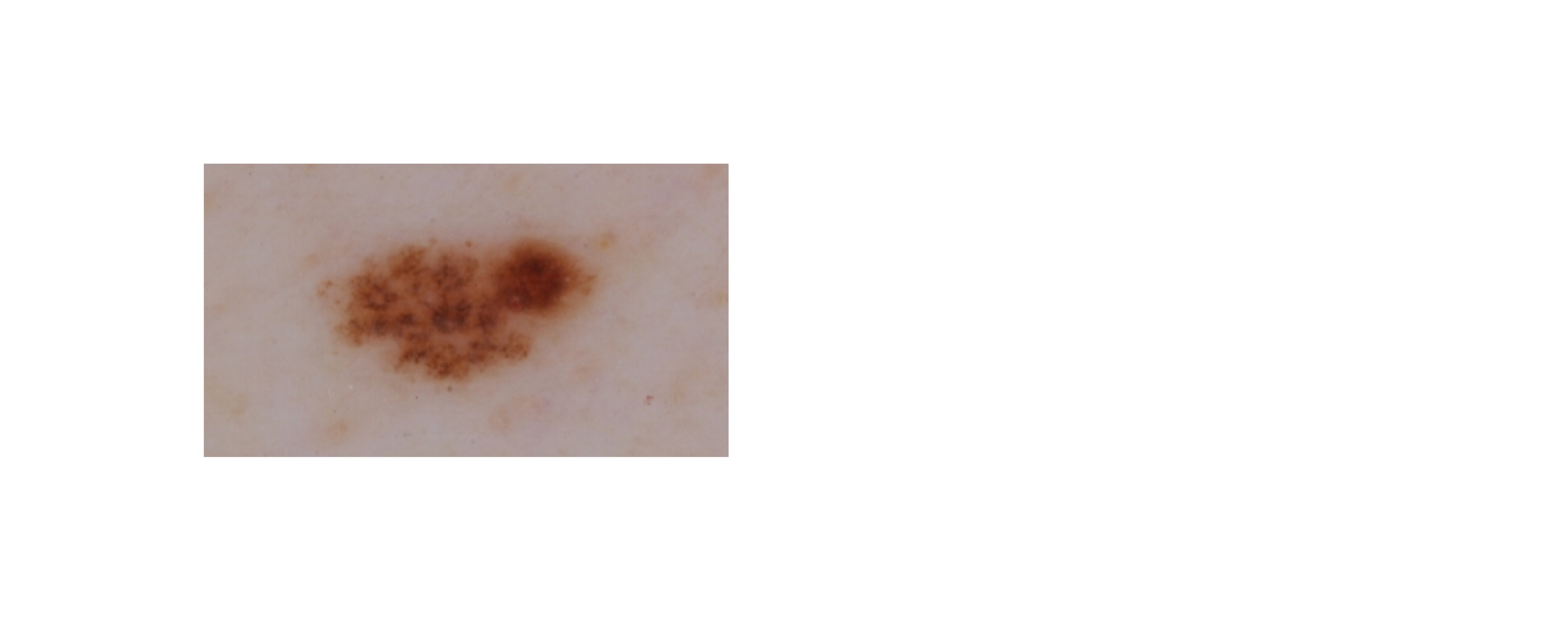

Supplement: Supplementary file 1 [file cancers-16-03077-s001.zip › cancers-3154863-supplementary/Supplementary File 2/006A.jpg]

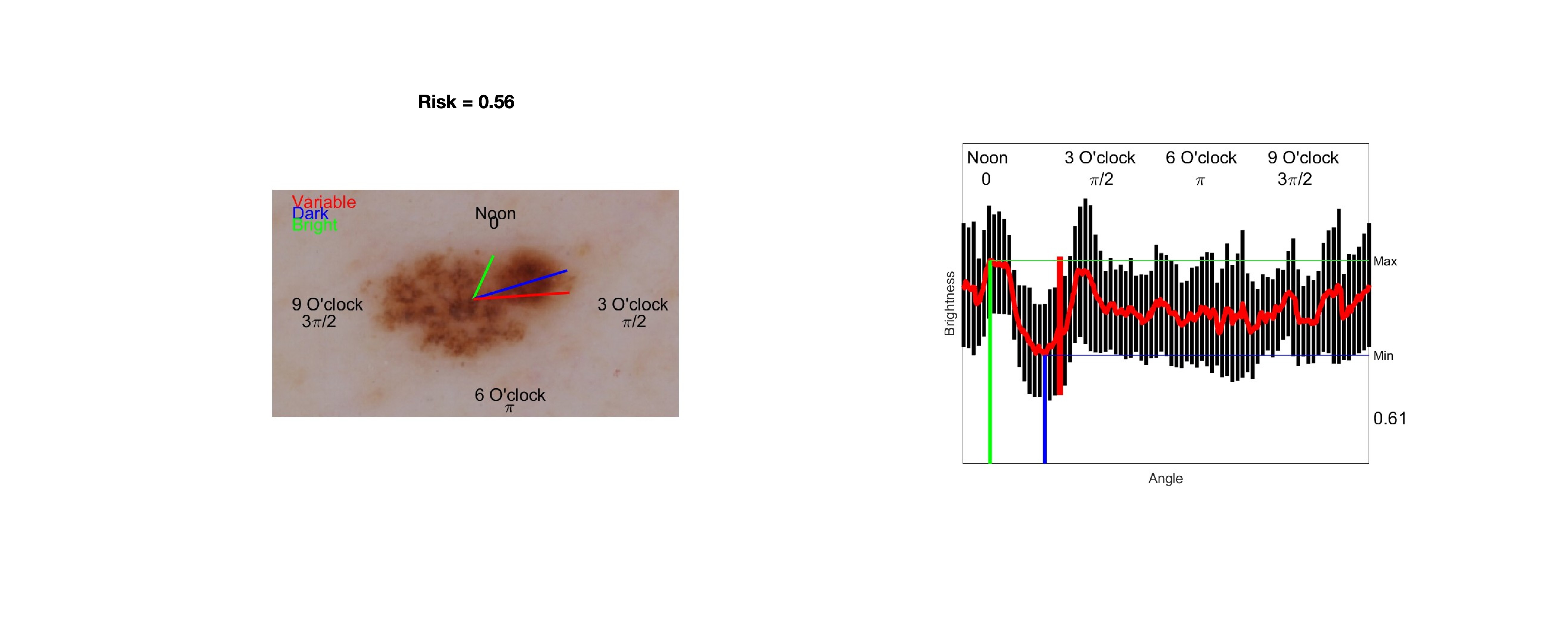

Supplement: Supplementary file 1 [file cancers-16-03077-s001.zip › cancers-3154863-supplementary/Supplementary File 2/006B.jpg]

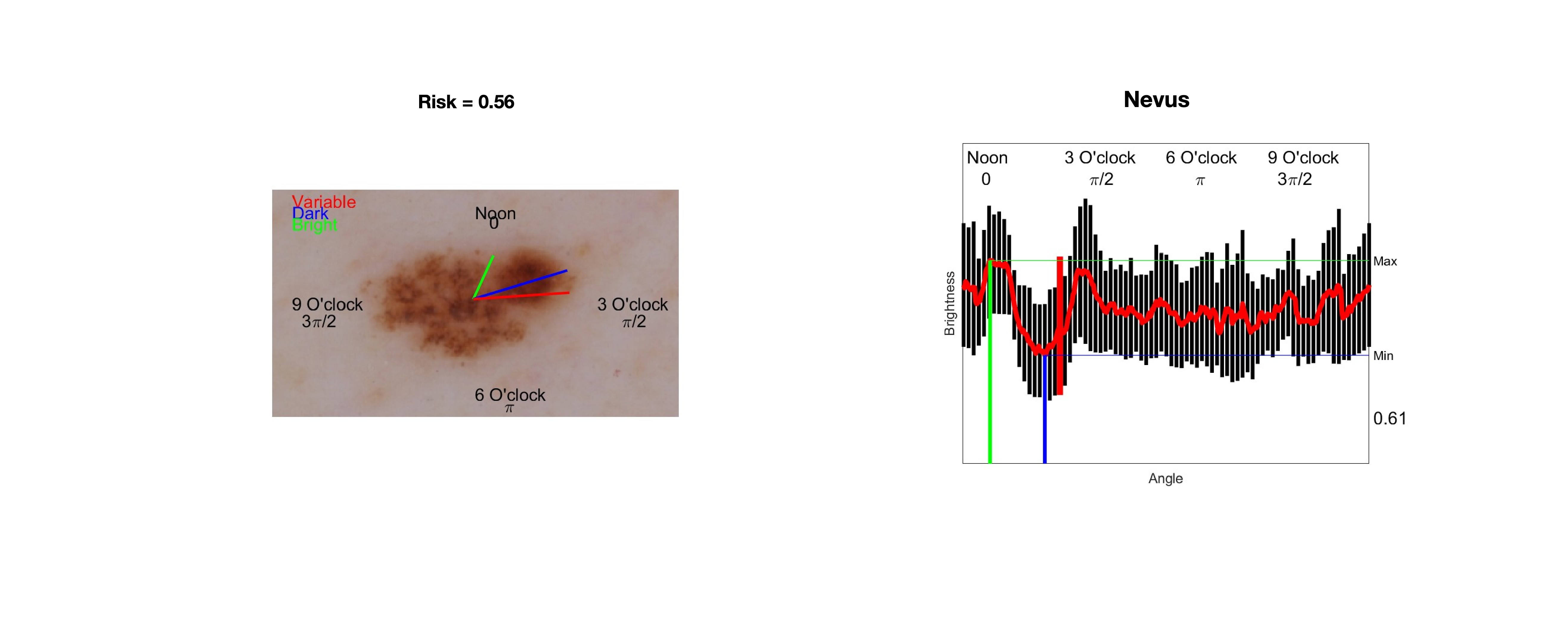

Supplement: Supplementary file 1 [file cancers-16-03077-s001.zip › cancers-3154863-supplementary/Supplementary File 2/006C.jpg]

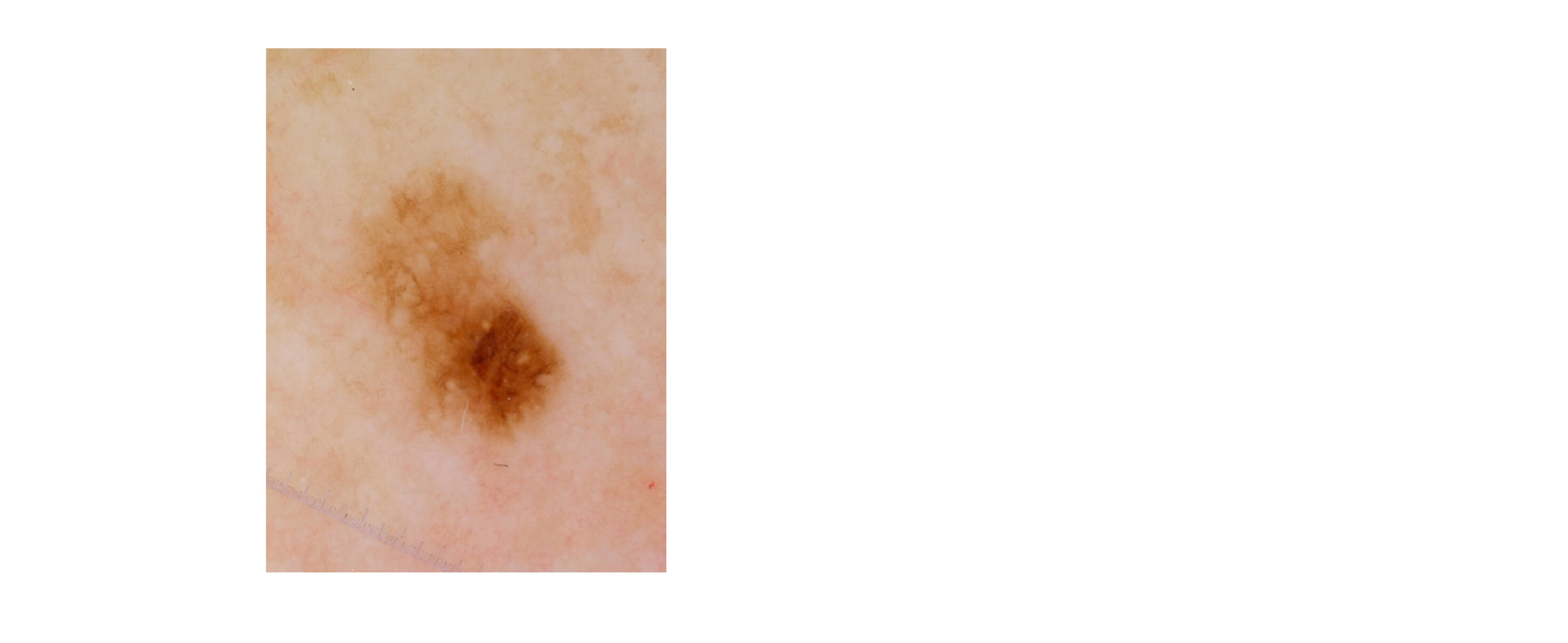

Supplement: Supplementary file 1 [file cancers-16-03077-s001.zip › cancers-3154863-supplementary/Supplementary File 2/007A.jpg]

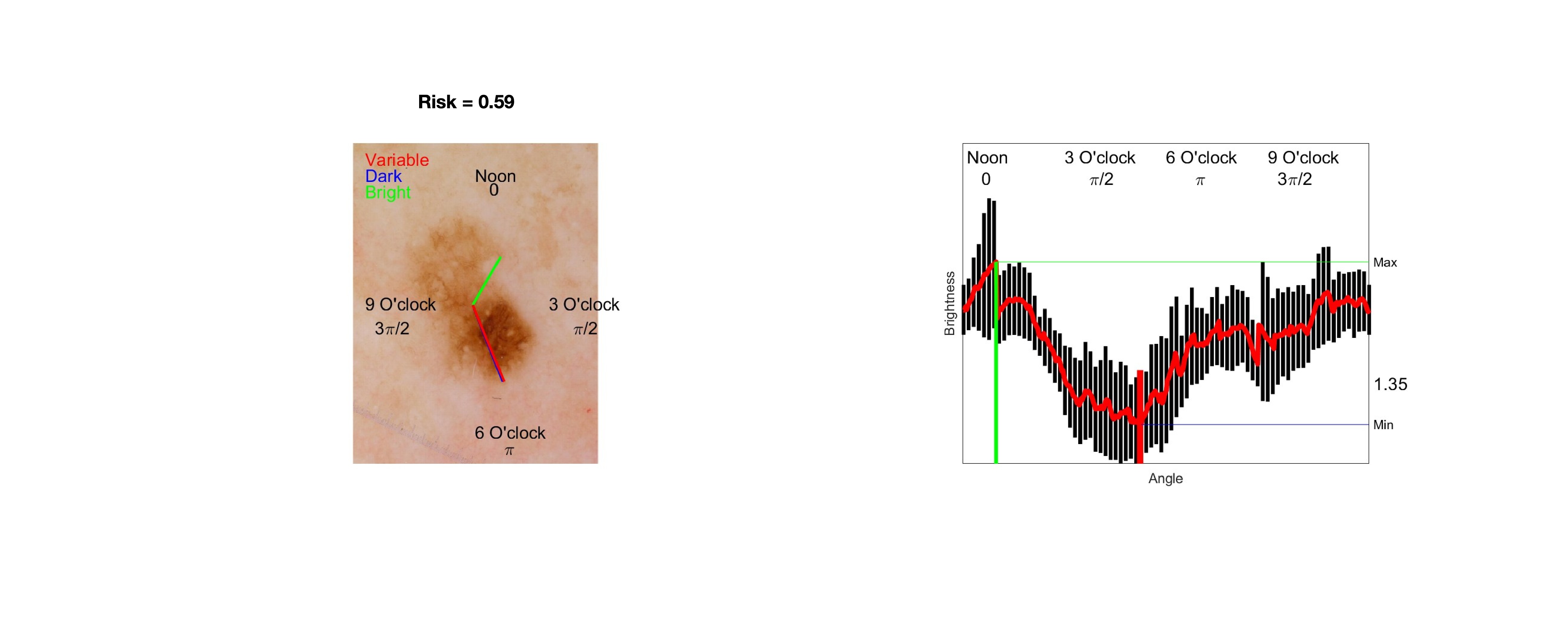

Supplement: Supplementary file 1 [file cancers-16-03077-s001.zip › cancers-3154863-supplementary/Supplementary File 2/007B.jpg]

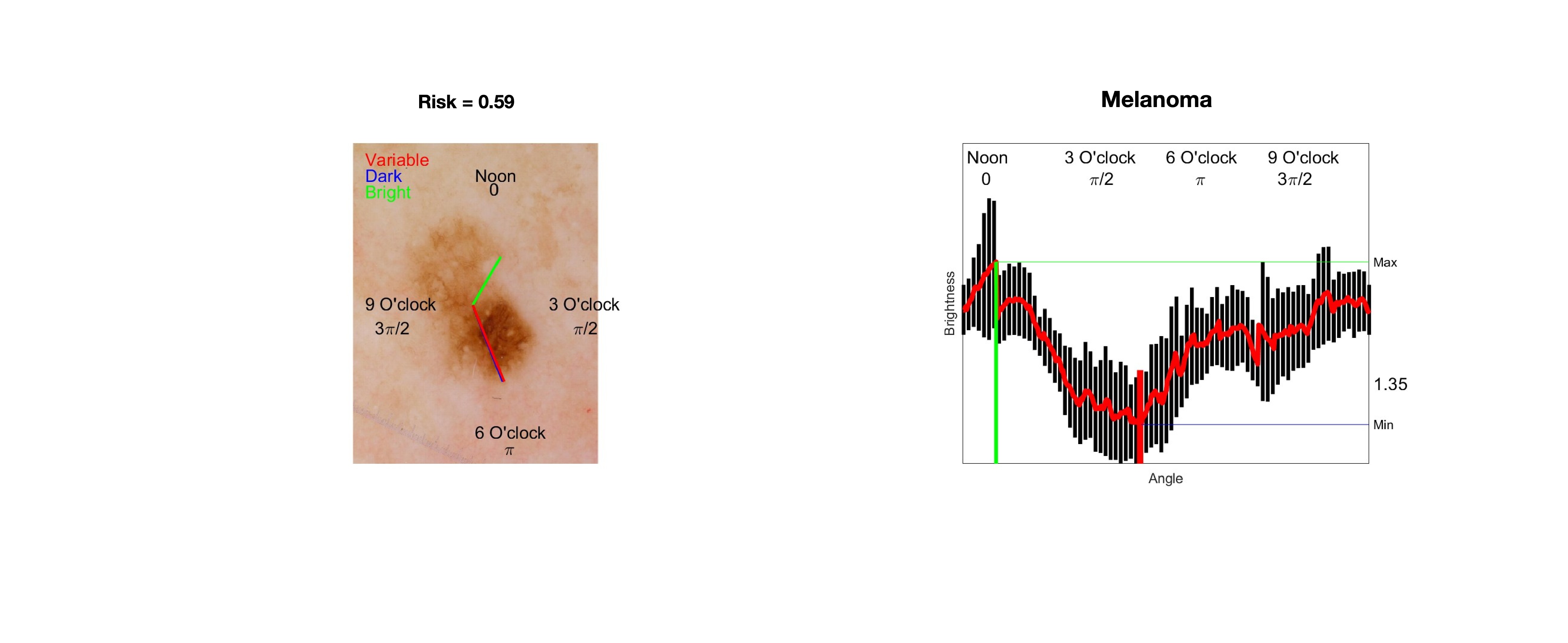

Supplement: Supplementary file 1 [file cancers-16-03077-s001.zip › cancers-3154863-supplementary/Supplementary File 2/007C.jpg]

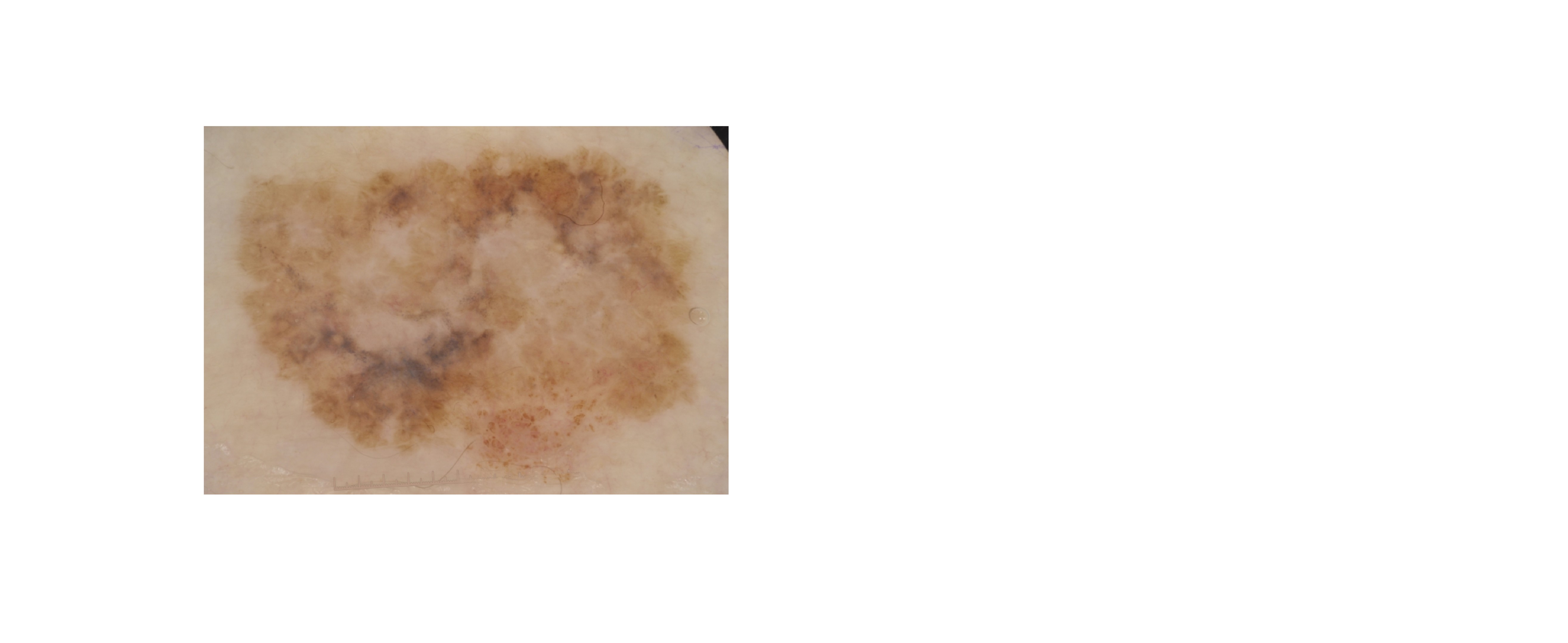

Supplement: Supplementary file 1 [file cancers-16-03077-s001.zip › cancers-3154863-supplementary/Supplementary File 2/008A.jpg]

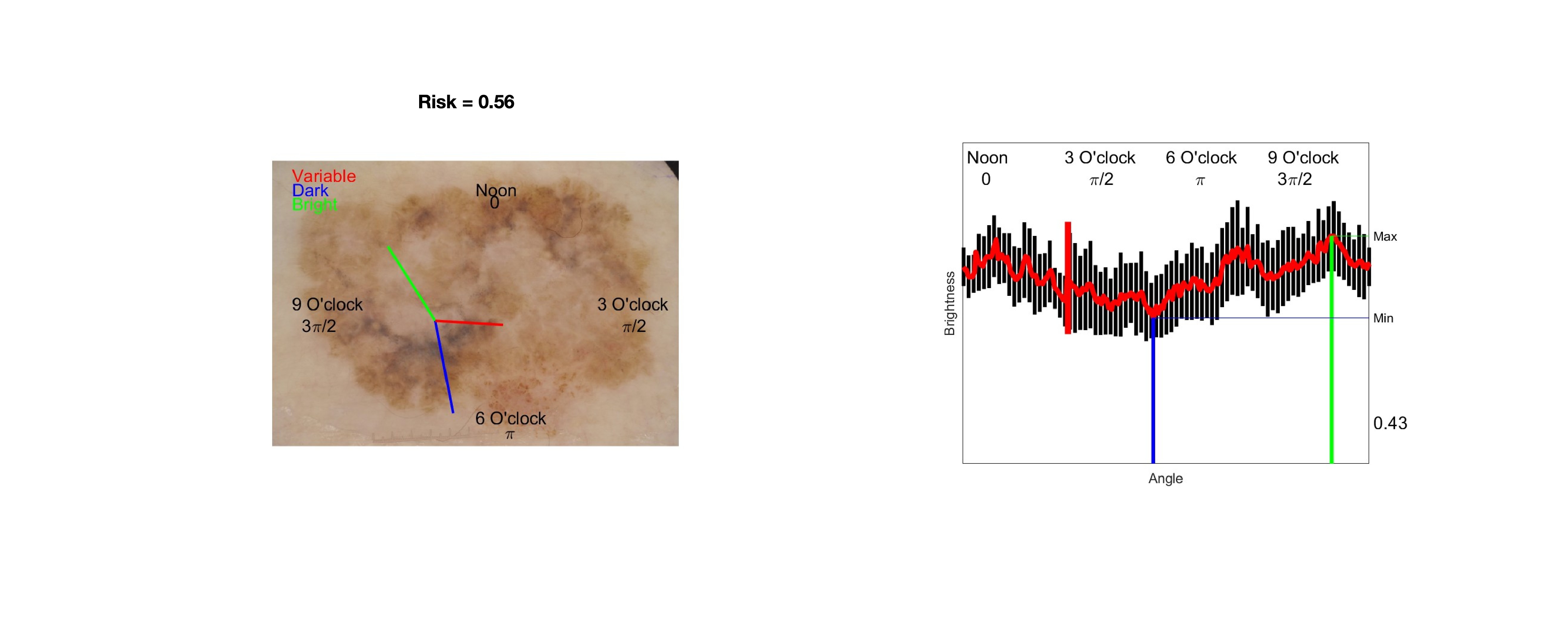

Supplement: Supplementary file 1 [file cancers-16-03077-s001.zip › cancers-3154863-supplementary/Supplementary File 2/008B.jpg]

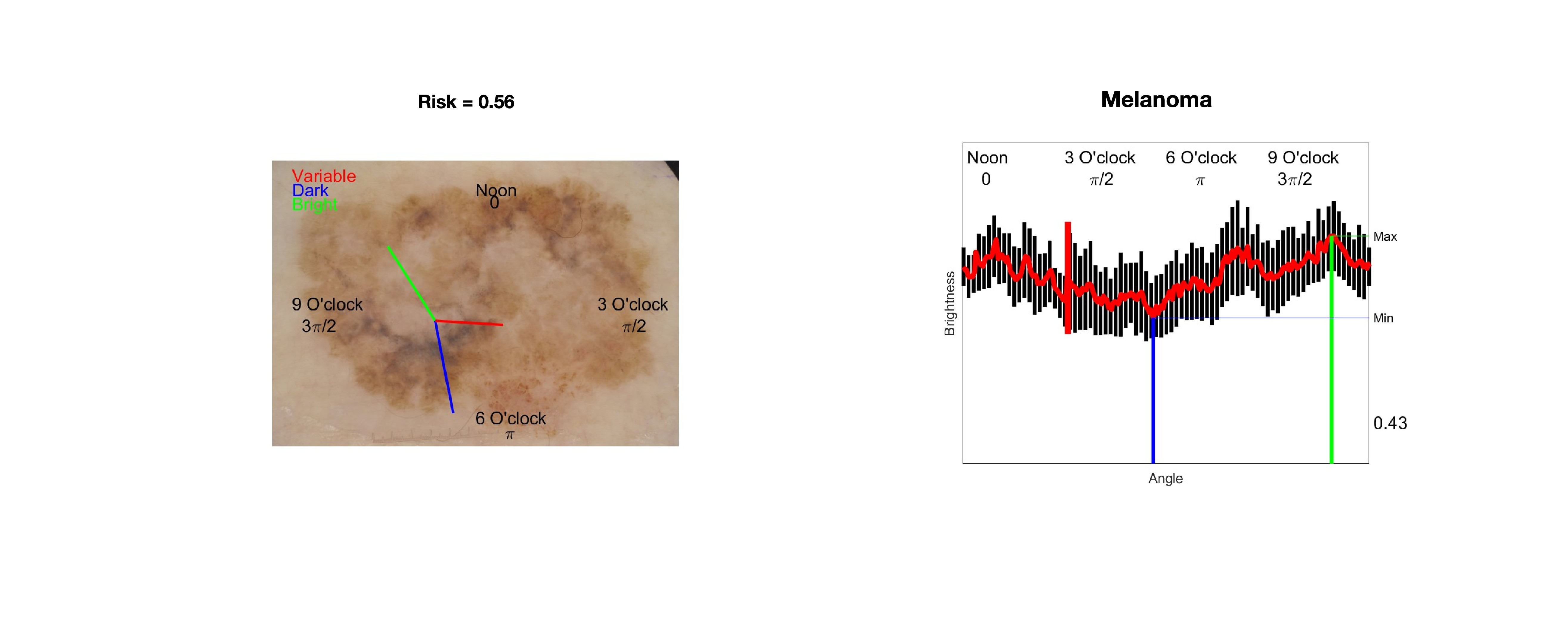

Supplement: Supplementary file 1 [file cancers-16-03077-s001.zip › cancers-3154863-supplementary/Supplementary File 2/008C.jpg]

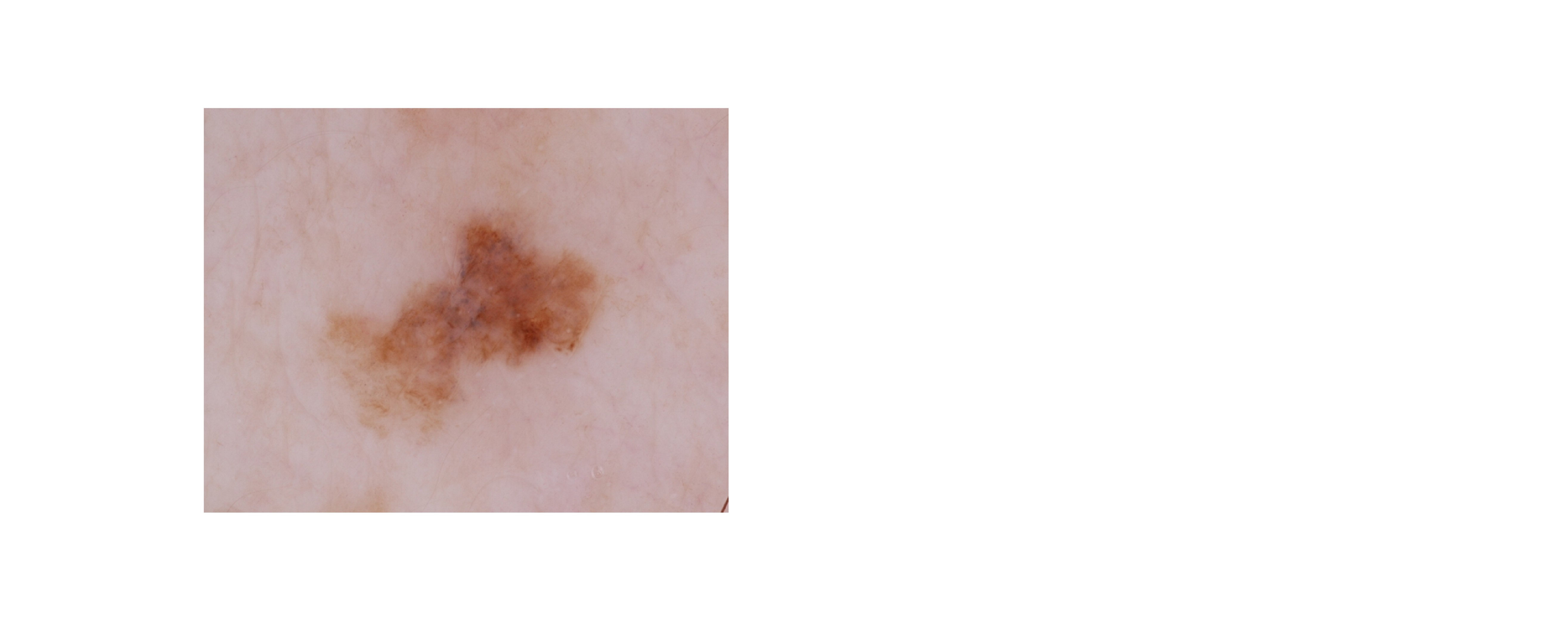

Supplement: Supplementary file 1 [file cancers-16-03077-s001.zip › cancers-3154863-supplementary/Supplementary File 2/009A.jpg]

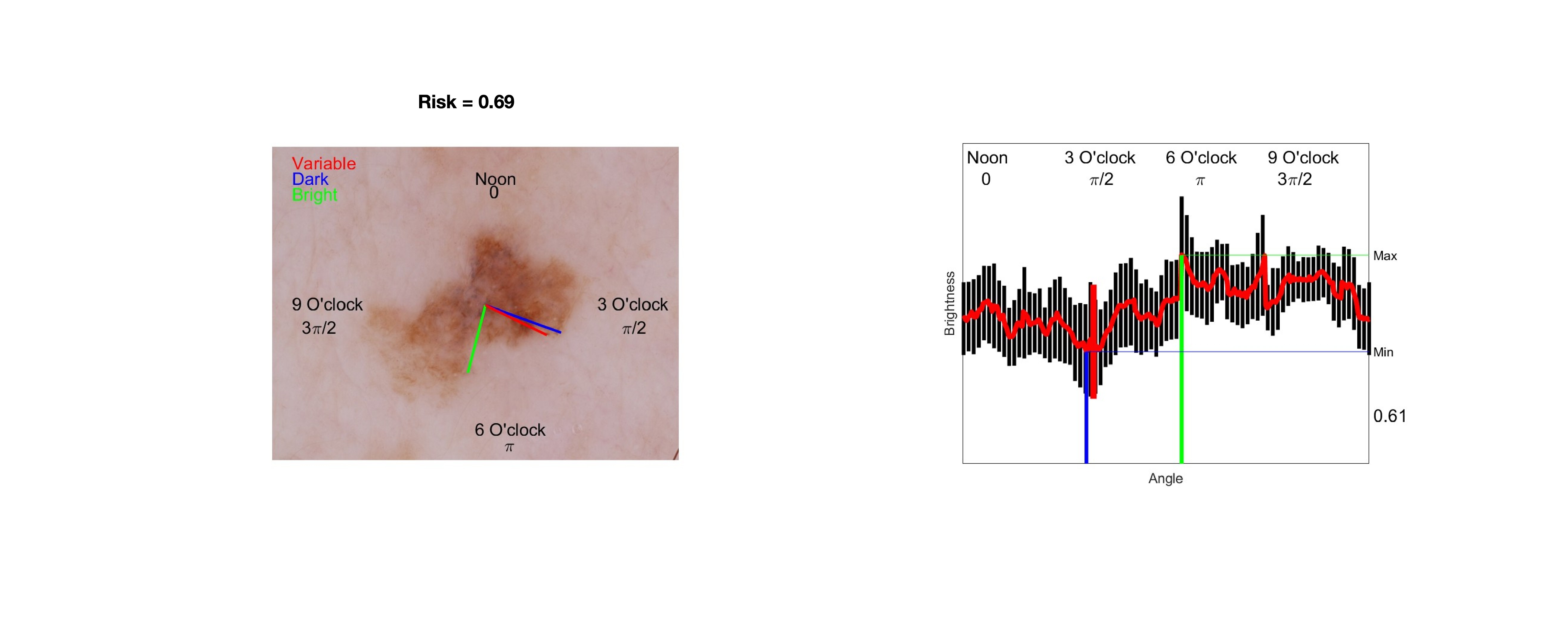

Supplement: Supplementary file 1 [file cancers-16-03077-s001.zip › cancers-3154863-supplementary/Supplementary File 2/009B.jpg]

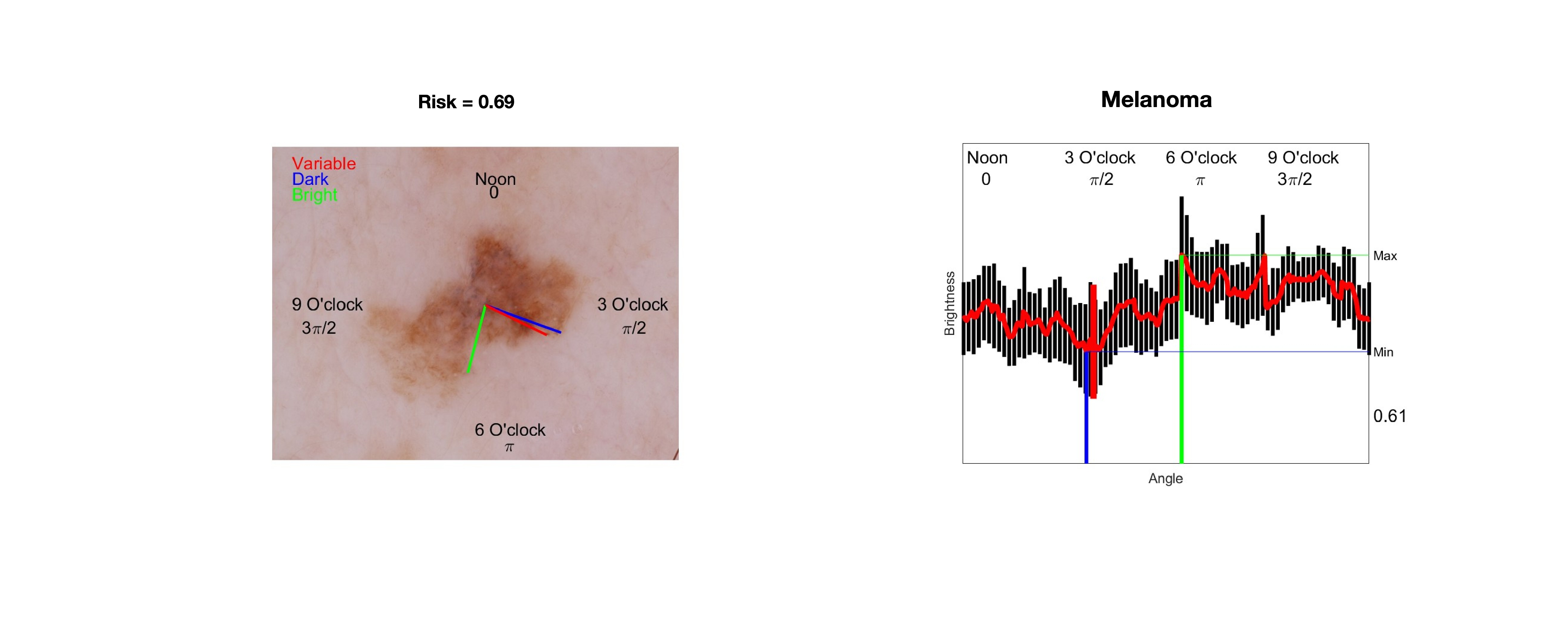

Supplement: Supplementary file 1 [file cancers-16-03077-s001.zip › cancers-3154863-supplementary/Supplementary File 2/009C.jpg]

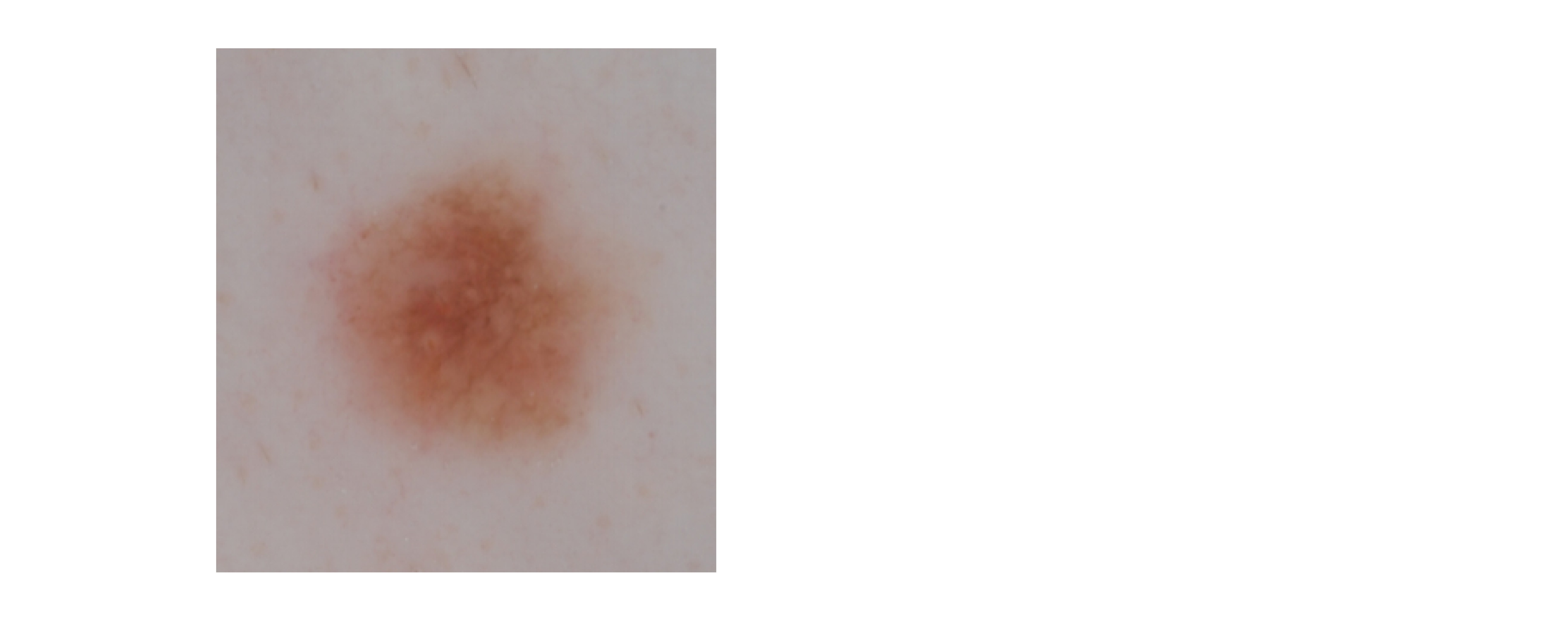

Supplement: Supplementary file 1 [file cancers-16-03077-s001.zip › cancers-3154863-supplementary/Supplementary File 2/010A.jpg]

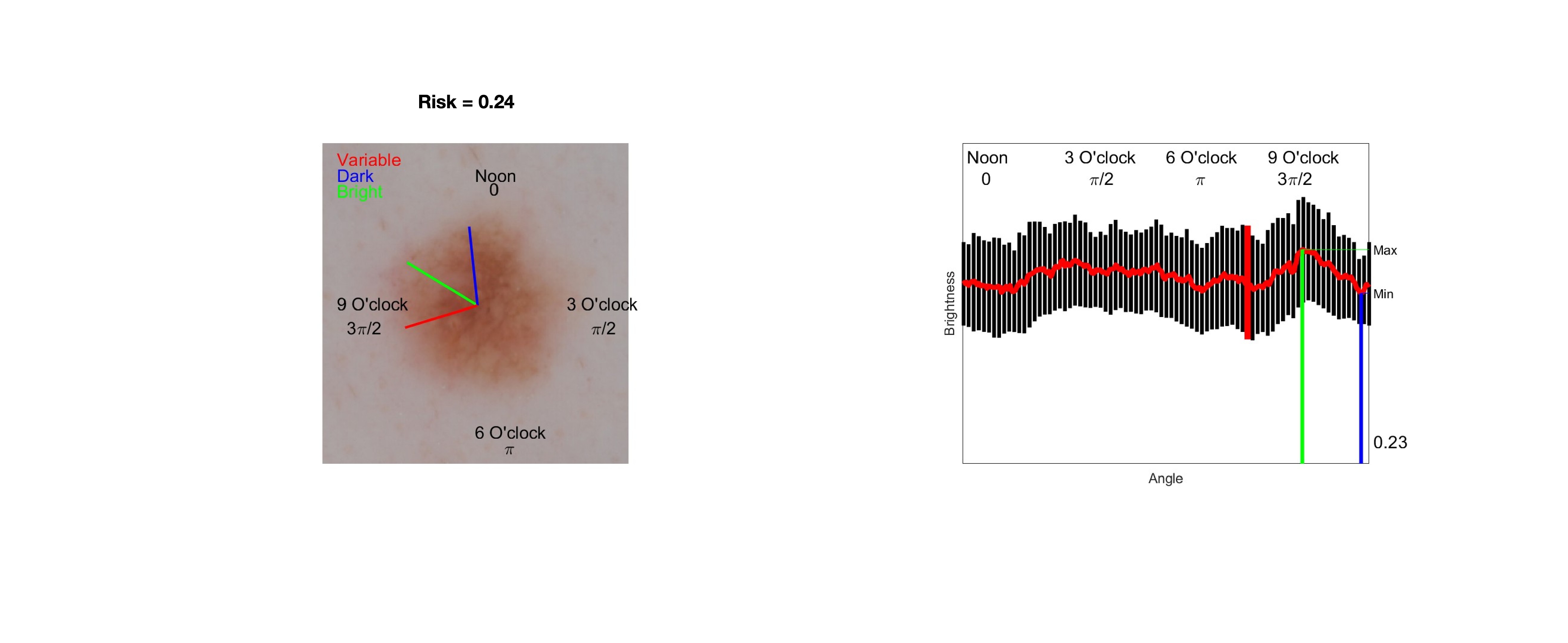

Supplement: Supplementary file 1 [file cancers-16-03077-s001.zip › cancers-3154863-supplementary/Supplementary File 2/010B.jpg]

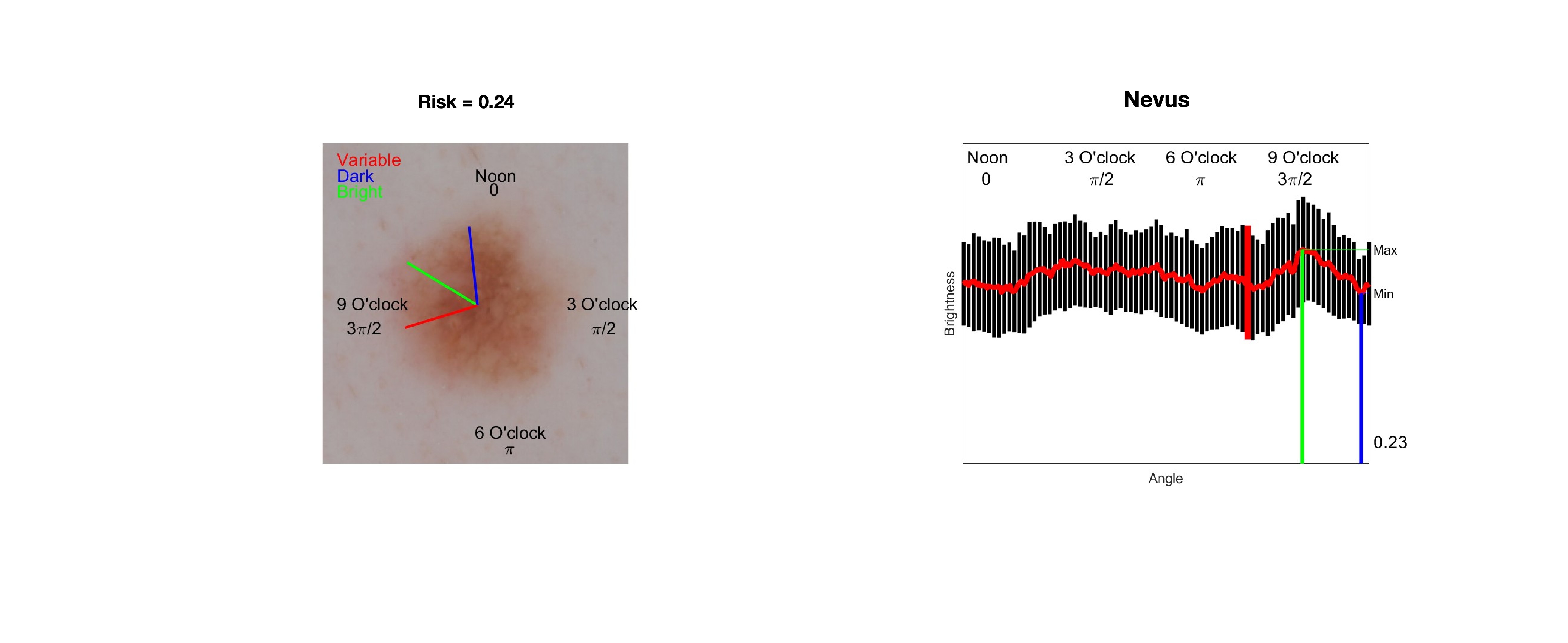

Supplement: Supplementary file 1 [file cancers-16-03077-s001.zip › cancers-3154863-supplementary/Supplementary File 2/010C.jpg]

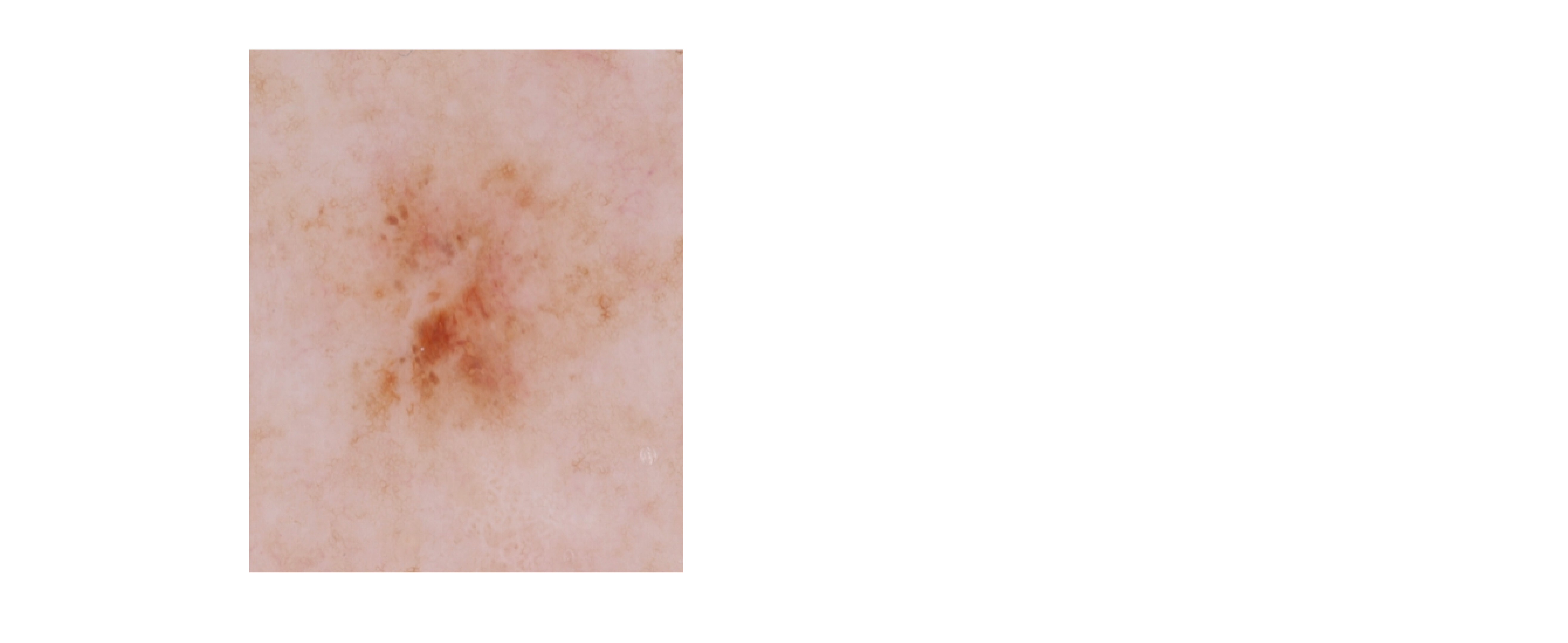

Supplement: Supplementary file 1 [file cancers-16-03077-s001.zip › cancers-3154863-supplementary/Supplementary File 2/011A.jpg]

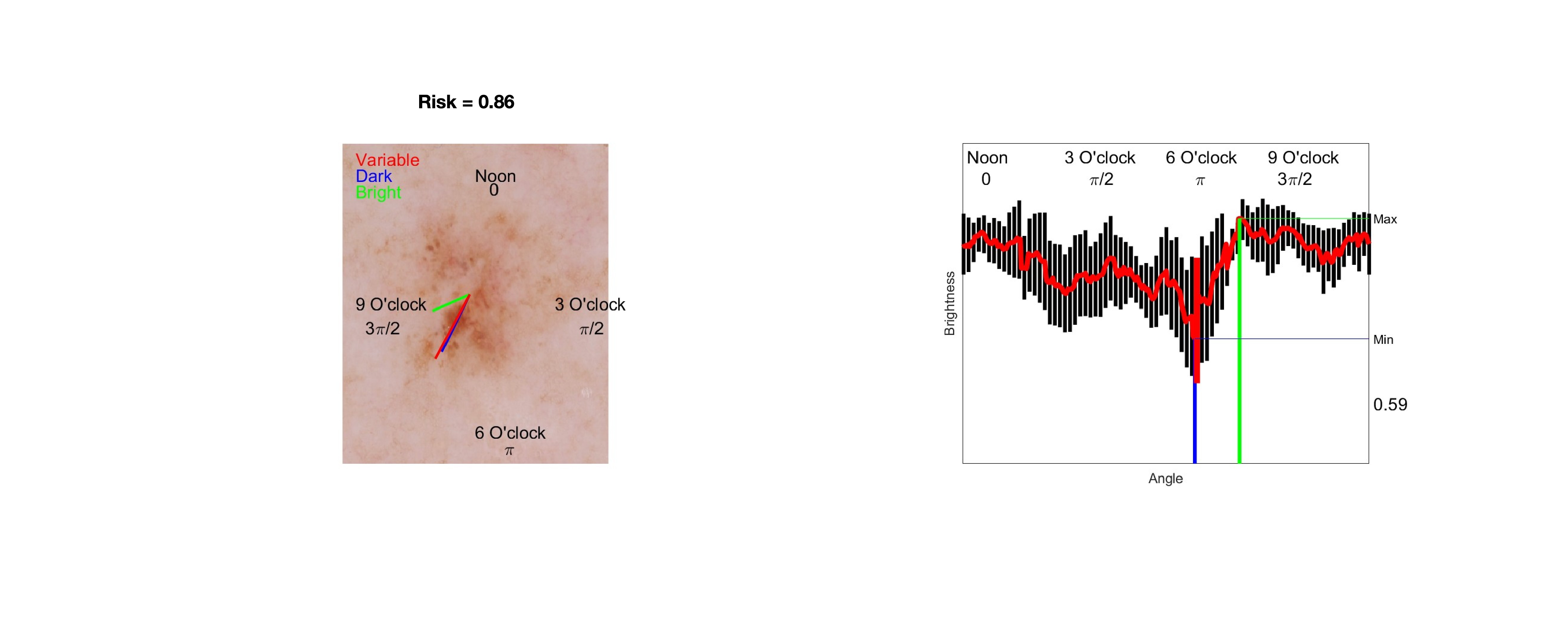

Supplement: Supplementary file 1 [file cancers-16-03077-s001.zip › cancers-3154863-supplementary/Supplementary File 2/011B.jpg]

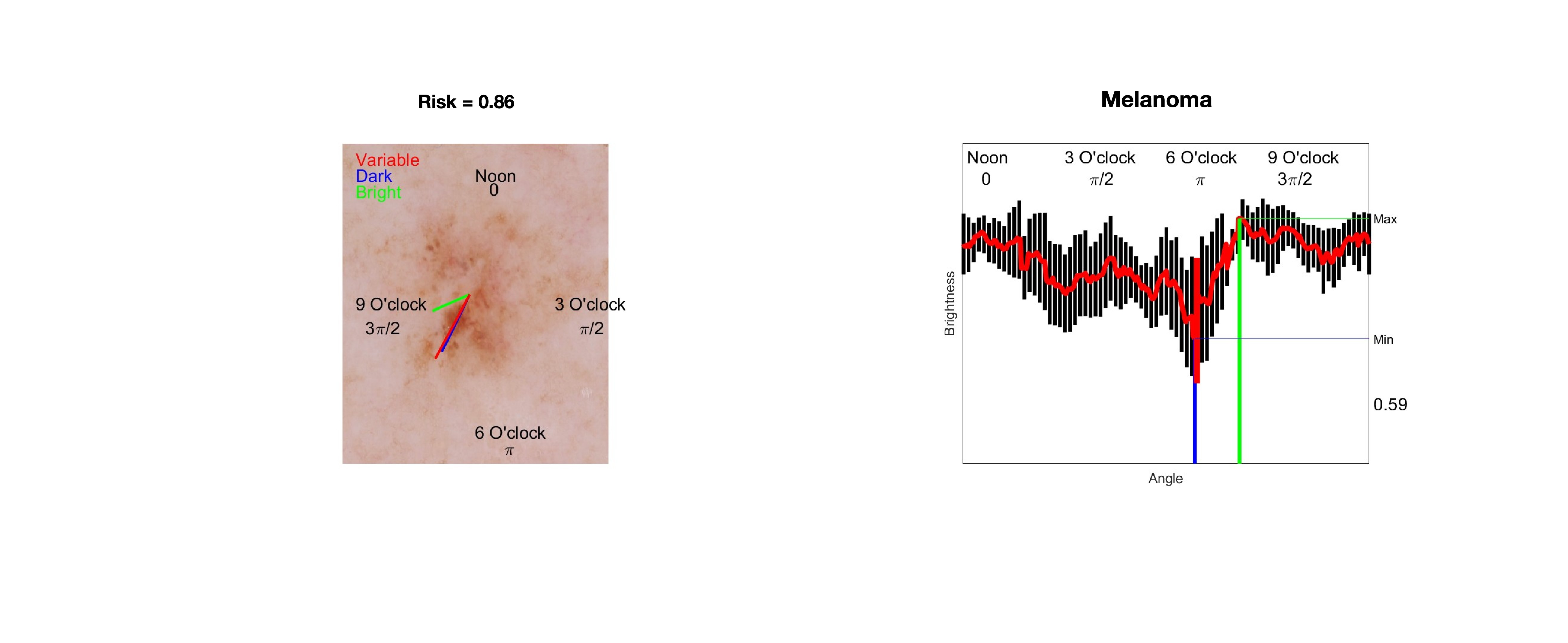

Supplement: Supplementary file 1 [file cancers-16-03077-s001.zip › cancers-3154863-supplementary/Supplementary File 2/011C.jpg]

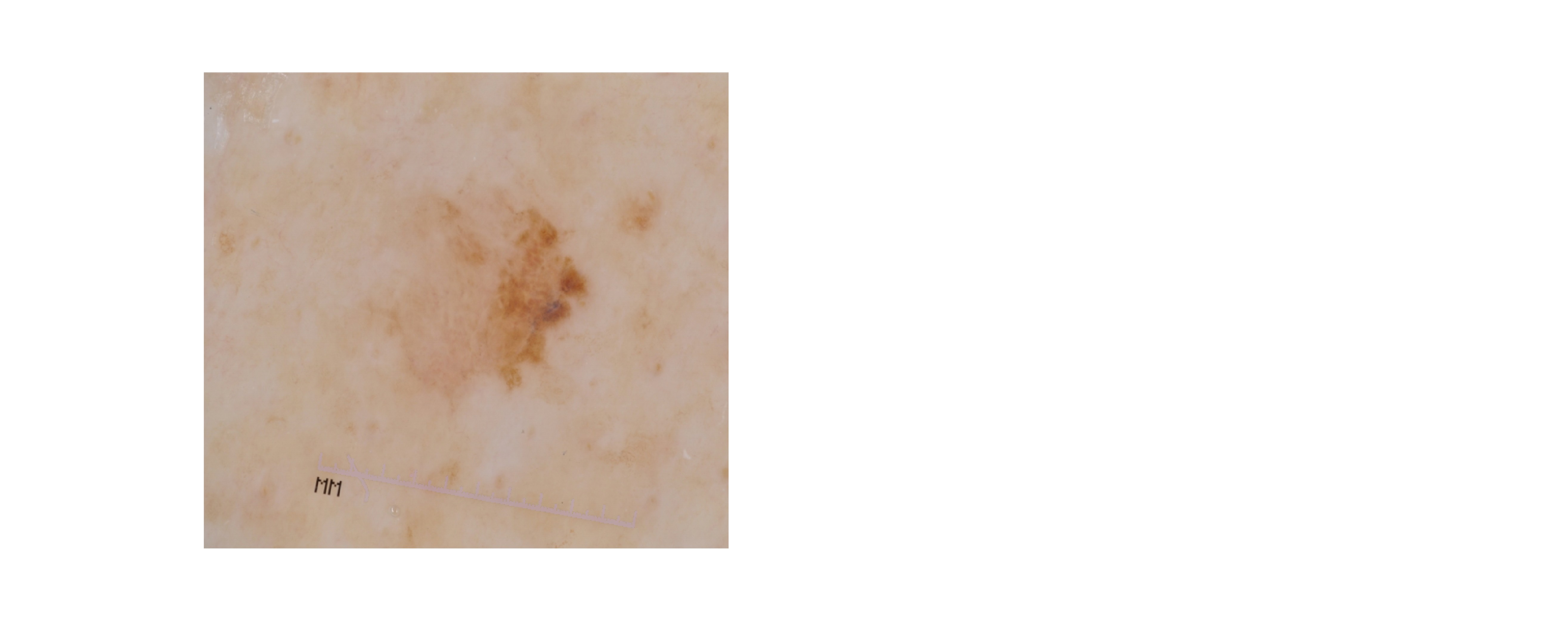

Supplement: Supplementary file 1 [file cancers-16-03077-s001.zip › cancers-3154863-supplementary/Supplementary File 2/012A.jpg]

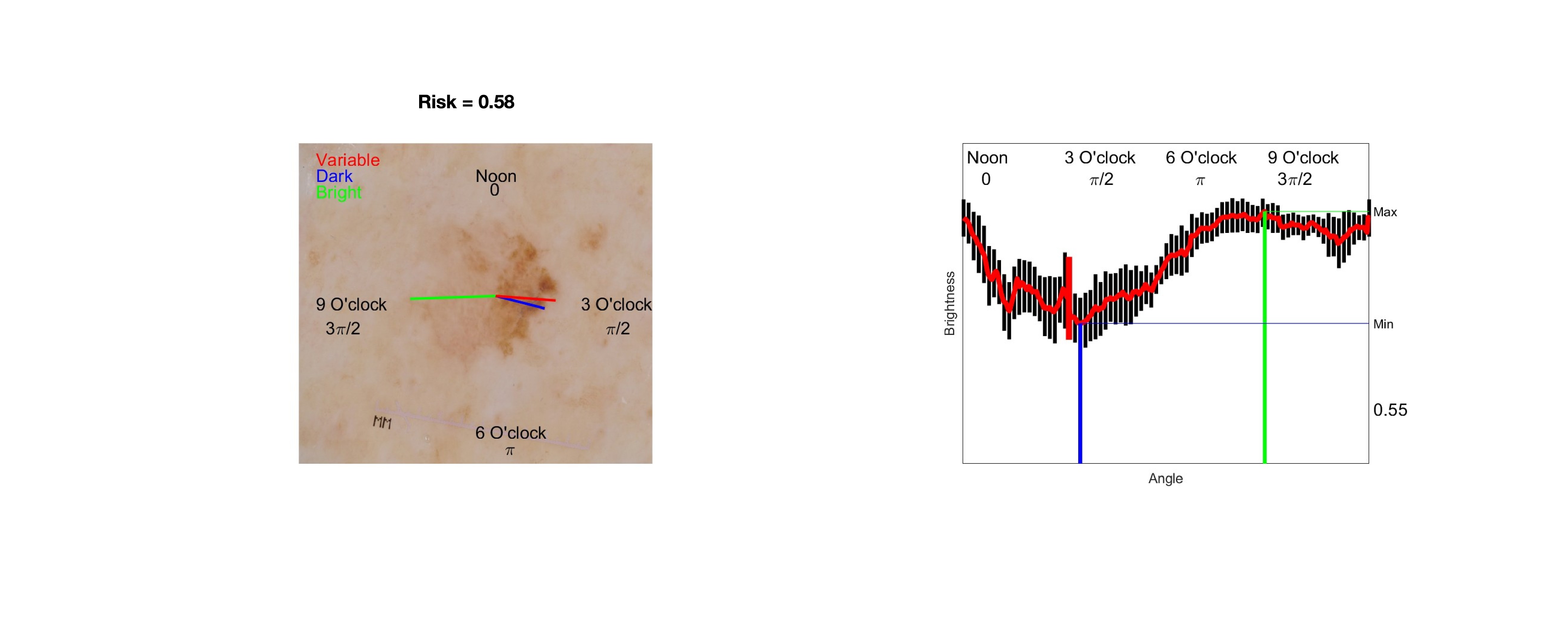

Supplement: Supplementary file 1 [file cancers-16-03077-s001.zip › cancers-3154863-supplementary/Supplementary File 2/012B.jpg]

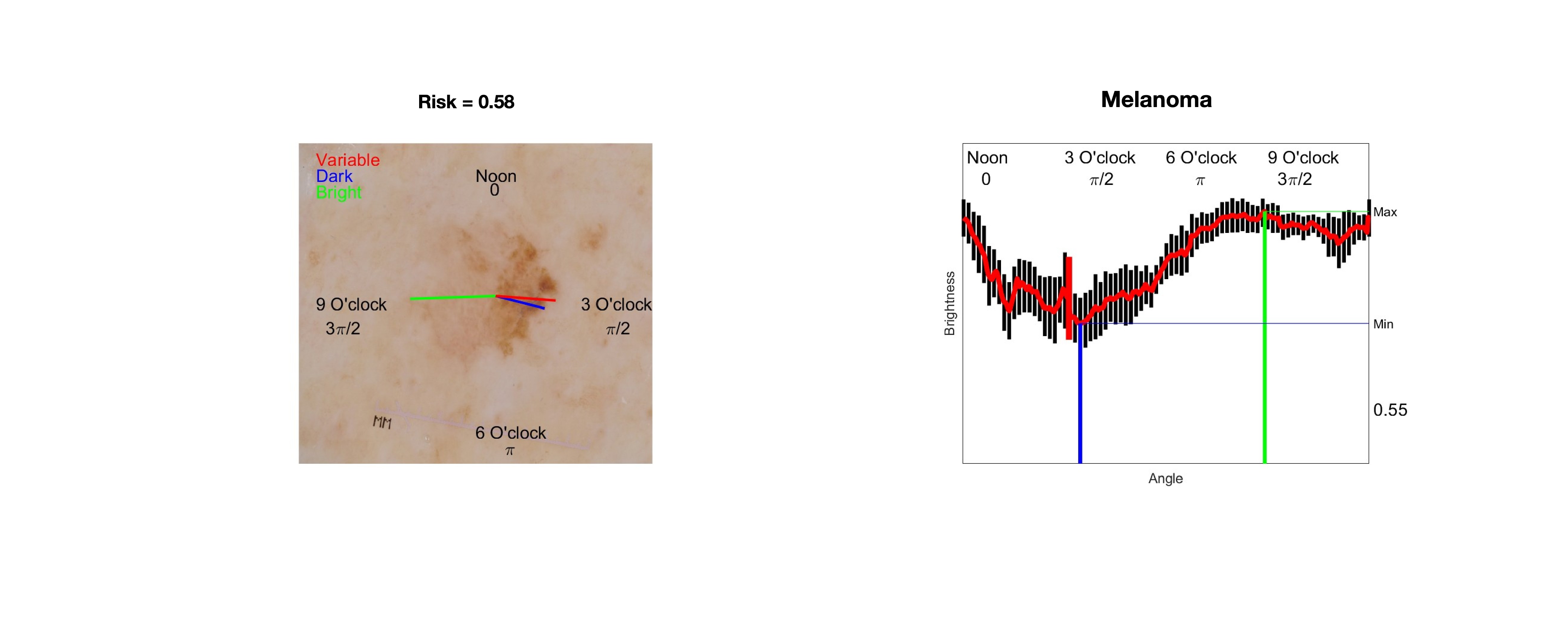

Supplement: Supplementary file 1 [file cancers-16-03077-s001.zip › cancers-3154863-supplementary/Supplementary File 2/012C.jpg]

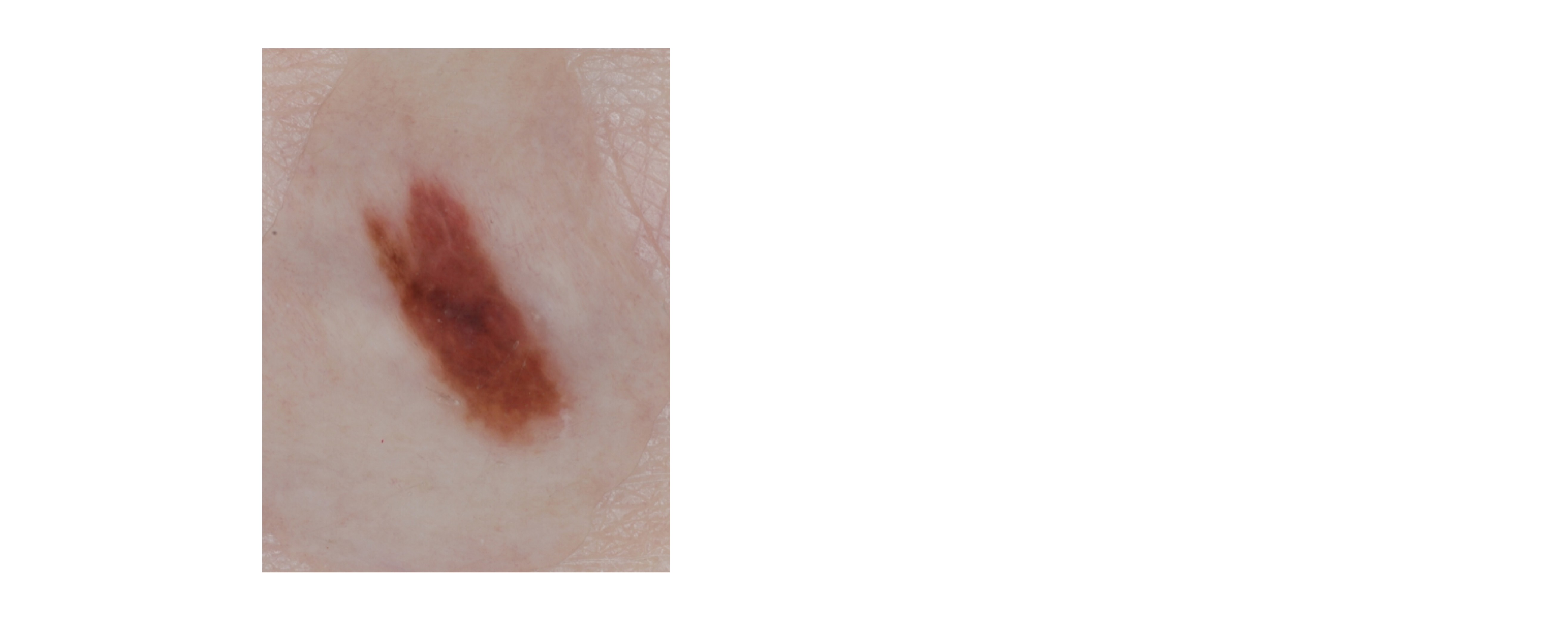

Supplement: Supplementary file 1 [file cancers-16-03077-s001.zip › cancers-3154863-supplementary/Supplementary File 2/013A.jpg]

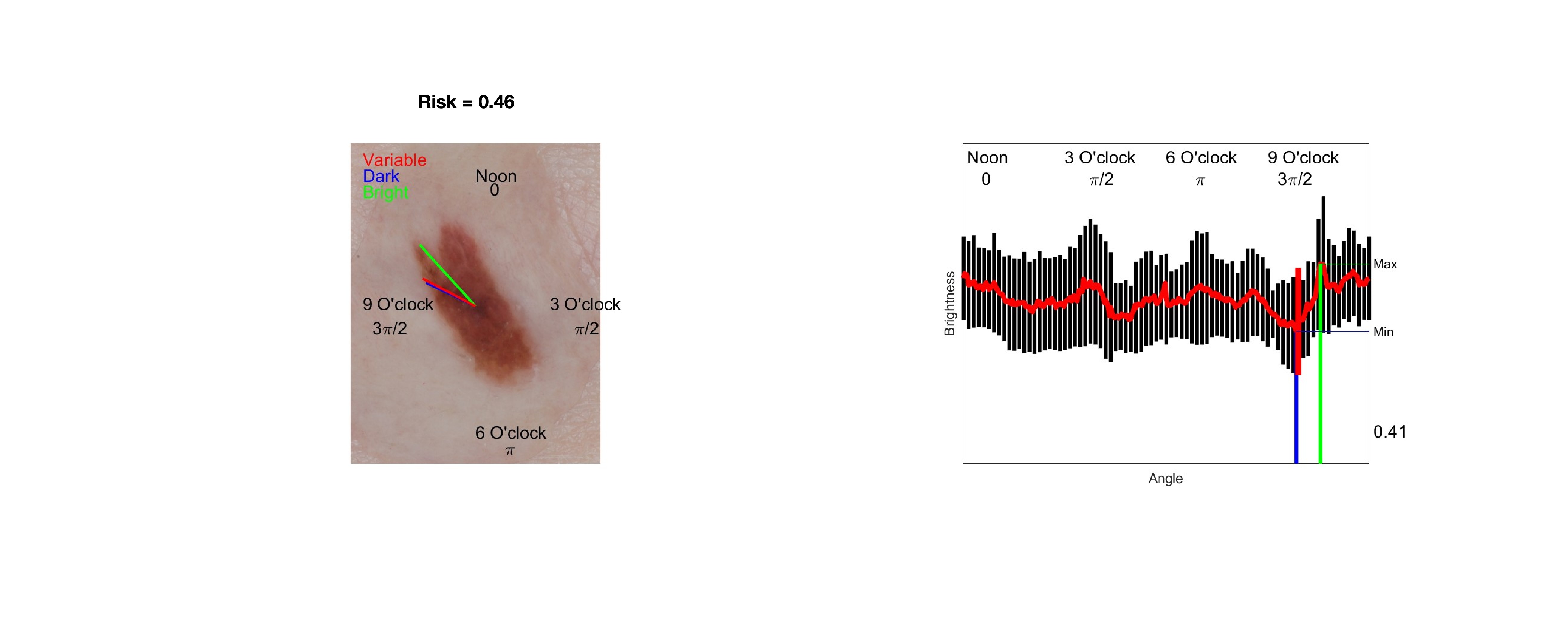

Supplement: Supplementary file 1 [file cancers-16-03077-s001.zip › cancers-3154863-supplementary/Supplementary File 2/013B.jpg]

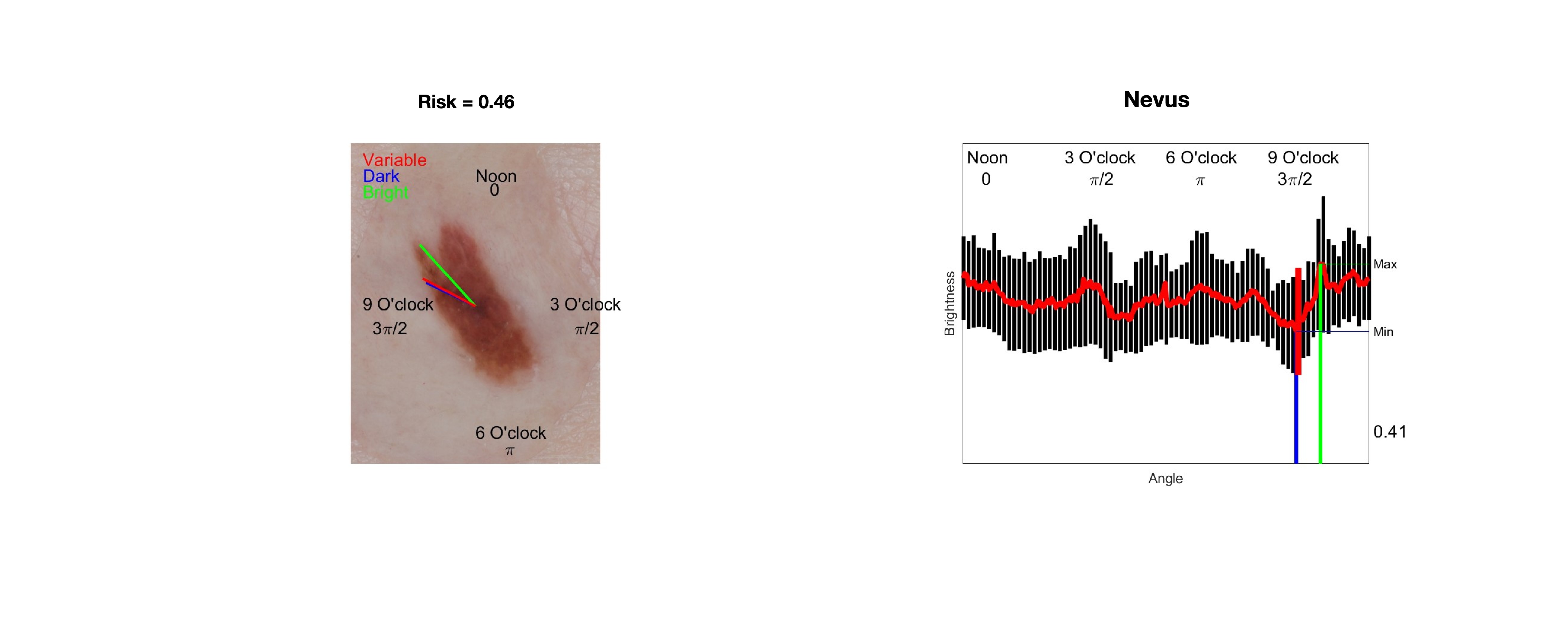

Supplement: Supplementary file 1 [file cancers-16-03077-s001.zip › cancers-3154863-supplementary/Supplementary File 2/013C.jpg]

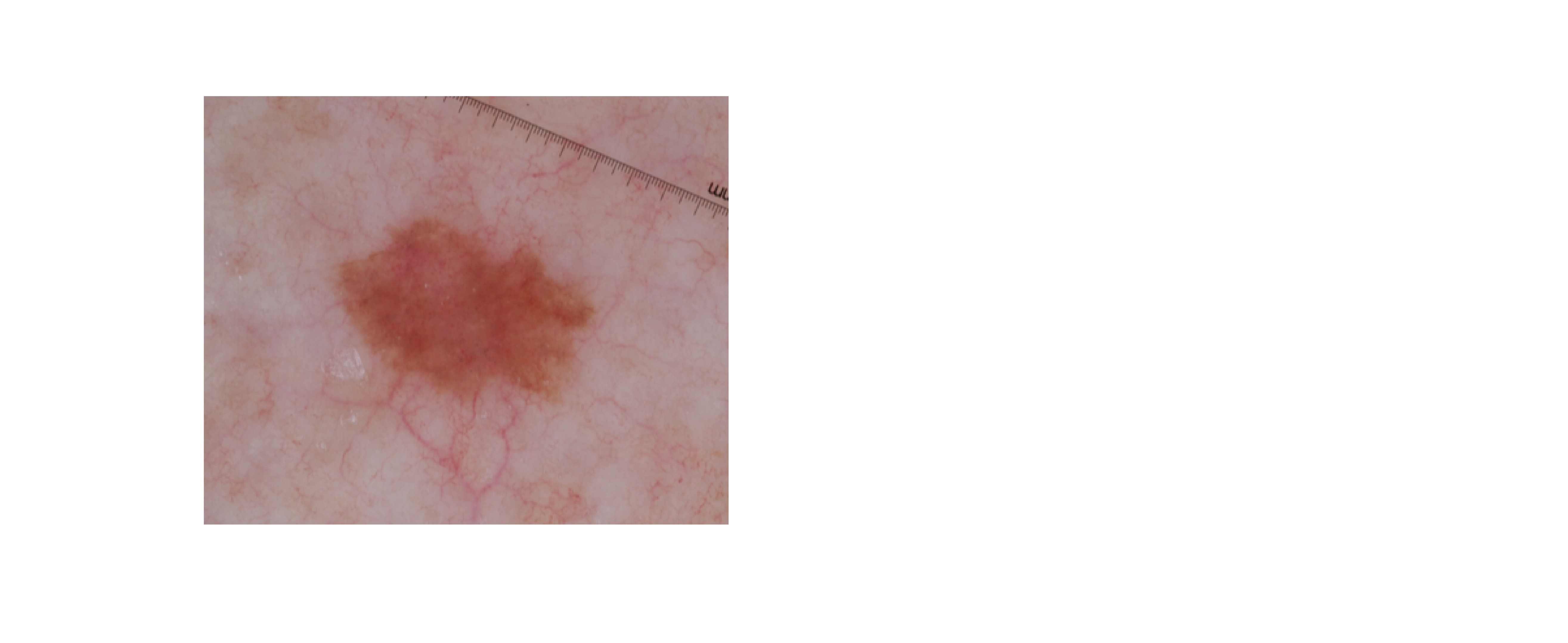

Supplement: Supplementary file 1 [file cancers-16-03077-s001.zip › cancers-3154863-supplementary/Supplementary File 2/014A.jpg]

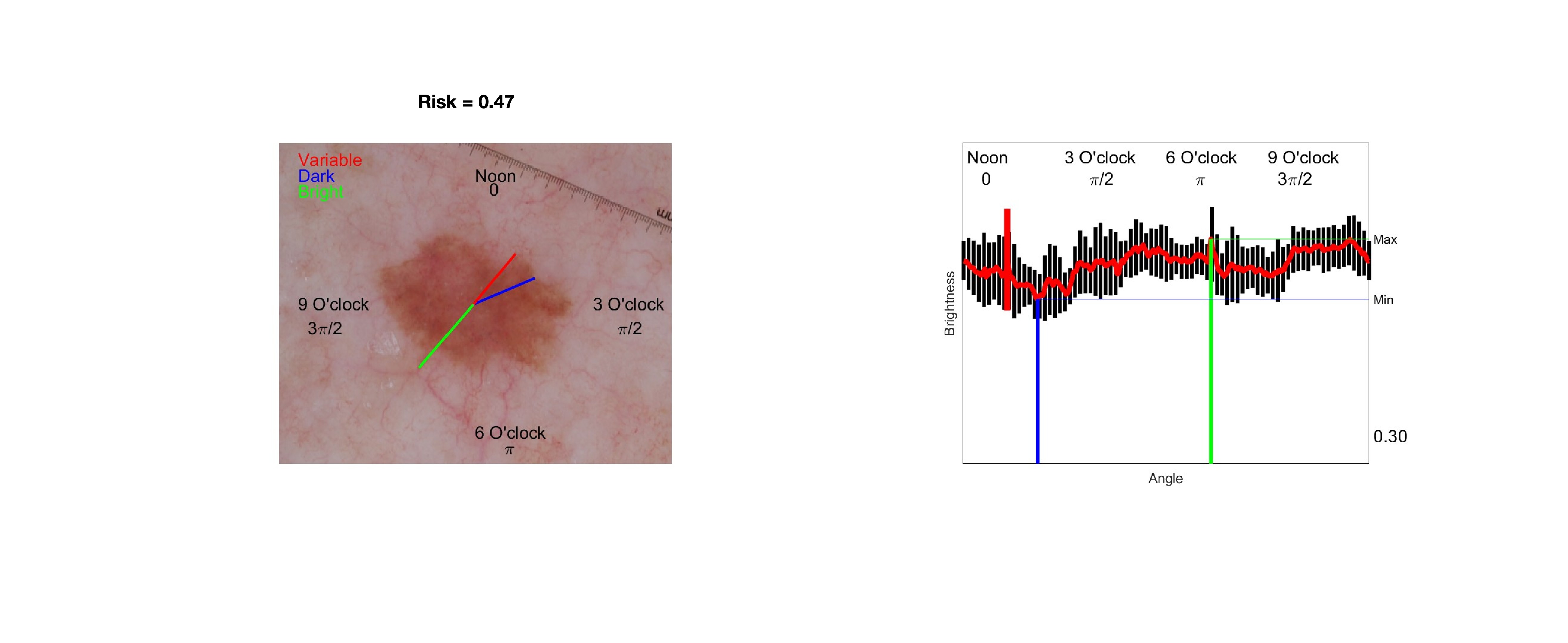

Supplement: Supplementary file 1 [file cancers-16-03077-s001.zip › cancers-3154863-supplementary/Supplementary File 2/014B.jpg]

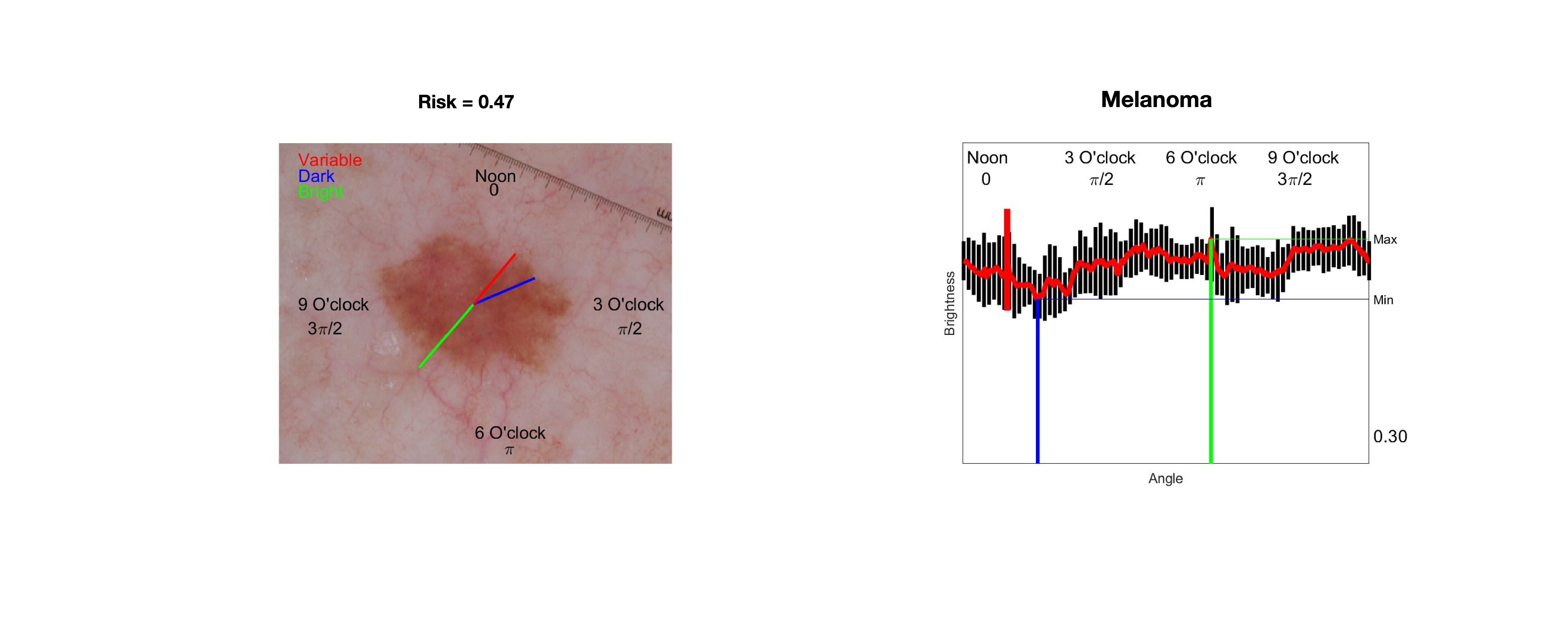

Supplement: Supplementary file 1 [file cancers-16-03077-s001.zip › cancers-3154863-supplementary/Supplementary File 2/014C.jpg]

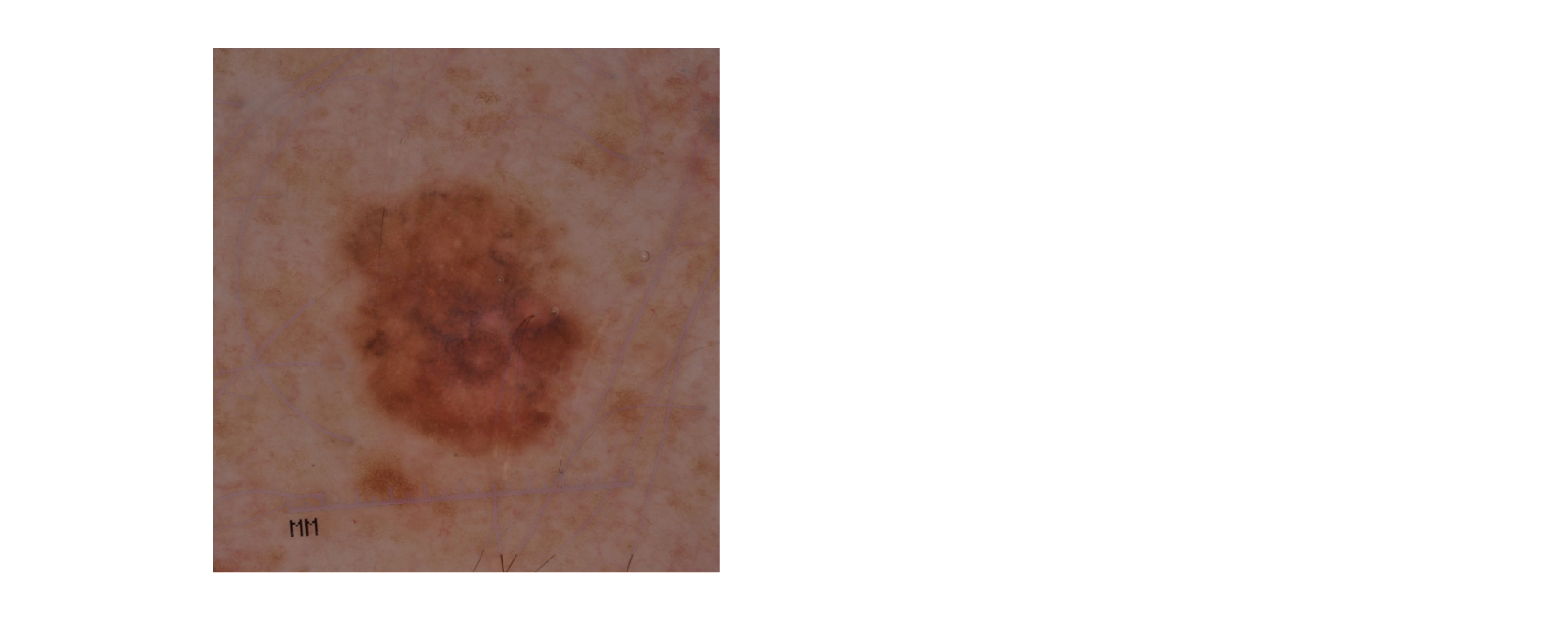

Supplement: Supplementary file 1 [file cancers-16-03077-s001.zip › cancers-3154863-supplementary/Supplementary File 2/015A.jpg]

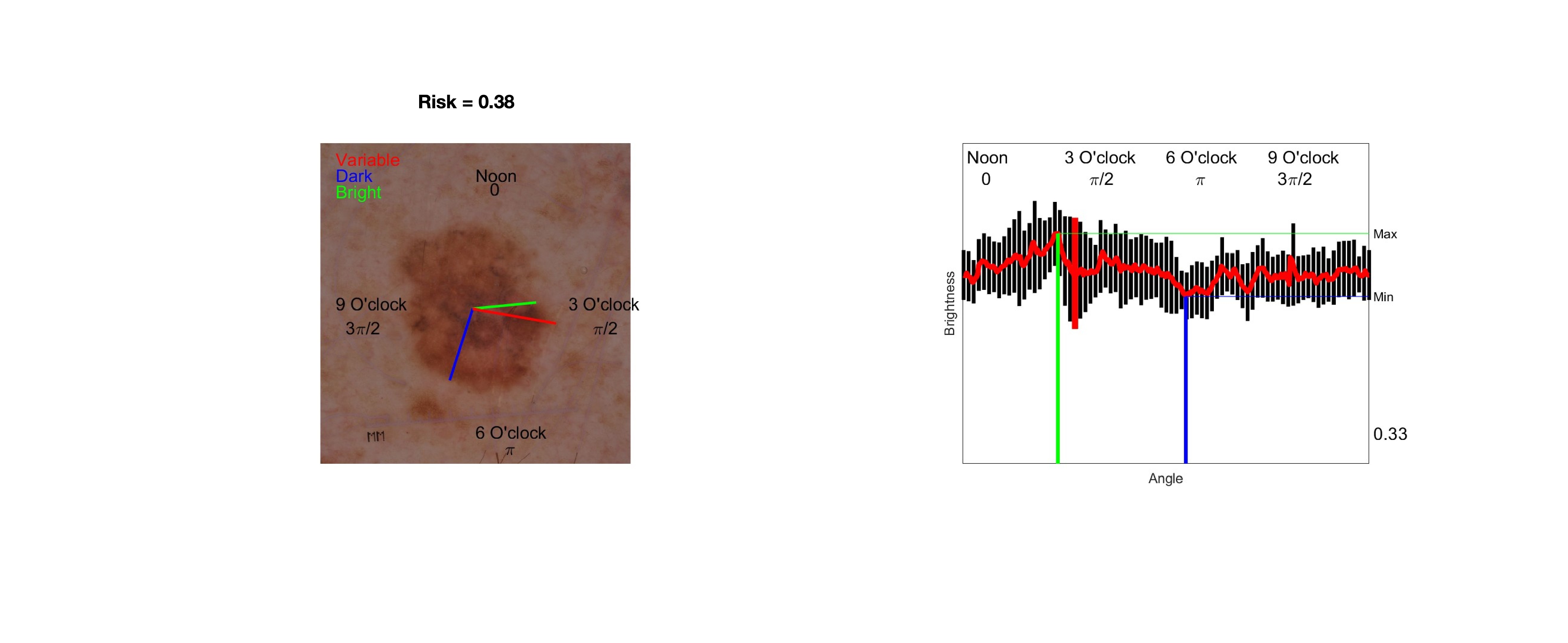

Supplement: Supplementary file 1 [file cancers-16-03077-s001.zip › cancers-3154863-supplementary/Supplementary File 2/015B.jpg]

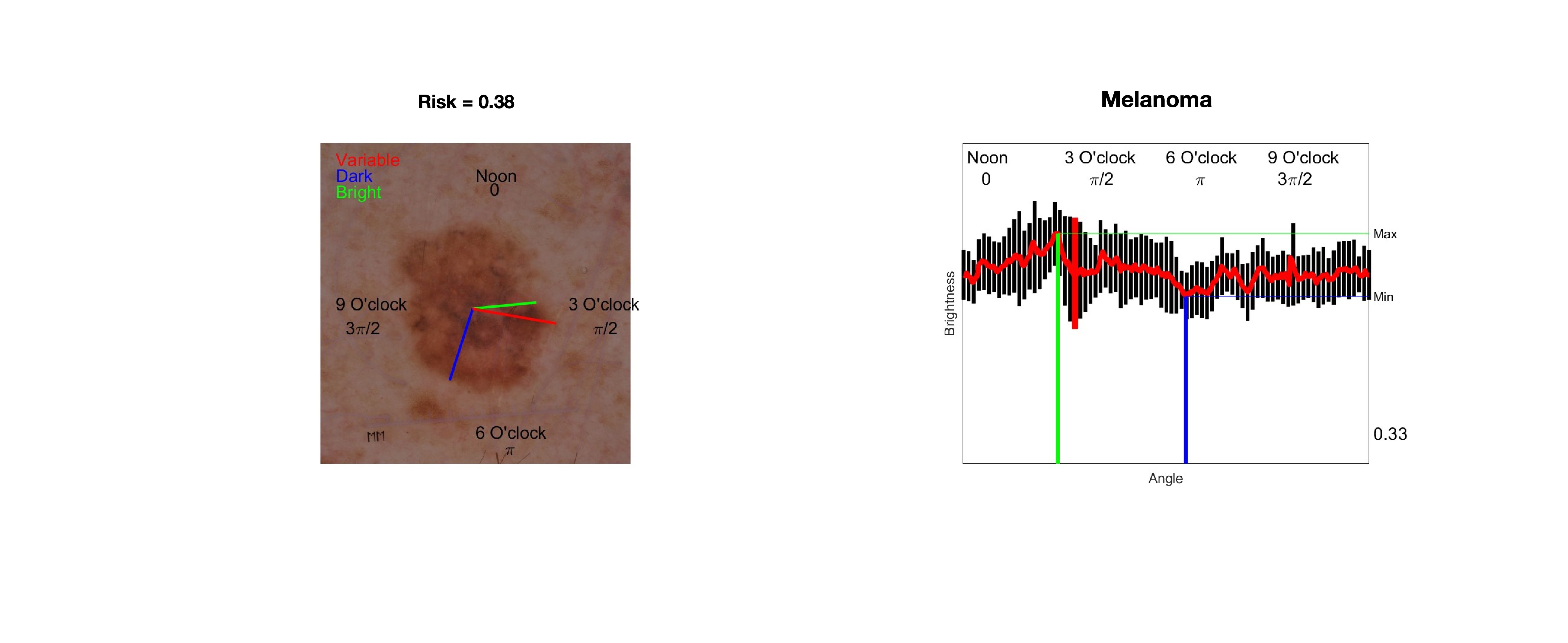

Supplement: Supplementary file 1 [file cancers-16-03077-s001.zip › cancers-3154863-supplementary/Supplementary File 2/015C.jpg]

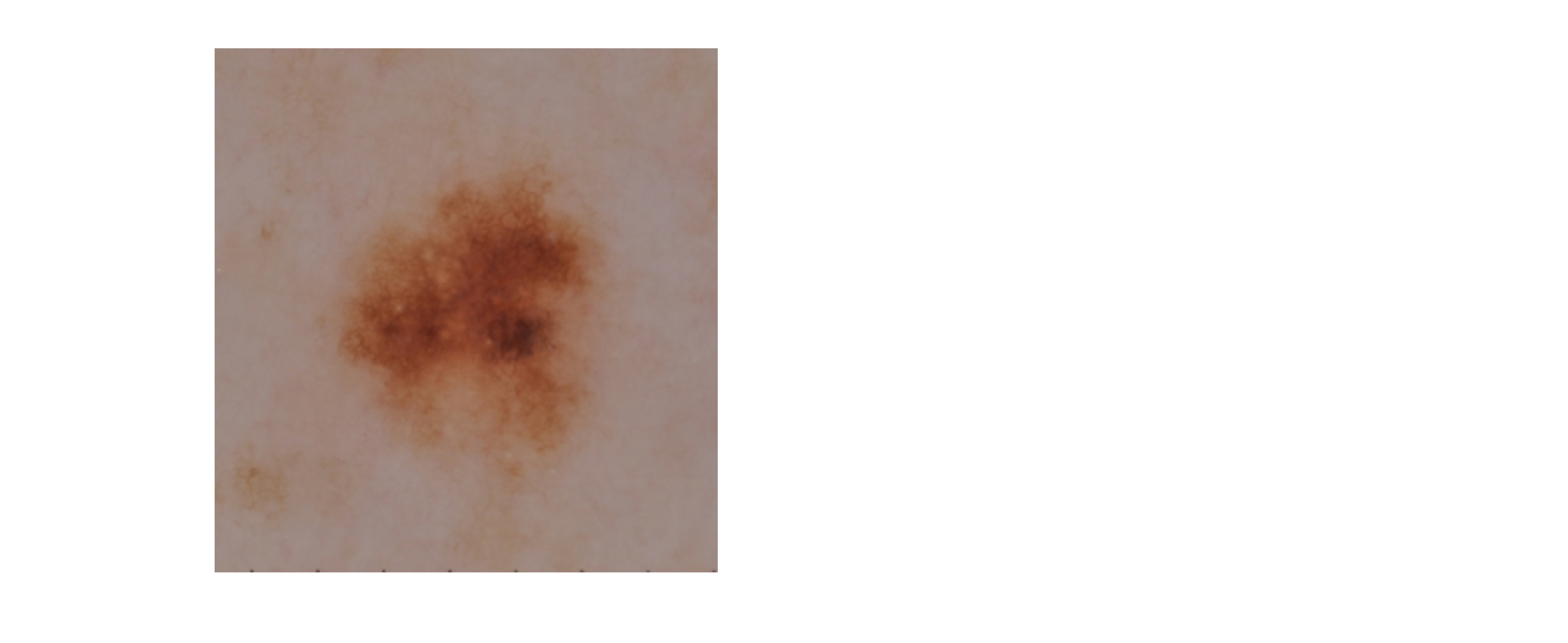

Supplement: Supplementary file 1 [file cancers-16-03077-s001.zip › cancers-3154863-supplementary/Supplementary File 2/016A.jpg]

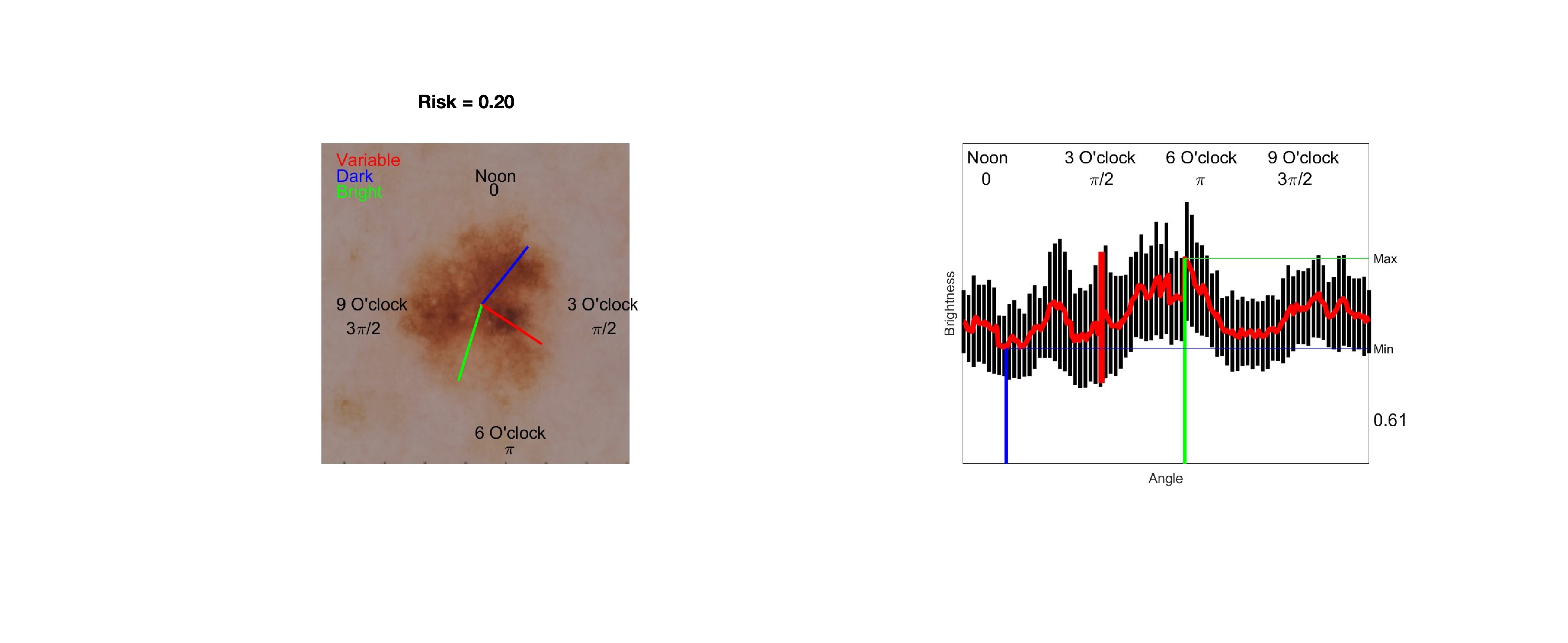

Supplement: Supplementary file 1 [file cancers-16-03077-s001.zip › cancers-3154863-supplementary/Supplementary File 2/016B.jpg]

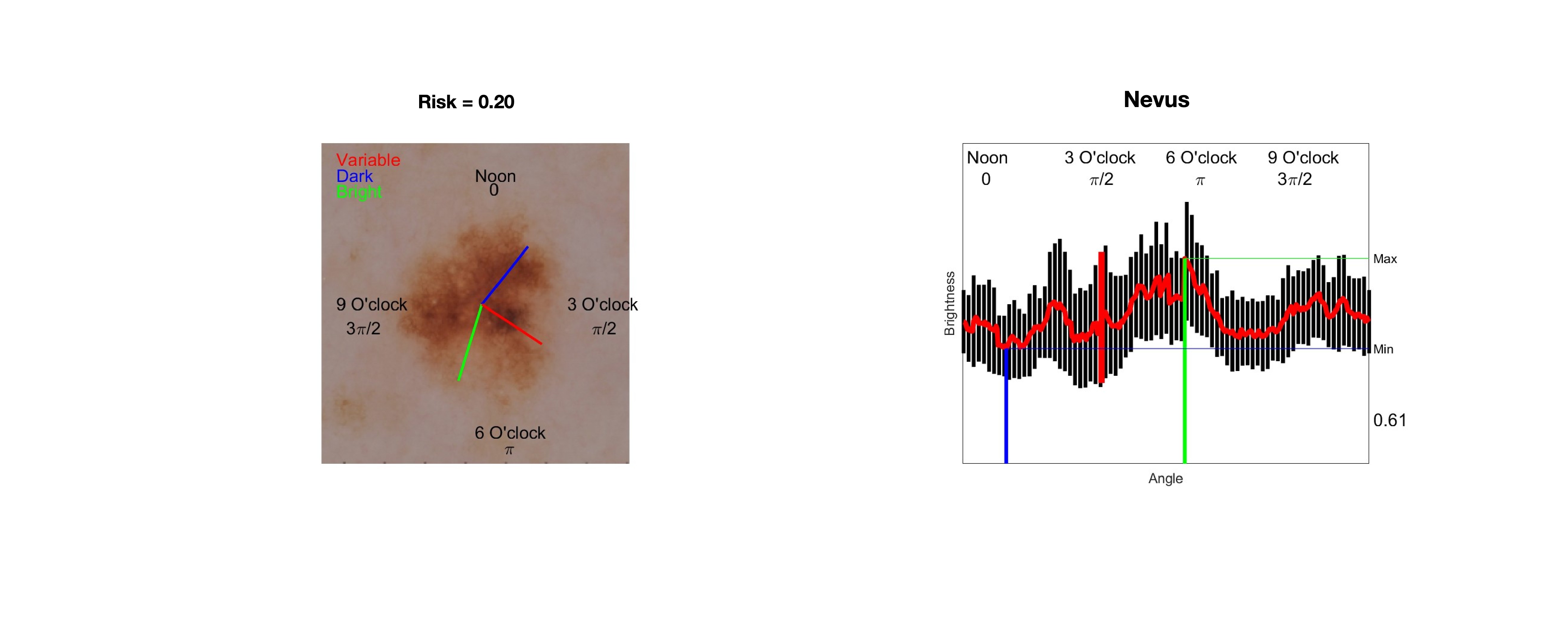

Supplement: Supplementary file 1 [file cancers-16-03077-s001.zip › cancers-3154863-supplementary/Supplementary File 2/016C.jpg]

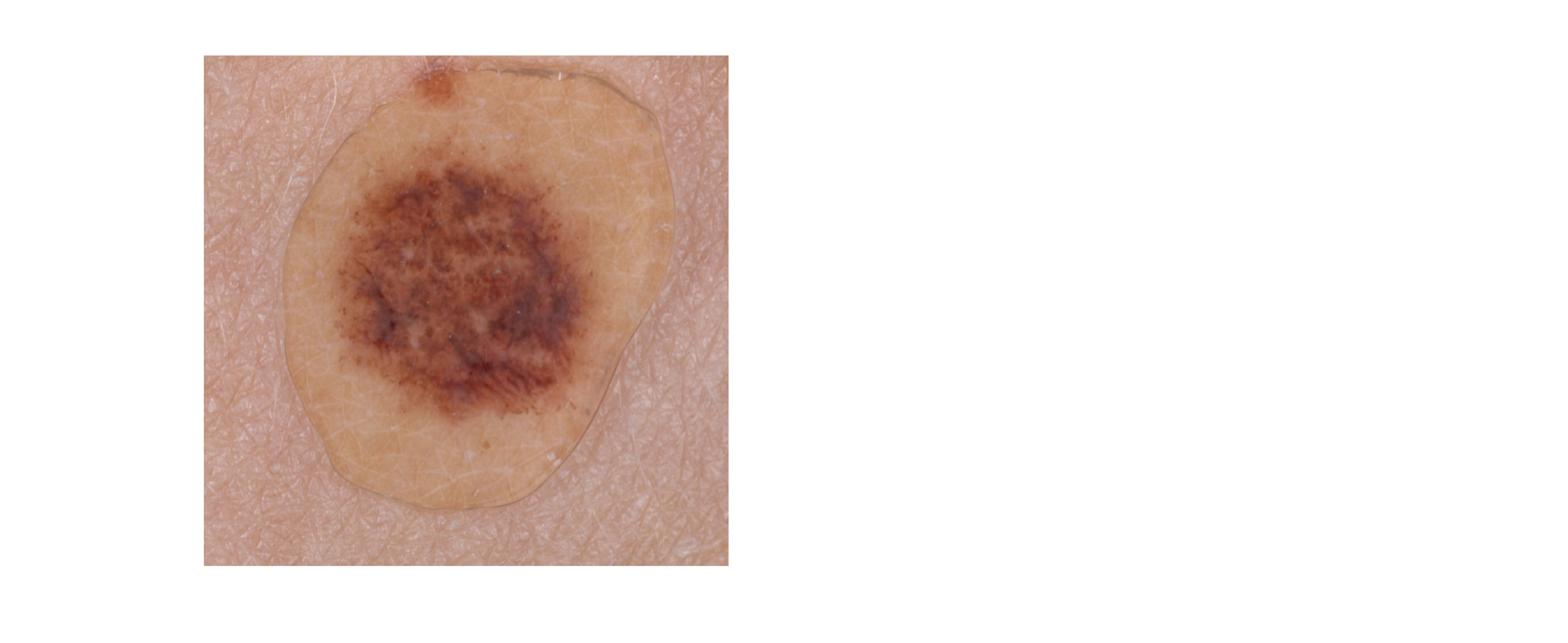

Supplement: Supplementary file 1 [file cancers-16-03077-s001.zip › cancers-3154863-supplementary/Supplementary File 2/017A.jpg]

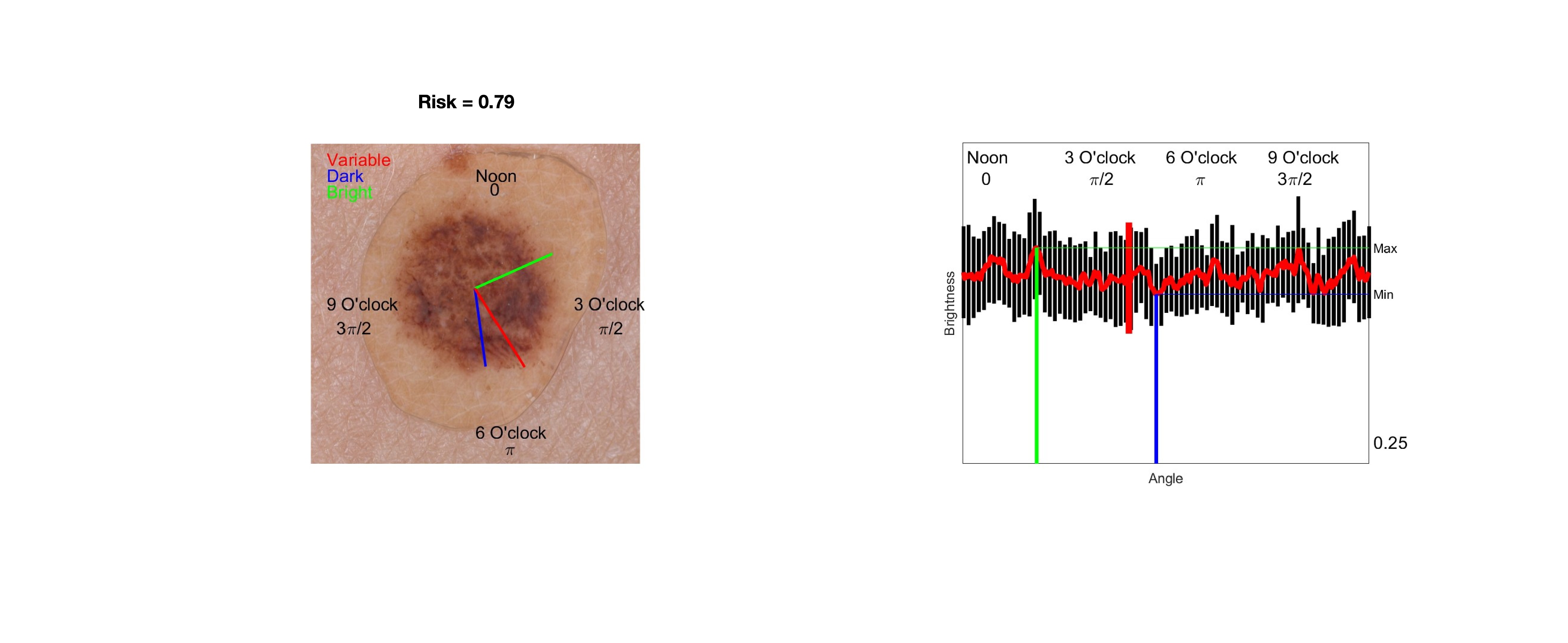

Supplement: Supplementary file 1 [file cancers-16-03077-s001.zip › cancers-3154863-supplementary/Supplementary File 2/017B.jpg]

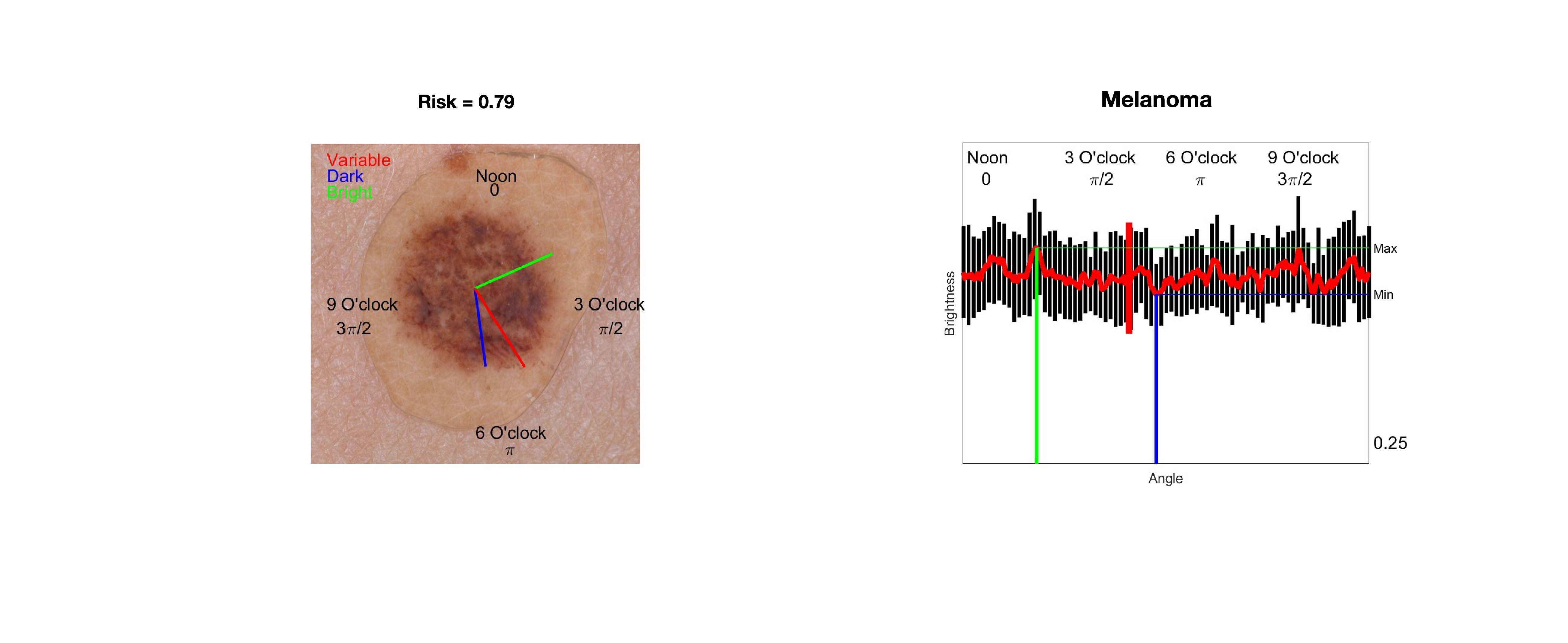

Supplement: Supplementary file 1 [file cancers-16-03077-s001.zip › cancers-3154863-supplementary/Supplementary File 2/017C.jpg]

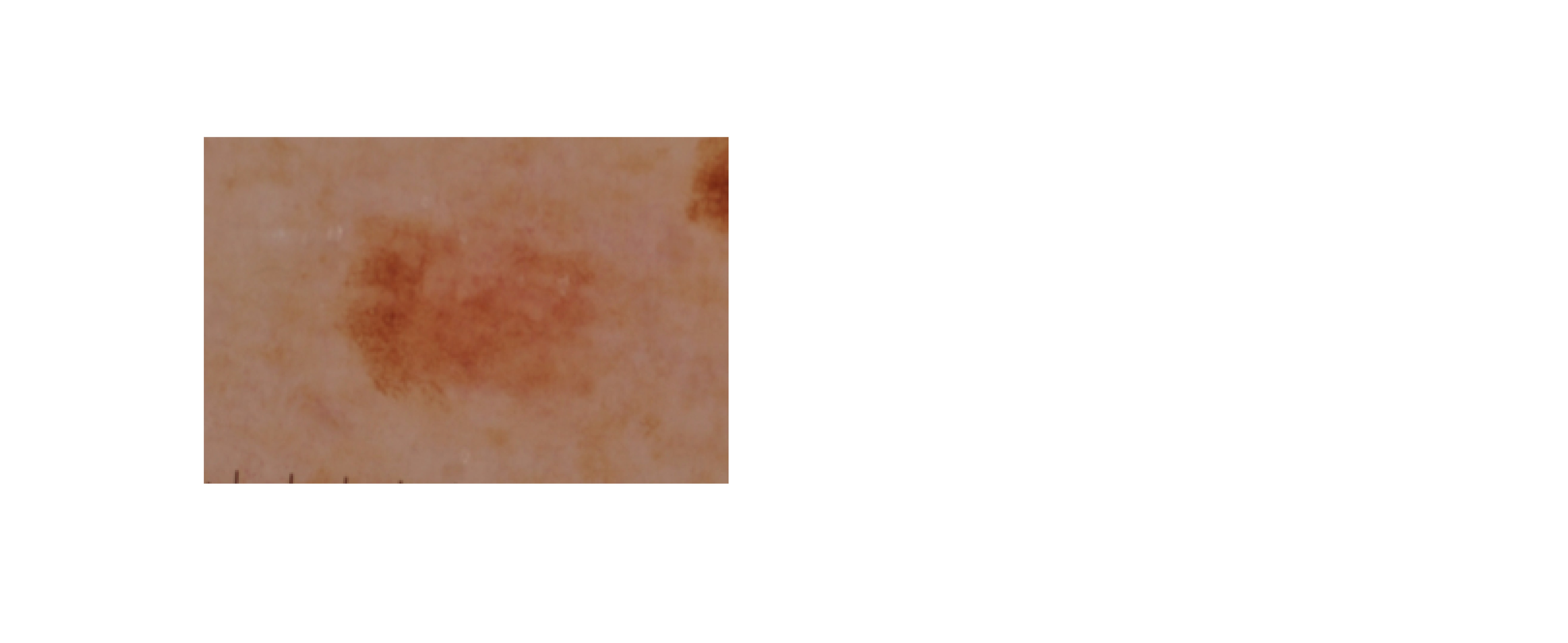

Supplement: Supplementary file 1 [file cancers-16-03077-s001.zip › cancers-3154863-supplementary/Supplementary File 2/018A.jpg]

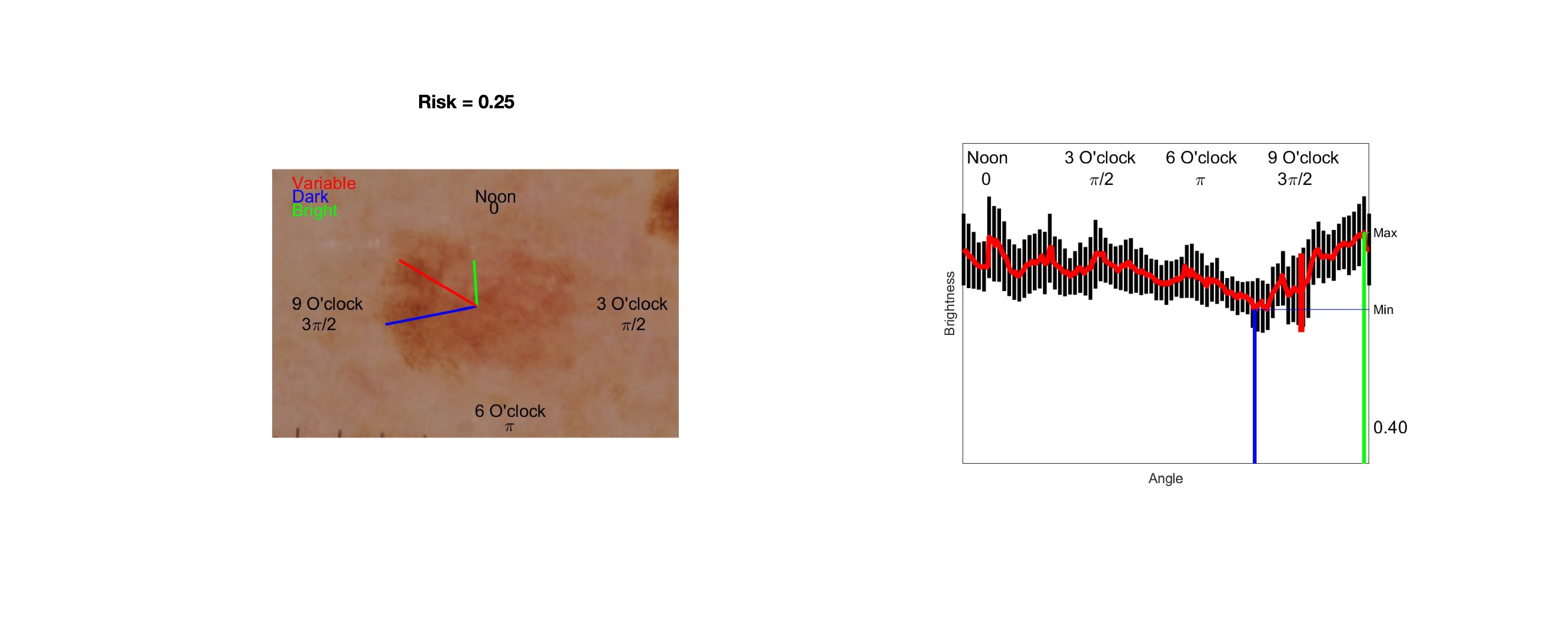

Supplement: Supplementary file 1 [file cancers-16-03077-s001.zip › cancers-3154863-supplementary/Supplementary File 2/018B.jpg]

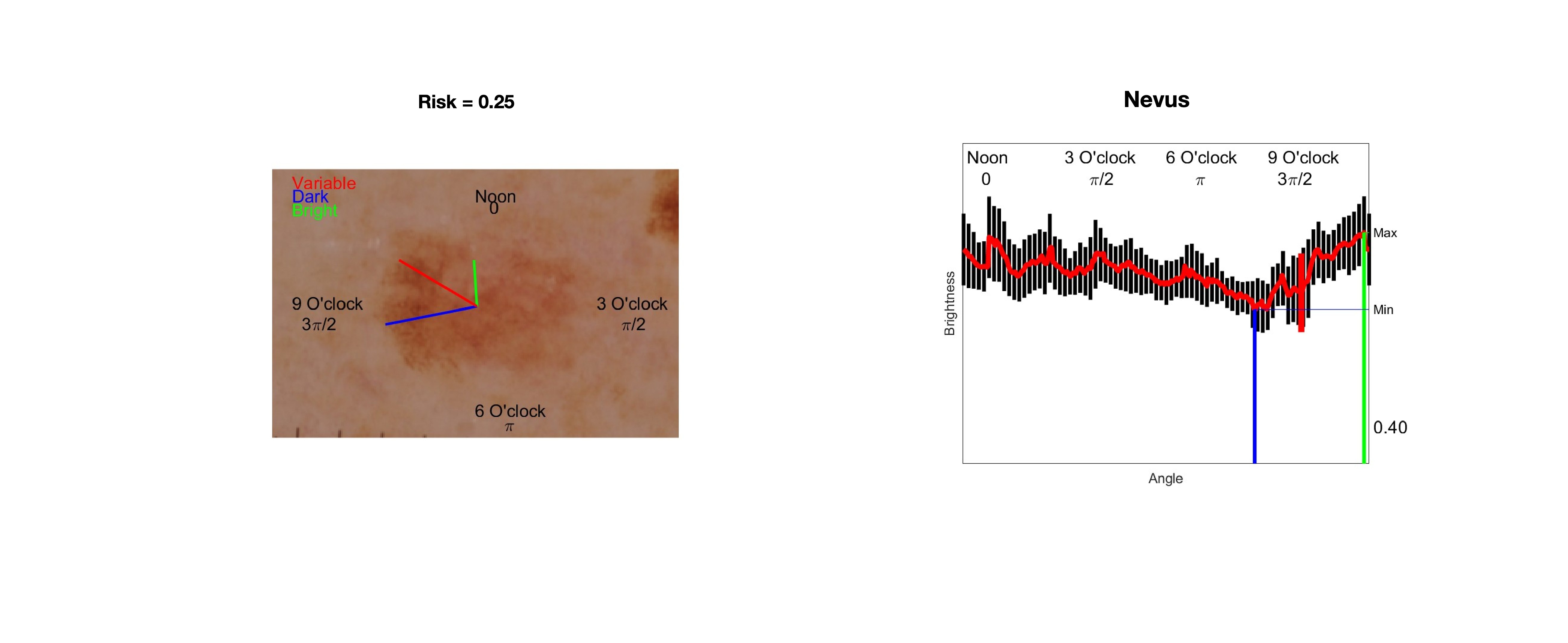

Supplement: Supplementary file 1 [file cancers-16-03077-s001.zip › cancers-3154863-supplementary/Supplementary File 2/018C.jpg]

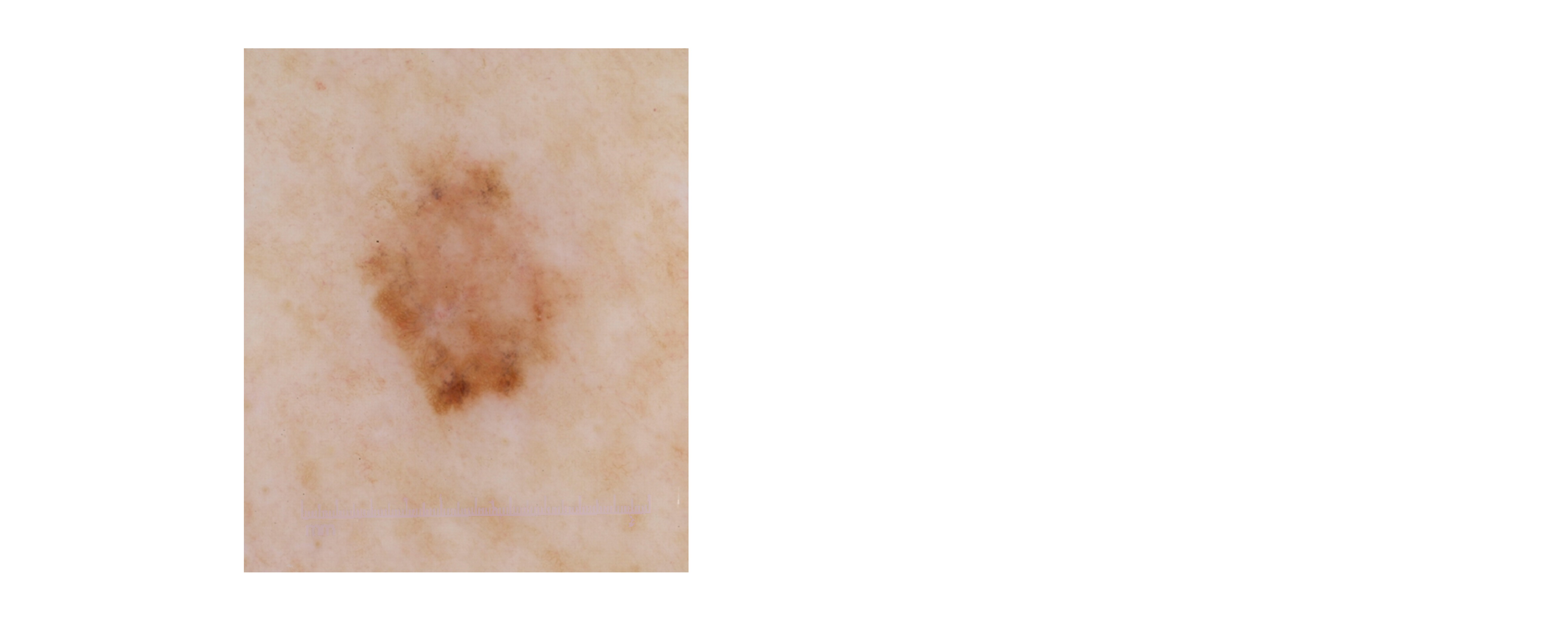

Supplement: Supplementary file 1 [file cancers-16-03077-s001.zip › cancers-3154863-supplementary/Supplementary File 2/019A.jpg]

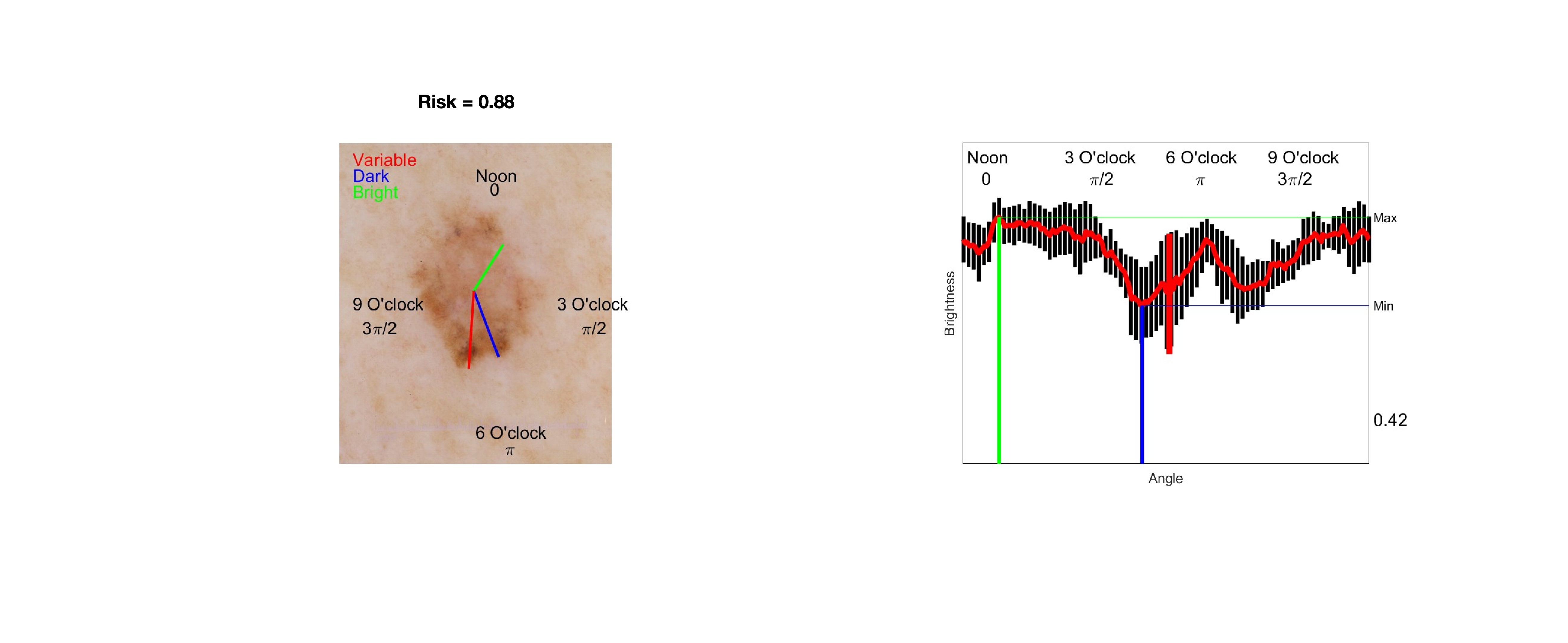

Supplement: Supplementary file 1 [file cancers-16-03077-s001.zip › cancers-3154863-supplementary/Supplementary File 2/019B.jpg]

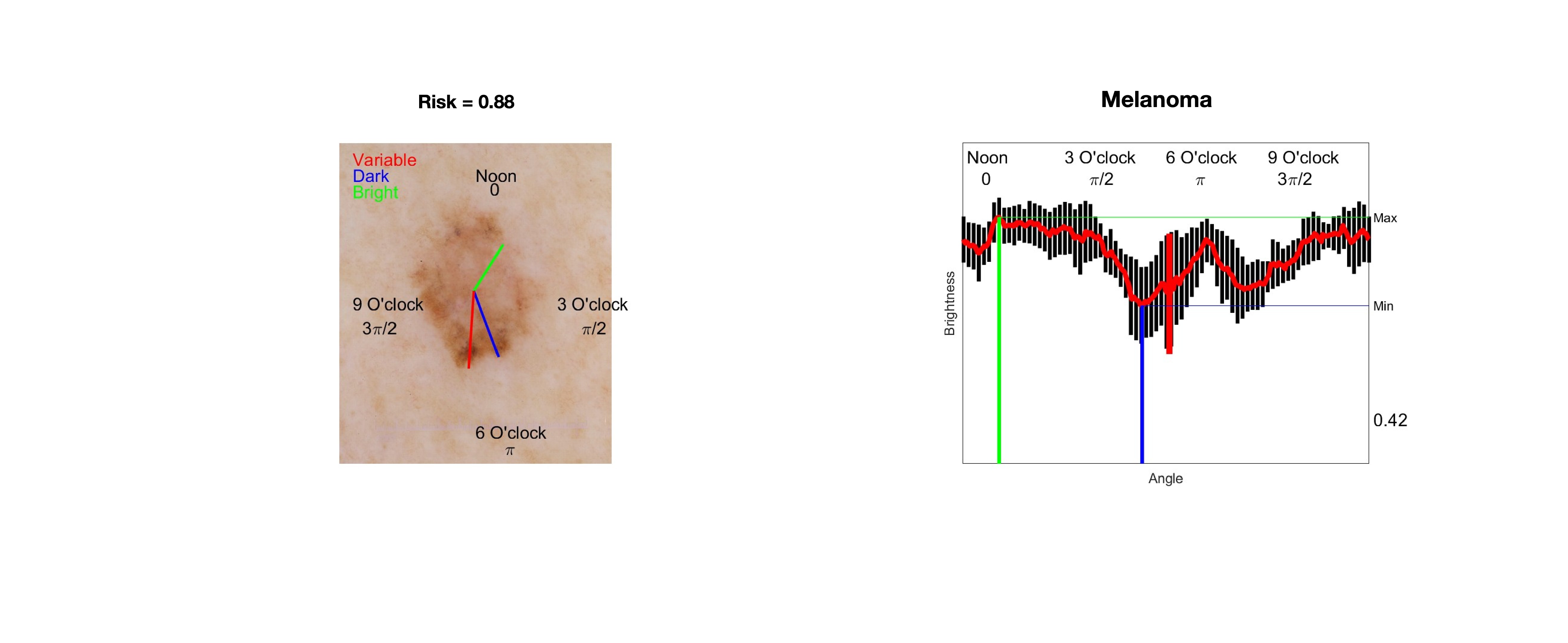

Supplement: Supplementary file 1 [file cancers-16-03077-s001.zip › cancers-3154863-supplementary/Supplementary File 2/019C.jpg]

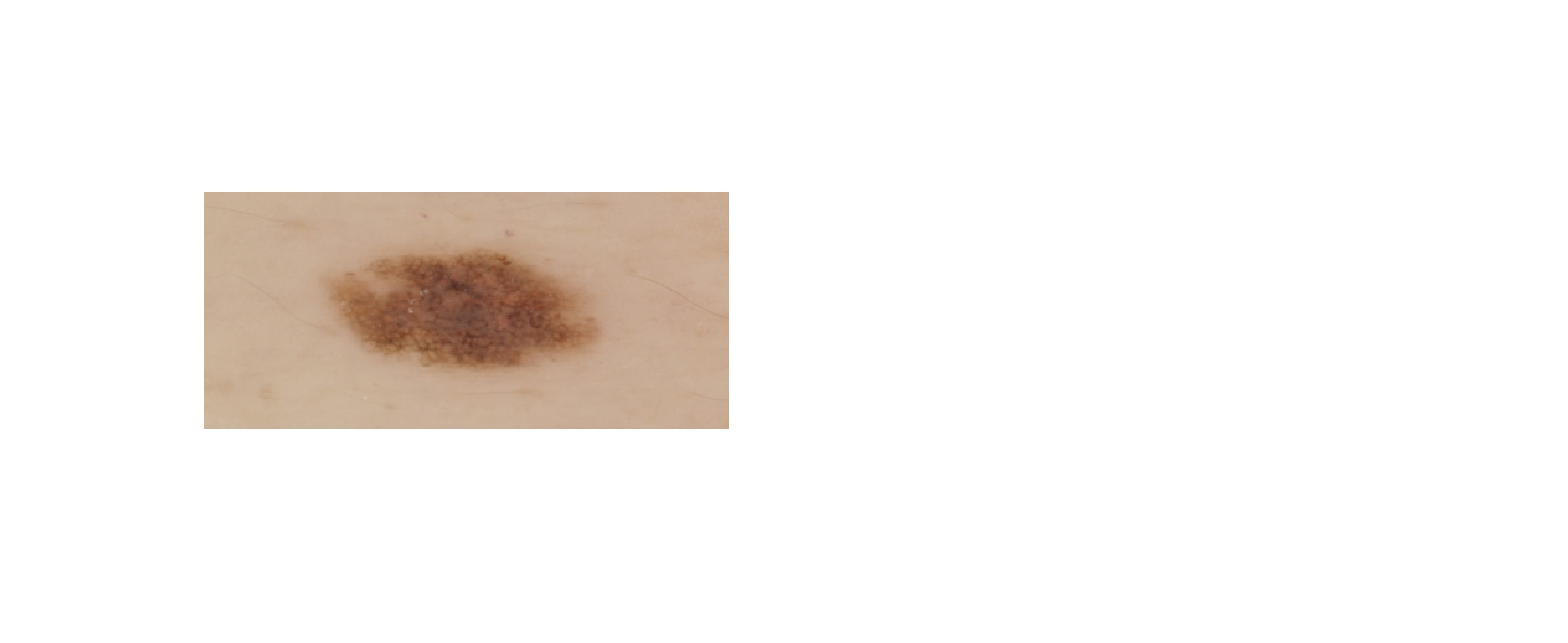

Supplement: Supplementary file 1 [file cancers-16-03077-s001.zip › cancers-3154863-supplementary/Supplementary File 2/020A.jpg]

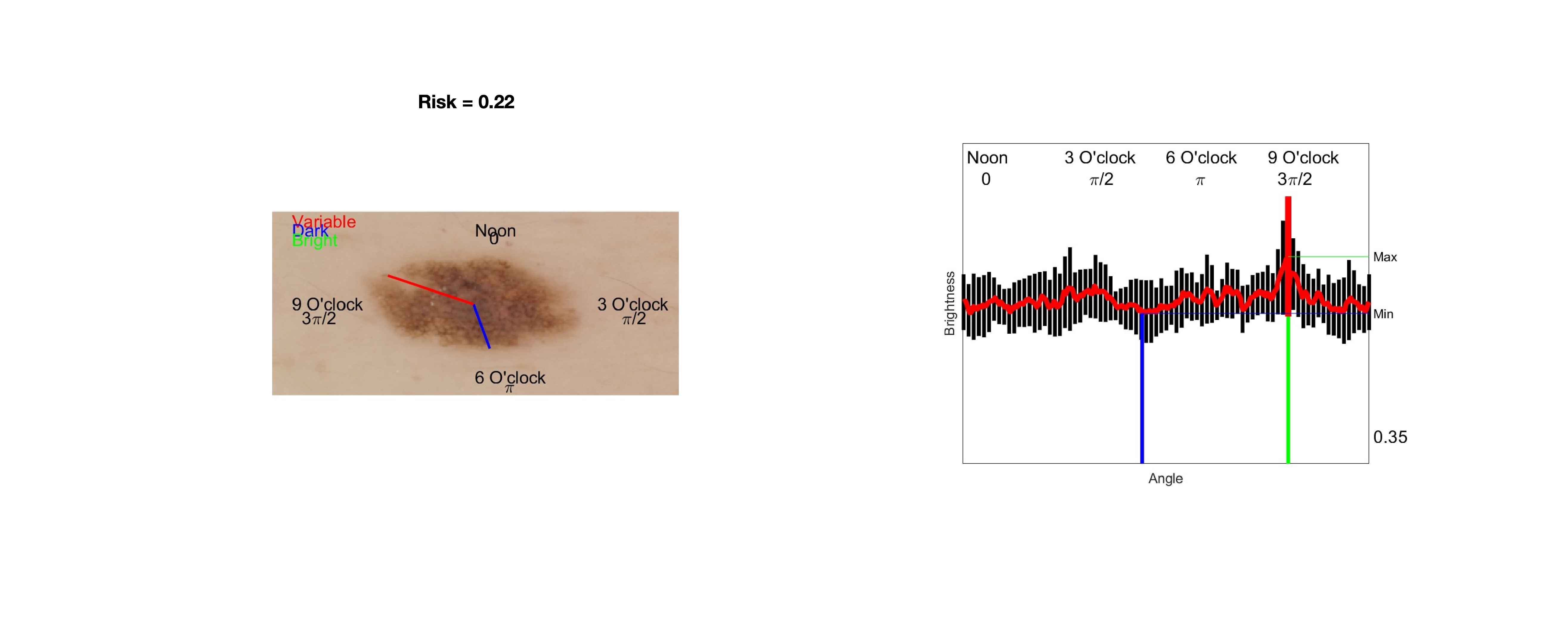

Supplement: Supplementary file 1 [file cancers-16-03077-s001.zip › cancers-3154863-supplementary/Supplementary File 2/020B.jpg]

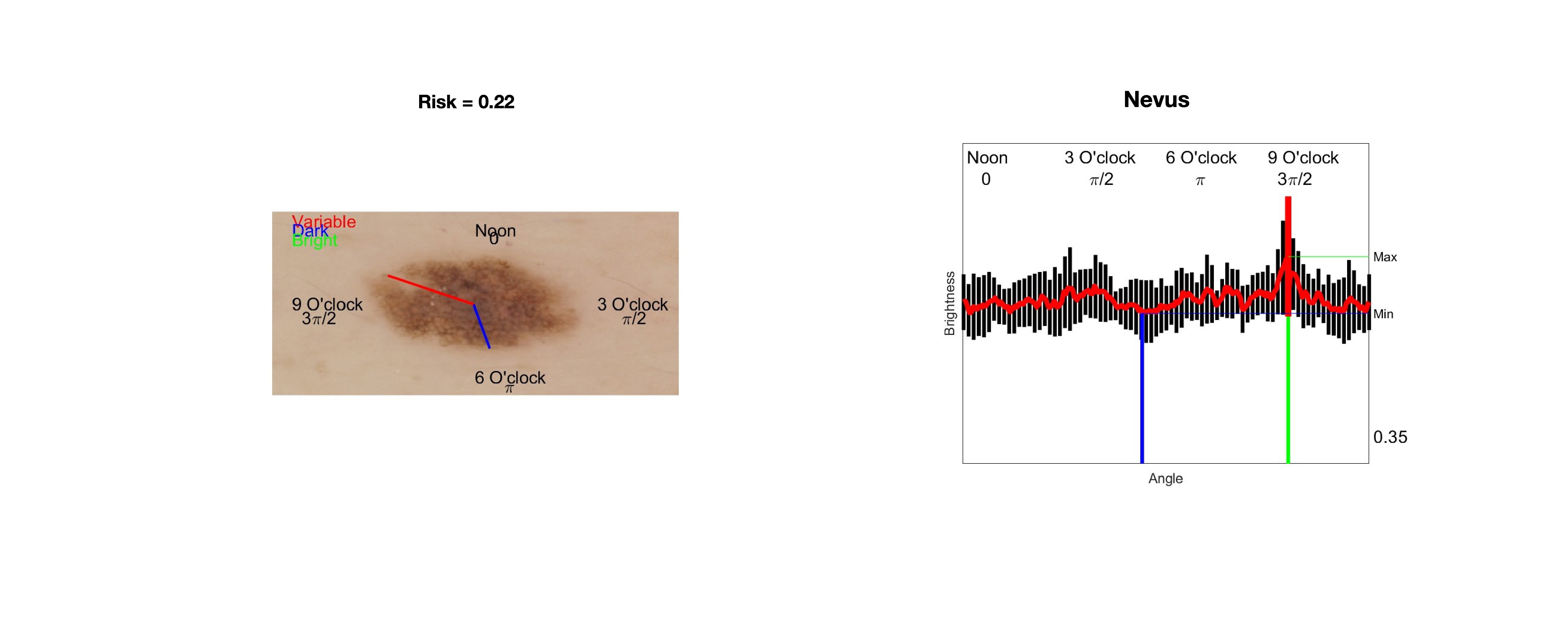

Supplement: Supplementary file 1 [file cancers-16-03077-s001.zip › cancers-3154863-supplementary/Supplementary File 2/020C.jpg]

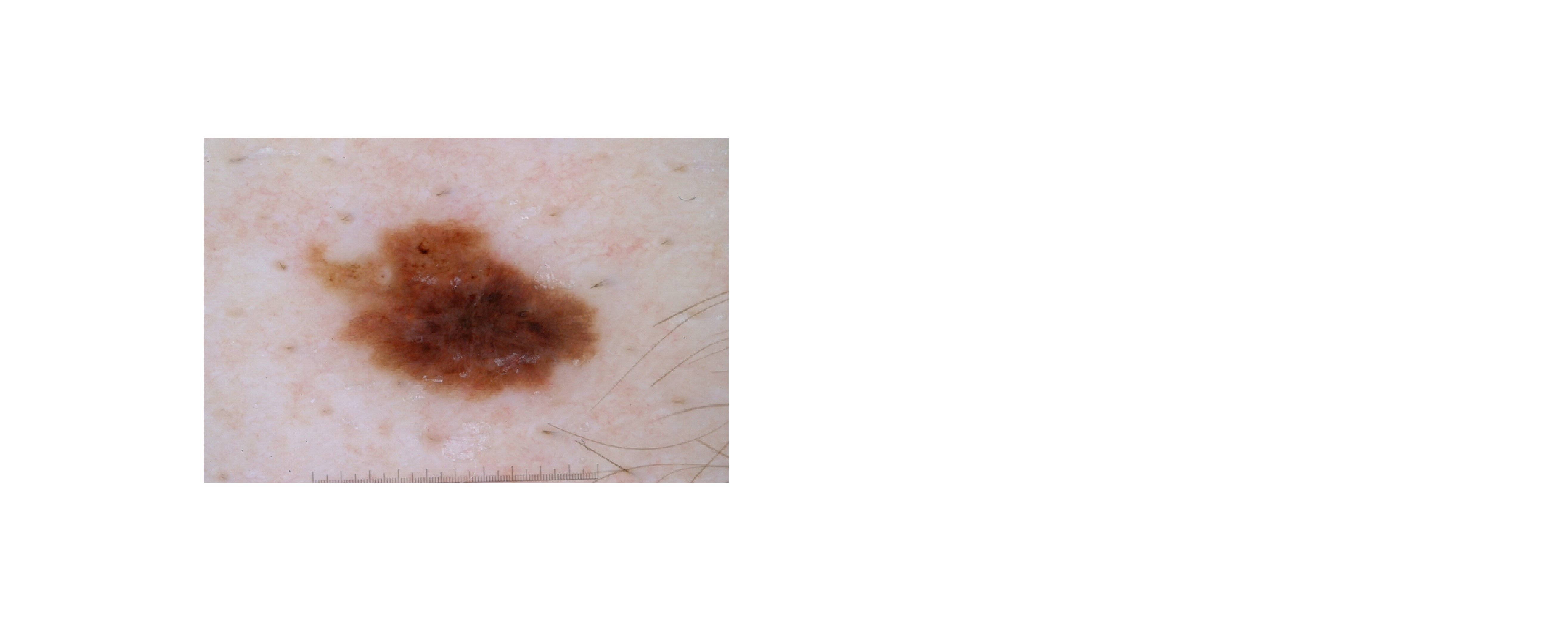

Supplement: Supplementary file 1 [file cancers-16-03077-s001.zip › cancers-3154863-supplementary/Supplementary File 2/021A.jpg]

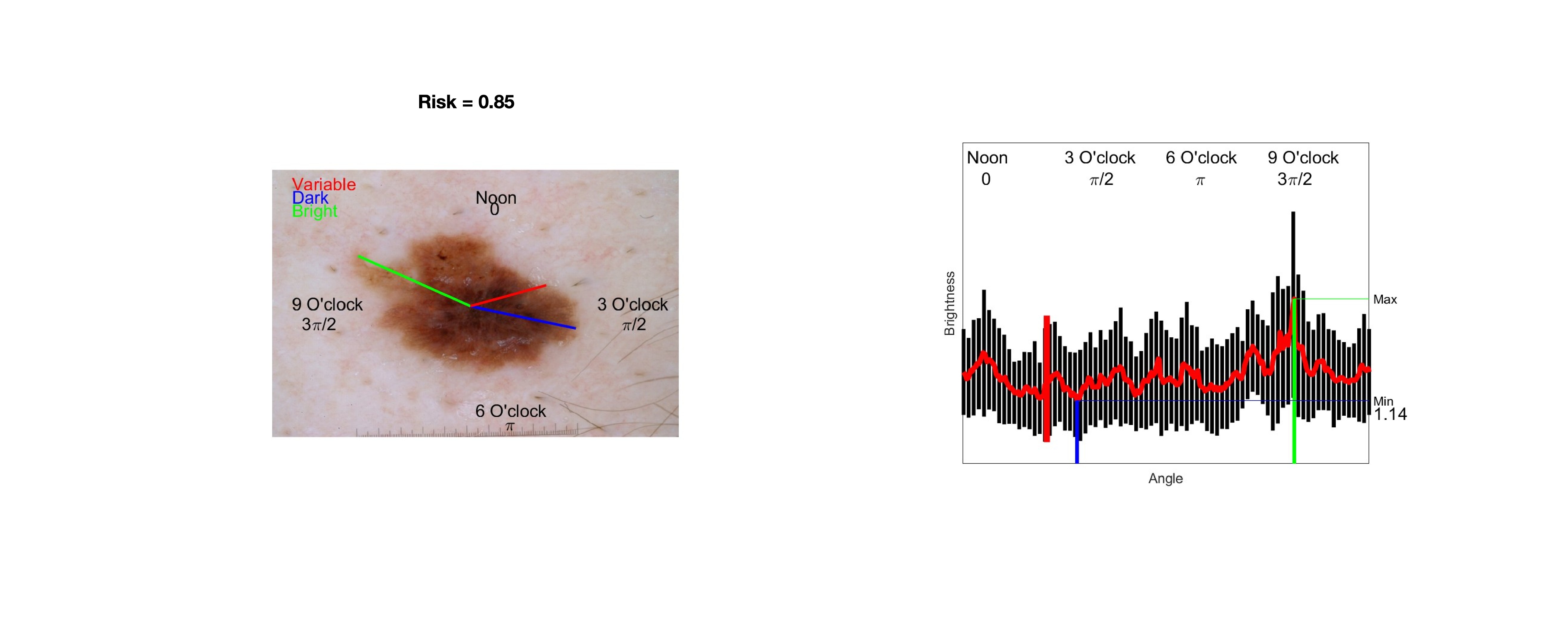

Supplement: Supplementary file 1 [file cancers-16-03077-s001.zip › cancers-3154863-supplementary/Supplementary File 2/021B.jpg]

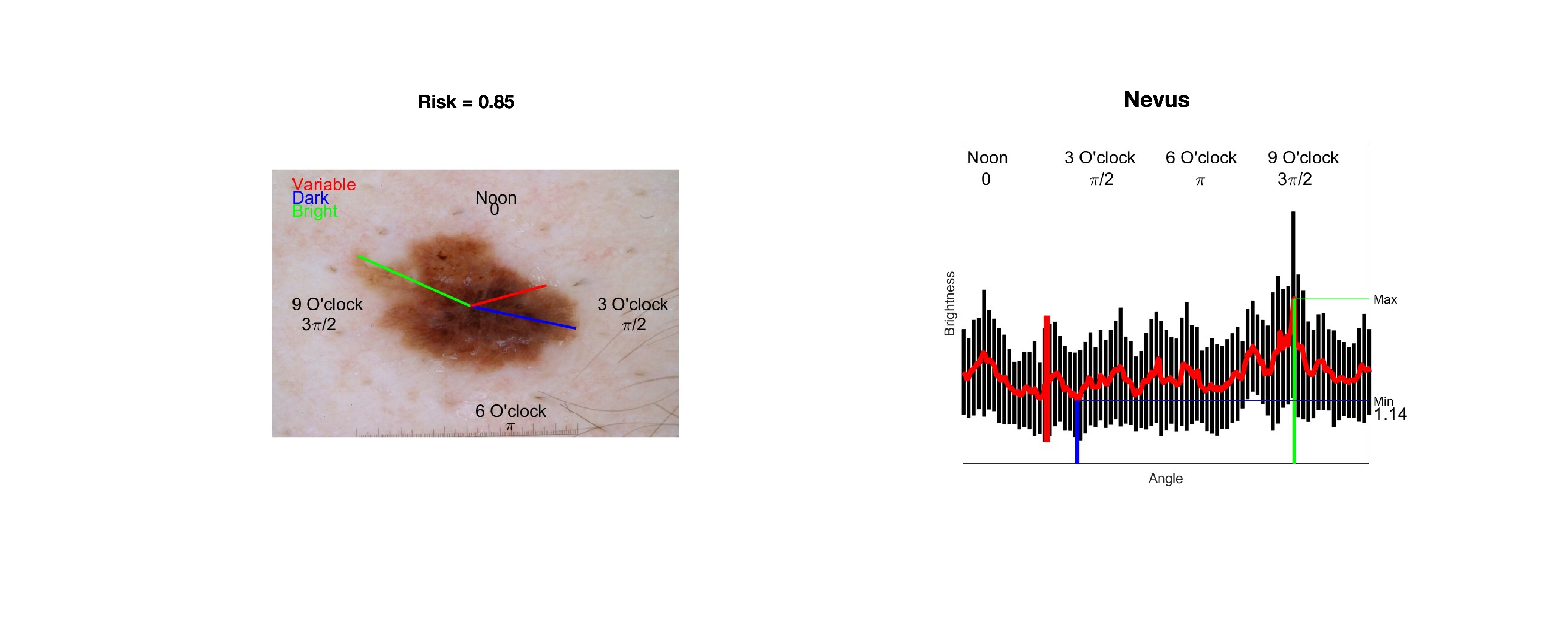

Supplement: Supplementary file 1 [file cancers-16-03077-s001.zip › cancers-3154863-supplementary/Supplementary File 2/021C.jpg]

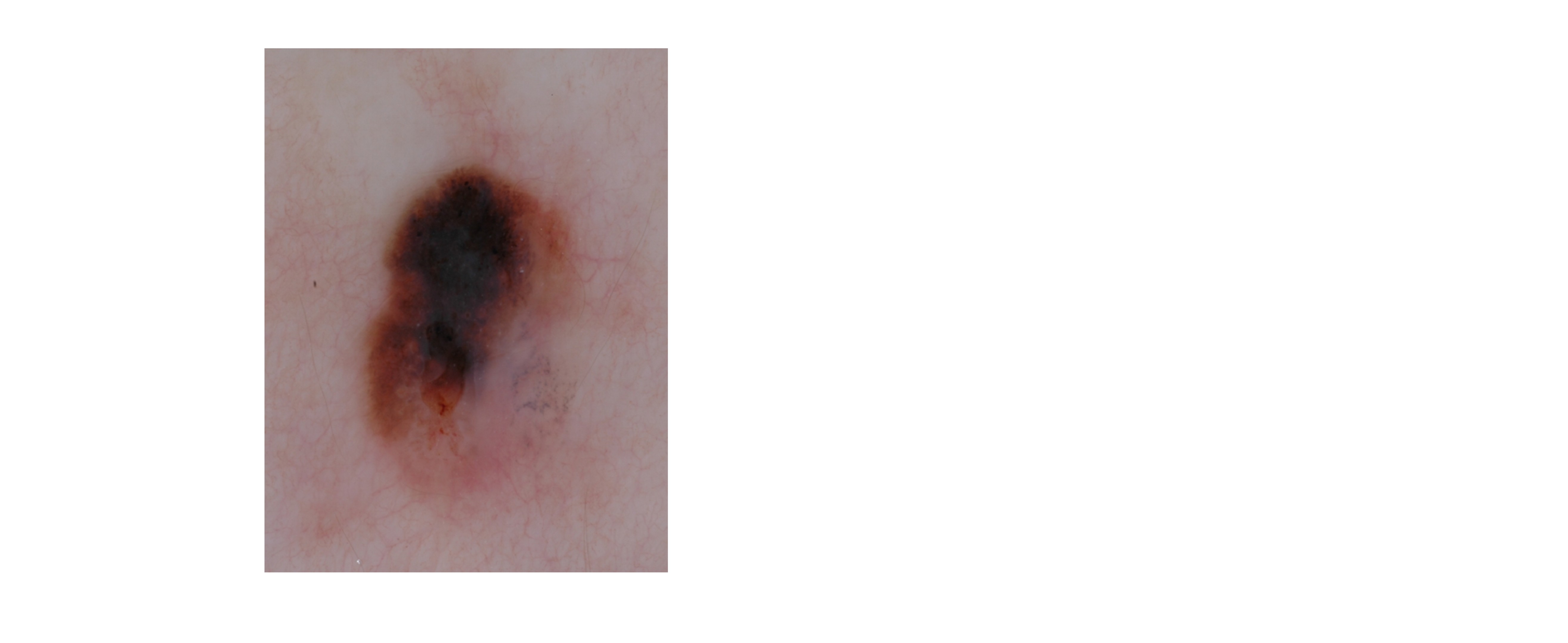

Supplement: Supplementary file 1 [file cancers-16-03077-s001.zip › cancers-3154863-supplementary/Supplementary File 2/022A.jpg]

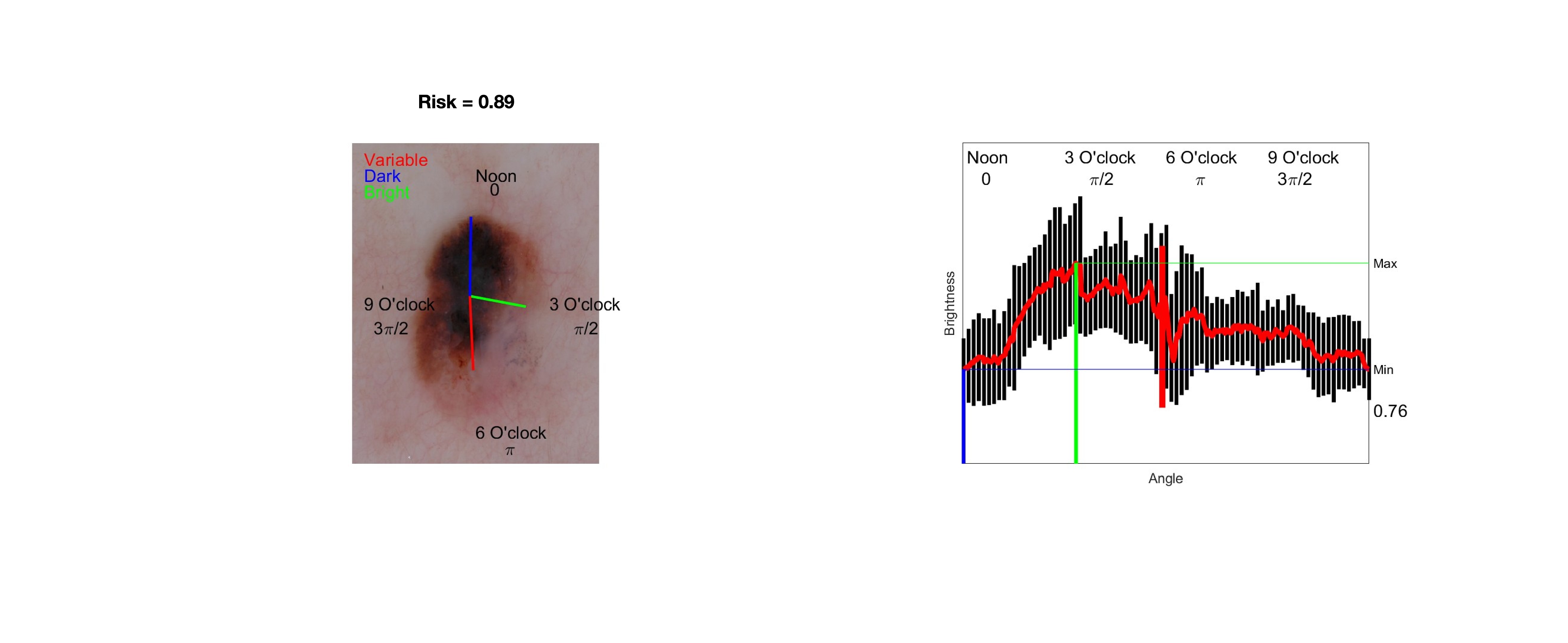

Supplement: Supplementary file 1 [file cancers-16-03077-s001.zip › cancers-3154863-supplementary/Supplementary File 2/022B.jpg]

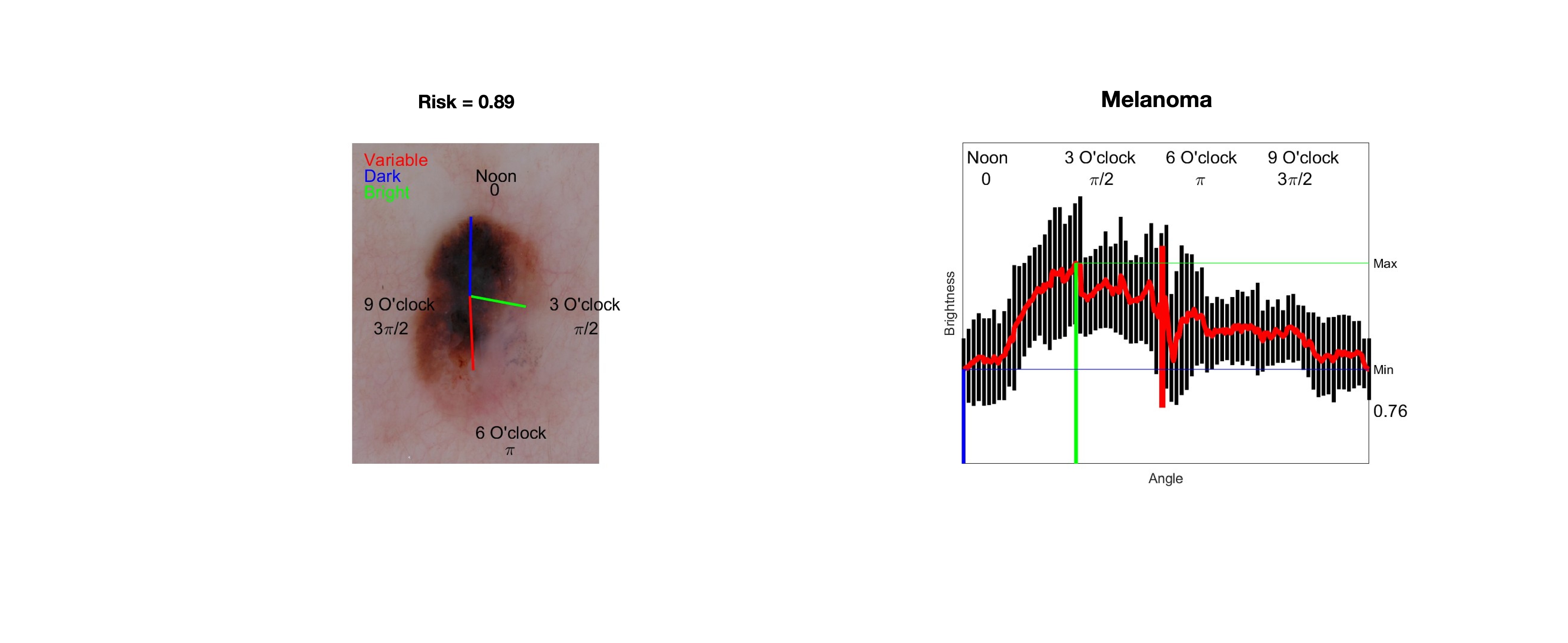

Supplement: Supplementary file 1 [file cancers-16-03077-s001.zip › cancers-3154863-supplementary/Supplementary File 2/022C.jpg]

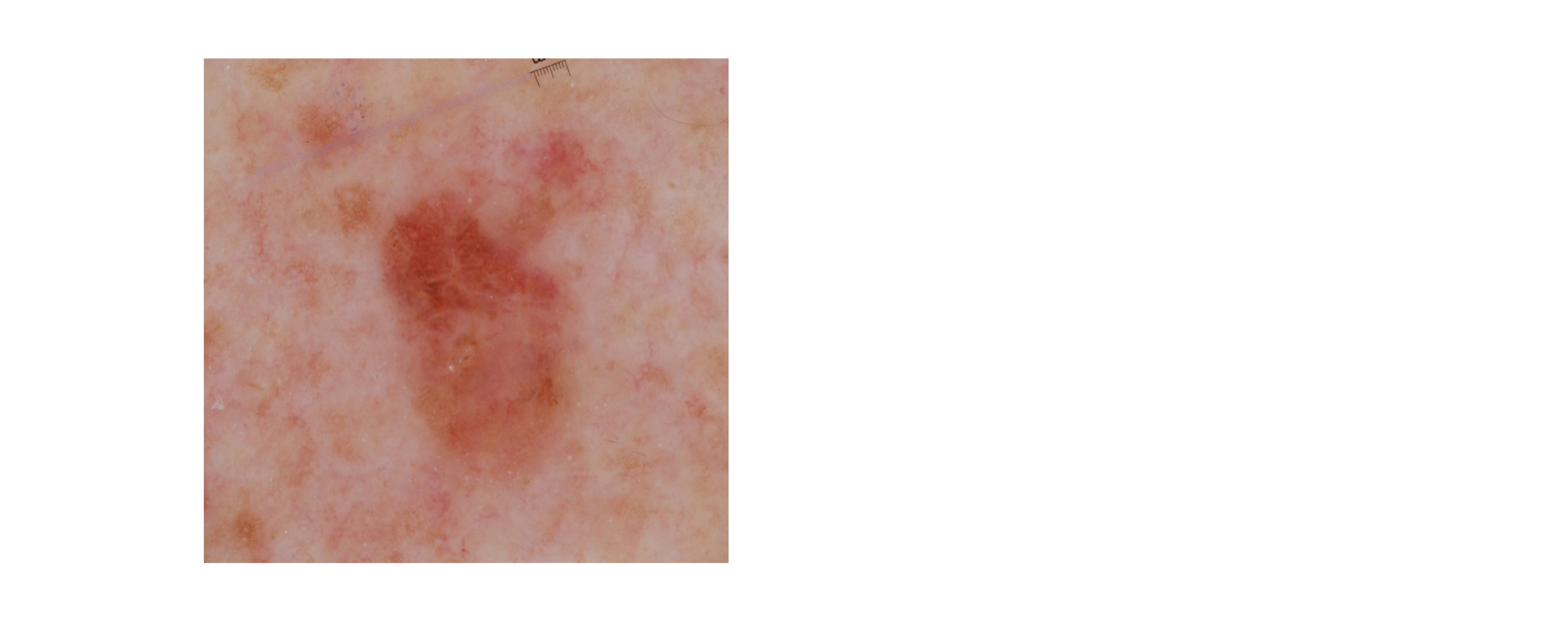

Supplement: Supplementary file 1 [file cancers-16-03077-s001.zip › cancers-3154863-supplementary/Supplementary File 2/023A.jpg]

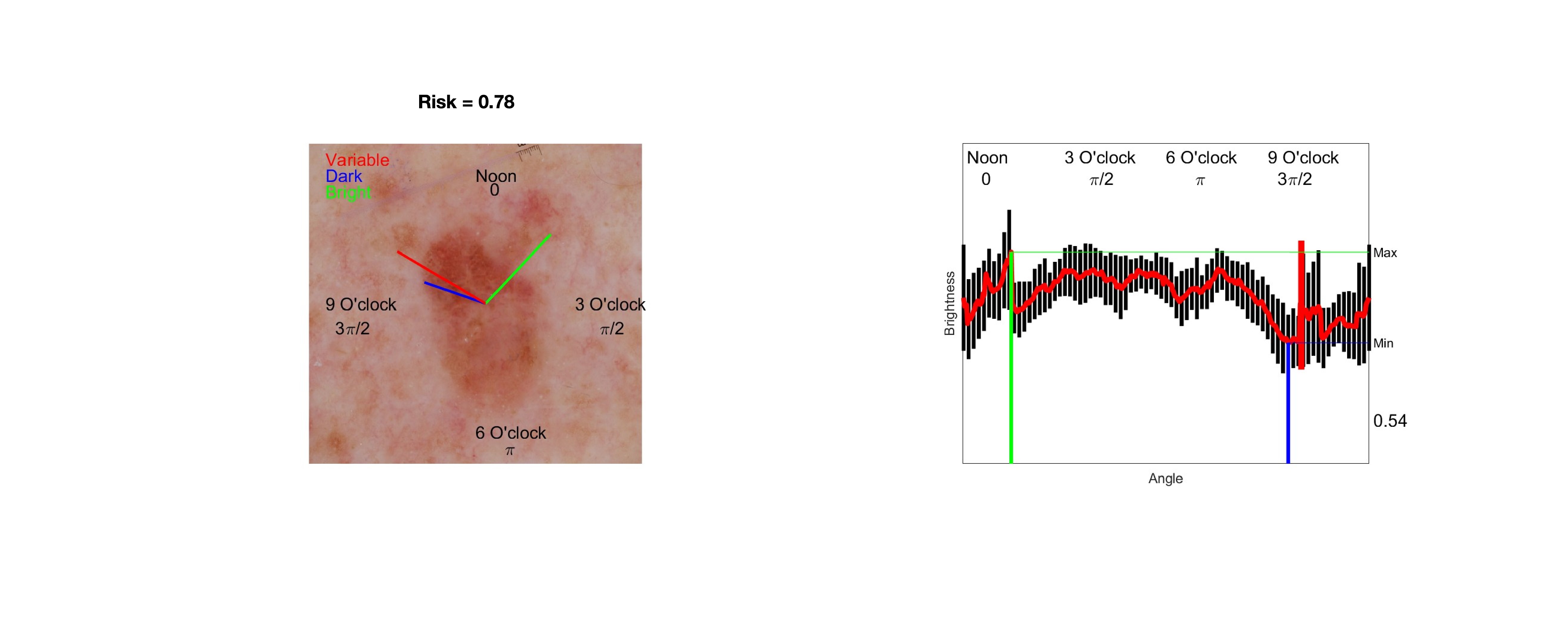

Supplement: Supplementary file 1 [file cancers-16-03077-s001.zip › cancers-3154863-supplementary/Supplementary File 2/023B.jpg]

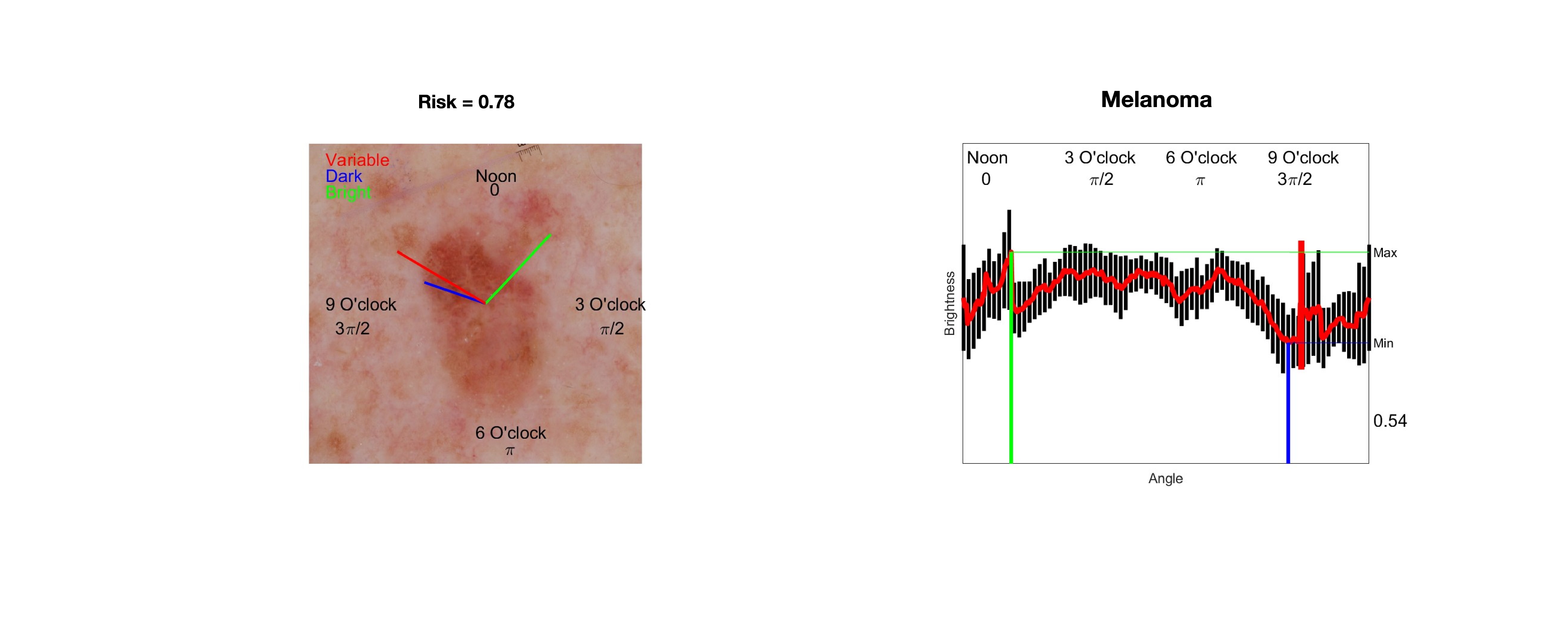

Supplement: Supplementary file 1 [file cancers-16-03077-s001.zip › cancers-3154863-supplementary/Supplementary File 2/023C.jpg]

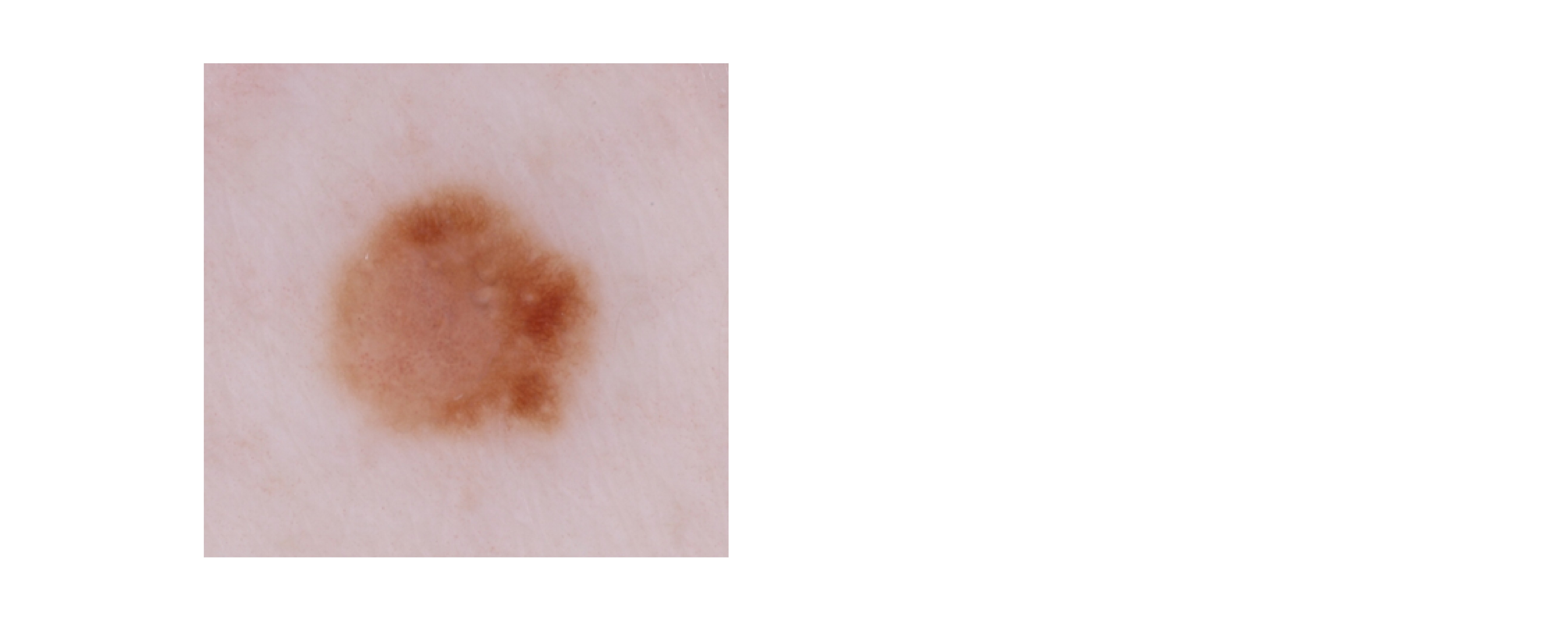

Supplement: Supplementary file 1 [file cancers-16-03077-s001.zip › cancers-3154863-supplementary/Supplementary File 2/024A.jpg]

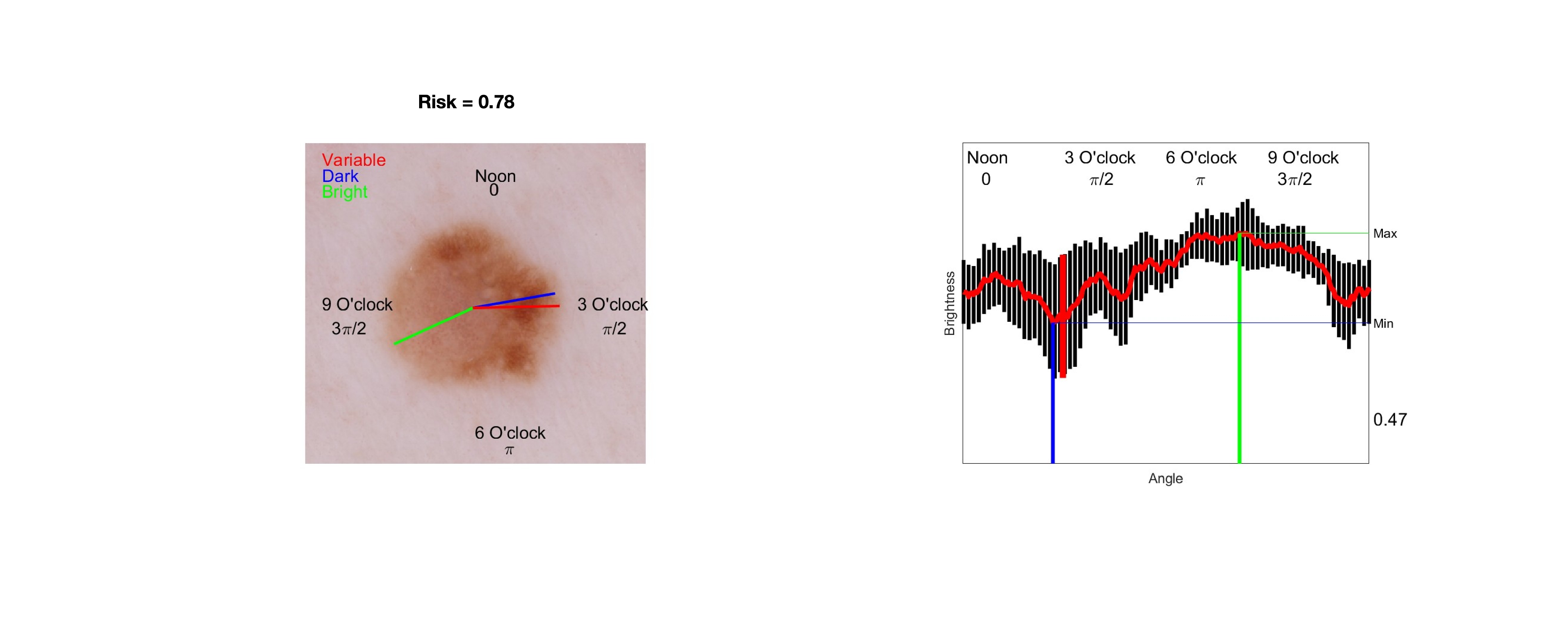

Supplement: Supplementary file 1 [file cancers-16-03077-s001.zip › cancers-3154863-supplementary/Supplementary File 2/024B.jpg]

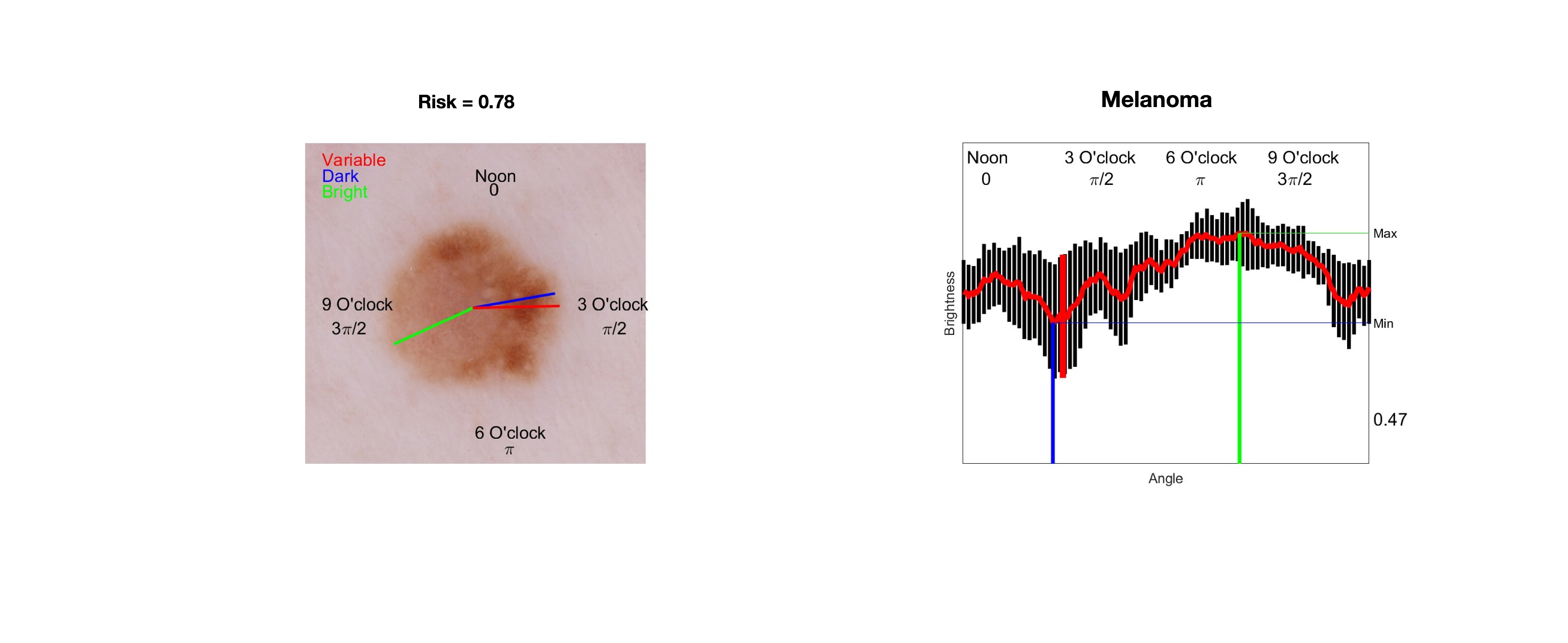

Supplement: Supplementary file 1 [file cancers-16-03077-s001.zip › cancers-3154863-supplementary/Supplementary File 2/024C.jpg]

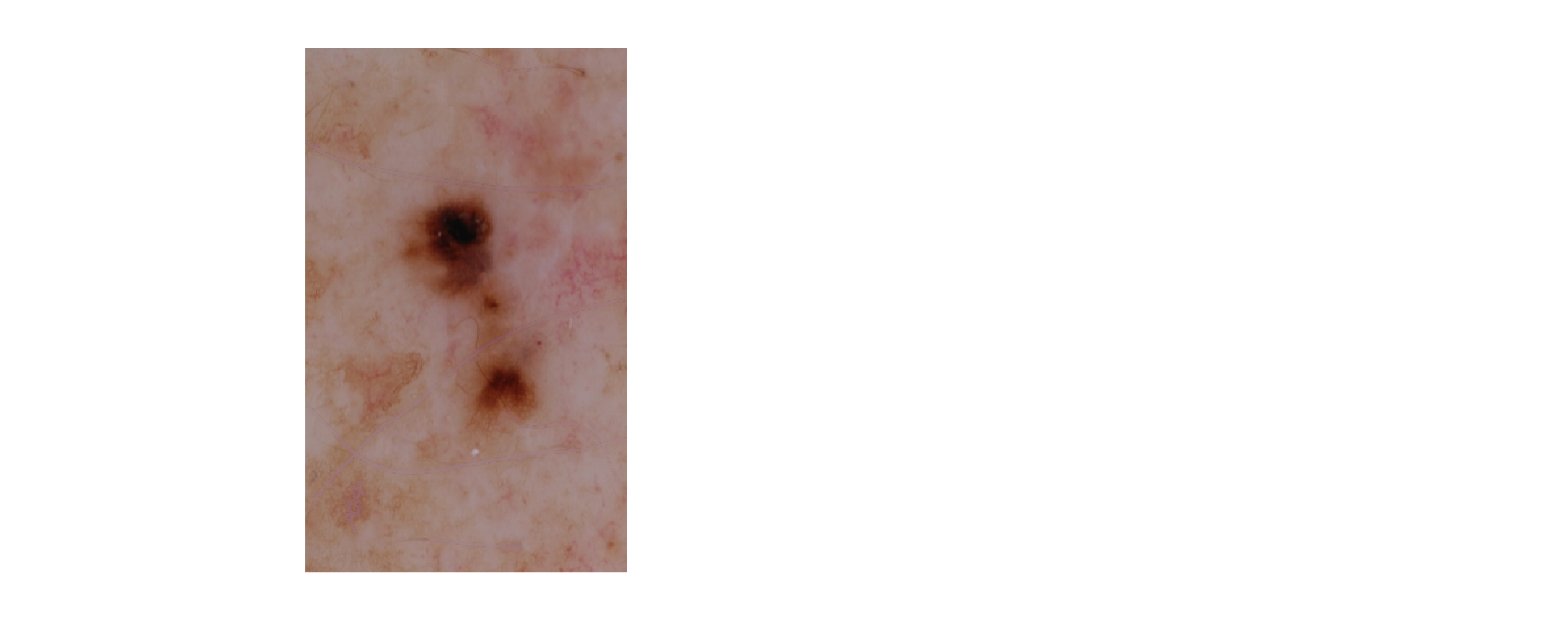

Supplement: Supplementary file 1 [file cancers-16-03077-s001.zip › cancers-3154863-supplementary/Supplementary File 2/025A.jpg]

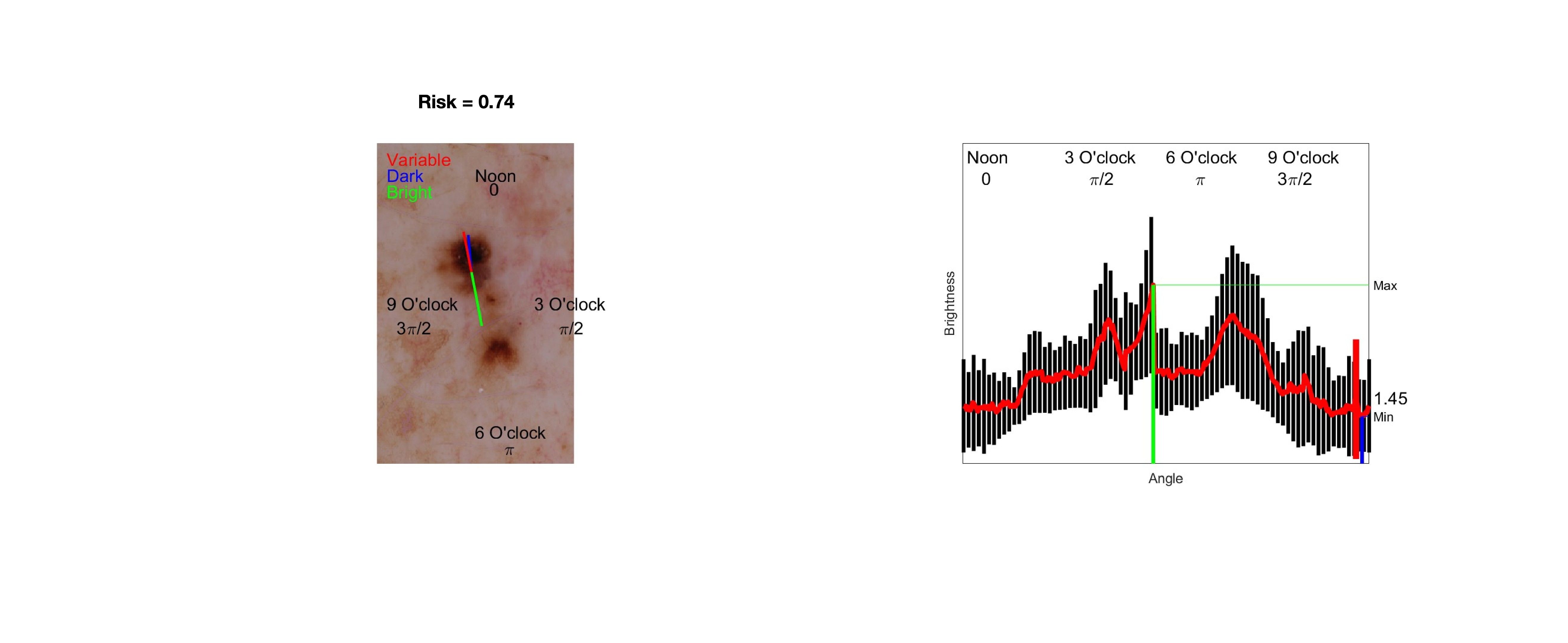

Supplement: Supplementary file 1 [file cancers-16-03077-s001.zip › cancers-3154863-supplementary/Supplementary File 2/025B.jpg]

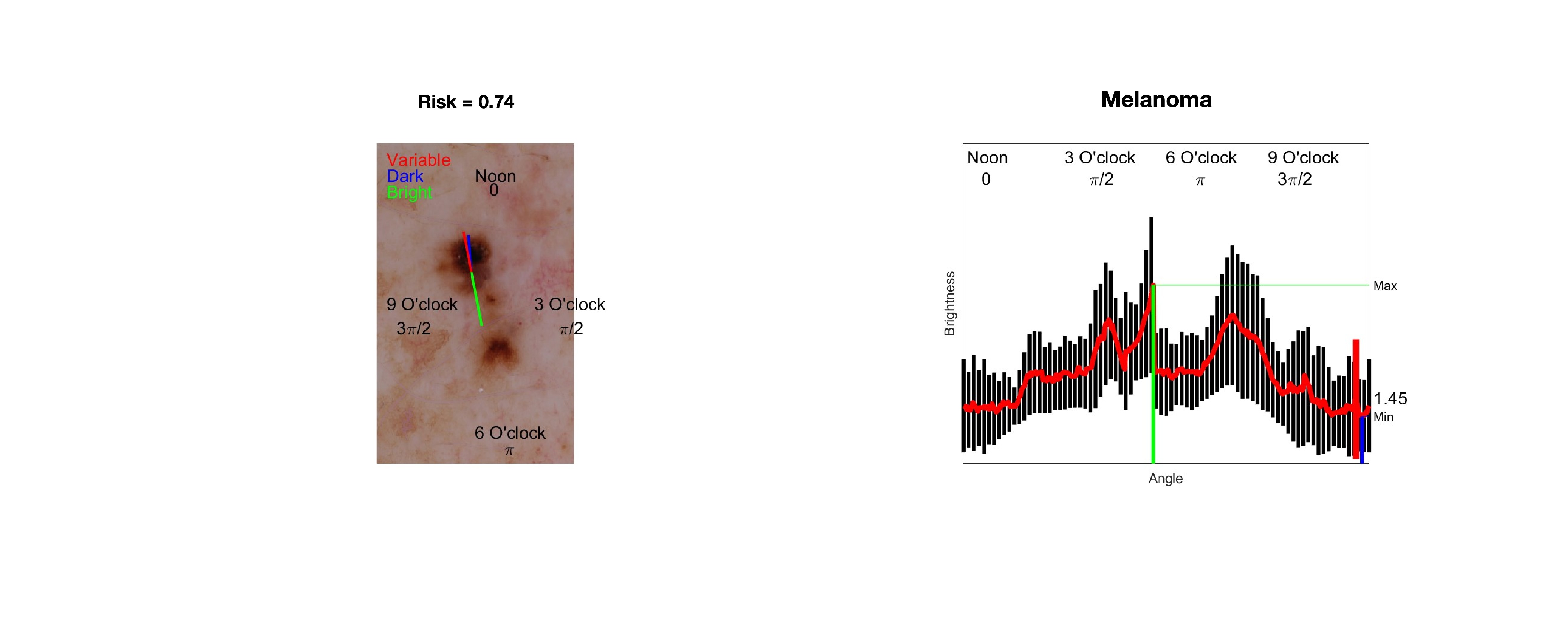

Supplement: Supplementary file 1 [file cancers-16-03077-s001.zip › cancers-3154863-supplementary/Supplementary File 2/025C.jpg]

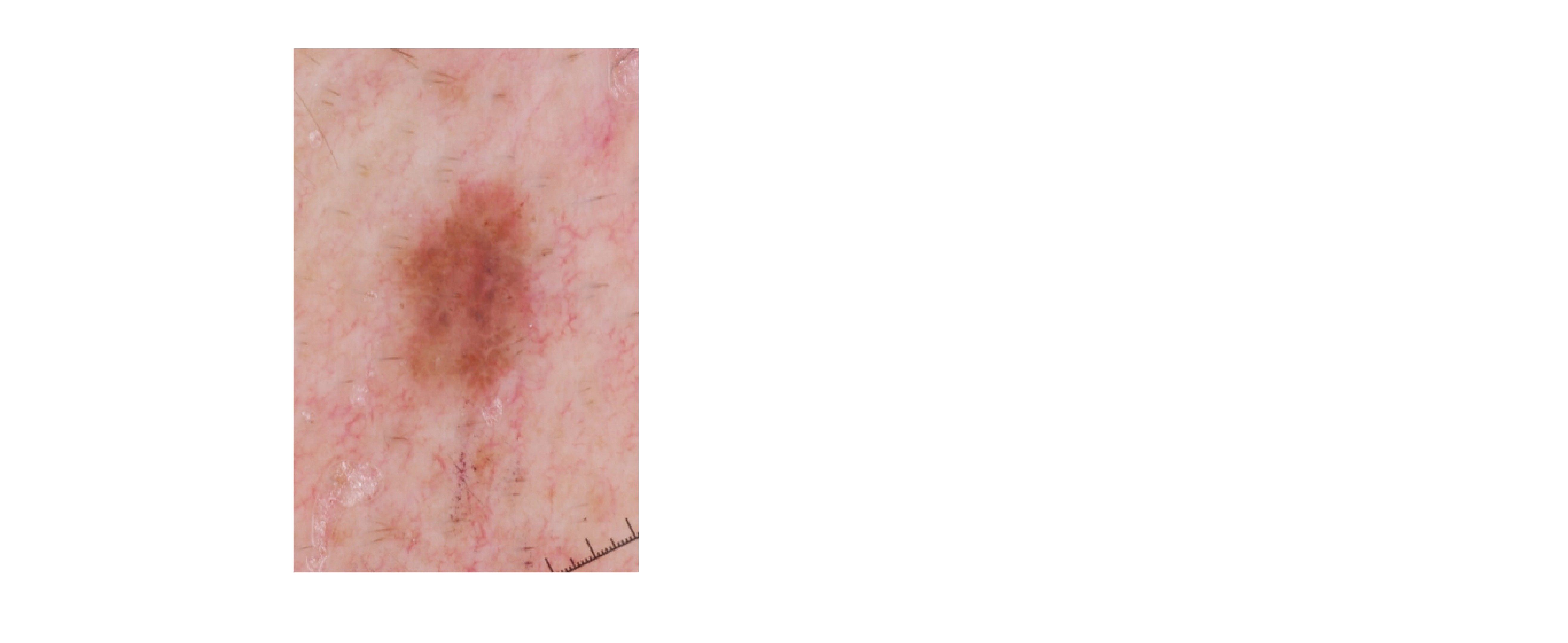

Supplement: Supplementary file 1 [file cancers-16-03077-s001.zip › cancers-3154863-supplementary/Supplementary File 2/026A.jpg]

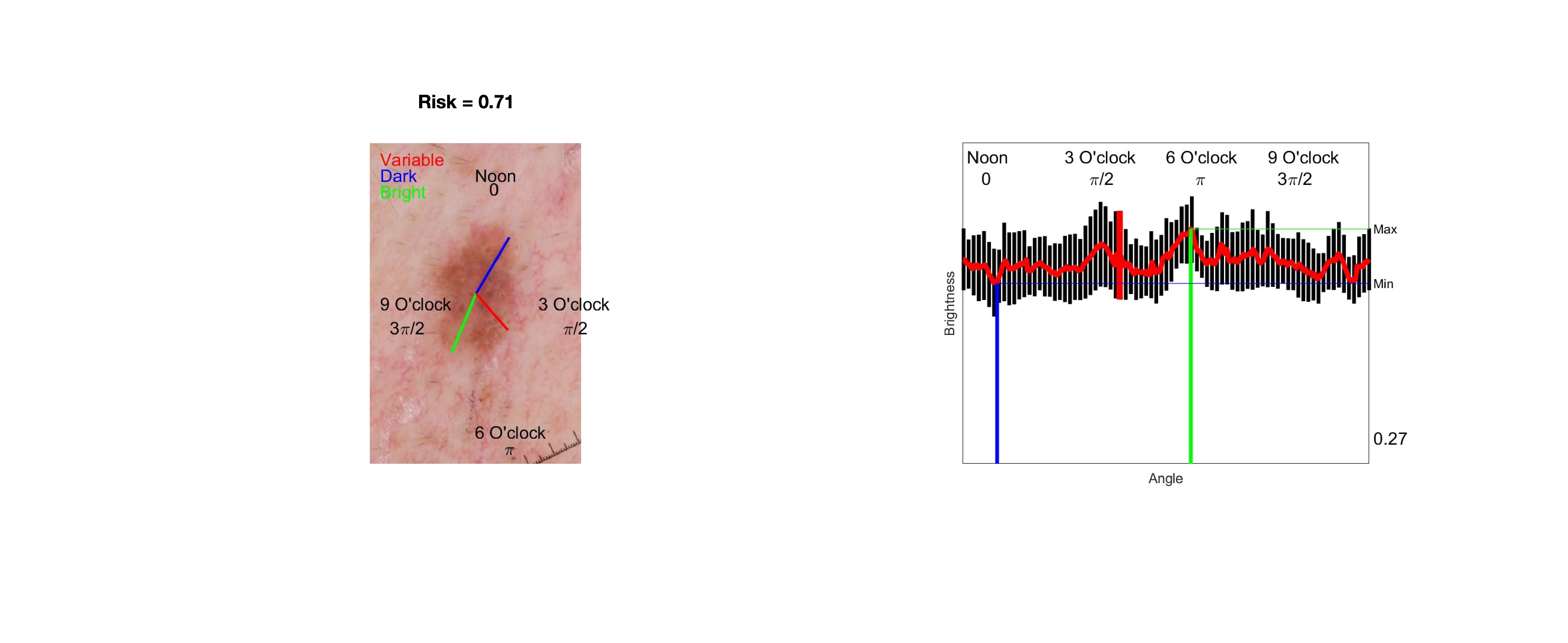

Supplement: Supplementary file 1 [file cancers-16-03077-s001.zip › cancers-3154863-supplementary/Supplementary File 2/026B.jpg]

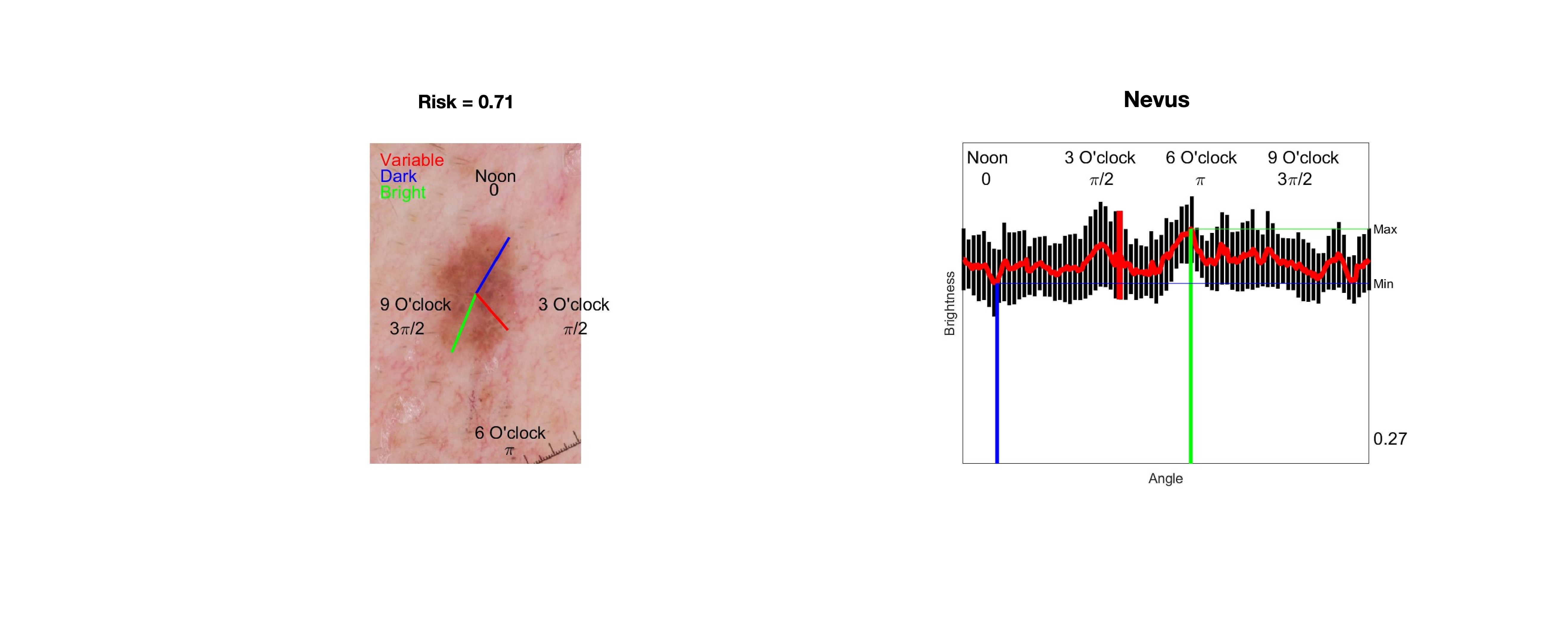

Supplement: Supplementary file 1 [file cancers-16-03077-s001.zip › cancers-3154863-supplementary/Supplementary File 2/026C.jpg]

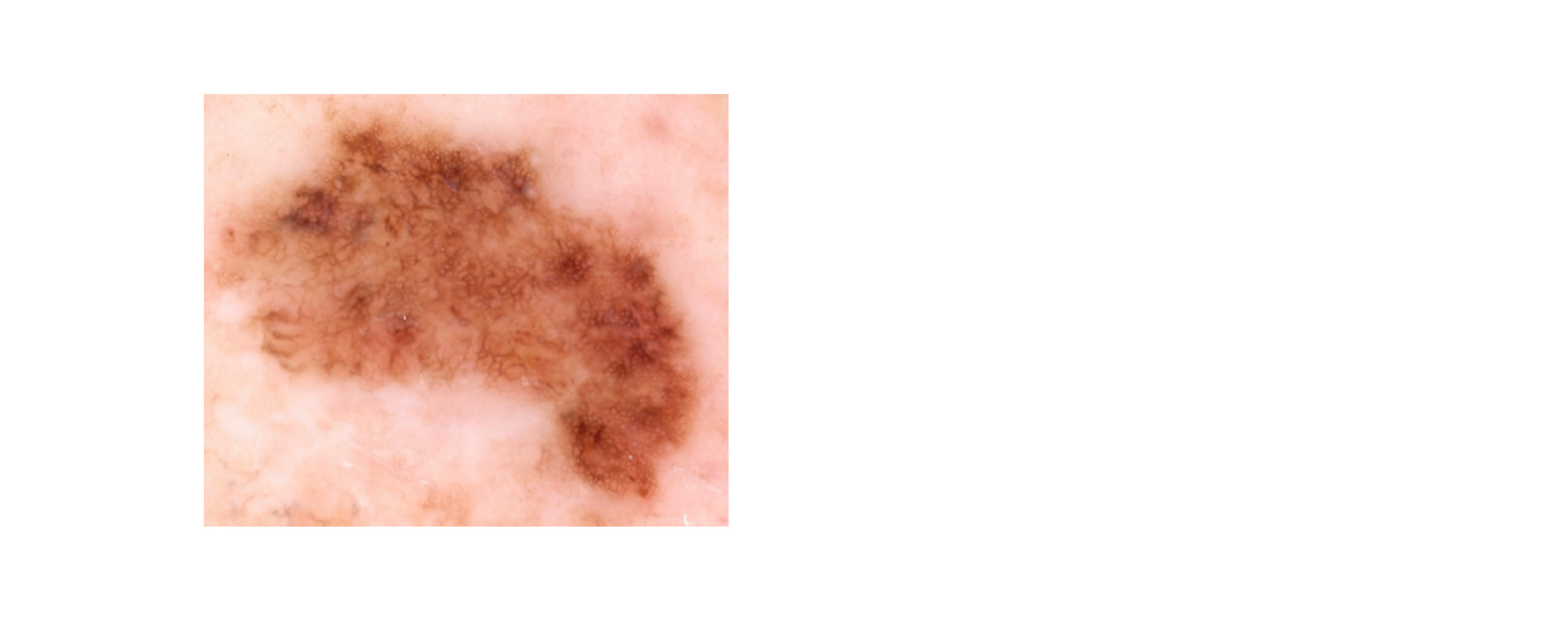

Supplement: Supplementary file 1 [file cancers-16-03077-s001.zip › cancers-3154863-supplementary/Supplementary File 2/027A.jpg]

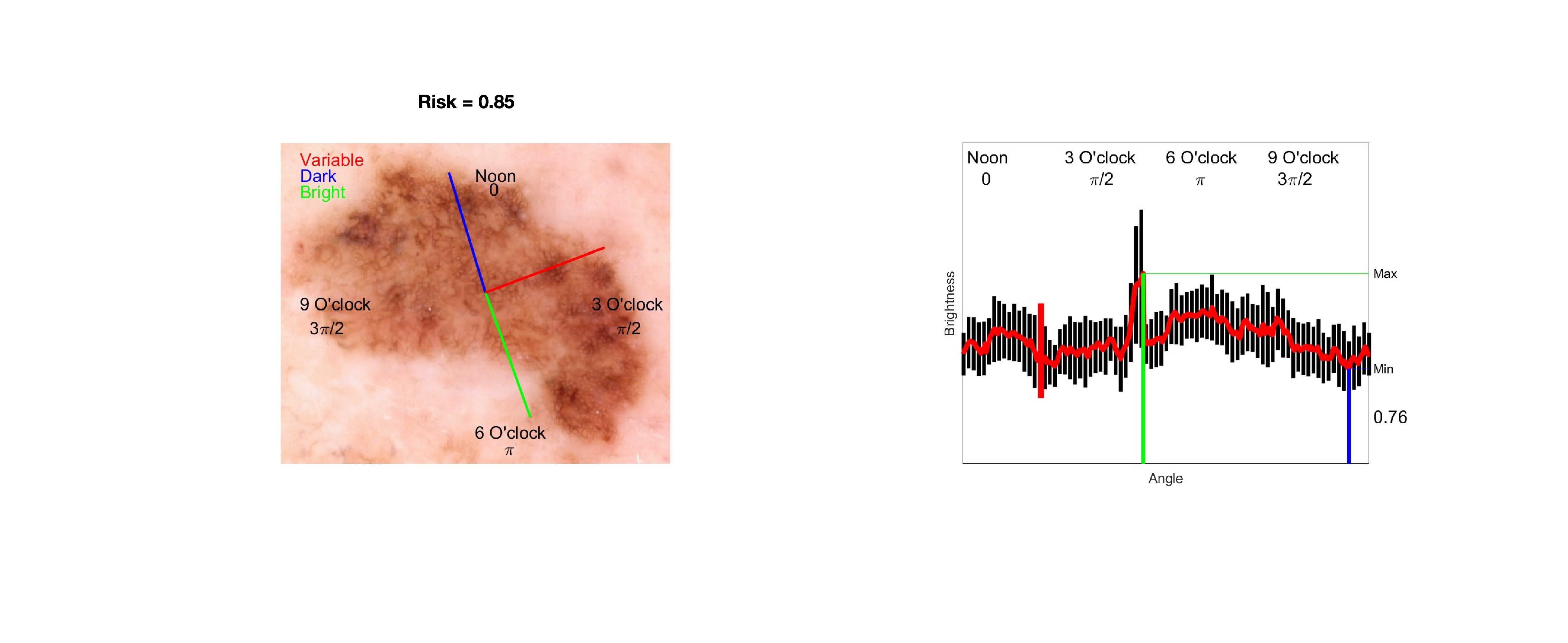

Supplement: Supplementary file 1 [file cancers-16-03077-s001.zip › cancers-3154863-supplementary/Supplementary File 2/027B.jpg]

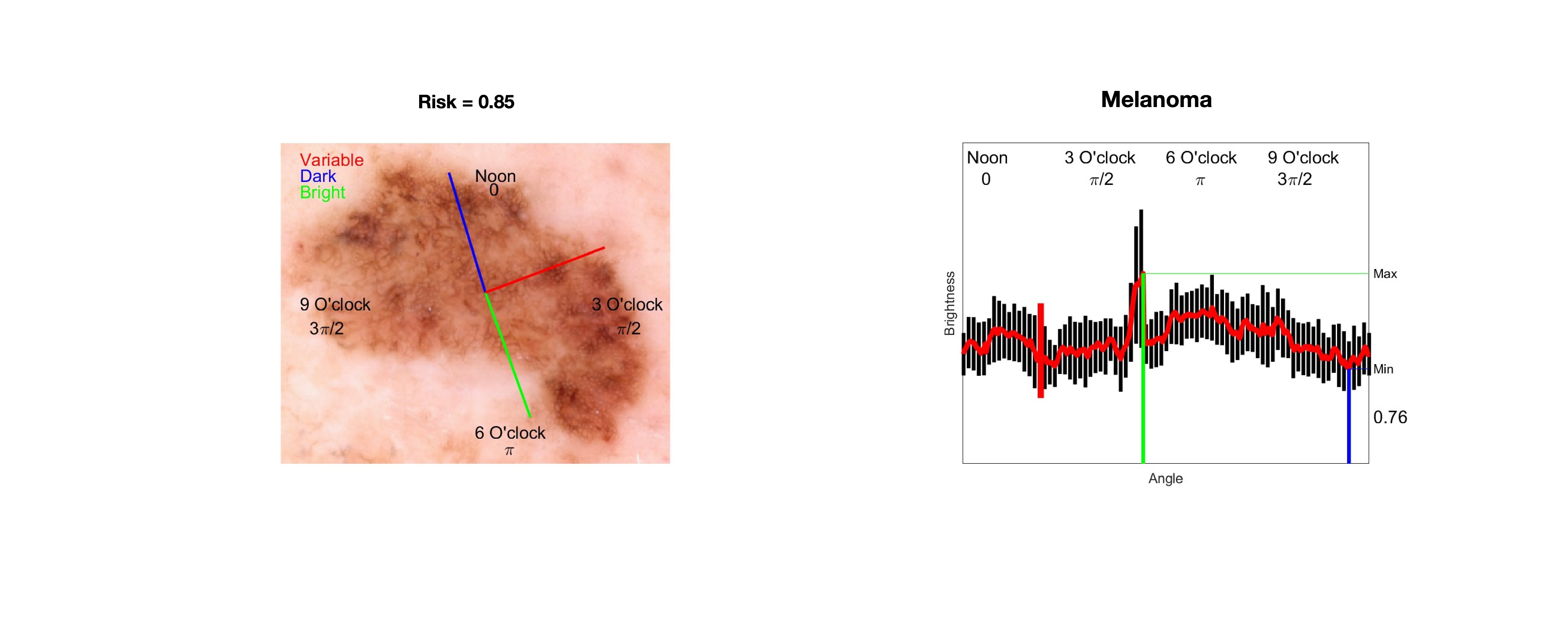

Supplement: Supplementary file 1 [file cancers-16-03077-s001.zip › cancers-3154863-supplementary/Supplementary File 2/027C.jpg]

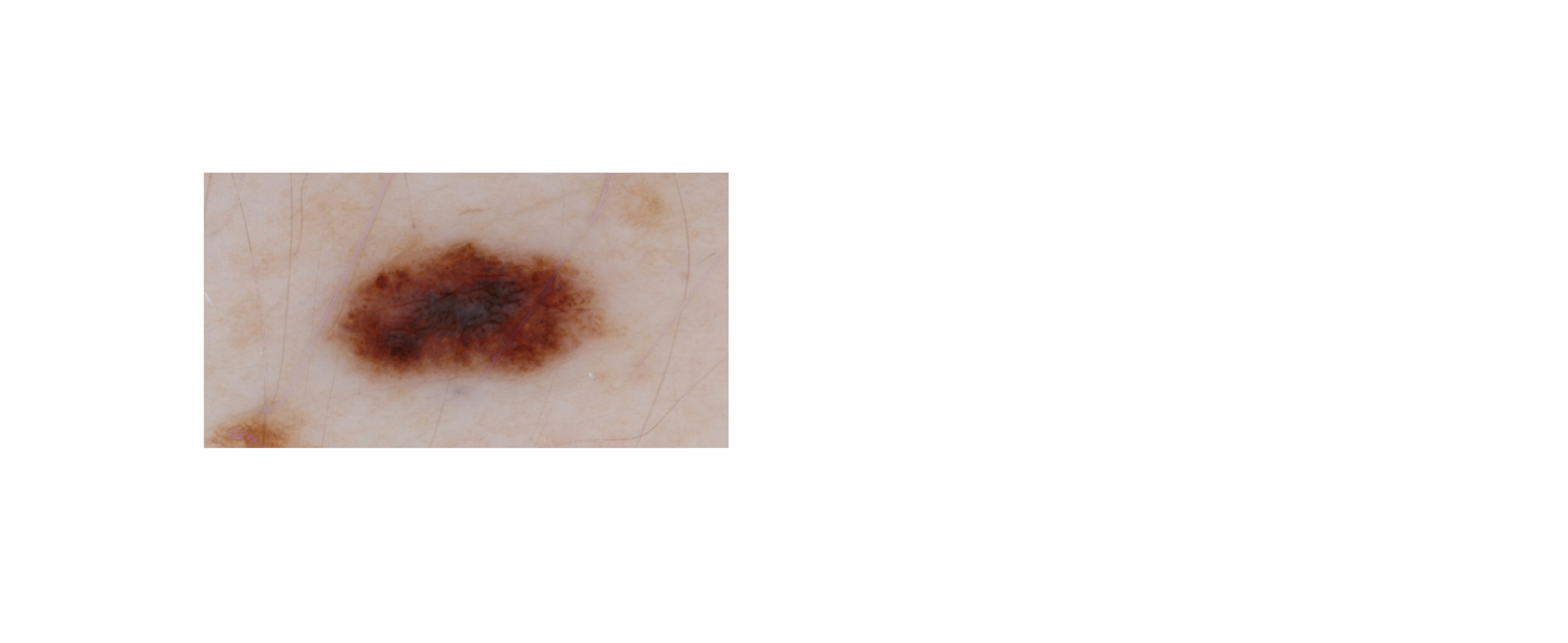

Supplement: Supplementary file 1 [file cancers-16-03077-s001.zip › cancers-3154863-supplementary/Supplementary File 2/028A.jpg]

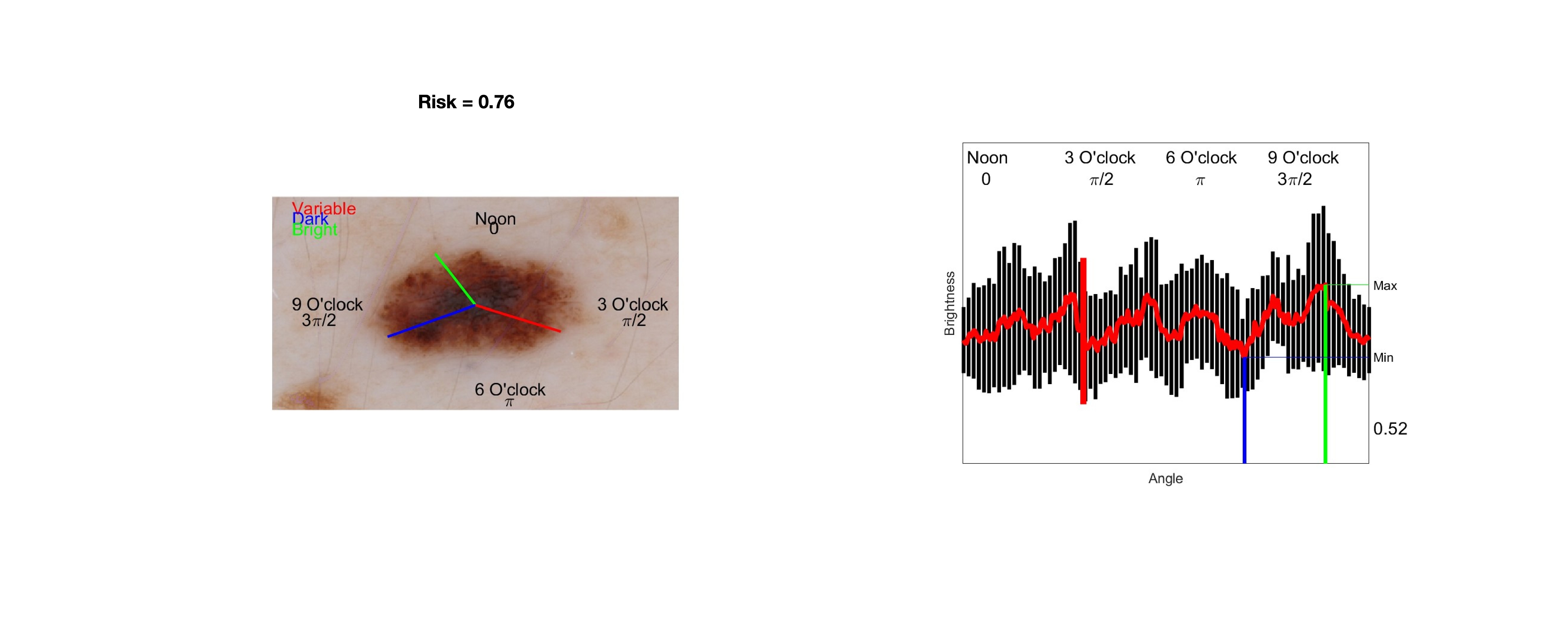

Supplement: Supplementary file 1 [file cancers-16-03077-s001.zip › cancers-3154863-supplementary/Supplementary File 2/028B.jpg]

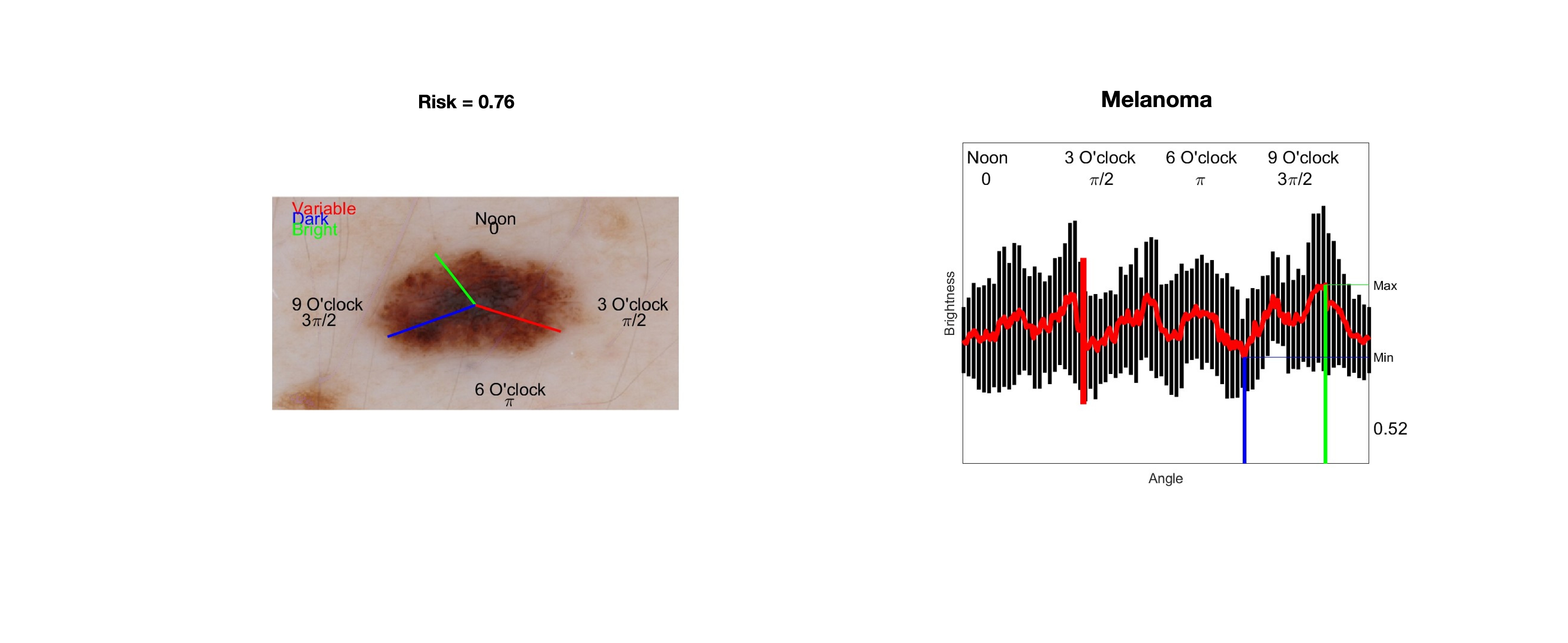

Supplement: Supplementary file 1 [file cancers-16-03077-s001.zip › cancers-3154863-supplementary/Supplementary File 2/028C.jpg]

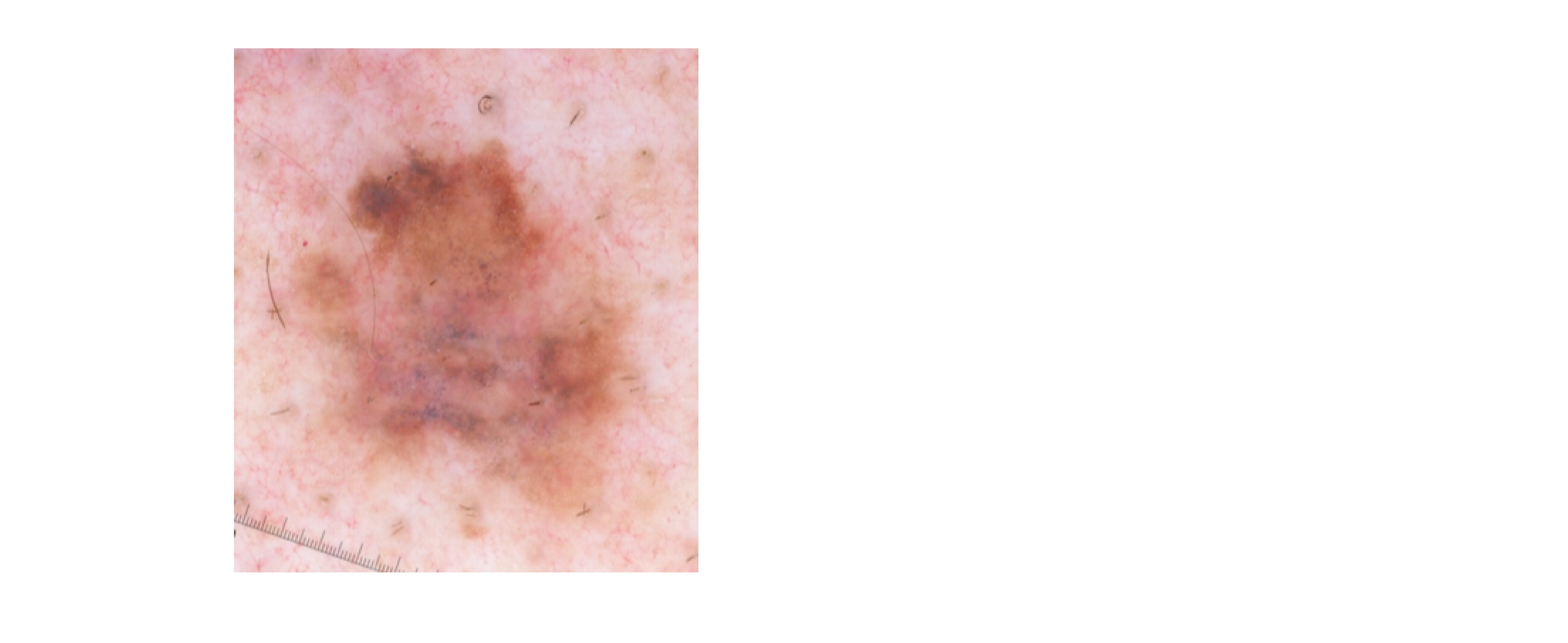

Supplement: Supplementary file 1 [file cancers-16-03077-s001.zip › cancers-3154863-supplementary/Supplementary File 2/029A.jpg]

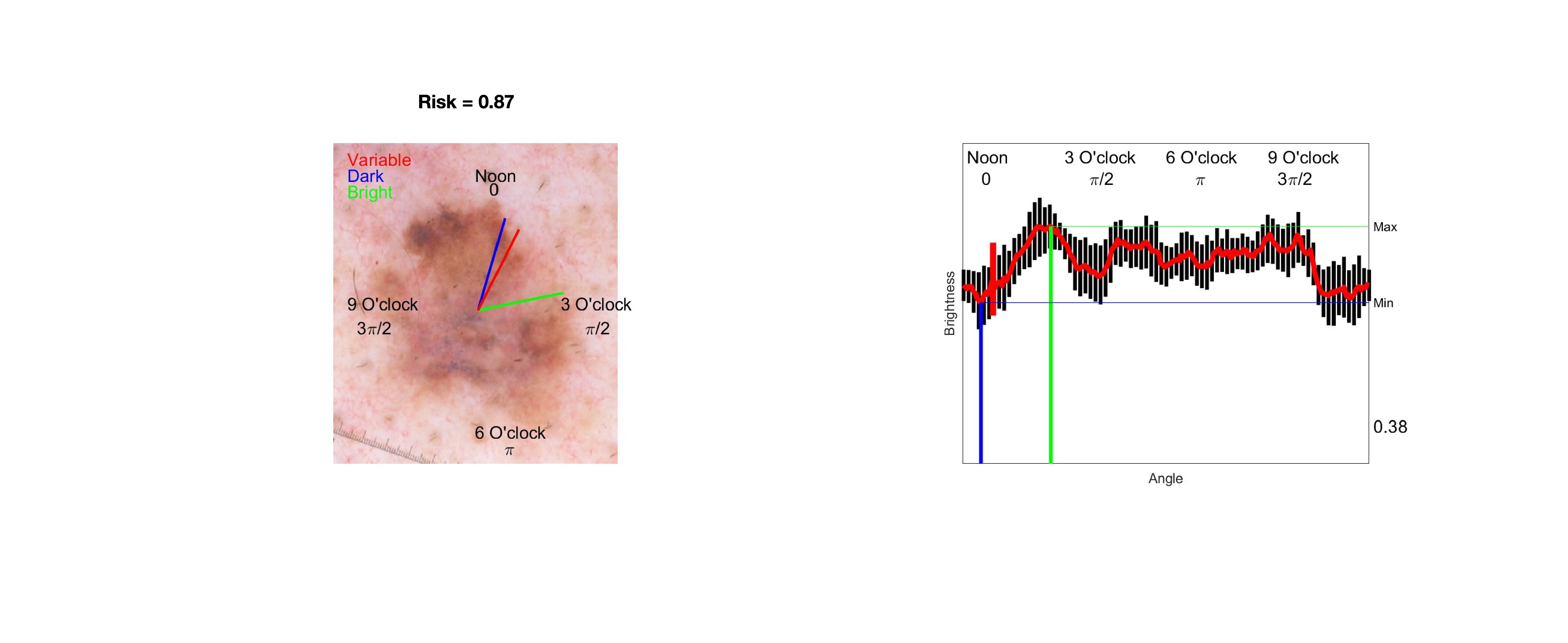

Supplement: Supplementary file 1 [file cancers-16-03077-s001.zip › cancers-3154863-supplementary/Supplementary File 2/029B.jpg]

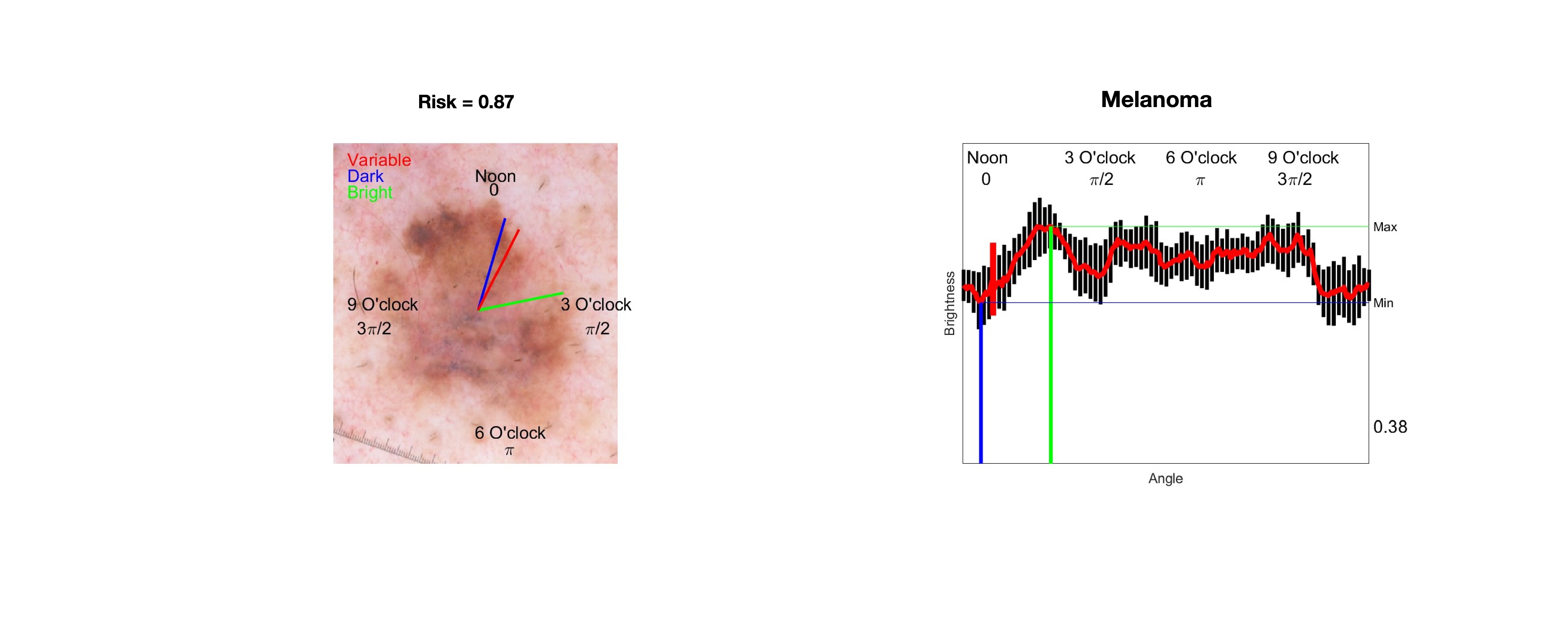

Supplement: Supplementary file 1 [file cancers-16-03077-s001.zip › cancers-3154863-supplementary/Supplementary File 2/029C.jpg]

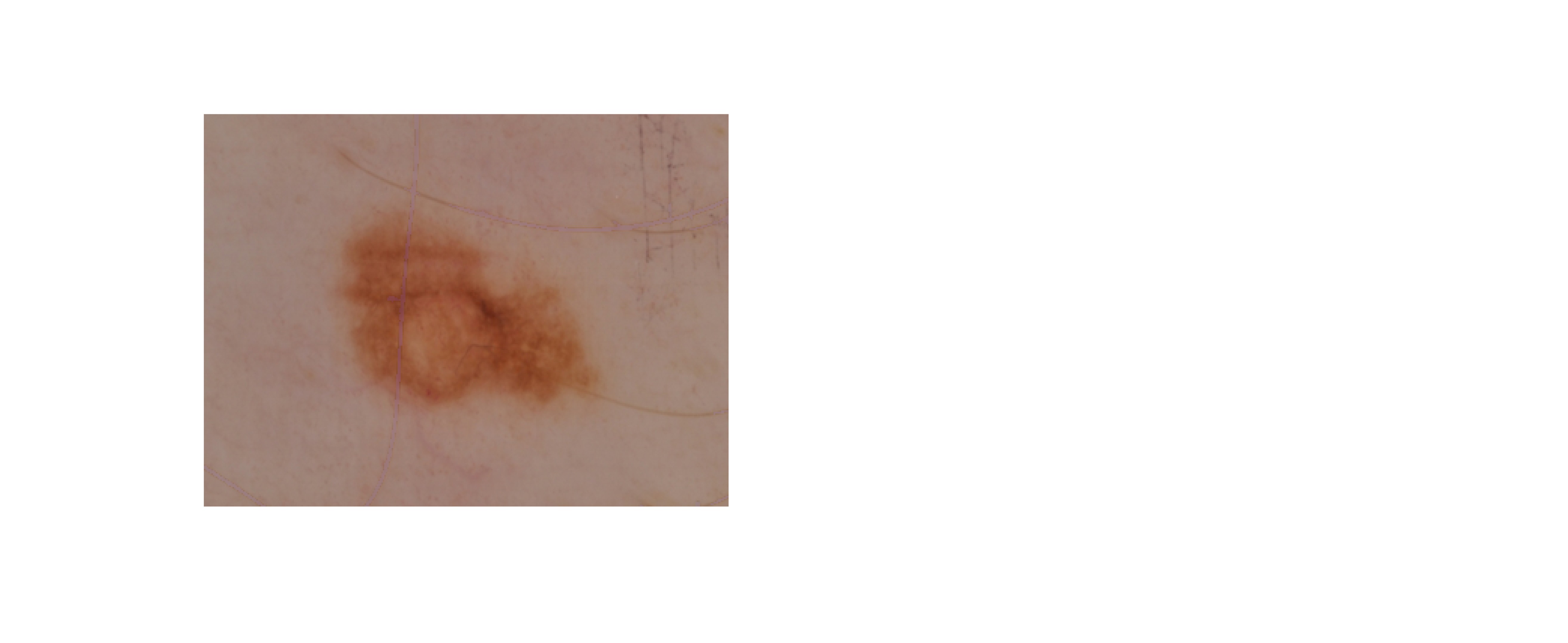

Supplement: Supplementary file 1 [file cancers-16-03077-s001.zip › cancers-3154863-supplementary/Supplementary File 2/030A.jpg]

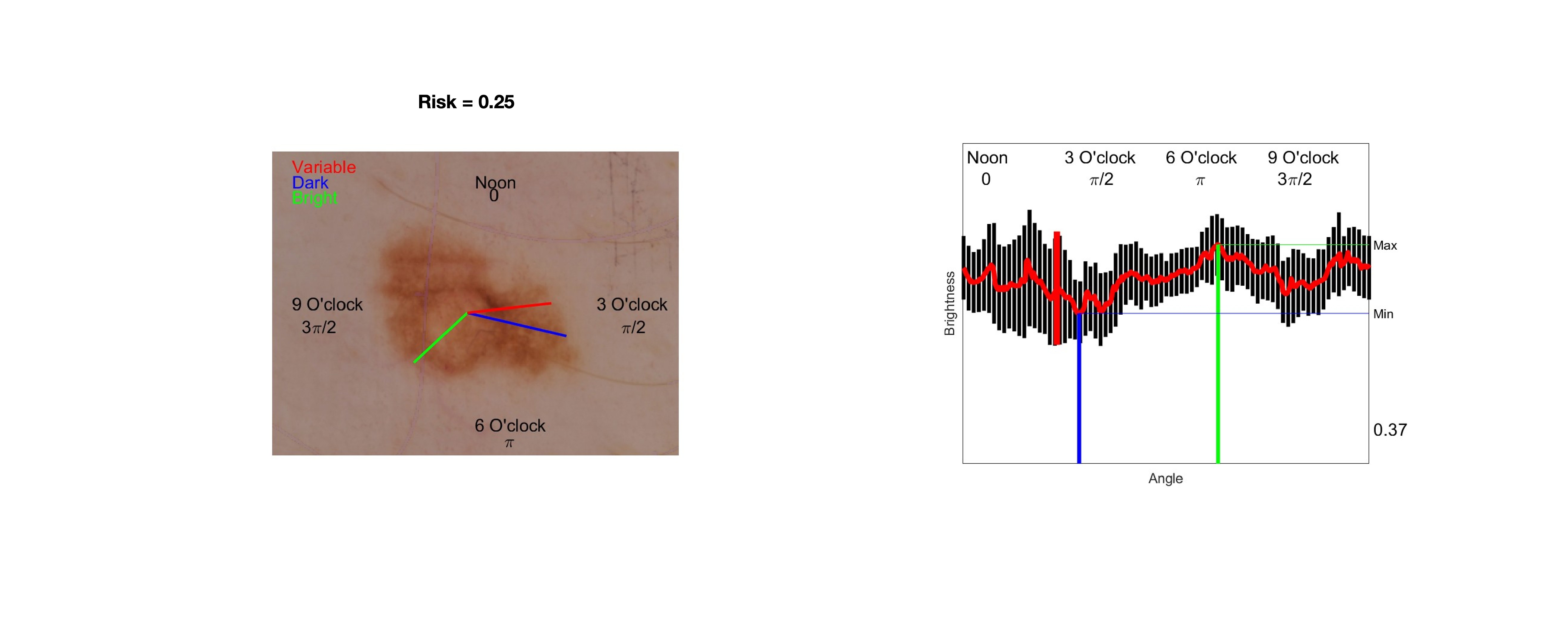

Supplement: Supplementary file 1 [file cancers-16-03077-s001.zip › cancers-3154863-supplementary/Supplementary File 2/030B.jpg]

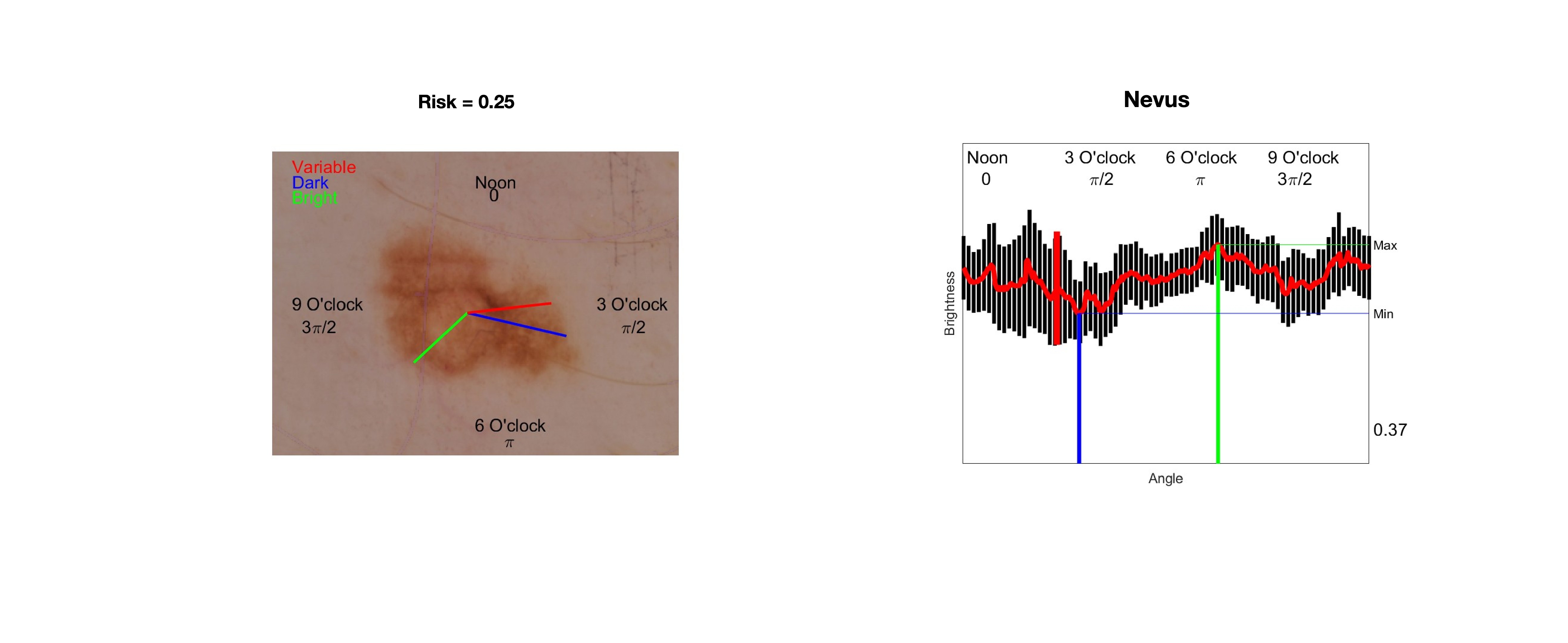

Supplement: Supplementary file 1 [file cancers-16-03077-s001.zip › cancers-3154863-supplementary/Supplementary File 2/030C.jpg]

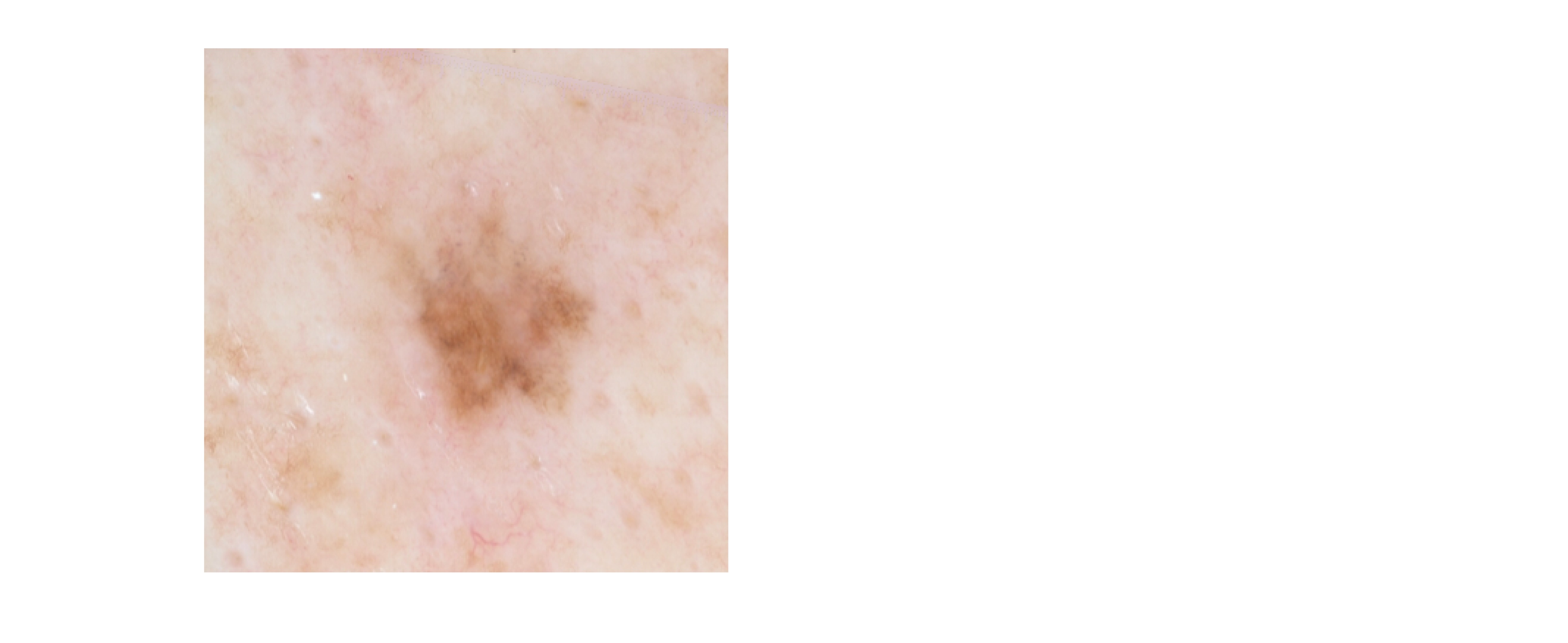

Supplement: Supplementary file 1 [file cancers-16-03077-s001.zip › cancers-3154863-supplementary/Supplementary File 2/031A.jpg]

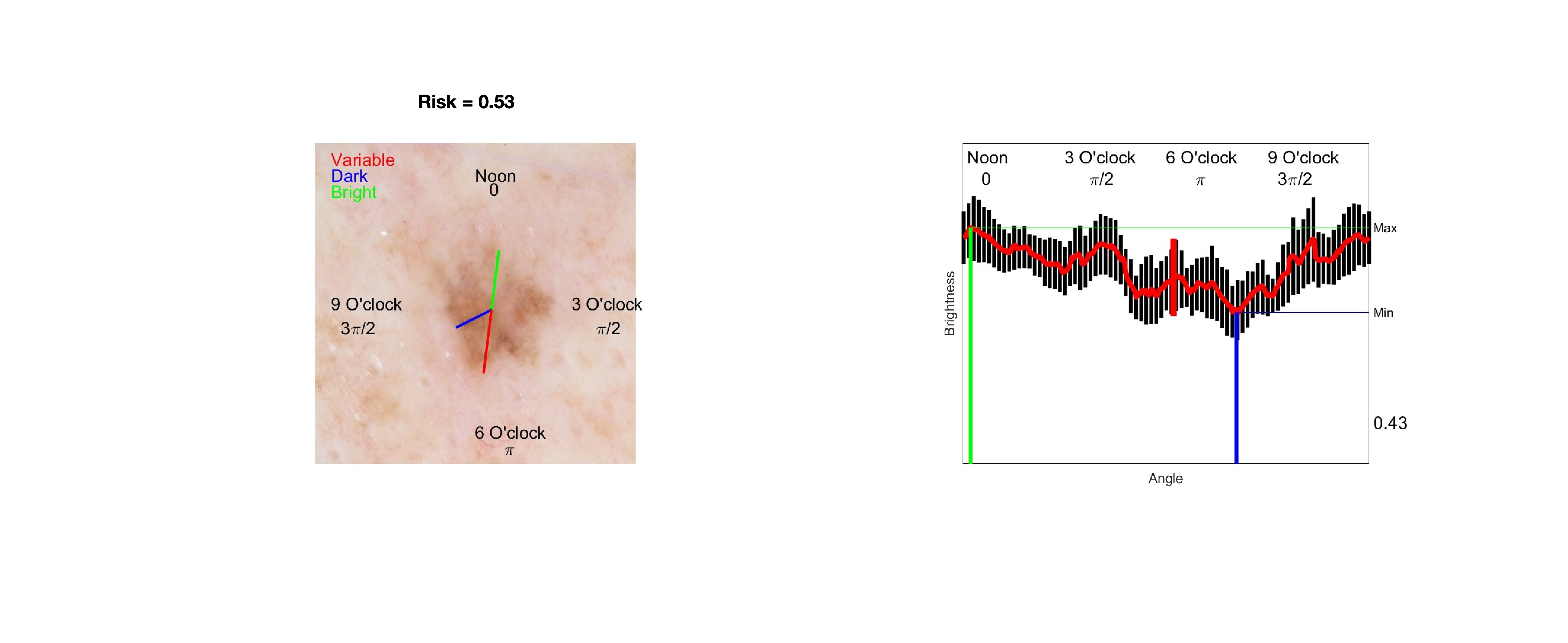

Supplement: Supplementary file 1 [file cancers-16-03077-s001.zip › cancers-3154863-supplementary/Supplementary File 2/031B.jpg]

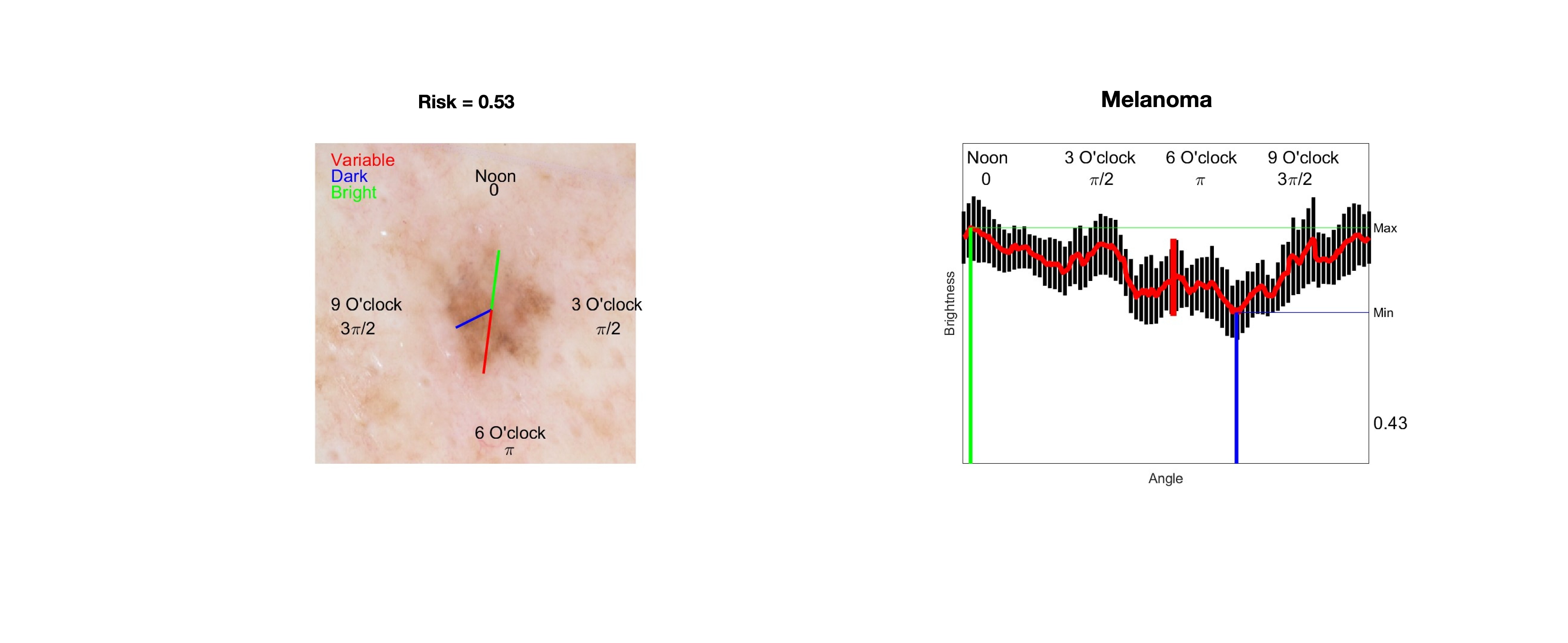

Supplement: Supplementary file 1 [file cancers-16-03077-s001.zip › cancers-3154863-supplementary/Supplementary File 2/031C.jpg]

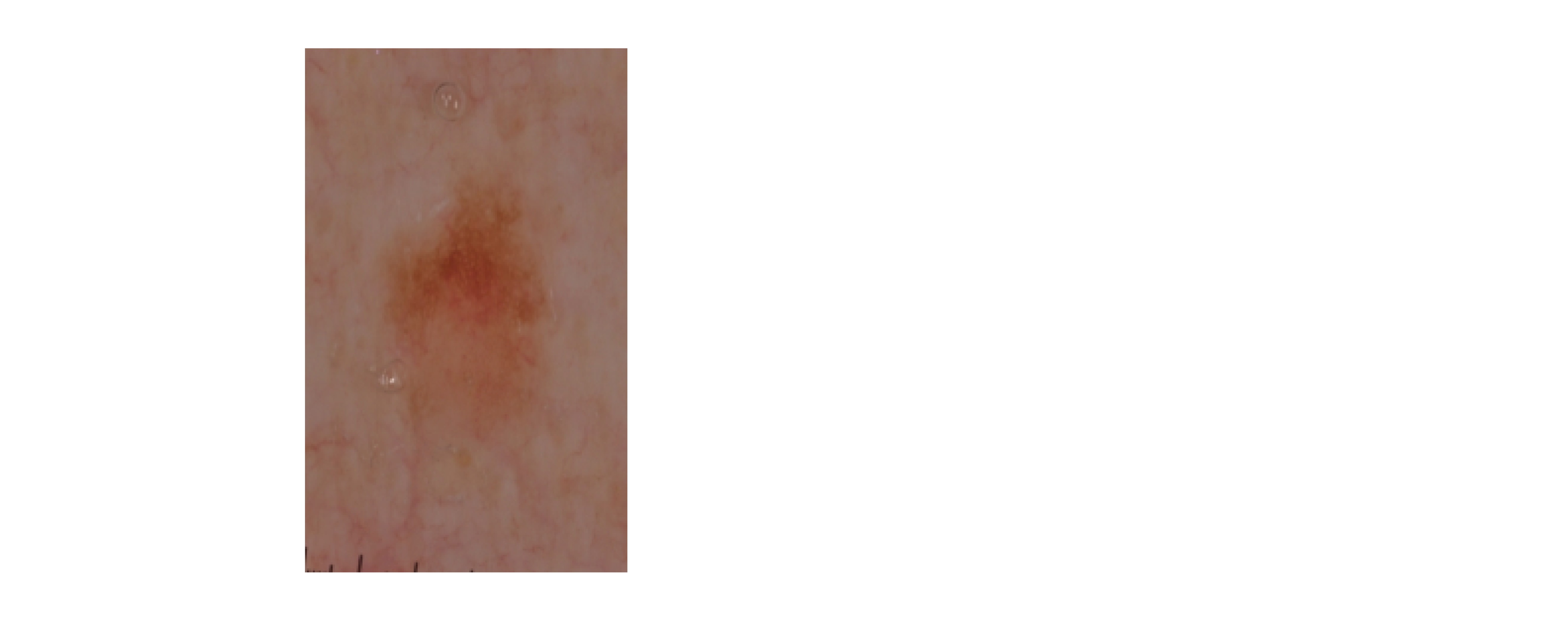

Supplement: Supplementary file 1 [file cancers-16-03077-s001.zip › cancers-3154863-supplementary/Supplementary File 2/032A.jpg]

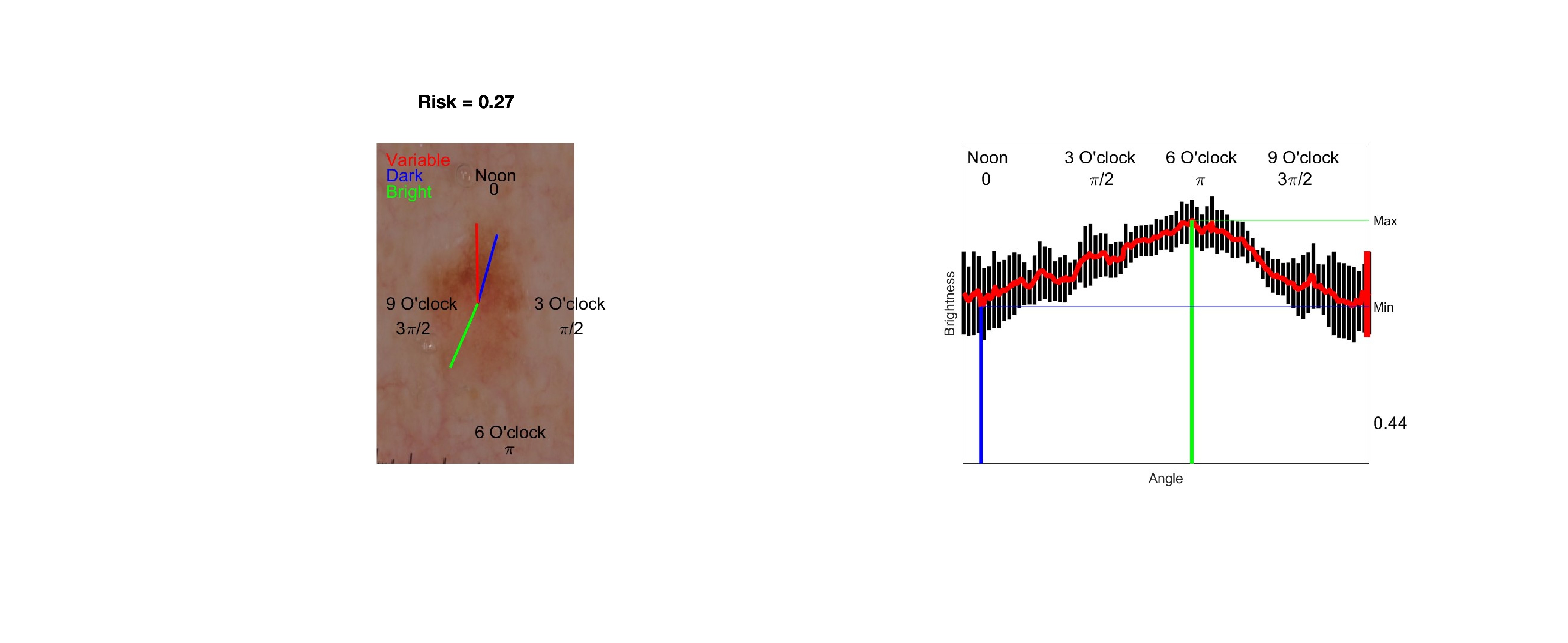

Supplement: Supplementary file 1 [file cancers-16-03077-s001.zip › cancers-3154863-supplementary/Supplementary File 2/032B.jpg]

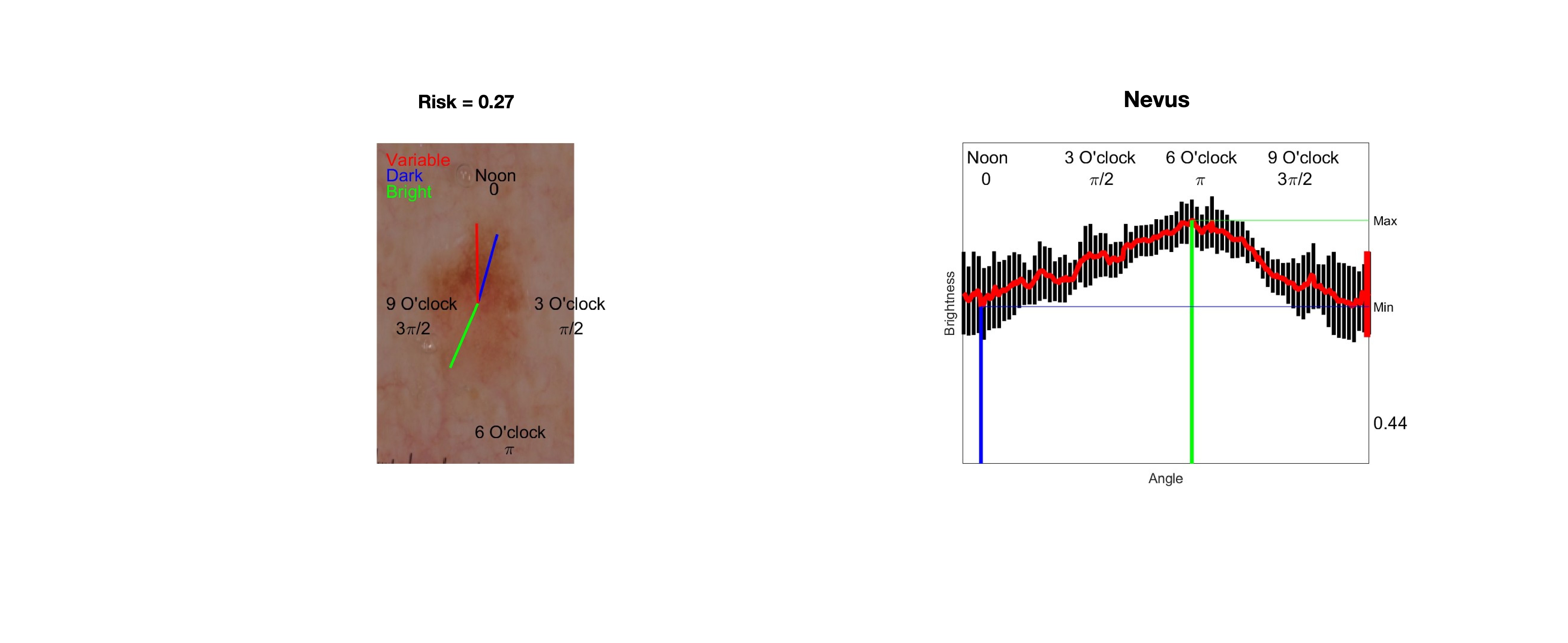

Supplement: Supplementary file 1 [file cancers-16-03077-s001.zip › cancers-3154863-supplementary/Supplementary File 2/032C.jpg]

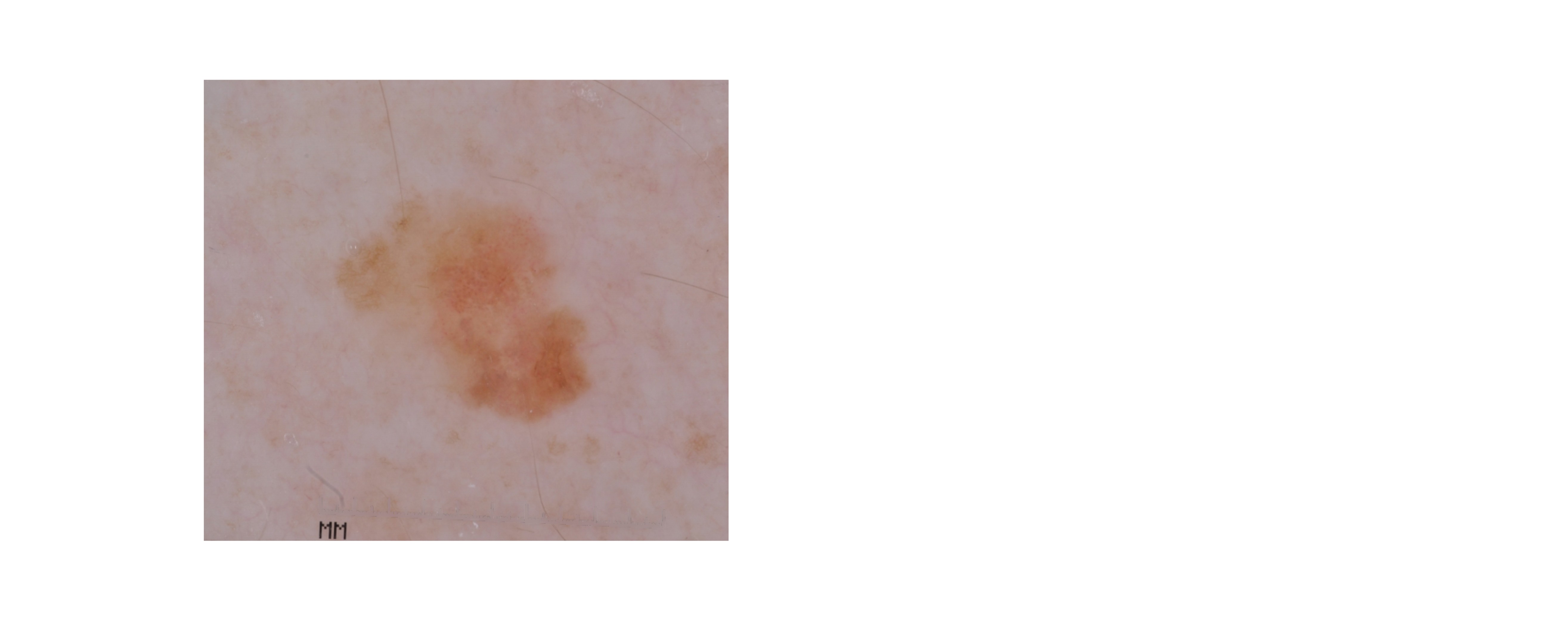

Supplement: Supplementary file 1 [file cancers-16-03077-s001.zip › cancers-3154863-supplementary/Supplementary File 2/033A.jpg]

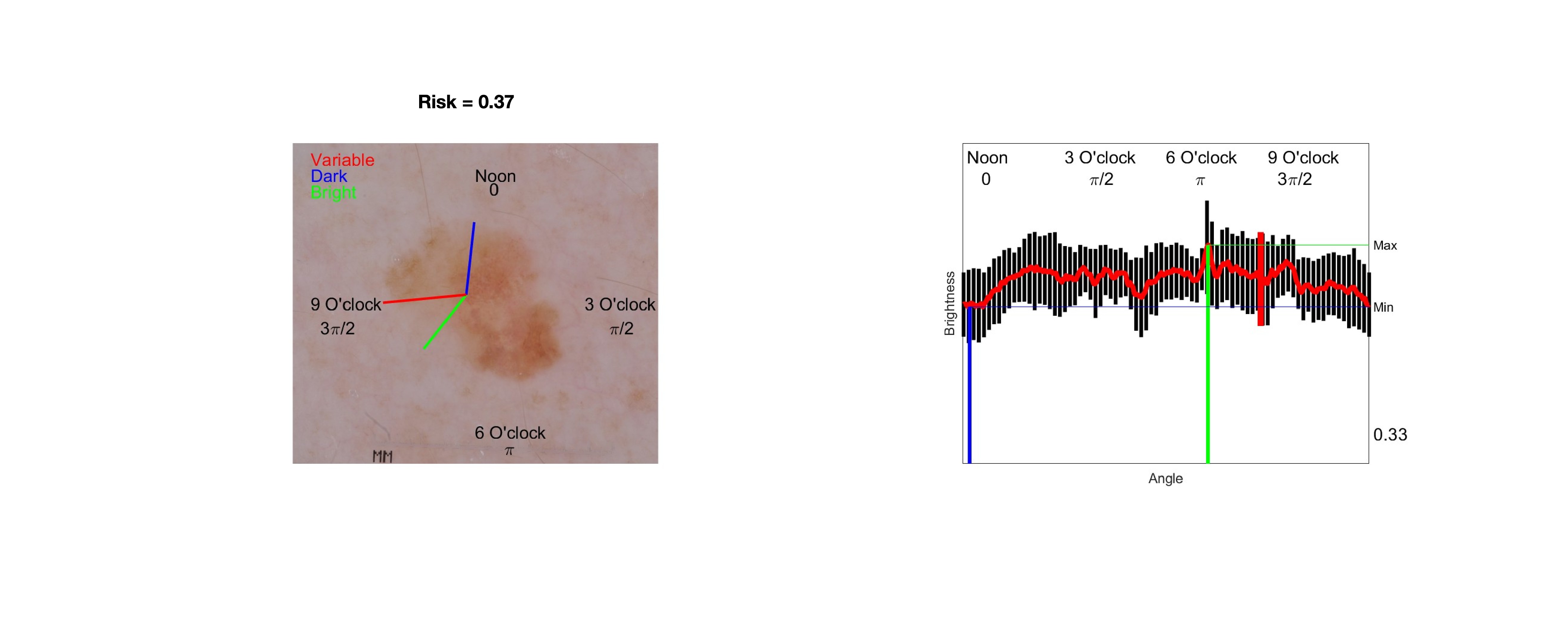

Supplement: Supplementary file 1 [file cancers-16-03077-s001.zip › cancers-3154863-supplementary/Supplementary File 2/033B.jpg]

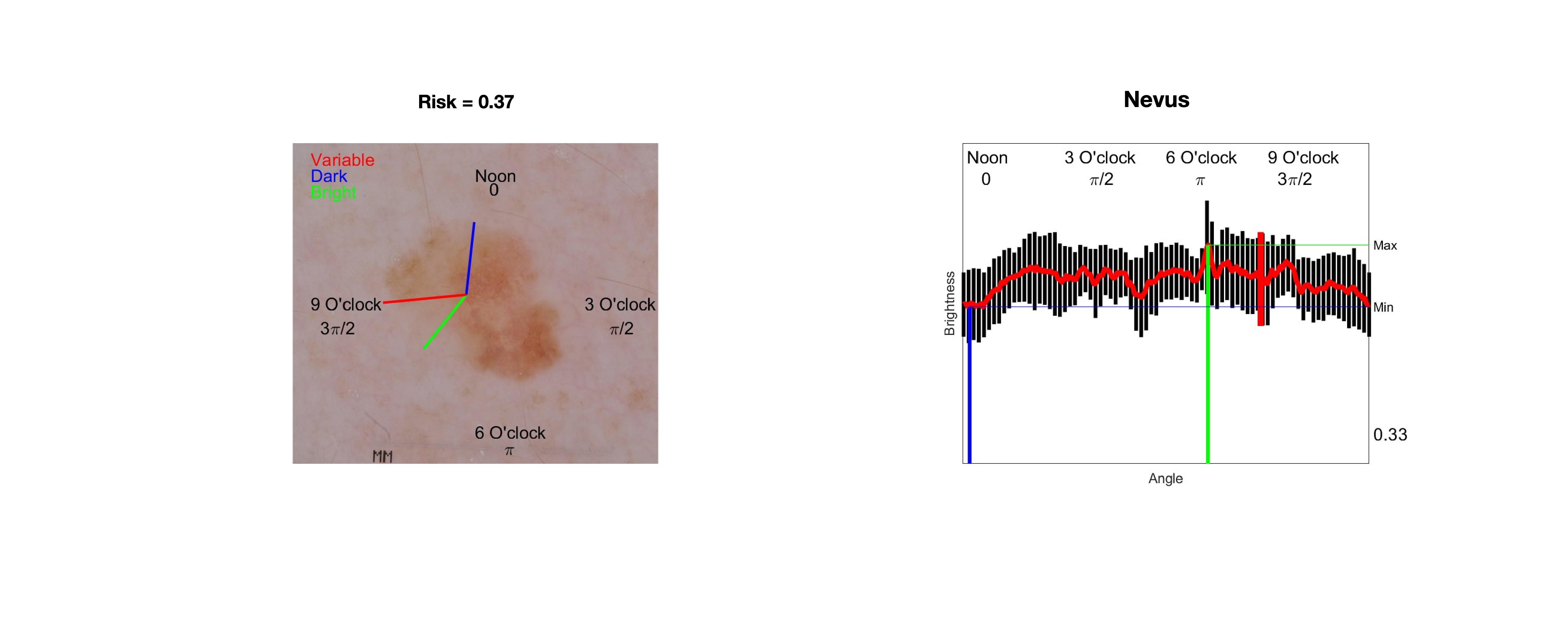

Supplement: Supplementary file 1 [file cancers-16-03077-s001.zip › cancers-3154863-supplementary/Supplementary File 2/033C.jpg]

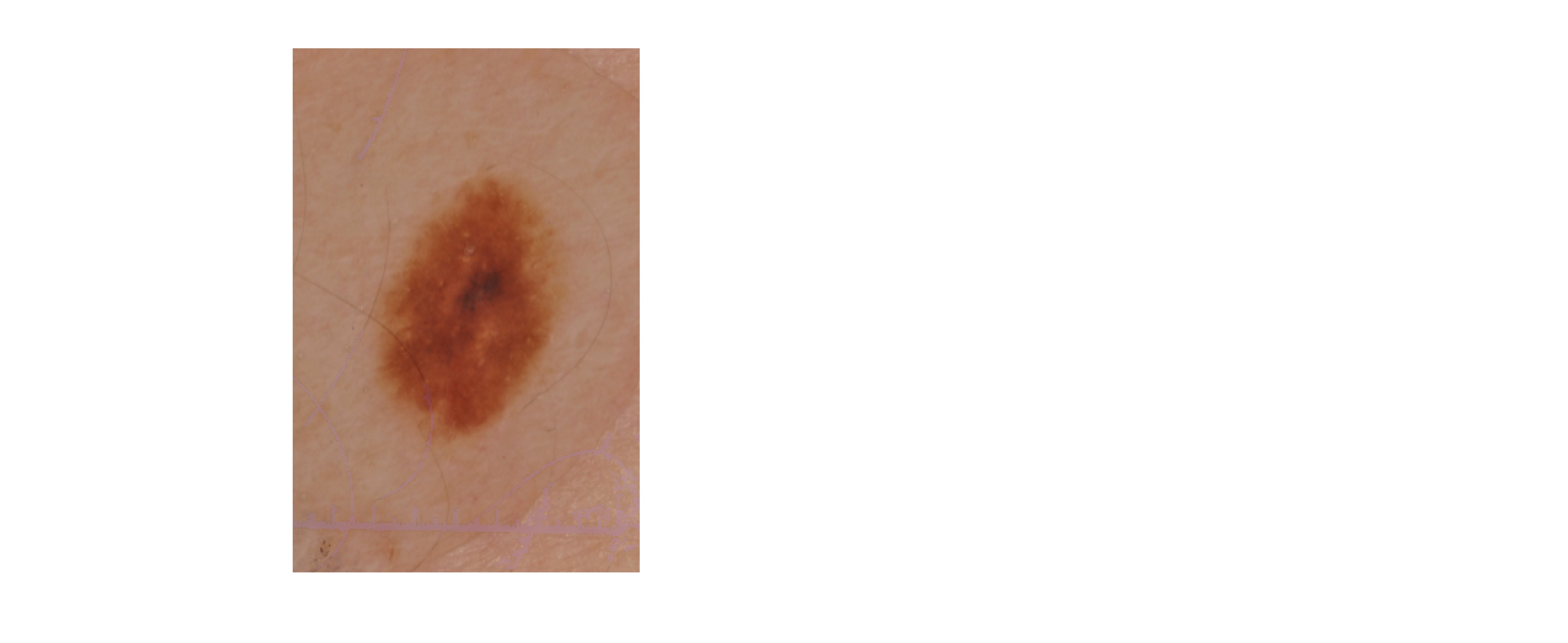

Supplement: Supplementary file 1 [file cancers-16-03077-s001.zip › cancers-3154863-supplementary/Supplementary File 2/034A.jpg]
